# High-resolution genome-wide scan of genes, gene-networks and cellular systems impacting the yeast ionome

**DOI:** 10.1186/1471-2164-13-623

**Published:** 2012-11-14

**Authors:** Danni Yu, John M C Danku, Ivan Baxter, Sungjin Kim, Olena K Vatamaniuk, Olga Vitek, Mourad Ouzzani, David E Salt

**Affiliations:** 1 Department of Statistics, Purdue University, West Lafayette, IN, USA; 2 Institute of Biological and Environmental Science, University of Aberdeen, Scotland, UK; 3 USDA-ARS Plant Genetics Research Unit, Donald Danforth Plant Science Center, St. Louis, MO, USA; 4 Department of Crop and Soil Sciences, Cornell University, Ithaca, NY, USA; 5 Department of Computer Science, Purdue University, West Lafayette, IN, USA; 6 Cyber Center, Purdue University, West Lafayette, IN, USA; 7 Current address: Qatar Computing Research Institute, Qatar Foundation, Doha, Qatar

**Keywords:** Ionome, Yeast, Clustering, Network analysis, Mitochondria, Vacuole, ESCRT, Genome-wide, ICP-MS, Ionomics

## Abstract

**Background:**

To balance the demand for uptake of essential elements with their potential toxicity living cells have complex regulatory mechanisms. Here, we describe a genome-wide screen to identify genes that impact the elemental composition (‘ionome’) of yeast *Saccharomyces cerevisiae*. Using inductively coupled plasma – mass spectrometry (ICP-MS) we quantify Ca, Cd, Co, Cu, Fe, K, Mg, Mn, Mo, Na, Ni, P, S and Zn in 11890 mutant strains, including 4940 haploid and 1127 diploid deletion strains, and 5798 over expression strains.

**Results:**

We identified 1065 strains with an altered ionome, including 584 haploid and 35 diploid deletion strains, and 446 over expression strains. Disruption of protein metabolism or trafficking has the highest likelihood of causing large ionomic changes, with gene dosage also being important. Gene over expression produced more extreme ionomic changes, but over expression and loss of function phenotypes are generally not related. Ionomic clustering revealed the existence of only a small number of possible ionomic profiles suggesting fitness tradeoffs that constrain the ionome. Clustering also identified important roles for the mitochondria, vacuole and ESCRT pathway in regulation of the ionome. Network analysis identified hub genes such as *PMR1* in Mn homeostasis, novel members of ionomic networks such as *SMF3* in vacuolar retrieval of Mn, and cross-talk between the mitochondria and the vacuole. All yeast ionomic data can be searched and downloaded at http://www.ionomicshub.org.

**Conclusions:**

Here, we demonstrate the power of high-throughput ICP-MS analysis to functionally dissect the ionome on a genome-wide scale. The information this reveals has the potential to benefit both human health and agriculture.

## Background

In addition to the assimilation of carbon and oxygen a vital function of all living cells is to control the accumulation from the environment of various chemical elements required for numerous essential biochemical processes, including incorporation into macromolecules (e.g. N, P, S, Se), as cofactors in enzymes (e.g. Cu, Fe, Mn, Zn, Mg), in cellular buffering (e.g. K), and as second messengers in signaling processes (e.g. Ca). Though essential for life, many of these elements are also potentially toxic. To balance the demand of these essential elements for normal growth and reproduction with their potential toxicity living cells have evolved complex regulatory mechanisms to control their uptake and accumulation. Identification of the genes and gene networks that control these regulatory processes is a critical first step to a complete understanding of the molecular mechanisms involved. Such an understanding will have many potential benefits, including in our understanding of human disease, and in the development of more nutritious food crops with increased concentrations of essential micronutrients (e.g. Fe and Zn), lower concentrations of potentially toxic trace elements (e.g. Cd and As), and that are produced using cropping systems that require less mineral nutrient inputs (e.g. N, P, K).

The yeast *Saccharomyces cerevisiae* has proved to be a powerful model for the study of mineral nutrient and trace element homeostasis, for recent reviews see [1-4]. The availability of full genome deletion and open reading frame (ORF) overexpression collections [5-7] have further enhanced the power of yeast as a model system, and these genome-wide tools have already been applied to the study of mineral nutrient and trace element homeostasis, indirectly by studying the growth effects of elevated transition metals [2,8], B [9], selenite [10], and Fe [11] and Zn [12] deficiency. Studies have also been undertaken in which accumulation of Fe [13], Cs, Sr [14], and P [15] have been directly quantified in yeast cells. In order to investigate the interactions between the mineral nutrient and trace element content of yeast (*aka* ‘ionome’ or ‘metallome’) an investigation of the simultaneous accumulation of multi-elements (Ca, Co, Cu, Fe, K, Mg, Mn, Ni, P, Se, Na, S and Zn) has also been performed [16].

Here, we build on these previous studies and extend them by directly measuring, by inductively coupled plasma-mass spectrometry (ICP-MS), the simultaneous accumulation of As, Ca, Cd, Cl, Co, Cu, Fe, K, Mg, Mn, Mo, Na, Ni, P, S, Se, and Zn in the 4940 yeast strains in the haploid deletion collection, the 1127 essential genes in the diploid heterozygous knockout collection, and the 5798 genes in the open reading frame (ORF) over expression collection. Results of this study provide new data for the functional characterization of yeast genes of unknown function, provide new functional insight into genes with known functions, and provide a deeper understanding of the cellular systems and gene networks that are involved in controlling the yeast ionome. A publically accessible database at http://www.ionomicshub.org has also been developed to facilitate broad utilization of this large data resource.

## Results and discussion

### Genome-wide scan of genes impacting the yeast ionome

We quantified the concentration of Ca, Cd, Co, Cu, Fe, K, Mg, Mn, Mo, Na, Ni, P, S and Zn (referred to as the ionome) in the 4940 viable mutant strains of yeast in which the open reading frames (ORFs) in haploid cells have been disrupted one at a time (referred to as knockouts abbreviated to KO), and in the 1127 viable mutant strains where a single copy of the ORFs in diploid cells have been disrupted to produce a heterozygous knockout (referred to as knockouts diploid abbreviated to KOd). Further, we also quantified the concentrations of Ca, Cd, Co, Cu, Fe, K, Mg, Mn, Mo, Na, Ni, P, S and Zn in the 5798 viable strains of yeast where each ORF in the genome has been ectopically overexpressed one at a time (referred to as over expression and abbreviated to OE). Improvements in our analytical methodologies also allowed use to additionally quantify As, Se and Cl in the OE collection. The data was collected as described by Danku et al., [17] and each data set (KO, KOd and OE) separately normalized and summarized over 4, 8 or 16 biological replicates. The data was analyzed statistically using linear mixed-effect models to determine strains and elements that significantly change in abundance as compared to the median trend after manually filtering for quality control and controlling the False Discovery Rate (FDR) [18]. Of the 4940 genes tested in the haploid knockout collection the loss of function of 584 of these genes (Additional file 1: Table S1) had a significant effect on the concentration of at least one element in the yeast ionome. The loss of function of 35 genes (Additional file 1: Table S2), out of the 1127 tested in the diploid heterozygous knockout collection, were also found to significantly affect the concentration of at least one element in the yeast ionome. Together, this establishes that 10% of the ORFs in the complete genome of yeast are involved directly or indirectly in controlling the ionome (Table 1). The ectopic over expression of 446 ORFs (Additional file 1: Table S3) out of the 5798 tested were also found to significantly affect the concentration of at least one element in the yeast ionome, establishing that ectopic over expression of 8% of the total ORFs in yeast affect the yeast ionome (Table 1). Of the genes we observed to significantly affect the yeast ionome from the KO, KOd and OE sets, we observe that 42 occur in both the knockout (KO + KOd) and OE sets (Additional file 1: Table S4). Using the hypergeometric test we establish that this overlap is not significant (p-value=0.4638). This supports the conclusion that on a genome-wide scale for control of the ionome in yeast loss and gain of gene function produce different, non-overlapping affects.

**Table 1:** Summary of yeast genes interrogated

	***Total genes***	***Significant genes***	***Group A***	***Group B***	***Group C***
Knockout(haploid)	4940	584	36	516	32
Knockout(diploid)	1127	35	7	25	3
Overexpression	5798	446	80	330	36

**Figure 1:**
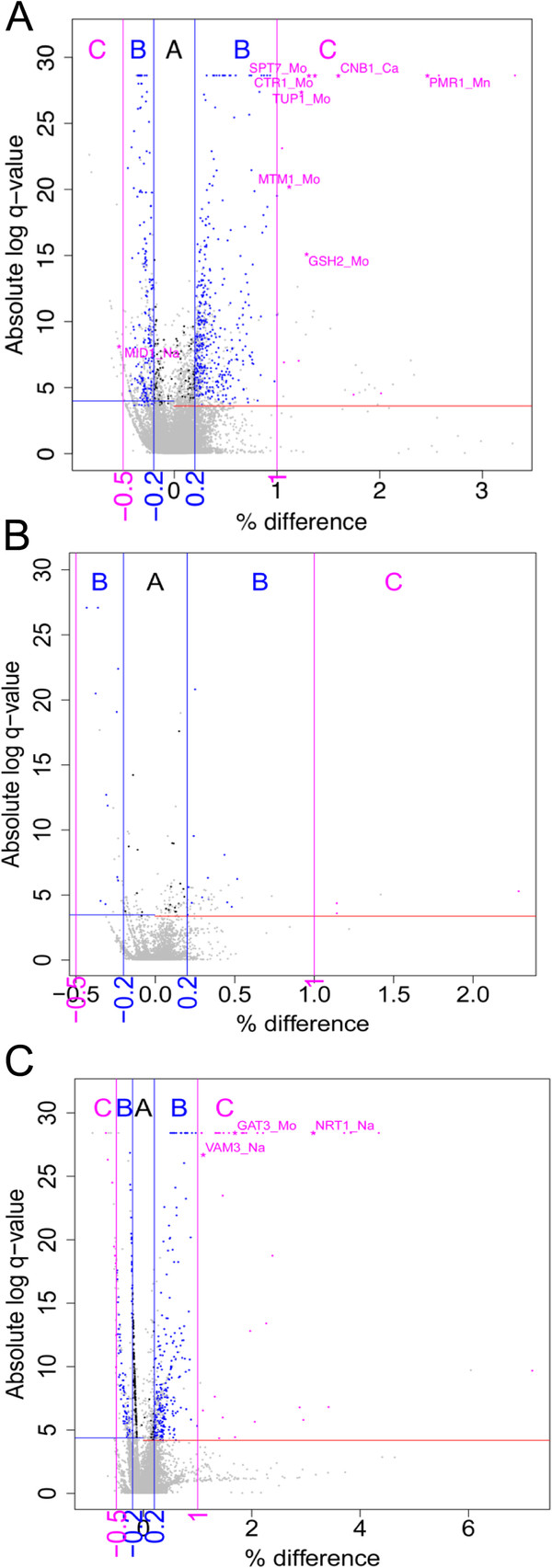
**Visualization of the association between biological significance and statistical significance using volcano plots for the KO (Figure**1**A), KOd (Figure**1**B) and OE (Figure**1**C) datasets.** In the plots the X-axis represents the percentage difference in elemental abundance and the Y-axis represents the absolute log of the q-values of the modified Z values. Q-values are the false discovery rate adjusted p-values and range between 0 and 1. Data points are colored in gray if elemental abundances were not statistically altered after gene perturbation. When elemental abundances were statistically altered data points are clustered into three groups according to the percentage difference. The three groups are group A (black dots) with percentage difference between −0.2 and 0.2, group B (blue dots) with percentage difference between −0.5 and −0.2 or between 0.2 and 1, and group C (magenta dots) with differences than −0.5 or greater than 1. Two blue vertical lines and two magenta vertical lines are added to represent the cutoffs for the three groups. The blue and red horizontal lines correspond to the negative and positive cutoff for statistical significance with 95% confidence. Labels of gene name and element names are added to the plots to identify those data points (pink stars) associated with genes from group C of either known function (Table 2) or proposed (Table 3) function in regulating the yeast ionome.

Group A - practical significance cutoff at −20 to 20%, Group B - practical cut off at 20 to 100% & -20 to −50%, Group C - practical cut off at 100% and −50%. Groups represented in volcano plots (Figure 1).

To help identify those genes which have a practical impact on the yeast ionome we divided the lists of genes that have a statistically significant effect on the ionome, for each set (KO, KOd and OE), into three groups based on the magnitude of the percent changes from the mean of the normalized element abundance across all mutants. Group C contains genes that cause a ≥ 100% increase or ≤ −50% decrease in any of the elements we quantified, group B contains genes that cause 20 to 100% & -20 to −50% change, and group A −20% to 20% change. The percentage change was calculated as the difference between the normalized element abundance of a mutant and the normalized mean element abundance across all mutants, divided by the normalized mean element abundance across all mutants. To illustrate the distribution of genes in each of the groups A, B and C for each of the gene sets (KO, KOd and OE) we have plotted the magnitudes and statistical significance of the impact of each gene on all the measured elements in the ionome (Figure 1A – C). Both the loss of function (KO and KOd) and gain of function (OE) gene sets have a similar range of statistical significance (−log[q-value] 0 – 30) but the effect size (percentage change) for the knockout (KO and KOd) and OE sets differ, with the OE set producing a larger range of effect sizes skewed towards larger positive effects. Arsenic, Cl and Se were included in the analysis of the OE set but not the KO and KOd set. However, of the 21 data elements in the OE set with an effect size > 3 only two were for Se and one for Cl. From this we determine that the increase in the effect size of the outliers in the OE data set is not an artifact due to the addition of As, Cl and Se to the OE data set.

### Validation of the identified gene sets against existing knowledge

To help validate the loss of function set of genes we observed to have a significant effect on the yeast ionome we compared our set to that generated by Eide et al., [16]. In the screen of a whole-genome homozygous diploid knockout collection, at a first pass through the collection at n = 1 replicate samples per yeast strain, Eide and coworkers identified 233 genes that reproducibly affected the yeast ionome. Of these 233 genes we observe 61 to significantly affect the ionome in our KO dataset. Using the hypergeometric test we determined that this overlap is highly significant (p-value = 5.236594e-10) and would not be expected to occur if the genes were selected at random. Of the overlapping 61 genes 57 fall into our middle effect Group B set of genes, with only 3 genes in our small effect Group A set of genes, and 1 in our large effect Group C set of genes. Such a significant overlap in the knockout gene set reported by Eide et al., [16] to affect the ionome and the knockout gene set we report here strongly validates our approach and gives us confidence that the more extensive high-resolution genome wide scan of the ionome reported here (n = 4 replicate samples per yeast strain) will reveal novel and significant insight into the regulation of the yeast ionome. Importantly, 16 of the large effect genes in our KO group C are altered in Mo, an element not measured by Eide and co-workers, explaining why these large effect genes were not identified by Eide et al., [16].

To further validate our sets of significant genes that affect the ionome in yeast we investigated if genes known to be involved in regulation of various elements in the ionome from the current literature also occurred in our gene sets. Based on the literature we generated a list of genes known to be involved in the homeostasis of the essential mineral nutrients Fe, Cu, Zn, Mn, Mg, Ca, K and Na in yeast [1,4,19,20] (Additional file 1: Table S5). Of this list of 104 genes known to be involved in mineral nutrient homeostasis in yeast we identified loss of function of 26 to have a significant impact on the yeast ionome (Table 2). A further 8 were found to have a significant effect on the ionome when overexpressed (Table 2). There are multiple explanations why we only identified about 1/3^rd^ of the genes known to be involved in mineral nutrient homeostasis in yeast. Particularly significant is the fact that many of the 104 genes known to be involved in mineral nutrient homeostasis are primarily involved during growth under nutrient deficiency and not the sufficiency conditions we performed our experiments under. Further, even though a gene is known to be involved in mineral nutrient homeostasis its loss of function may be compensated for by other genes and thus might not in itself be manifest as a change in the ionome as measured in our experiment.

**Table 2:** Genes known to be involved in mineral nutrient homeostasis that also have an ionomic phenotype expressed as moderated Z-score

***Significant ionomic changes in yeast with gene knockout in haploid background***																				
**Known to regulate**	**Gene**	**Gene**	**Group**	**Ca**	**Cd**	**Co**	**Cu**	**Fe**	**K**	**Mg**	**Mn**	**Mo**	**-**	**Ni**	**P**	**S**	**Zn**			
Ca	YNL291C	MID1	C	5.14	-	-	-	-	-	-	-	-	-	-	-	-	-			
Ca	YGL167C	PMR1	C	-	*note 1*	-	-	-	-	-	15.49	-	-	-	-	-	-			
Ca	YKL190W	CNB1	C	15.95	-	-	-	-	-	-	-	-	-	-	-	-	-			
K/Na	YDR456W	NHX1	B	-	3.98	-	-	-	-	-	−3.63	-	-	-	-	-	-			
P	YJL012C	VTC4	B	-	-	-	-	-	-	−8.45	-	-	-	-	−6.10	-	-			
P	YER072W	VTC1	B	-	-	-	-	-	-	−8.94	-	-	-	-	−8.59	-	-			
S	YOL049W	GSH2	C	-	5.42	-	-	-	-	-	-	6.15	-	-	-	-	-			
S	YJR137C	MET5	B	-	-	-	-	-	-	-	5.53	-	-	-	-	-	-			
Fe	YHL040C	ARN1	B	-	4.66	-	-	-	-	-	-	-	-	-	-	-	-			
Fe	YMR058W	FET3	B	-	-	-	-	-	-	-	-	6.50	-	-	-	-	-			
Fe	YGL220W	FRA2	B	-	-	-	-	-	−3.39	-	-	-	-	-	-	-	-			
Fe	YER145C	FTR1	B	-	-	-	-	-	-	-	-	5.01	-	-	-	-	-			
Fe	YOL122C	SMF1	B	-	-	-	-	-	-	-	−7.18	-	-	-	-	-	-			
Fe	YLR034C	SMF3	B	-	4.49	-	-	-	-	-	8.49	-	-	-	-	-	-			
Cu	YPR124W	CTR1	C	-	-	-	-	-	-	-	-	10.37	-	-	-	-	-			
Cu	YHR175W	CTR2	B	-	-	-	-	-	−3.91	-	-	-	-	-	-	-	-			
Cu	YMR021C	MAC1	B	-	-	-	-	-	-	-	-	-	3.61	-	-	-	-			
Mn	YBR290W	BSD2	B	-	9.30	-	-	-	-	-	9.15	-	-	-	-	-	-			
Mn	YGR257C	MTM1	C	-	-	-	-	-	-	-	-	6.97	-	-	-	-	-			
Mn	YGL167C	PMR1	C	-	*note 1*	-	-	-	-	-	15.49	-	-	-	-	-	-			
Mn	YOL122C	SMF1	B	-	-	-	-	-	-	-	−7.18	-	-	-	-	-	-			
Mn	YHR050W	SMF2	B	-	-	-	-	-	-	-	−7.40	-	-	-	-	-	-			
Zn	YJL056C	ZAP1	B	3.72	−4.73	−3.79	-	-	-	-	-	-	-	-	-	-	-			
Zn	YKL175W	ZRT3	A	-	3.76	-	-	-	-	-	-	-	-	-	-	-	-			
***Significant ionomic changes in yeast with single copy gene knockouts in diploid background***																				
**Known to regulate**	**Gene**	**Gene**	**Group**	**Ca**	**Cd**	**Co**	**Cu**	**Fe**	**K**	**Mg**	**Mn**	**Mo**	**Na**	**Ni**	**P**	**S**	**Zn**			
Mn	YER125W	RSP5	B	4.05	-	-	-	-	-	-	-	-	-	-	-	-	-			
Mg	YOL130W	ALR1	B	-	-	-	-	-	-	−3.24	3.65	-	-	-	-	-	-			
***Significant ionomic changes in yeast with single open reading frame over expressed***																				
**Known to regulate**	**Gene**	**Gene**	**Group**	**As**	**Ca**	**Cd**	**Cl**	**Co**	**Cu**	**Fe**	**K**	**Mg**	**Mn**	**Mo**	**Na**	**Ni**	**P**	**S**	**Se**	**Zn**
Ca	YGL006W	PMC1	B	-	4.23	-	-	-	-	-	-	-	-	-	-	-	-	-	-	-
Ca	YKL159C	RCN1	A	-	-	-	-	-	-	-	-	-	-	-	-	-	-	-	-	−5.90
Mg	YOR334W	MRS2	A	-	-	-	-	-	-	-	-	-	-	-	-	-	-	-	-	−4.34
Mg	YFL050C	ALR2	B	-	-	-	-	-	-	-	-	9.62	-	-	-	6.60	-	-	-	-
P	YPL031C	PHO85	B	-	-	-	-	-	-	-	-	5.85	-	4.93	-	4.52	-	-	-	-
P	YML123C	PHO84	B	-	-	-	-	-	-	-	-	-	-	-	-	-	-	-	-	−5.30
P	YNR013C	PHO91	A	-	-	-	-	-	-	-	-	-	-	-	-	-	-	-	-	−6.27
Cu	YPR124W	CTR1	B	-	-	-	-	-	5.60	-	-	-	-	-	-	-	-	-	-	-

note 1: YGL168W that overlaps PMR1 also has significant increase in Cd, and YGL167C is also elevated in Cd but just below the significance cut off.

**Figure 2:**
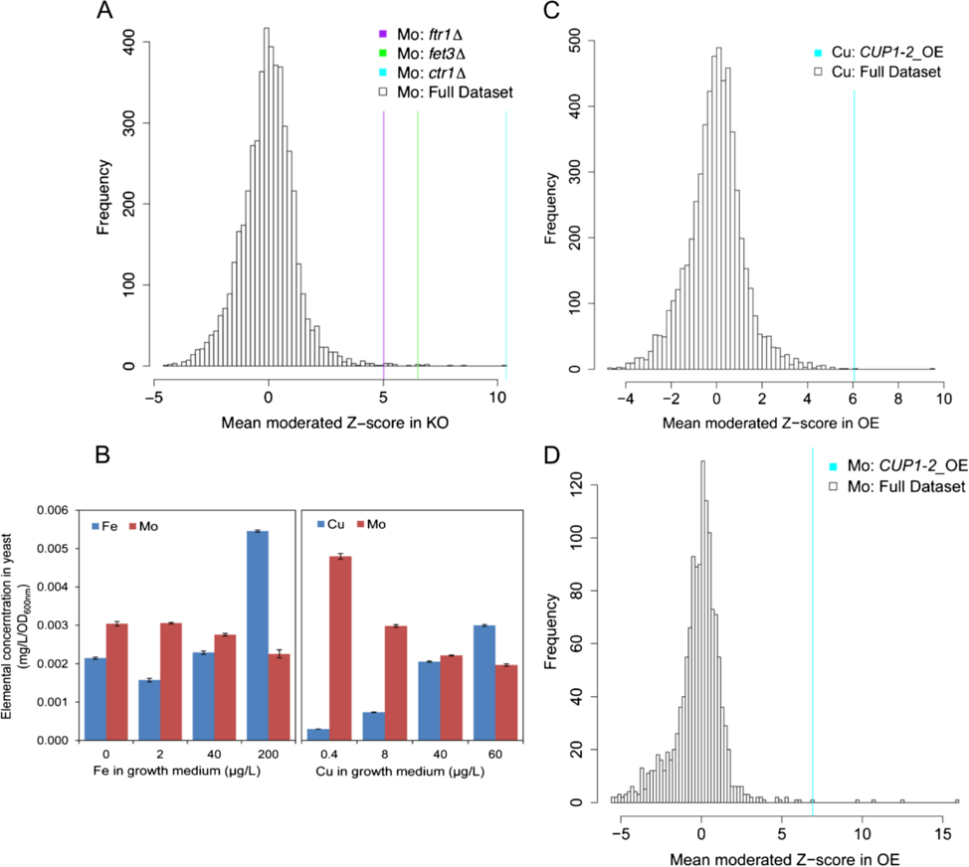
**Specific elemental abundances in selected loss of function mutants.** Abundance of Mo in yeast *ftr1Δ*, *fet3Δ*, *ctr1Δ* loss of function mutants compared to the distribution of abundances in the full KO population (**A**). Accumulation of Mo, Fe and Cu in yeast cultured in medium containing different concentrations of Fe or Cu (**B**). Data represents the mean (n = 6) ± SE and is a single experiment representative of 3 replicates. Abundance of Cu (**C**) and Mo (**D**) in yeast when *CUP1* was over expressed compared to the distribution of abundances in the full OE population.

Interestingly, we observed a high level of Mo accumulation in strains with loss of function alleles of several known Fe accumulation genes (*FET3*, *FRA2* and *CTR1*) (Table 2, Figure 2A) even though no changes in Fe accumulation are observed. *FTR1* and *FET3* encode the high affinity Fe transporter and ferric chelate reductase that work together for high-affinity Fe uptake [21]. *CTR1* encodes the high affinity Cu transporter and is responsible for Cu uptake [22,23], which is required for the function of Ftr1. Therefore, without a functional *CTR1* gene yeast have impaired high-affinity Fe uptake. Given the critical role these genes play in Fe homeostasis we might have expected to see a change in Fe accumulation in loss of function mutants of these genes. However, uptake of Fe by the low affinity Fet4 transporter may have compensated. The connection of Fe homeostasis and Mo accumulation was surprising since yeast do not require Mo [24] unlike plants in which a connection between Fe and Mo has been observed [25]. In the wild-type BY4741 with low Fe or Cu in the growth media we also observe an increase in Mo accumulation (Figure 2B), supporting the link between reduced Cu uptake, diminished high-affinity Fe uptake and increased Mo accumulation. Also, ectopic over expression of *CUP1* (Cu-binding metallothionein) causes a specific accumulation of Cu and Mo (Figure 2C & D). In this situation we might expect that the elevated Cu is bound to Cup1 causing Fe deficiency via inactivation of Ftr1-Fet3 high affinity uptake system. The Mo accumulation in this strain also supports the link between Fe-deficiency and Mo accumulation. One speculative explanation for the connection between Mo accumulation and Fe-deficiency could be increased adsorption of molybdate to positively charged components of the yeast cell wall such as Fe-oxides and Fe-phosphate. During Fe-deficiency and growth on glucose yeast switch from respiration to fermentation, for review see [3], causing enhanced acidification of the growth media which would promote increased adsorption of molybdate to Fe-oxides on the cell wall. Another possibility is that in response to Fe-deficiency FeMoO_4_ uptake is stimulated by an unidentified transporter, in a similar way to the proposed transport of MnHPO_4_ by the phosphate transporter Pho84 reviewed in [26].

**Figure 3:**
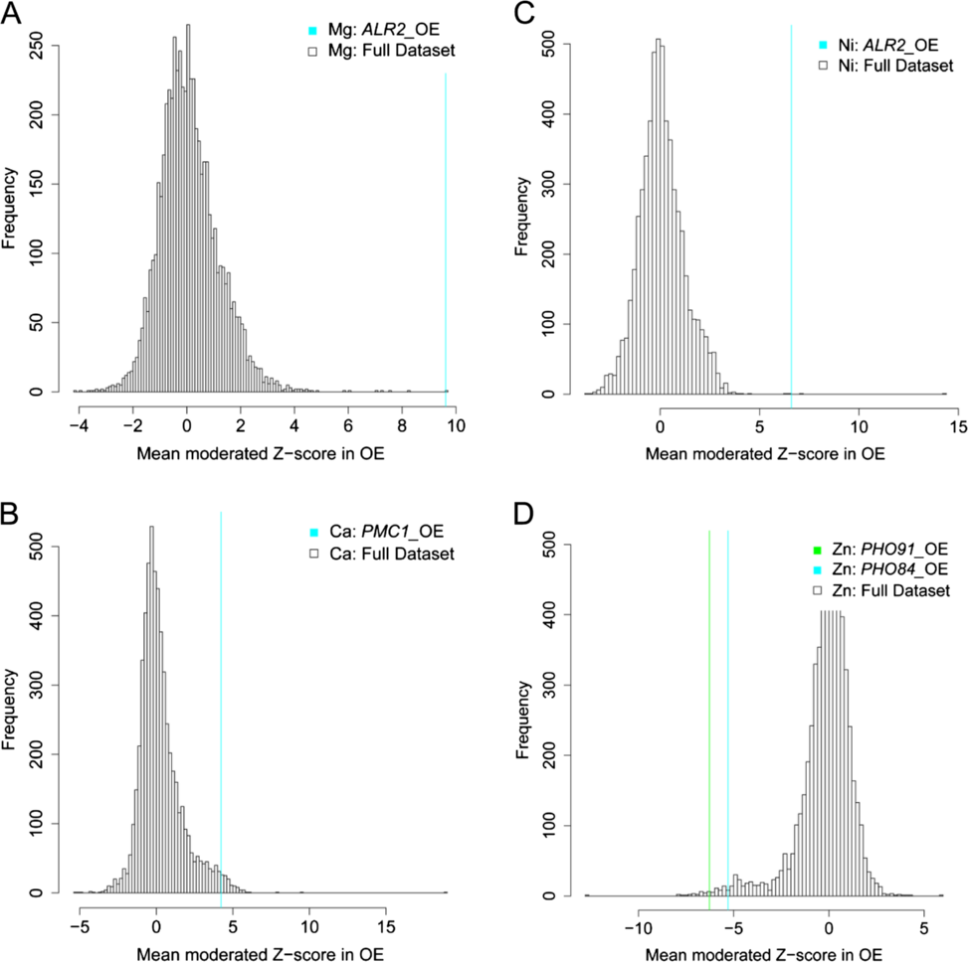
**Specific elemental abundances in selected over expression mutants.** Abundance of Mg (**A**) and Ni (**C**) when *ALR2* was over expressed, abundance of Ca (**B**) when *PMC1* was over expressed, and abundance of Zn (**D**) when *PHO91* and *PHO84* were over expressed all compared to the distribution of abundances in the full OE population.

Over expression of the high affinity Cu transporter *CTR1* caused a significant and specific increase in Cu in the yeast, suggesting that this transporter can accumulate when over expressed, even though it is known to be post-transcriptionally degraded in high Cu concentrations [27,28]. This is similar to the Ca transporters Pmc1 and the putative Mg transporter Alr2 which also cause specific increases in Ca and Mg (and Ni as Mg transporters are known to also transport Ni) when over expressed (Figure 3A - C). However, this contrasts with Fe, Zn, Mn, K and Na for which we find that over expression of known transporters does not affect the accumulation of these elements. This buffering is possibly explained by compensatory changes in post-transcriptional, translational or post-translational processes, or expression of other genes required for homeostasis of these elements. We do observe an effect of over expression of genes encoding the high affinity and low affinity phosphate transporters Pho84 and Pho91 on the yeast ionome, causing a significant reduction in Zn (Figure 3D) and with a trend towards elevated P.

**Figure 4:**
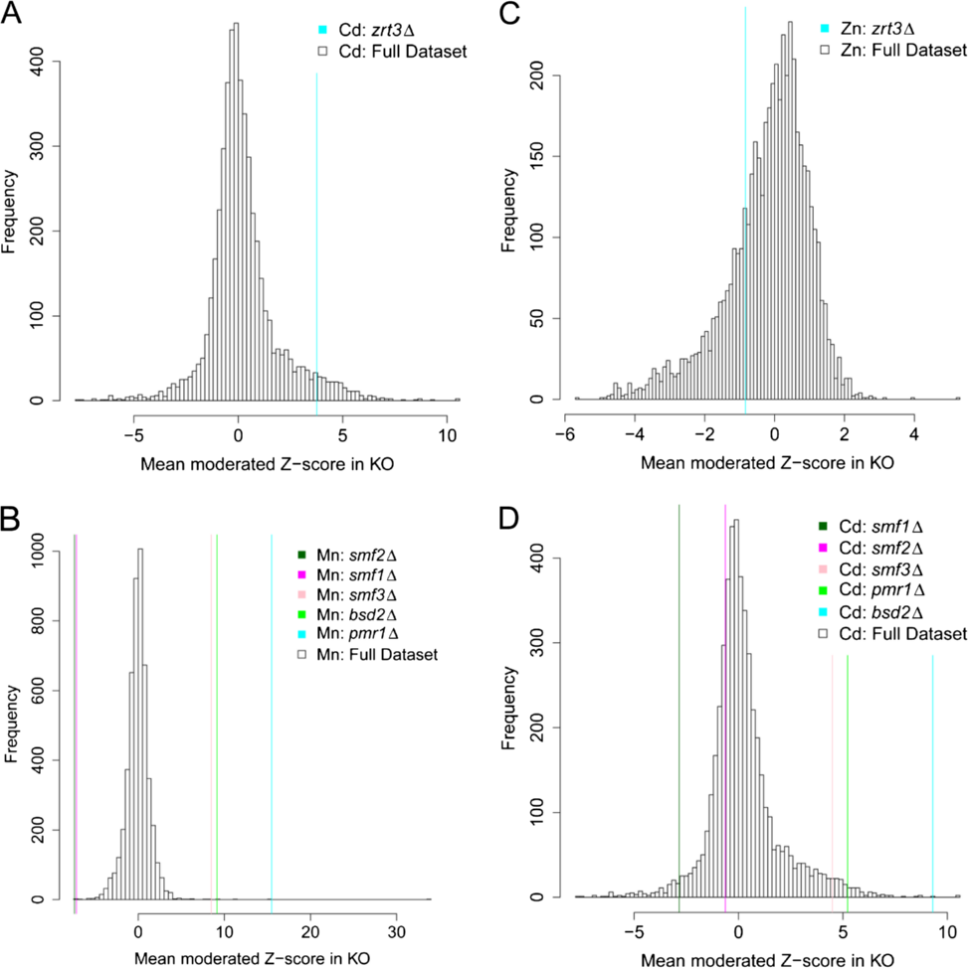
**Specific elemental abundances in selected loss of function mutants.** Abundance of Cd (**A**) and Zn (**C**) in *zrt3Δ* loss of function mutants, and abundance of Mn (**B**) and Cd (**D**) in *smf1Δ*, *smf2Δ*, *smf3Δ*, *bsd2Δ* and *pmr1Δ* loss of function mutants all compared to the distribution of abundances in the full KO population.

Loss of function of the Zn regulated transcription factor Zap1 causes reductions in Cd and Co, elevated Ca, and also shows a tendency for reduced Zn which is just below the significance cut off. Zap1 is involved in activating expression of *ZRT1* and *ZRT2* genes that encode the Zn transporters responsible for Zn uptake across the plasma membrane. Such effects of the loss of function of *ZAP1* suggest that under the conditions of our experiments one or more of these Zn transporters plays an important role in transporting Zn, Cd and Co. The connection between loss of expression of these Zn transporters and increased Ca is unknown. However, both Zn deficiency and loss of function of the Zn transporter *ZIP2* are known to cause elevated Ca in mice embryos [29]. Further, loss of function of the Zn transporter *ZRT3*, known to be involved in retrieval of Zn from the vacuole, causes increased Cd but no change in Zn (Figures 4A & C). At the low Zn concentration used in our experiments (6 μM) and based on the previous data of MacDiarmid et al., [30] we would not expect to see a difference in Zn accumulation in *zrt3Δ*. However, our observation that Cd accumulates in *zrt3Δ* suggests that Zrt3 may also transport Cd out of the vacuole. Loss of function of *ZRT3* is also known to limit the ability of yeast to mobilize Zn from the vacuole. This is thought to cause a compensatory increase in the expression of the genes encoding the plasma membrane Zn transporters Zrt1 and Zrt2 [30]. Increased activity of the Zn transporters Zrt1 and Zrt2 in *zrt3Δ* may therefore also be responsible for the increased accumulation of Cd we observe in *zrt3Δ*. Loss of function of *ZRT3* was also observed to cause an increase in the Cd sensitivity of this strain compared to the control (BY4741) when both were grown in liquid culture (Additional file 2: Figure S1), validating the elevated Cd observed in this strain. Accumulation of Cd in strains with perturbed Zn homeostasis suggests that Cd might be a good ‘tracer’ for perturbation of Zn homeostasis. This may be due to the fact that cells can sense and respond to perturbations in Zn homeostasis mechanisms in order to maintain cellular Zn balance. However, because cells do not specifically sense Cd they are unable to respond to changes in Cd accumulation directly.

Loss of function of the genes that encode the Mn influx transporters Smf1 and Smf2 both specifically cause low Mn (Figure 4B). The role of Smf1 in uptake of Mn across the PM is debated since the *smf1Δ* null mutant showed only a small reduction in Mn uptake [31], reviewed in [26]. However, our data suggests that both Smf1 and Smf2 play significant, and non-redundant, roles in Mn uptake. In contrast, loss of function of *BSD2* involved in recycling Smf1 and Smf2 from the PM, for review see [26], causes significant accumulation of Mn and Cd (Figures 4B & D). It is likely that loss of *BSD2* causes accumulation of Smf1 and Smf2 at the PM driving the over accumulation of both Cd and Mn because these transporters transport both Mn and Cd. Interestingly, we also observe that loss of function of *SMF3* causes significant Mn accumulation (Figure 4B). It has been proposed that Smf3 functions as a transporter that retrieves Fe from the yeast vacuole [32]. We observe no impact of loss of function of *SMF3* on Fe accumulation, though we do see a non-significant elevation of Mo (36%) consistent with a role in Fe homeostasis. However, we also see significant increases in Mn and Cd (90 and 23%, respectively) in strains where *SMF3* has been deleted, suggesting Smf3 is involved in retrieval of Mn and Cd from the vacuole (Figures 4B & D). It is currently unknown how Mn is released from the vacuole [26]. Loss of function of *PMR1*, which drives Golgi/ER sequestration and exocytosis of Mn, causes Mn over accumulation along with Cd (Figures 4C & D), most likely because Mn and Cd are now accumulating in the cytosol because they cannot be exported via exocytosis. Even though Pmr1 is a Ca^2+^ATPase we see no changes in Ca in *pmr1Δ*. Our data strongly supports the conclusion that both Mn and Cd are transported by Smf1, Smf2, Smf3 and Pmr1 confirming what has previously been shown for Pmr1 [33], and Smf1 [34].

The homozygous knockout of *ALR1* which encodes a plasma membrane Mg transporter is lethal, establishing its absolute requirement for survival. Interestingly, in the heterozygous knockout (KOd) of *ALR1* we observe a reduction in Mg accumulation, though it is just below the significance cutoff. Over expression of *ALR1* had no significant effect on Mg and Ni accumulation.

**Figure 5:**
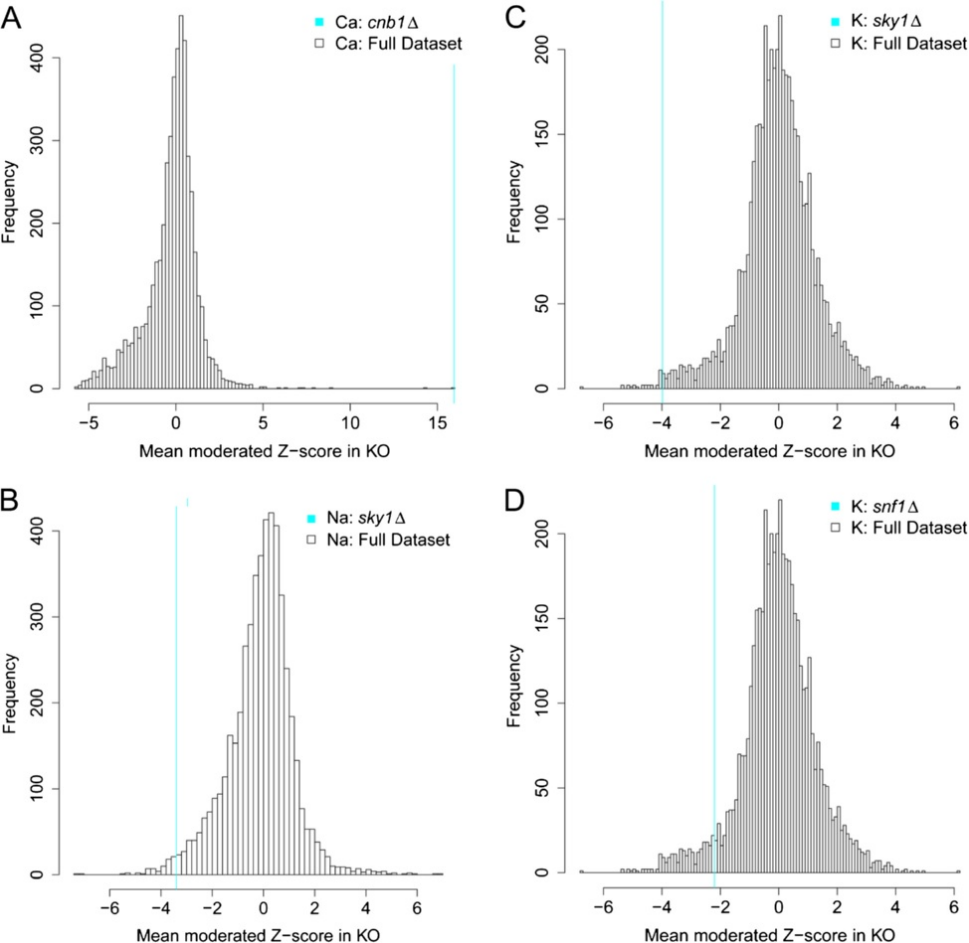
**Specific elemental abundances in selected loss of function mutants.** Abundance of Ca (**A**) in *cnb1Δ* loss of function mutant, abundance of K (**C**) and Na (**B**) in *sky1Δ* loss of function mutant, and abundance of K (**D**) in *snf1Δ* loss of function mutant all compared to the distribution of abundances in the full KO population.

We observe the loss of function of *CNB1*, encoding the regulatory subunit of calcineurin, to cause a specific elevation of Ca (Figure 5A). The Cnb1 protein is known to associate with calcineurin and is required for function [35]. Calmodulin with bound Ca binds and activates the calcineurin complex which then negatively regulates the vacuole Ca/H exchanger Vcx1, and positively regulates the vacuolar Ca ATPase Pmc1 and ER and Golgi localized Pmr1. Loss of function of *CNB1* may therefore drive over accumulation of Ca through the increased activity of Vcx1, though over expression of *VCX1* has no effect on Ca accumulation.

Loss of function of either *TRK1* or *TRK2* encoding K uptake transporters has no effect on the accumulation of K. The serine/arginine-rich (SR) protein kinase Sky1 has been implicated in regulating K uptake via Trk1 and 2 [36,37]. Interestingly, we observe a reduction in K in the *sky1Δ* mutant (Figure 5C) consistent with Sky1 playing a regulatory role in K uptake. However, Forment et al., [36] observed increased K uptake in K-starved *sky1Δ* cells. The difference between these two experiments may be explained by the fact that yeast cells were K-starved in the Forment and coworkers experiments whereas our experiment was performed under K replete conditions. We also observed that the *sky1Δ* mutant has reduced Na (Figure 5B) and this fits with the enhanced Na-tolerance and reduced uptake previously observed in this mutant [36]. The *snf1Δ* null mutant grown with high glucose has been previously shown to have a deficiency in K uptake [38] and we also see reduced K in this mutant (Figure 5D). Under high glucose Snf1 is dephosphorylated by Reg1/Glc7 and deactivated causing glucose-repression of many genes. The low K observed in *snf1Δ* must therefore be due to a function of the non-phosphorylated form of Snf1. It is possibly caused by the fact that the non-phosphorylated form of Snf1 activates Trk1,2 mediated K-uptake, thus the *snf1Δ* mutant has reduced Trk1,2 activity and reduced K-uptake [38]. Reg1 is known to be involved in regulating the phosphorylation of Snf1. In a *reg1Δ* mutant Snf1 is hyperphosphorylated and we observe an increase in K in these cells, though the mechanism is unclear.

We observed that the over expression of *PHO85* encoding a cyclin-dependant kinase causes a reduction in Zn, similar to that observed for the overexpression of *PHO84* and *PHO91* both encoding P transporters (Figure 3D). This is consistent with a role for Pho85 in nutrient sensing [39].

### Highlighted genes with novel function in regulation of the ionome

In Table 3 we have selected a limited number of genes from groups B and C which show a significant and previously unknown effect on regulating accumulation of specific elements (Ca, Na, Mg, K, S, Mo, Mn, Cu, and Cd), or groups of elements we observe to be related (e.g. Mg & P, Mn & Cd) in the yeast ionome. For a complete list of genes that significantly affect the yeast ionome see Additional file 1: Table S1 - S3. Such genes provide a source of potentially novel hypotheses regarding the way these mineral nutrients are regulated in yeast, and hopefully will lead to the development of follow up experiments to test these hypotheses.

**Table 3:** Potential novel genes involved in mineral nutrient and trace element homeostasis in yeast with ionomic phenotypes expressed as moderated Z-scores

***Significant ionomic changes in yeast with gene knockout in haploid background***																				
**Element being regulated**	**Gene**	**Gene**	**Group**	**Ca**	**Cd**	**Co**	**Cu**	**Fe**	**K**	**Mg**	**Mn**	**Mo**	**Na**	**Ni**	**P**	**S**	**Zn**			
Ca	YNL323W	LEM3	B	7.15	-	-	-	-	-	-	-	-	-	-	-	-	-			
Ca	YOL052C	SPE2	B	6.32	-	-	-	-	-	-	-	-	-	-	-	-	-			
Ca	YGR162W	TIF4631	B	4.34	-	-	-	-	-	-	-	-	-	-	-	-	-			
Ca	YBL047C	EDE1	B	4.29	-	-	-	-	-	-	-	-	-	-	-	-	-			
Ca	YBL094C		B	4.06	-	-	-	-	-	-	-	-	-	-	-	-	-			
Na	YNR051C	BRE5	B	-	-	-	-	-	-	-	-	-	5.87	-	-	-	-			
Na	YOR039W	CKB2	B	-	-	-	-	-	-	-	-	-	4.33	-	-	-	-			
K	YJL179W	PFD1	B	-	-	-	-	-	4.99	-	-	-	-	-	-	-	-			
K	YLR242C	ARV1	B	-	-	-	-	-	4.31	-	-	-	-	-	-	-	-			
Mg & P [Ni trend]	YPL179W	PPQ1	B	-	-	-	-	-	-	19.50	-	-	-	-	5.70	-	-			
Mg & P [Ni trend]	YGR129W	SYF2	B	-	-	-	-	-	-	9.28	-	-	-	-	8.61	-	-			
S	YBR279W	PAF1	B	-	-	-	-	-	-	-	-	-	-	-	-	8.15	-			
S	YPL178W	CBC2	B	-	-	-	-	-	-	-	-	-	-	-	-	7.94	-			
S	YIL097W	FYV10	B	-	-	-	-	-	-	-	-	-	4.79	-	-	7.88	-			
S	YIL165C		B	-	-	-	-	-	-	-	-	-	-	-	-	7.00	-			
S	YIL155C	GUT2	B	-	-	-	-	-	-	-	-	-	-	-	-	6.71	-			
Mo [surrogate Fe]	YBR081C	SPT7	C	-	-	-	-	-	-	-	-	8.47	-	-	-	-	-			
Mo [surrogate Fe]	YCR084C	TUP1	C	-	-	-	-	-	-	-	-	7.97	-	-	-	-	-			
Mo [surrogate Fe]	YGR257C	MTM1	C	-	-	-	-	-	-	-	-	6.97	-	-	-	-	-			
Mo [surrogate Fe]	YGL066W	SGF73	C	-	-	-	-	-	-	-	-	6.86	-	-	-	-	-			
Mo [surrogate Fe]	YBR112C	CYC8	C	-	-	-	-	-	-	-	-	6.73	-	-	-	-	-			
Mn & Cd	YLR034C	SMF3	B	-	4.50	-	-	-	-	-	8.48	-	-	-	-	-	-			
Mn & Cd	YDR322W	MRPL35	B	-	7.15	-	-	-	-	-	5.27	-	-	-	-	-	-			
Mn & Cd	YNR006W	VPS27	B	-	6.34	-	-	-	-	-	4.98	-	-	-	-	-	-			
Mn & Cd	YGL124C	MON1	B	-	7.36	-	-	-	-	-	4.18	-	-	-	-	-	-			
Mn & Cd	YGR089W	NNF2	B	-	3.78	-	-	-	-	-	3.95	-	-	-	-	-	-			
Mn	YEL050C	RML2	B	-	-	-	-	-	-	-	−6.95	-	-	-	-	-	-			
Mn	YCR024C	SLM5	B	-	-	-	-	-	-	-	−4.74	-	-	-	-	-	-			
Mn	YDR477W	SNF1	B	-	-	-	-	-	-	-	−4.39	-	-	-	-	-	-			
Cd	YGL095C	VPS45	B	-	10.49	-	-	-	-	-	-	-	-	-	-	-	-			
Cd	YKL041W	VPS24	B	-	8.61	-	-	-	-	-	-	-	-	-	-	-	-			
Cd	YBL024W	NCL1	B	-	8.61	-	-	-	-	-	-	-	-	-	-	-	-			
Cd	YOR036W	PEP12	B	-	7.80	-	-	-	-	-	-	-	-	-	-	-	-			
Cd	YML097C	VPS9	B	-	7.27	-	-	-	-	-	-	-	-	-	-	-	-			
***Significant ionomic changes in yeast with single copy gene knockouts in diploid background***																				
**Element being regulated**	**Line**	**Name**	**Group**	**Ca**	**Cd**	**Co**	**Cu**	**Fe**	**K**	**Mg**	**Mn**	**Mo**	**Na**	**Ni**	**P**	**S**	**Zn**			
Ca	YJL111W	CCT7	B	3.96	-	-	-	-	-	-	-	-	-	-	-	-	-			
Ca	YER125W	RSP5	B	4.05	-	-	-	-	-	-	-	-	-	-	-	-	-			
Na	YGL097W	SRM1	B	-	-	-	-	-	-	-	-	-	−7.22	-	-	-	-			
Na	YLR197W	NOP56	B	-	-	-	-	-	-	-	-	-	−3.67	-	-	-	-			
Mg	YIL033C	BCY1	B	-	-	-	-	-	-	−5.09	-	-	-	-	-	-	-			
Mg	YDL145C	COP1	A	-	-	-	-	-	-	4.41	-	-	-	-	-	-	-			
Mg	YDR188W	CCT6	A	-	-	-	-	-	-	5.20	-	-	-	-	-	-	-			
Mo	YKL059C	MPE1	B	-	-	-	-	-	-	-	-	4.11	-	-	-	-	-			
Mo	YGL150C	INO80	B	-	-	-	-	-	-	-	-	4.59	-	-	-	-	-			
Mo	YJR057W	CDC8	B	-	-	-	-	-	-	-	-	4.61	-	-	-	-	-			
Mo	YDL003W	MCD1	B	-	-	-	-	-	-	-	-	5.01	-	-	-	-	-			
Cd	YGL112C	TAF6	B	-	3.92	-	-	-	-	-	-	-	-	-	-	-	-			
Cd	YLR293C	GSP1	A	-	4.13	-	-	-	-	-	-	-	-	-	-	-	-			
Cd	YML093W	UTP14	B	-	4.35	-	-	-	-	-	-	-	-	-	-	-	-			
Mn	YNL112W	DBP2	B	-	-	-	-	-	-	-	4.49	-	-	-	-	-	-			
Mn	YNL062C	GCD10	B	-	-	-	-	-	-	-	7.29	-	-	-	-	-	-			
***Significant ionomic changes in yeast with single open reading frame over expressed***																				
**Element being regulated**	**Line**	**Name**	**Group**	**As**	**Ca**	**Cd**	**Cl**	**Co**	**Cu**	**Fe**	**K**	**Mg**	**Mn**	**Mo**	**Na**	**Ni**	**P**	**S**	**Se**	**Zn**
Na	YOR071C	NRT1	C	-	5.41	-	-	-	-	-	-	-	-	-	23.54	-	-	-	-	-
Na	YOR106W	VAM3	C	-	-	-	-	-	-	-	-	-	-	-	7.96	-	-	-	-	-
Na	YDR281C	PHM6	B	-	-	-	-	-	-	-	-	-	-	-	7.06	-	-	-	-	-
Ca	YER068W	MOT2	B	-	7.87	-	-	-	-	-	-	-	-	-	-	-	-	-	-	-
Ca	YGR027C	RPS25A	B	-	6.11	-	-	-	-	-	-	-	-	-	-	-	-	-	-	-
Ca	YGL163C	RAD54	B	-	5.86	-	-	-	-	-	-	-	-	-	-	-	-	-	-	-
Ca	YLR196W	PWP1	B	-	5.26	-	-	-	-	-	-	-	-	-	-	-	-	-	-	-
K	YPR138C	MEP3	A	-	-	-	-	-	-	-	−4.67	-	-	-	-	-	-	-	-	-
Mg & Ni	YPL031C	PHO85	B	-	-	-	-	-	-	-	-	5.85	-	4.93	-	4.52	-	-	-	-
Mo [Fe]	YLR013W	GAT3	C	-	-	-	-	-	-	-	-	-	-	15.8	-	-	-	-	-	-
Mo [Fe]	YDR222W		B	-	-	-	-	-	-	-	-	-	-	5.26	-	-	-	-	-	-
Mo [Fe]	YPL089C	RLM1	B	-	-	-	-	-	-	-	-	-	-	5.20	-	-	-	-	-	-
Mo [Fe]	YIL079C	AIR1	B	-	-	-	-	-	-	-	-	-	-	4.69	-	-	-	-	-	-
Mo [Fe]	YMR235C	RNA1	B	-	-	-	-	-	-	-	-	-	-	3.94	-	-	-	-	-	-
Cu	YDR441C	APT2	B	-	-	-	-	-	9.42	-	-	-	-	-	-	-	-	-	-	-
Cu	YIL118W	RHO3	B	-	-	-	-	-	4.80	-	-	-	-	-	-	-	-	-	-	-
Cu	YNL201C	PSY2	B	-	-	-	-	-	4.77	-	-	-	-	-	-	-	-	-	-	-
Cu	YPR009W	SUT2	B	-	-	-	-	-	4.76	-	-	-	-	-	-	-	-	-	-	-
Mn	YCL022C			-	-	-	-	-	-	-	-	-	−4.25	-	-	-	-	-	-	-
Cd	YJL031C	BET4	B	-	-	5.06	-	-	-	-	-	-	-	-	-	-	-	-	-	-
Cd	YDR452W	PPN1	B	-	-	4.46	-	-	-	-	-	-	-	-	-	-	-	-	-	-
Cd	YDL055C	PSA1	B	-	-	4.43	-	-	-	-	-	-	-	-	-	-	-	-	-	-

### GO term enrichment in group B and group C genes

In group C of the KO dataset the most over represented Biological Process (BP) GO term was found to be *cellular protein metabolic process* (e.g. ubiquitination, phosphorylation, folding, translation, acetylation) with the occurrence of *vesicular trafficking* and *ion-transport* ranking 2^nd^ and 3^rd^. This contrasts with group B of the KO dataset in which the BP terms *protein localization*, *vesicle-mediated transport* and *cellular ion homeostasis* predominate. This difference suggests that disruption of protein metabolism or trafficking has the highest likelihood of overcoming the resilience of the ionomic homeostasis networks, leading to large changes in the ionome. However, direct loss of function of ion-transport mechanisms can also have a significant impact on ionomic regulation as would be expected. The role of vesicular trafficking and the vacuole in regulation of the ionome is also supported by the observation for group B of enrichment of the Cellular Component (CC) terms *Golgi*, *endosome*, *vacuole* and *V-type ATPase*. In both group C and B enrichment in the CC term *mitochondrial matrix* also suggests an important role for mitochondria in regulation of the ionome. Similar conclusions for the importance of the vacuole and the mitochondria in regulating the yeast ionome were also made by Eide et al., [16]. Enrichment in the CC terms *ribonucleoprotein complex* in group B and *SAGA complex* in group C also support important roles for ribosomal function and transcription, respectively. Similar trends are also observed in the KOd dataset suggesting dosage of these essential genes in these processes e.g. *cellular protein metabolism* and *trafficking*, is also important in regulating the ionome. For a full description of all the GO term enrichments for the complete KO, KOd and OE data sets see Additional file 3: Figure S2 A-F.

### Gene clustering using ionomic profiles

In order to further uncover the functions of the genes and gene networks that are involved in controlling the yeast ionome we performed exhaustive significance clustering. This approach allowed us to take those genes that have a significant impact on the yeast ionome in the KO, KOd and OE sets and group them based on the directions of statistically significant changes in element abundance across all the elements (Additional file 4: Figure S3 A-C). For each strain and each element there can be a statistically significant increase, a statistically significant decrease, or no significant change in element abundance. The maximum possible number of patterns is therefore 3 to the power of the number of elements. We group the strains in clusters according to these patterns, and represent each cluster by its median profile of normalized element abundance. 508 out of 584 genes in the KO dataset were successfully grouped into 26 clusters with at least 3 strains. In KOd screen, 21 out of 35 strains were clustered into 5 groups. In OE screen, 406 out of 446 strains were clustered into 18 groups. The numbers of clusters are small as compared to the total number of possibilities, indicating that overall there is a limited number of relatively common ionomic phenotypes suggesting that the ionome only has a limited number of sets points that are consistent with cell viability.

**Figure 6:**
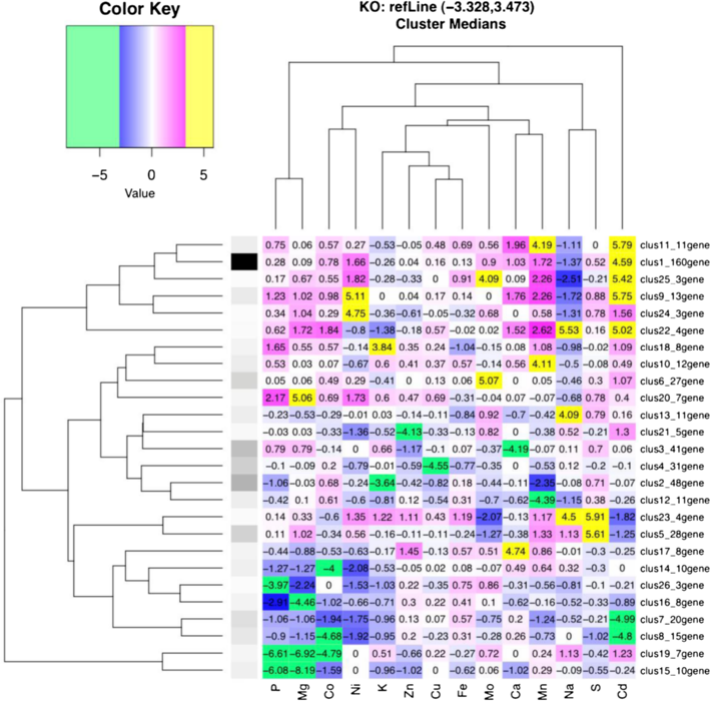
**Median elemental abundances (quantified as moderated Z-scores) of clusters generated using exhaustive significance clustering (ESC) on the ionome of yeast mutants with loss of function of genes that have a significant effect on at least one element in the ionome are visualized using a heat map.** The clusters including less than three genes are not shown. Clusters are represented in rows and elements are represented in columns. The dendrogram represents relationships between cluster (left) and elements (top) using hierarchical clustering. The grey-scale shading on the left of the heat map visually represents the number of genes in a cluster (darker represents more genes). Numbers on the heat map are moderated Z-scores for each element within each cluster. The highlight color of the numbers represents the magnitude of the abundance - green if the median elemental is less than −3.328, yellow if it is greater than 3.473, a gradient of blue if between −3.328 and 0, and a gradient of magenta if between 0 and 3.473. The yellow and green colors emphasize the elements that are positively (significance cut off = 3.473 moderated Z-scores) or negatively (significance cut of = −3.328 moderated Z-scores) changed in each of the clusters.

**Figure 7:**
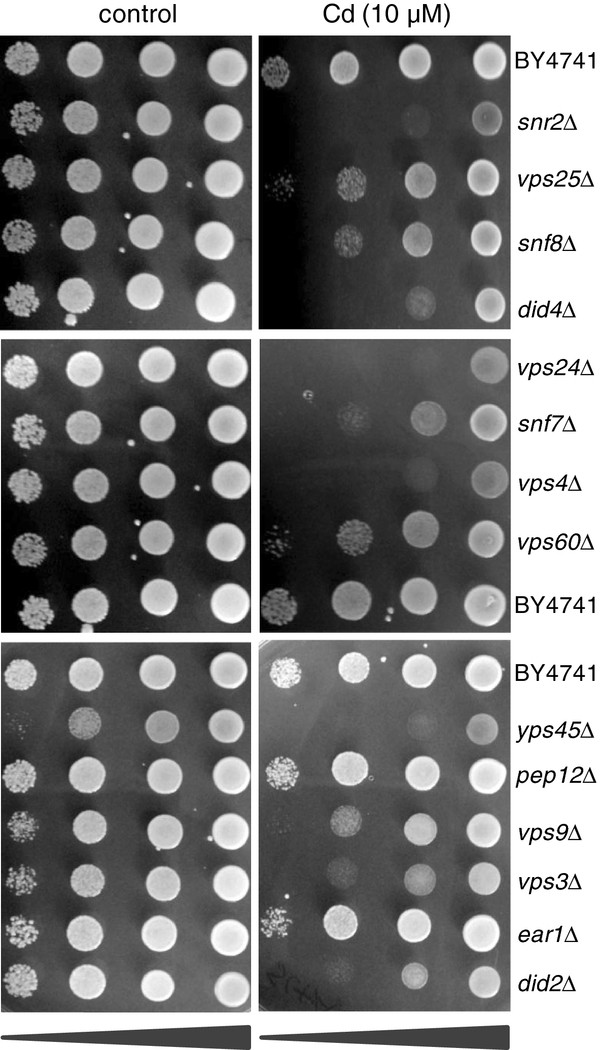
**Experimental validation of Cd accumulation in selected genes involved in the ESCRT and MVB pathways.** Yeast cells were grown overnight in liquid medium to an OD_600nm_ = 1.1. Cells were then serially 10-fold diluted, spotted onto solid media containing YNB without amino acids (6.7 g/L), CSM without uracil (0.77 g/L), uracil (0.086 g/L), NaCl (0.508 g/L) and 2% glucose and grown at 30°C for 72 hr.

To organize these clusters by similarity of their elemental profiles, and further identify functional patterns, their representative median elemental profiles were further subjected to hierarchical clustering for each of the KO, KOd and OE gene sets to organize the clusters based on similarity (Figure 6; Additional file 5: Figure S4 A & B). For the KO gene data set the largest cluster CLUSTER_1 contains 160 genes. Loss of function of genes in this cluster specifically cause elevated Cd. CLUSTER_1 is highly enriched in genes with the BP GO term *establishment of localization* (45 out of 160 genes) and *cellular protein catabolic process* (12 out of 160 genes). Our evidence supports the hypothesis that the Zn transporters Zrt1p, Zrt2p and Zrt3p play a role in Cd transport in yeast, with the plasma membrane localized transporters Zrt1p and Zrt2p transporting Cd into the cell and Zrt3p possibly transporting Cd out of the vacuole. Therefore, any process that either negatively affecting Zrt3p function, or positively affects Zrt1p and Zrt2p, through modified trafficking or turnover would be expected to cause an increased accumulation of Cd. This high Cd CLUSTER_1 contains multiple genes encoding proteins involved in the *endosomal sorting complex required for transport* (ESCRT) pathway which is involved in sorting ubiquitinated transmembrane proteins to the vacuole for degradation, via the formation of multivesicular bodies (MVB). CLUSTER_1 also contains several genes involved in the formation of MVB. To validate the high Cd accumulation in strains carrying a loss of function mutation in genes encoding proteins part of the ESCRT or involved in the formation of MVB in CLUSTER_1 we tested for Cd sensitivity in 14 strains with loss of function of genes (*SRN2, VPS25, SNF8, DID4, VPS24, SNF7, VPS4, VPS60, VPS45, PEP12, VPS9, VPS3, EAR1, DID2*) in these pathways, and that we had shown to have elevated Cd abundance. Of the 14 strains tested 12 displayed increased sensitivity to Cd compared to the control (BY4741), and strains with loss of function of *PEP12* and *EAR1* showed no change from the control (BY4741) (Figure 7). Other clusters showing similarity to CLUSTER_1 are CLUSTER_11 with high Cd and Mn, CLUSTER_25 with high Cd and Mo, CLUSTER_9 with high Cd and Ni and CLUSTER_22 with high Cd and Na (Figure 6).

CLUSTER_15 & 19 group together based on their median ionomic profiles (Figure 6) which are both low in P, Mg and Co but with CLUSTER_19 showing a significant reduction in Co while CLUSTER_15 shows only a trend to lower Co. Together these two clusters contain multiple genes encoding the V-type ATPase (*VMA1-5*, *VMA7*, *VMA9*, *VMA11*) and associated genes required for its function (*VPH2*, *VMA22*), along with genes encoding members of the vacuolar transport complex (*VTC4* and *VTC1*). Further, *vma6Δ*, *8Δ*, *10Δ*, *13Δ*, *16Δ* and *21Δ* also all show significant reductions in Co, Mg and P but also have other changes such as elevated Mo and Na and reduced Mn, though none of these six genes falls into a cluster. In fact, all the genes known to be involved in encoding proteins for the V-type ATPase are represented in the KO set of significant genes (*VMA1-13*, *16*, *21*) reinforcing the critical function the V-type ATPase plays in regulating the yeast ionome. Eide et al., [16] also identified half of these genes encoding V-Type ATPase subunits in their screen of the yeast ionome using a similar knockout collection. However, most likely due to the initial n = 1 sampling 50% of these genes were not identified, suggesting a 50% false negative rate in this screen [16] compared to the data reported here based on n = 4 replicates per strain. This is also consistent with the lower number of genes reported by Eide et al., [16] to affect the yeast ionome compared to this study (212 compared to 584). Furthermore, loss of function of the genes encoding the V-type ATPase subunits that Eide et al., [16] did identify caused reductions in Co, P and Mg as we report here. Screening of a similar set of yeast deletion strains for P accumulation [15], and Cs and Sr accumulation [14] also identified loss of function of genes encoding V-type ATPase subunits, regulators and assembly factors as causing significant reductions in these elements, confirming the central role the vacuole plays in regulation of the yeast ionome.

CLUSTER_2 has low K and Mn and the genes in this cluster are enriched in the Cellular Component (CC) GO term, ‘*mitochondrion*’ (34 out of 48 genes). Also, the related group CLUSTER_12 (Figure 6) showing low Mn is also highly enriched in the CC GO term ‘*mitochondrion*’ (8 out of 11 genes). Treatment with ethidium bromide is known to specifically destroy mitochondrial DNA and block mitochondrial function [40]. We observe that ethidium bromide treatment causes a significant (p < 0.01) reduction in K (39%) and Mn (11%) (data not shown) partially mimicking the ionomic phenotypes of the CLUSTER_2 and 12, supporting the association of this ionomic phenotype with a disruption in mitochondrial function. Eide et al., [16] also reported that genes involved in mitochondrial function have a significant impact on the yeast ionome.

If GO term enrichment is observed for the clusters for the KO, KOd and OE datasets they can be explored in full in Additional file 3: Figure S2A-F.

### Ionomic interaction gene networks

To explore the interrelationships between the genes involved in regulating the ionome we constructed networks of genes based on their pair-wise physical and genetic interactions. Gene sets used to build these networks were either genes for which loss of function caused a significant change in a particular element, or genes from particular ionomic clusters.

**Figure 8:**
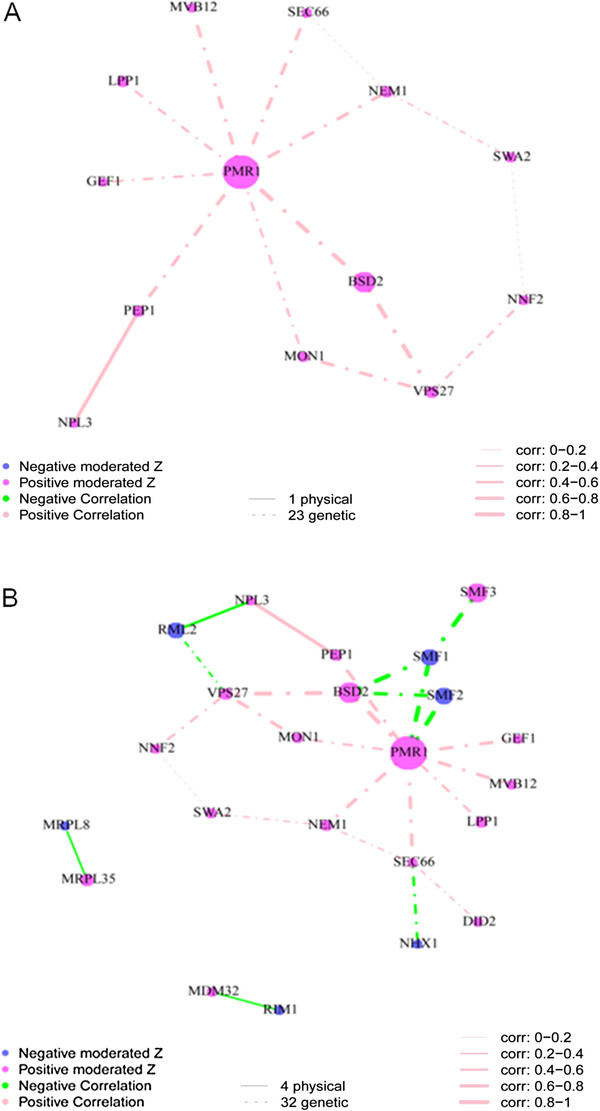
**Visualization of the interaction network of selected genes for which loss of function impacts the ionome.** (**A**) The 22 genes from CLUSTER_10 and CLUSTER_11 that increase the abundance of Mn (see Figure 6) were selected and an interaction network built based on known protein protein and genetic interactions. There is 1 physical interaction and 23 genetic interactions among 13 of the selected 22 genes. (**B**) The 51 genes that significantly affect the abundance (increase and decrease) of Mn were selected and an interaction network built based on known protein protein and genetic interactions. There are 4 physical interactions and 32 genetic interactions among 23 of the 51 selected genes. Protein protein and genetic interaction information were obtained from BioGRID (48). Nodes represent genes, node color represents the direction of changes in elemental abundance (magenta increase in abundance, blue decrease in abundance), and node size represents the magnitude of the change in the ionome based on moderated Z-score. Lines joining the nodes (edges) in the graph represent the interactions. The color of the edges represents the direction of the correction (pink is positive and green negative) between the interacting pair of genes based on the ionomic profiles of the loss of function mutants. The width of the edges represents the strength of the correlation. A dotted edge represents a genetic interaction and solid edge represents a physical interaction.

The interaction network built from genes that cause elevated Mn (genes that group in CLUSTER_10 & 11) identifies *PMR1* as being at the centre of a network interacting with *BSD2* and genes involved in protein import and sorting (Figure 8A). Of the 23 genes in the two clusters in this network 13 of them interact genetically or through protein interactions. Of these 13 genes eight interact directly with *PMR1*, three are one interaction away and one gene is two interactions away from *PMR1*. The correlation of the ionomic profile for all genes directly interacting with *PMR1* is > 0.5 except for *YDR503C* (*LPP1*), the highest is *BSD2* at 0.839. The high Mn clusters are therefore very likely driven primarily by perturbations of Pmr1, a protein known to drive Golgi/ER sequestration and exocytosis of Mn. This network therefore highlights the central role Pmr1 plays in controlling cellular Mn levels and identifies numerous genes that control the function of Pmr1. When all genes are included in the network that cause both increases or decreases in Mn (Figure 8B) genes encoding the Mn transporters Smf1 and Smf2 appear in the network interacting with *PMR1*, and also bring in *SMF3* via a genetic interaction with *SMF1*. In this network 10 genes interact directly with *PMR1*, the eight genes observed in Figure 8A and *SMF1* and *SMF2*. The correlation of the ionomic profile for all 10 genes that interact with *PMR1* in this larger Mn network is > 0.5 except for *SMF1* and *SMF2* which are strongly negatively correlated (−0.9). This network also supports the placement of *SMF3* in the Mn homeostasis network. From this network a picture of Mn homeostasis emerges. Smf1 and Smf2 both play non-redundant roles in Mn uptake by the cell. Pmr1 plays a central role in exporting Mn from the cell via Golgi/ER sequestration and exocytosis, and Smf3 is required for unloading of Mn from the vacuole. Uptake and export of Mn by Smf1/Smf2 and Pmr1 is integrated by Bsd2. Further, the Mn export activity of Pmr1 requires correct protein import and vesicular trafficking (e.g. *PEP1*, *VPS27*, *MON1*, *SWA2*, *NHX1*, *SEC66*, *DID2* and *MVB12*). In this extended network we also now see evidence of genes involved in mitochondrial function (e.g. *MRPL8, MRPL35, MDM32, RML2* and *RIM1*) supporting the enrichment in genes with mitochondrial function in the low Mn CLUSTER_12 and CLUSTER_2 clusters.

**Figure 9:**
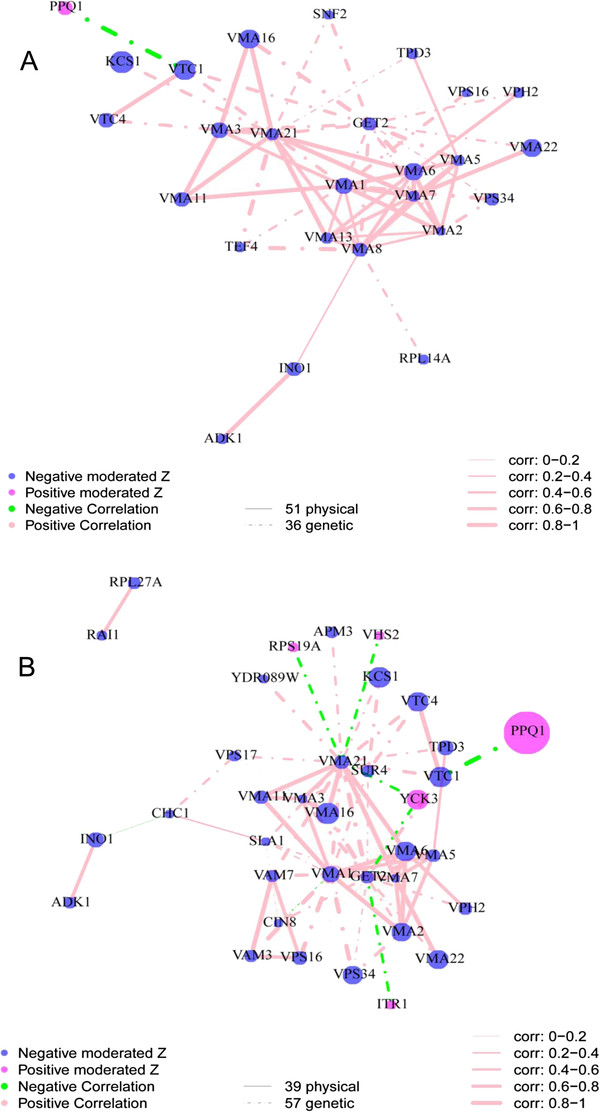
**Visualization of the interaction network of selected genes for which loss of function impacts the ionome.** (**A**) The 33 genes that affect the abundance (increase and decrease) of P were selected and an interaction network built based on known protein protein and genetic interactions. There are 51 physical interactions and 36 genetic interactions among 26 of the 33 selected genes. (**B**) The 55 genes that affect the abundance (increase and decrease) of Mg were selected and an interaction network built based on known protein protein and genetic interactions. There are 39 physical interactions and 57 genetic interactions among 36 of the 55 selected genes. Protein protein and genetic interaction information were obtained from BioGRID [48]. Nodes represent genes, node color represents the direction of changes in elemental abundance (magenta increase in abundance, blue decrease in abundance), and node size represents the magnitude of the change in the ionome based on moderated Z-score. Lines joining the nodes (edges) in the graph represent the interactions. The color of the edges represents the direction of the correction (pink is positive and green negative) between the interacting pair of genes based on the ionomic profiles of the loss of function mutants. The width of the edges represents the strength of the correlation. A dotted edge represents a genetic interaction and solid edge represents a physical interaction.

The network generated from the 33 genes that affect P in the KO dataset contains 26 genes that interact genetically or via protein interactions (Figure 9A). This network contains 12 genes that directly encode subunits of the V-type ATPase or are involved indirectly in its functions (*VMA1 – 3*, *5 – 8, 11, 13, 16, 21, 22*). It also clearly identifies a novel extended ionomic network of genes involved in P homeostasis. This network includes genes involved in polyphosphate accumulation and vacuolar transport chaperone (*VTC1* &*4*), protein trafficking and inositol-3-phosphate biosynthesis (Figure 9A). The networks generated for all genes that alter either P or Mg have similar topographies (Figure 9A & B) since 21 of the 26 genes in the P regulatory network also affect the Mg network, suggesting a significant overlap in the regulatory networks for P and Mg, and this supports the correlation of these two elements in CLUSTER_15 and 19 (Figure 6).

**Figure 10:**
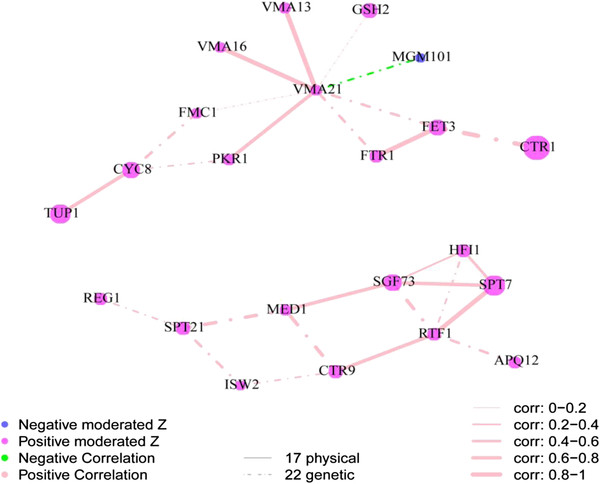
**Visualization of the interaction network of selected genes for which loss of function impacts the ionome.** The 37 genes that affect the abundance of Mo (increase and decrease) were selected and an interaction network built based on known protein protein and genetic interactions. There are 17 physical interactions and 22 genetic interactions among 22 of the 37 selected genes. Protein-protein and genetic interaction information were obtained from BioGRID [48]. Nodes represent genes, node color represents the direction of changes in elemental abundance (magenta increase in abundance, blue decrease in abundance), and node size represents the magnitude of the change in the ionome based on moderated Z-score. Lines joining the nodes (edges) in the graph represent the interactions. The color of the edges represents the direction of the correction (pink is positive and green negative) between the interacting pair of genes based on the ionomic profiles of the loss of function mutants. The width of the edges represents the strength of the correlation. A dotted edge represents a genetic interaction and solid edge represents a physical interaction.

The interaction network of genes that affect Mo accumulation (Figure 10) contains a set of 22 genes divided into two clear sub networks. The sub network with *VMA21* appears to link vacuolar (e.g. *VMA 13, 16* and *21*) with mitochondrial function (e.g. *FMC1*, and *MGM10*) supporting cross-talk between these two important organelles. Further, this sub network also appears to link Fe- and Cu-homeostasis (*FET3*, *FTR1* and *CTR1*) with vacuolar and mitochondrial function. The second sub network contains genes primarily involved in transcription. Mo is thought to have no biochemical function in *S. cerevisiae*[24], and we hypothesize that loss of function of genes that affect Mo accumulation in yeast may be acting indirectly through modification of the pH of the media by affecting the respiratory quotient (fermentation/respiration). Increased fermentation leads to increased media acidification [41], and this can be caused by loss of mitochondrial viability. Within the Mo interaction network we observe *MGM101* (required for mitochondrial genome maintenance), and *FMC1* (loss of function of which causes no proton gradient blocking import of proteins into the mitochondria leading to a failure of the mitochondria to develop correctly), supporting this hypothesis. In fact, the *MGM101* null mutant is known to display the petite phenotype [42] due to loss of mitochondrial DNA. Loss of mitochondrial DNA can also be caused by ethidium bromide treatment which phenocopies the petite phenotype of *MGM101*, and importantly were observe (data not shown) that treatment with 100 mg/L ethidium bromide also causes a significant (p < 0.01) increase in Mo from 0.0025 mg/L/OD_600nm_ under control conditions to 0.0048 mg/L/OD_600nm_ (n = 18 replicate cultures per treatment). This further reinforces the possible connection between mitochondrial function and Mo accumulation. Fe-deficiency is also known to reduced those biochemical processes that require Fe, for review see [3], including respiration. The occurrence of key genes involved in Fe-homeostasis (*FTR1*, *FET3*, *CTR1*) in the Mo network supports this. Further, *GSH2* also occurs in the Mo network and this might be explained by the fact that a loss of GSH biosynthesis will impair the biogenesis of Fe-S cluster containing proteins required for mitochondrial function, for review see [43], leading to impairment of respiration. The Mo interaction network also contains key genes required for the correct assembly and function of V-type ATPase (*VMA21, VMA16, VMA13 and PKR1*). It is possible that disruption of these genes interferes with vacuole – mitochondria cross-talk [44,45] leading to impaired mitochondrial function. Loss of function strains of these four genes show reduced Mg/P/Co but also high Mo, unlike other V-type ATPase genes in the CLUSTER_15 & 19. In fact, the high Mo and reduced K and Mn of these mutants show similarities to the genes found in the mitochondria CLUSTER_2, further supporting a connection between the vacuole and the mitochondria.

**Figure 11:**
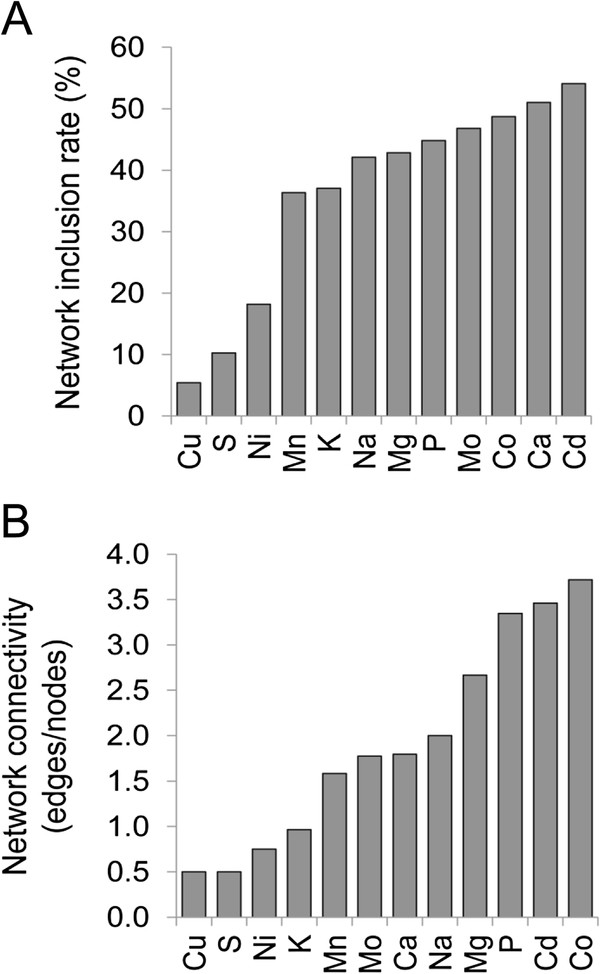
**Summary of ionomic gene interaction networks.** (**A**) Rate of inclusion in a particular ionomic interaction network is presented for each network of interacting genes built from genes identified as causing a change in a specific element in loss of function mutants. Network inclusion rate is calculated as the total number of genes tested for network interaction for a given element divided by the number of genes for that element identified to interact (protein protein or genetic interactions) multiplied by 100 and is presented for each element interacting network. (**B**) The connectivity in a particular ionomic interaction network is presented for each network of interacting genes built from genes identified as causing a change in a specific element in the loss of function mutant. Network connectivity is calculated as the number of edges divided by the number of nodes in a given network and is presented for each element interaction network.

Inclusion within these interacting gene networks provides further validation that those genes identified to have a significant effect on the yeast ionome are part of true functional units. Network analysis of all significant genes for each of the 14 elements quantified in the KO dataset (Additional file 6: Figure S5) revealed networks with at least 2 nodes for all elements, except Fe and Zn. Within these networks two groups were suggested based on differences in the number of nodes in the network as a percentage of the significant genes tested for network connectivity (network inclusion rate) (Figure 11A). In the first group composed of networks for Mn, K, Na, Mg, P, Mo, Co, Ca and Cd the network inclusion rate ranged from 36 – 54%, providing support for a functional role in regulation of the ionome in yeast for these genes. In the second group composed of Cu, S and Ni, the network inclusion rate fell to 5 – 18% suggesting that the genes identified to have a significant effect on these elements may be less likely to be a part of functional systems. The complete lack of network support for genes identified to significant affects on Fe and Zn suggests that these may not be part of systems that regulate these elements in yeast. Evaluation of the connectivity within each network using edges/node reveals large differences between the networks (Figure 11B). Co, Cd, P and Mg have the highest network connectivity and this can be explained by the fact that they contain single large networks of interconnected genes. The Cd network is highly enriched in genes from CLUSTER_1 involved in protein localization including genes in the ESCRT pathway involving sorting of ubiquitinated transmembrane proteins to the vacuole for degradation. The P, Mg and Co networks contain multiple genes from CLUSTER_15 and 19 which are highly enriched in genes involved in vacuolar function, including the activity of the V-Type ATPase. The Mn and Mo networks have intermediate levels of network connectivity perhaps because these networks appear to have 2 independent sub networks (Figures 8B &10). For Mo one sub network contains genes involved in transcription and the other is an interconnected set of genes with functions in the mitochondria, vacuole and Fe homeostasis. For Mn one sub network contain genes involved in mitochondrial function, and the other genes involved in trafficking and activity of Mn transporters.

These results further validate the methodologies developed to screen the ionome of yeast in high throughput using 96 well plates as well as the statistical methods developed to deal with the highly sensitive data. Overall, this approach appears to approximately double the ability to detect ionomic differences compared to previous studies providing a much high resolution of the genes and gene networks involved in regulating the yeast ionome. The results presented here are however likely a small subsample of the genes affecting the ionome of a single celled Eukaryote. The lack of several known ionome altering genes in our datasets suggests that rescreening of these populations in different environments would yield another set of known and novel genes. Such full genome screens could be accomplished in approximately 6 months per condition and could greatly increase our understanding of the gene by environment interactions controlling elemental accumulation.

### Hypothesis testing using the online yeast ionomics database

A yeast ionomics database has been developed and is accessible through the ionomicsHUB (iHUB) (http://www.ionomicshub.org). From the homepage follow the link to the *Yeast Database*. The database contains ionomic data on the 4940 yeast strains in the KO set, 1127 in the KOd set and 5798 in the OE set. The data consists of the raw solution concentration data for each element measured in each strain, and the same data normalized to the optical density (A_600_) of the yeast culture just prior to preparation for ICP-MS analysis. Further, the database also contains the ionomic data from the KO, KOd and OE sets normalized separately with common strains analyzed in each 96-well plate using a mixed model approach, as described previously by Yu et al., [18]. This normalization allows removal of plate effects and facilitates comparisons of different stains across the complete KO, KOd and OE datasets. This database can be used to rapidly retrieve, display and download ionomics data (both raw, OD normalized and population normalized) to test if the loss or gain of function of any gene of interest in the full yeast genome has an effect on the yeast ionome.

**Figure 12:**
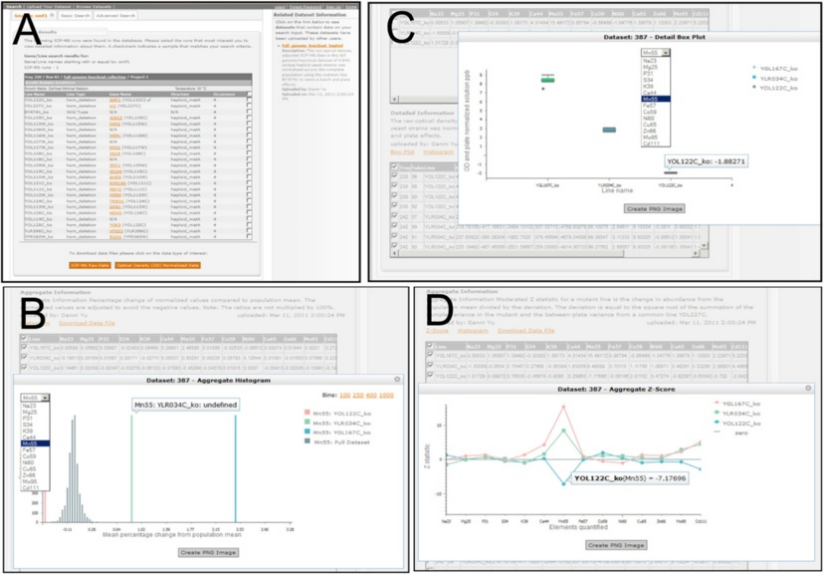
**Searching and displaying yeast ionomics data at the ionomicsHUB (iHUB).** Screen shots of example search results (**A**), and available histogram (**B**), box plot (**C**) and Z-score (**D**) plotting tools in the database. The iHUB can be accessed at http://www.ionomicshub.org and following the link to the *Yeast Database*.

Ionomic data for any of the viable yeast strains in the KO, KOd and OE collections can be retrieved from the database using the *Basic Search* feature by simply typing in the name of the mutant or gene of interest. Multiple genes can also be searched simultaneously by separating the name of each mutant/gene using a comma. The *Basic Search* returns the 96-well plate(s) (*tray*) in which the mutant of interest was analyzed. An individual strain, multiple strains or the complete 96-well plate can be selected using the check boxes (Figure 12A) and data for solution concentrations (*ICP-MS Raw Data*) or OD normalized (*Optical Density (OD) Normalized Data*) downloaded as a comma-separated values (CSV) file. The *Advance Search* also allows retrieval of data based on a specific 96-well plate or ICP-MS run. A search on a mutant/gene also returns links to other normalized datasets that contain data on the mutant/gene being search e.g. *Full genome knockout haploid* (Figure 12A). Following the link for a particular dataset presents tools for viewing, plotting and downloading the normalized data on the mutant/gene of interest from that particular dataset. A description of the data set and each type of data is presented. For example, in the *Full genome knockout haploid* dataset, which has been normalized following Yu et al., [18], data can be viewed for percentage change of normalized values compared to the population mean, change in concentration from the population mean divided by the deviation (*Moderated Z statistic*), and the optical density adjusted concentration data after normalization across population. The line(s) of interest can be selected using the check boxes and data plotted or downloaded (as CSV file). Plots available are *Histogram* (Figure 12B), *Box Plots* (Figure 12C), and *Z-Score* (Figure 12D) depending on the type of data. In Figure 12 B-D data is displayed in various ways illustrating the fact that loss of function of *SMF1* (*YOL122C*) causes reduced Mn accumulation whereas loss of function of *SMF3* (*YLR034C*) and *PMR1* (*YGL167C*) causes elevated Mn.

Finally, complete normalized datasets for all ~12,000 strains tested can be accessed via the *Browse Datasets* tab and downloaded as zipped CSV files.

## Conclusions

Here, we report a genome-wide scan of approximately 6000 genes in yeast using ICP-MS to quantify 14 to 17 different elements (As, Ca, Cd, Cl, Co, Cu, Fe, K, Mg, Mn, Mo, Na, Ni, P, S, Se, and Zn) in both loss of function and gain of function mutant yeast strains. Of the 6067 knockout strains analyzed 619 showed a significant ionomic phenotype. Disruption of protein metabolism or trafficking has the highest likelihood of overcoming the resilience of the ionomic homeostasis networks, leading to the largest changes in the ionome. Of the genes significantly impacting the ionome 35 genes were lethal when knocked, but showed an ionomic phenotype when present as a single copy in diploid strains, suggesting that gene dosage can be important in ionomic regulation. Of the 5798 yeast ORFs over expressed 446 impacted the ionome, and as might be expected over expression tends to produce more extreme ionomic phenotypes than loss of function. Interestingly, no significant overlap between the gene sets identified from the loss and gain of function experiments was found, suggesting that on a genome-wide scale loss of function and over expression do not produce related phenotypes. Analysis of our dataset revealed numerous novel genes which significantly impact the ionome when knocked out or over expressed, and these genes could form the basis of many follow up experiments. Clustering based on the ionomic phenotype of over expression or knockout strains identified a limited number of clusters (18 – 26) with three or more members, and analysis of gene functions enriched within these clusters revealed that perturbation of particular cellular functions creates unique ionomic phenotypes, such as the mitochondria, vacuolar and ESCRT pathway, providing a robust way to predict the potential function of genes. Analysis of the protein-protein and genetic interaction of genes identified to impact the yeast ionome also revealed novel functional gene networks that illustrate the physical and genetic interactions between genes within a particular mineral nutrient regulatory network. The high network inclusion rate (approx 45%) for genes impacting most elements strongly suggests that these genes are truly part of functional systems involved in ionomic regulation. Such networks help identify important hub genes that play a critical role in network function, such as *PMR1* in Mn homeostasis; novel members of mineral nutrient homeostasis networks, such as the identification of *SMF1* as a major pathway for Mn uptake non-redundant with *SMF2*, and *SMF3* as the unknown gene involved in Mn retrieval from the vacuole; and cross-talk between cellular functions such as the mitochondria and the vacuole. A better understanding of such regulatory networks is also critical if we are to be able to functionally model ionomic regulation within yeast and other organisms. To facilitate such understanding we have developed a publically accessible database (http://www.ionomicshub.org) through which all the yeast ionomic data described here can be searched, downloaded and analyzed for hypothesis testing and experimental follow up.

## Methods

### Yeast strains analyzed

Three different yeast collections were used in the present study: the deletion (knockout) strains—the *MAT*a haploid (KO) and heterozygous diploid (KOd) [5] - and ORF overexpression strains (*aka* OE) [7]. All strains were obtained in 96-well plates from Open Biosystems (Huntsville, AL, USA) and were maintained at −80°C. The deletion strains were in YPD media and glycerol (15%) while the ORF collection came in SD-URA medium supplemented with 15% glycerol.

### Culture conditions

All yeast strains were cultivated in 96-well square deep-well plates using synthetic defined minimal media with supplementation as previously described [17]. Original cultures for each strain (KO and KOd) was amplified by inoculating 5 μL of culture into 500 μL of SD-Ura plus uracil growth medium per plate well and cultured for 48 hr at 30°C and 400 rpm. Final cultures for ICP-MS analysis were prepared by inoculating 750 μl of SD-Ura plus uracil growth medium supplemented with cobalt (0.03 μM), cadmium (0.18 μM), molybdenum (0.52 μM), nickel (1.70 μM) and sodium (8.70 μM) with 20 μL of the amplified culture per plate well. The ORF overexpression collection (OE) was cultivated in two stages after amplification as above. In the first stage the strains were grown in SD-Ura with 2% raffinose with supplementation with the same elements as above plus arsenate (6.67 μM) and selenate (63.30 μM) by the addition of 10 μL of SD-Ura amplified inoculum to 500 μL of growth medi per plate well and cultured at 30°C and 400 rpm for 48 h. This was followed by a protein induction stage where 3X SD-Ura plus 6% galactose was added (250 μL per well) and cultivation at 30°C and 400 rpm for a further 12 h. Yeast cells were harvested for analysis at the post-diauxic growth phase.

### Sample preparation and ICP-MS analysis

Until the final step, all sample processing for ICP-MS analysis was performed in AcroPrep™ 96 PVDF filter membrane multi-well plates (0.45 μm, 350 μL). The PVDF (polyvinylidene fluoride) membrane is hydrophobic but can be transformed into the hydrophilic state by wetting with methanol to enable yeast cells wash and rinse. The reverse transformation is accomplished by drying the membrane. The cultivated yeast cultures were transferred from the growth plates into wetted filter plates using multichannel pipettes (200 or 400 μL per well; wells could hold more than stated capacity). Concurrently, the same amounts (200 μL well^-1^) were pipetted into Clear View micro-titer plates and the optical densities measured using a plate reader. The yeast cells in the filter plates were washed and rinsed (4X each) in situ with EDTA (1 μM, pH ~ 8) and deionized water, respectively, using a vacuum manifold. Next, the plates containing the cells on the membrane were dried (88°C for 150 min) to restore membrane hydrophobicity. The yeast cells were acid digested directly inside the filter plates (100 μL well^-1^ nitric acid at 88°C for ~40 min) using a multi-block heater. The digested yeast solutions were drawn through the filter and into 96 deep-well collection plates and diluted to a final volume of 1.6 mL per well (including gallium internal standard and Triton X-100 surfactant to reduce surface tension for smoother self aspiration of the ICP-MS micro-nebulizer). The processed yeast samples were analyzed using an Elan DRC II ICP-MS (PerkinElmer) equipped with ESI SC-2 autosampler unit that could accommodate 96 deep-well plates and an Apex Q (Elemental Scientific Inc.) sample introduction system. External calibration method (Elan instrument software version 3.4) was used together with calibration and internal standards to obtain the concentration data for the 14 or 17 monitored elements.

### Data normalization and significance testing

The calculation of the normalized ion abundance (that we call moderated Z statistic) used as input to this study was previously described by [18]. Briefly, 96-well plates included four control strains and up to 20 mutant strains, and three plates were analyzed with an ICP-MS instrument in a day. Fourteen or 17 elements were quantified in concentration unit of ppm (parts per million) in each sample. To remove spurious technical variation, the values were normalized to control strains with linear mixed-effect models. Next, moderated Z statistic for each element and each mutant strain was calculated as standardized difference between the normalized phenotype of a gene and the median across genes as shown in (Eq. 1). The significance cutoff was determined to control the False Discovery Rate at the level of 0.05. An additional manual filtering was performed for quality control. Specifically, the list of all the statistically significant changes was further reduced to the strains and elements where at least 75% of biological replicates deviated from the median in the same direction, by at least 2 times of median absolute deviation of all samples across genes. The final output of this step is the list of strains with statistically significant changes in element abundance that satisfy this criterion.

(1) $$Z_{\mathrm{ge}}=\frac{D_{\mathrm{ge}}-median\left(D_{\mathrm{ge}}\right)}{\underset{g}{median}\left(\left|D_{\mathrm{ge}}-median\left(D_{\mathrm{ge}}\right)\right|\right)\cdot 1.4826}$$

where *D*_*ge*_ is the phenotype for gene *g* and element *e* normalized according to the mixed model as described in [18], and 1.4826 rescaled the denominator to be a robust unbiased estimation of the standard deviation [46].

Genes with same statistically significant phenotypes in all elements were clustered using exhaustive significance clustering, as follows. For each strain and each element there can be a statistically significant increase, a statistically significant decrease, or no significant change in the abundance. For example, when 14 elements were quantified, there can possibly be 3^14^ = 4782969 clusters in total. The exhaustive significance clustering groups the strains according to the 3^14^ significance patterns.

Each cluster was represented by its median profile of moderated statistics Z_*ge*_. Using the function heatmap.2 in R package gplots 2.10.1, we drew the heatmap for the median profiles in clusters. In the heatmap, a row represents the median profiles of a cluster and a column corresponds to an element. Using the function hclust in R package stats 2.15.0, the dendrograms of rows or columns were added on the margin of the heatmap. The hierarchical clustering with “method=’complete’” was utilized for the dendrograms based on the Euclidean distances among clusters in rows or among elements in columns.

In addition to the statistical significance we considered practical significance of changes in element abundance. The practical significance was represented by the percentage change in the observed phenotype *perCh*_*ge*_ defined as in (Eq. 2) (i.e. the difference between the ion expression for a mutant and the average ion expression across mutants in the unit of the average ion expression across mutants). The 584 (KO), 35 (KOd), and 446 (OE) candidate genes were binned into groups A, B and C according to the *perCh*_*ge*_ cutoff at −20 to 20% for group A, 20 to 100% or −20 to −50% for group B, greater than 100% or less than −50% for group C.

(2) $$perCh_{\mathrm{ge}}=\frac{{\tilde{\sigma }}_{\mathrm{ge}}D_{\mathrm{ge}}-mean\left({\tilde{\sigma }}_{\mathrm{ge}}D_{\mathrm{ge}}\right)}{\underset{g}{mean}\left({\tilde{\sigma }}_{\mathrm{ge}}D_{\mathrm{ge}}\right)}$$

where σ˜_*ge*_ estimates the standard deviation for gene *g* and element *e* based on normalized ion abundance [18].

We investigated whether particular functions were enriched for a group of genes. Standardized vocabulary of terms annotating gene functions and products consist of the Gene Ontology (GO) database. Those GO terms are constructed in trees under three categories, Biological Process (BP), Molecular Function (MF), and Cellular Component (CC). We performed GO enrichment analysis to statistically identify the over-represented GO terms and those particular functional genes in the group. The R package GOstats 2.18.0 [47] was utilized. For a GO term, the number of matched genes in a selecting group was counted. The p-value of conditional hypergeometric test is the statistical method that quantifies the chance of having more extreme value than the observed number. In the relationship of children and parents in the GO database, many significant GO terms were redundant since their significance may be caused by the significance of their children. To account for such redundancy, the genes matched by a significant GO term would not be counted when performing the statistical test for the parents of the GO term. This test is known as the conditional hypergeometric testing.

The protein-protein interactions and the genetic interactions in yeast were extracted from BioGRID [48] a database that contains curated data from published experiments. We selected sub-networks which represent the physical and the genetic interactions of the genes in each cluster. The sub-networks were visualized using the R package igraph 0.5.5-3 [49]. In this visualization genes are nodes and interaction between the genes are edges in the network. If there is an interaction between genes in a cluster, then both genes appeared as nodes in the sub-networks and the ineraction appears as the nodes. The genes in a cluster that had no edge to the others were not shown in the graph. The nodes were colored in magenta to represent positive changes and blue for negative changes. Solid lines represent physical interactions, and dashed lines represent genetic interactions.

## Competing interests

The authors declare that they have no competing interests.

## Authors’ contributions

DY and OV performed the statistical analyses and helped draft the manuscript. IB helped perform the statistical analyses and data interpretation, design of the experiments and database, and drafting of the manuscript. SK and OKV designed and performed the Cd tolerance testing on selected yeast strains. JMCD developed the ICP-MS analytical methodologies, performed all ICP-MS analyses and input the data into the database. MO designed the database and managed its development and implementation. DES designed the experimental approach, helped with the statistical analyses and database design, performed the data interpretation and wrote the manuscript. All authors read and approved the manuscript.

## Supplementary Material

Additional file 1: Table S1 Moderated Z-scores for all significant genes in the KO dataset that pass the annealing process. Elements highlighted in yellow with red text passed the significance difference cut of (−3.328, 3.473). **Table S2** Moderated Z-scores for all significant genes in the KOd dataset that pass the annealing process. Elements highlighted in yellow with red text passed the significance difference cut of (−3.527, 3.572). **Table S3** Moderated Z-scores for all significant genes in the OE dataset that pass the annealing process. Elements highlighted in yellow with red text passed the significance difference cut of (−3.801, 3.735). **Table S4** The overlap of significant genes in the KO, KOd and OE data sets. **Table S5** Genes known to be involved in regulating the ionome from the literature. Click here for file

Additional file 2: Figure S1 Growth of yeast wild-type and *zrt3* deletion mutant in the presence of Cd in the growth media. Yeast were grown in YNB at 30°C with shaking (200 rpm) in the presence of various concentrations of Cd^2+^ and optical density measured after 48hr. Data represents means (n = 9) ± standard deviations. Click here for file

Additional file 3: Figure S2 Directed Acyclic Graph (DAG) and pie charts for Gene Ontology (GO) data for KO (**A** &**B**), KOd (**C** &**D**) and OE (**D** &**F**) gene datasets. The R packages GOstats, Rgraphviz and graphics were utilized to perform GO enrichment, generate the DAG plots and the pie plots. Click here for file

Additional file 4: Figure S3 Clusters of ionomic profiles using the exhaustive significance clustering (ESC) method for the KO (**A**), KOd (**B**) and OE (**C**) data sets of genes that have a significant impact on the ionome. The X-axis represents the elements used in the clustering and the Y-axis represents the moderated Z values used for each element. Only the genes that significantly affect at least one element and pass the annealing process are included, and only the clusters that include at least 3 genes are shown. Click here for file

Additional file 5: Figure S4 Median elemental abundances (quantified as moderated Z-scores) of clusters generated using exhaustive significance clustering (ESC) on the ionome of yeast mutants from the KOd (**A**) or OE (**B**) data sets are visualized using a heat map. The clusters including less than three genes are not shown. Clusters are represented in rows and elements are represented in columns. The dendrogram represents relationships between cluster (left) and elements (top) using hierarchical clustering. The grey-scale shading on the left of the heat map visually represents the number of genes in a cluster (darker represents more genes). Numbers on the heat map are moderated Z-scores for each element within each cluster. The highlight color of the numbers represents the magnitude of the abundance - green if the median elemental is less than −3.527 (**A**) or −3.801 (**B**) , yellow if it is greater than 3.572 (**A**) or 3.735 (**B**), a gradient of blue if between the lower significance cut of and 0, and a gradient of magenta if between 0 and the upper significance cut off. The yellow and green colors emphasize the elements that are significantly positively changed or significantly negatively changed in each of the clusters. Click here for file

Additional file 6: Figure S5 Visualization of ionomic gene interaction networks for all elements quantified in the ionome of yeast in the knockout collection (KO). For each element quantified in the KO data genes that significantly affect the abundance of that element were selected and an interaction network built based on known protein protein and genetic interactions. Protein protein and genetic interaction information were obtained from BioGRID [48]. Nodes represent genes, node color represents the direction of changes in elemental abundance (magenta increase in abundance, blue decrease in abundance), and node size represents the magnitude of the change in the ionome based on moderated Z-score. Lines joining the nodes (edges) in the graph represent the interactions. The type of line used for the edge represents the type of known interaction between the pairs of genes, with a dotted line representing a genetic interaction, and solid line represents a physical interaction. Numbers on the edges represent the correlation between connected nodes (genes) based on the ionomic profiles of the loss of function mutants in genes represented by the nodes. Only networks are shown with at least 2 nodes and 1 edge. Click here for file
