# Supplementary figures and images for: High-resolution genome-wide scan of genes, gene-networks and cellular systems impacting the yeast ionome
Source: BMC Genomics. 2012 Nov 14;13:623. doi: 10.1186/1471-2164-13-623 (PMC3652779; doi:10.1186/1471-2164-13-623)

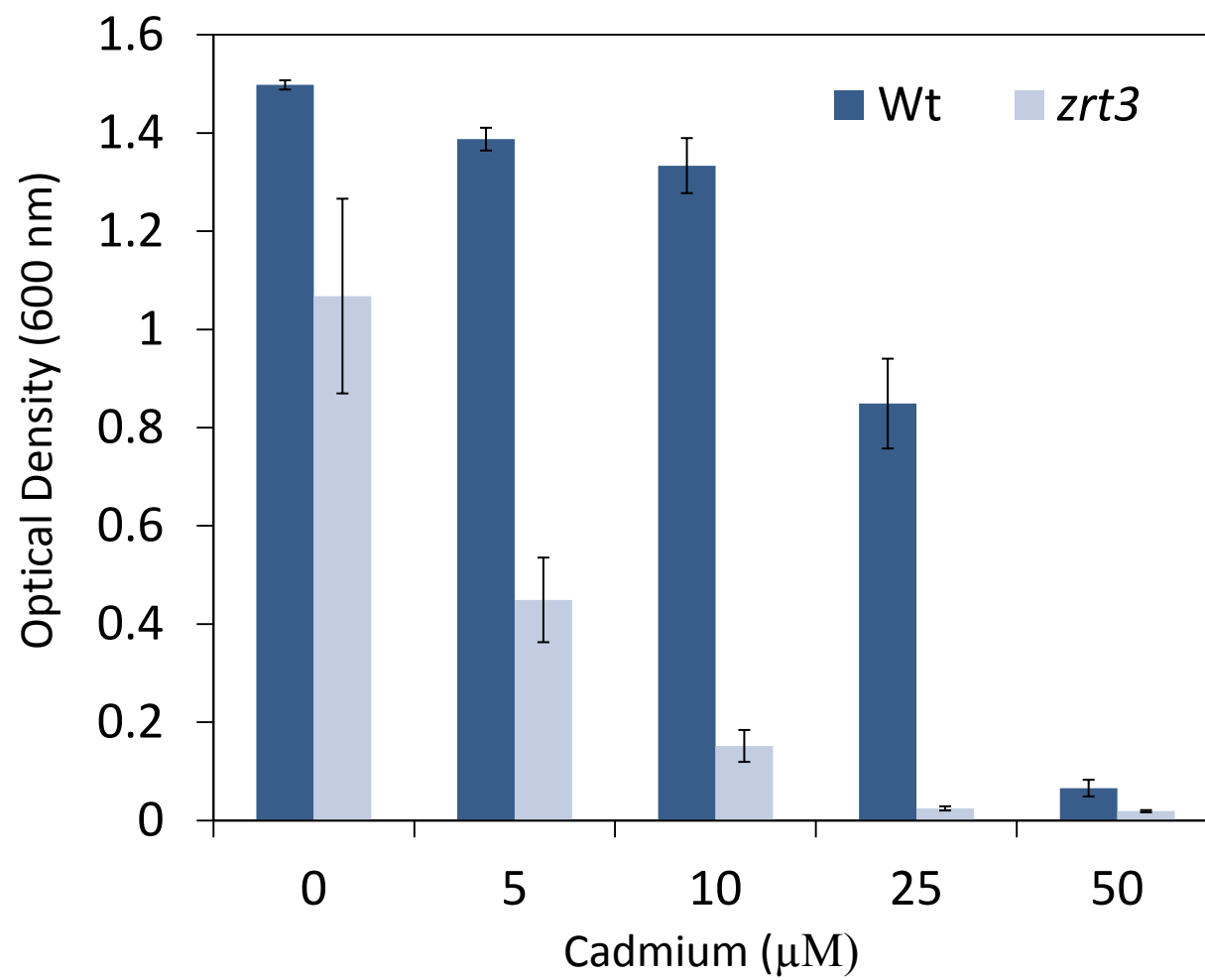

Supplement: Additional file 2: Figure S1 — Growth of yeast wild-type and zrt3 deletion mutant in the presence of Cd in the growth media. Yeast were grown in YNB at 30°C with shaking (200 rpm) in the presence of various concentrations of Cd2+ and optical density measured after 48hr. Data represents means (n = 9) ± standard deviations. [file 1471-2164-13-623-S2.pdf]

Color Key

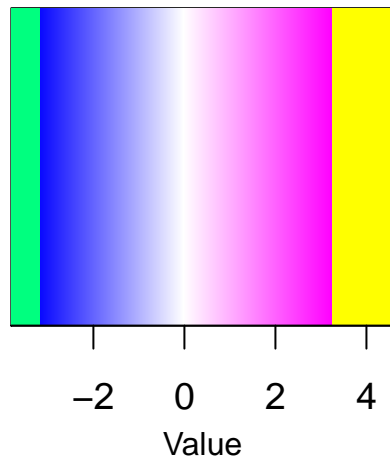KOd: refLine (-3.527,3.572)  
Cluster Medians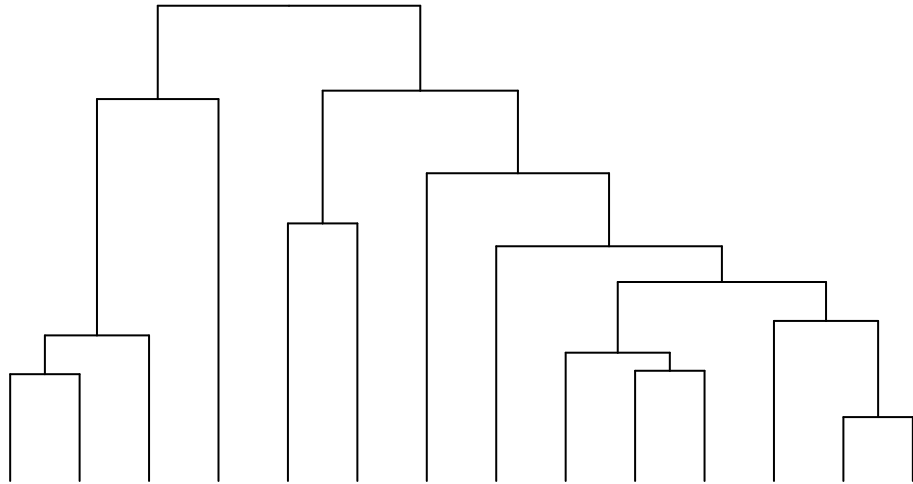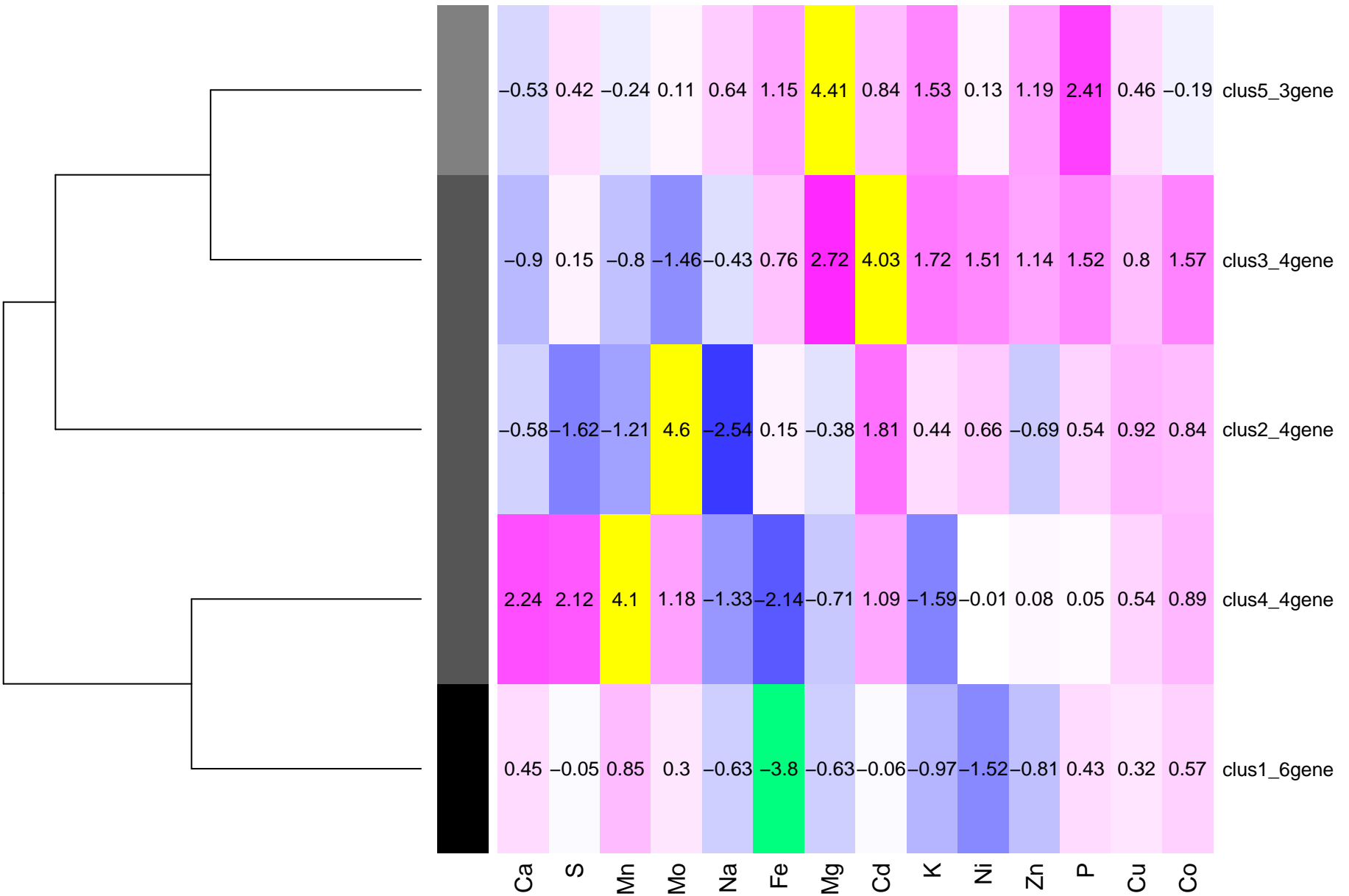

Supplement: Additional file 5: Figure S4 — Median elemental abundances (quantified as moderated Z-scores) of clusters generated using exhaustive significance clustering (ESC) on the ionome of yeast mutants from the KOd (A) or OE (B) data sets are visualized using a heat map. The clusters including less than three genes are not shown. Clusters are represented in rows and elements are represented in columns. The dendrogram represents relationships between cluster (left) and elements (top) using hierarchical clustering. The grey-scale shading on the left of the heat map visually represents the number of genes in a cluster (darker represents more genes). Numbers on the heat map are moderated Z-scores for each element within each cluster. The highlight color of the numbers represents the magnitude of the abundance - green if the median elemental is less than −3.527 (A) or −3.801 (B) , yellow if it is greater than 3.572 (A) or 3.735 (B), a gradient of blue if between the lower significance cut of and 0, and a gradient of magenta if between 0 and the upper significance cut off. The yellow and green colors emphasize the elements that are significantly positively changed or significantly negatively changed in each of the clusters. [file 1471-2164-13-623-S5.zip › Figure S4A.pdf]

Color Key

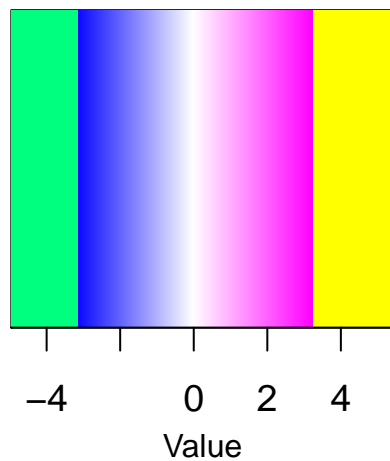

OE: refLine (-3.801,3.735)  
Cluster Medians

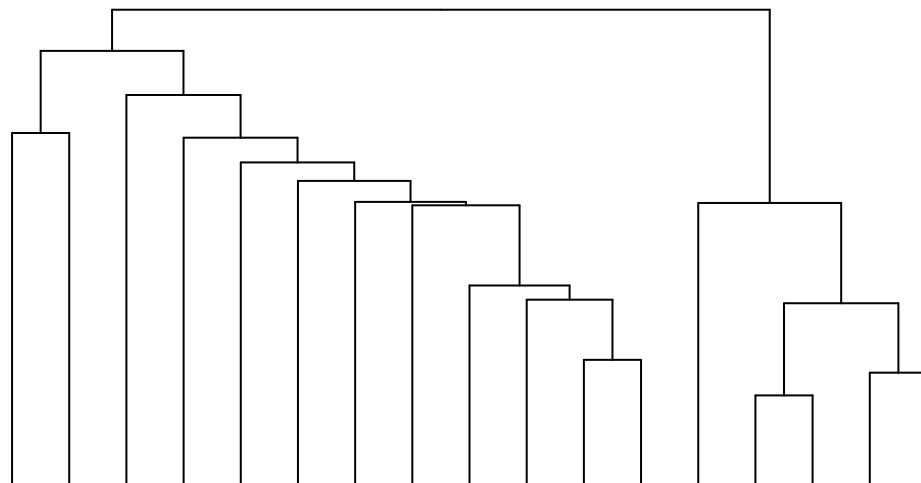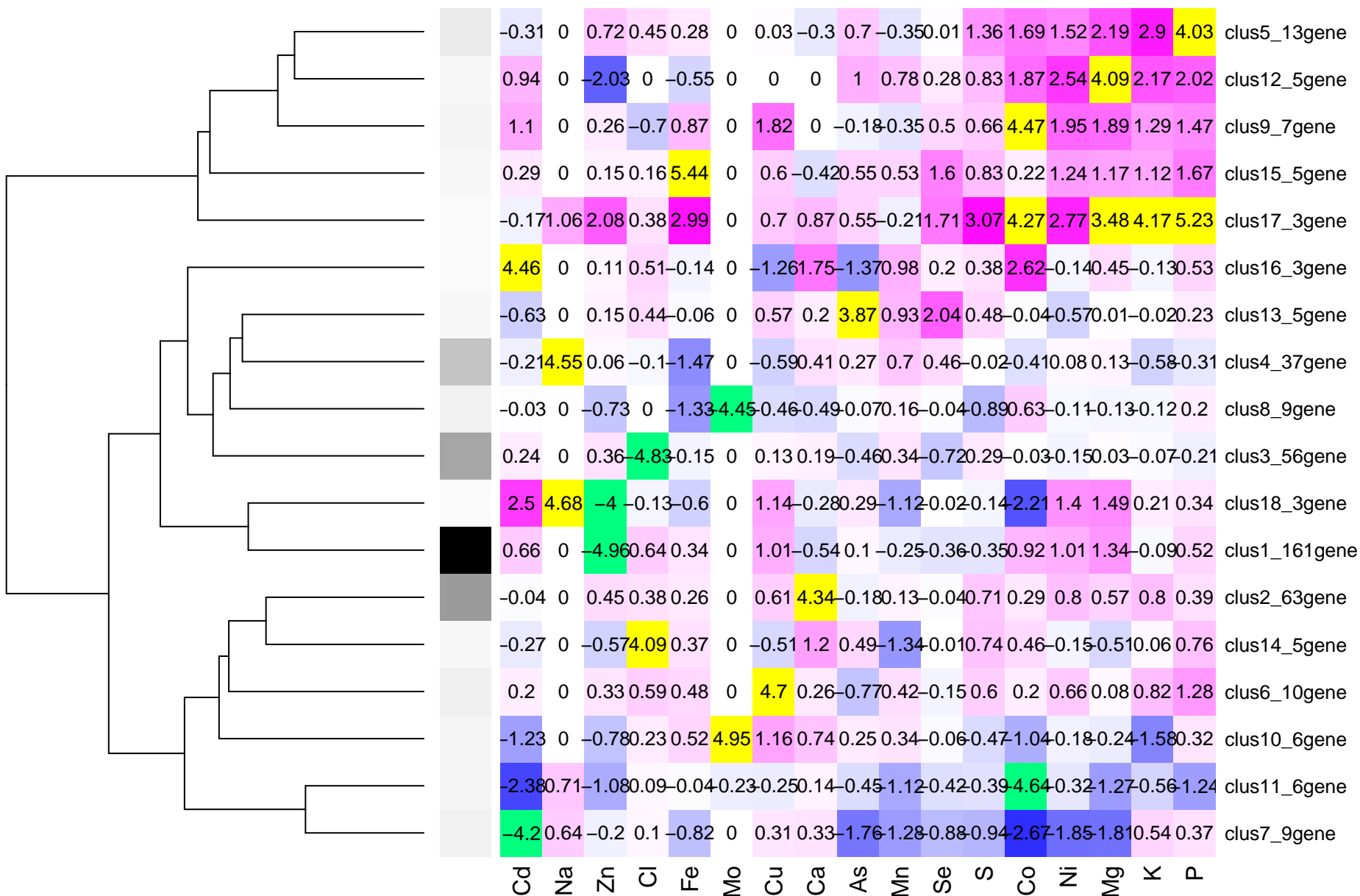

Supplement: Additional file 5: Figure S4 — Median elemental abundances (quantified as moderated Z-scores) of clusters generated using exhaustive significance clustering (ESC) on the ionome of yeast mutants from the KOd (A) or OE (B) data sets are visualized using a heat map. The clusters including less than three genes are not shown. Clusters are represented in rows and elements are represented in columns. The dendrogram represents relationships between cluster (left) and elements (top) using hierarchical clustering. The grey-scale shading on the left of the heat map visually represents the number of genes in a cluster (darker represents more genes). Numbers on the heat map are moderated Z-scores for each element within each cluster. The highlight color of the numbers represents the magnitude of the abundance - green if the median elemental is less than −3.527 (A) or −3.801 (B) , yellow if it is greater than 3.572 (A) or 3.735 (B), a gradient of blue if between the lower significance cut of and 0, and a gradient of magenta if between 0 and the upper significance cut off. The yellow and green colors emphasize the elements that are significantly positively changed or significantly negatively changed in each of the clusters. [file 1471-2164-13-623-S5.zip › Figure S4B.pdf]
