# Supplementary material for: High-resolution genome-wide scan of genes, gene-networks and cellular systems impacting the yeast ionome
Source: BMC Genomics. 2012 Nov 14;13:623. doi: 10.1186/1471-2164-13-623 (PMC3652779; doi:10.1186/1471-2164-13-623)

Directed Acyclic Graph of the 8 significant GO terms of the 48 genes in KO screen, Group C, CC

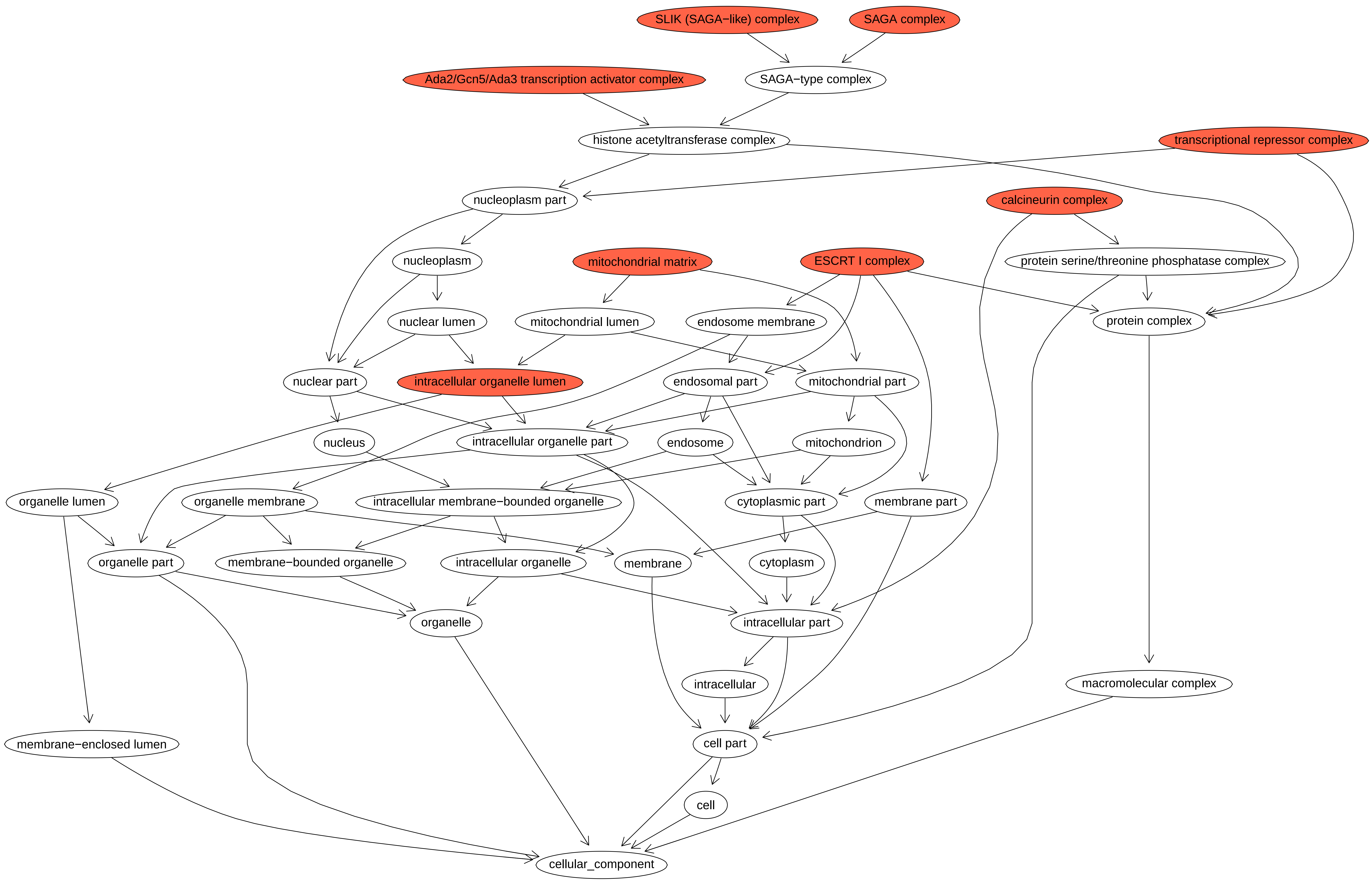

Directed Acyclic Graph of the 27 significant GO terms of the 48 genes in KO screen, Group C, BP

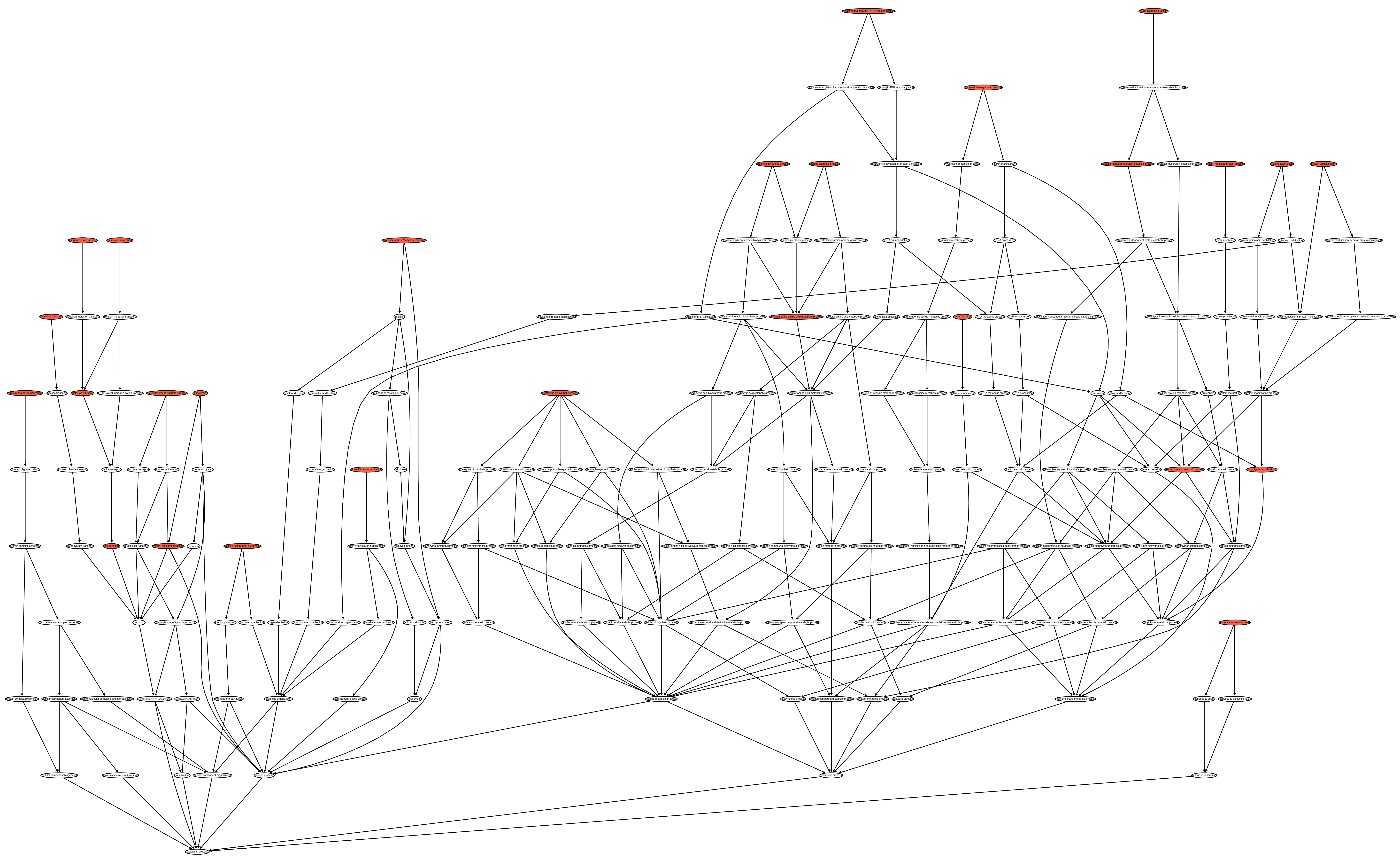

Directed Acyclic Graph of the 34 significant GO terms of the 48 genes in KO screen, Group C, MF

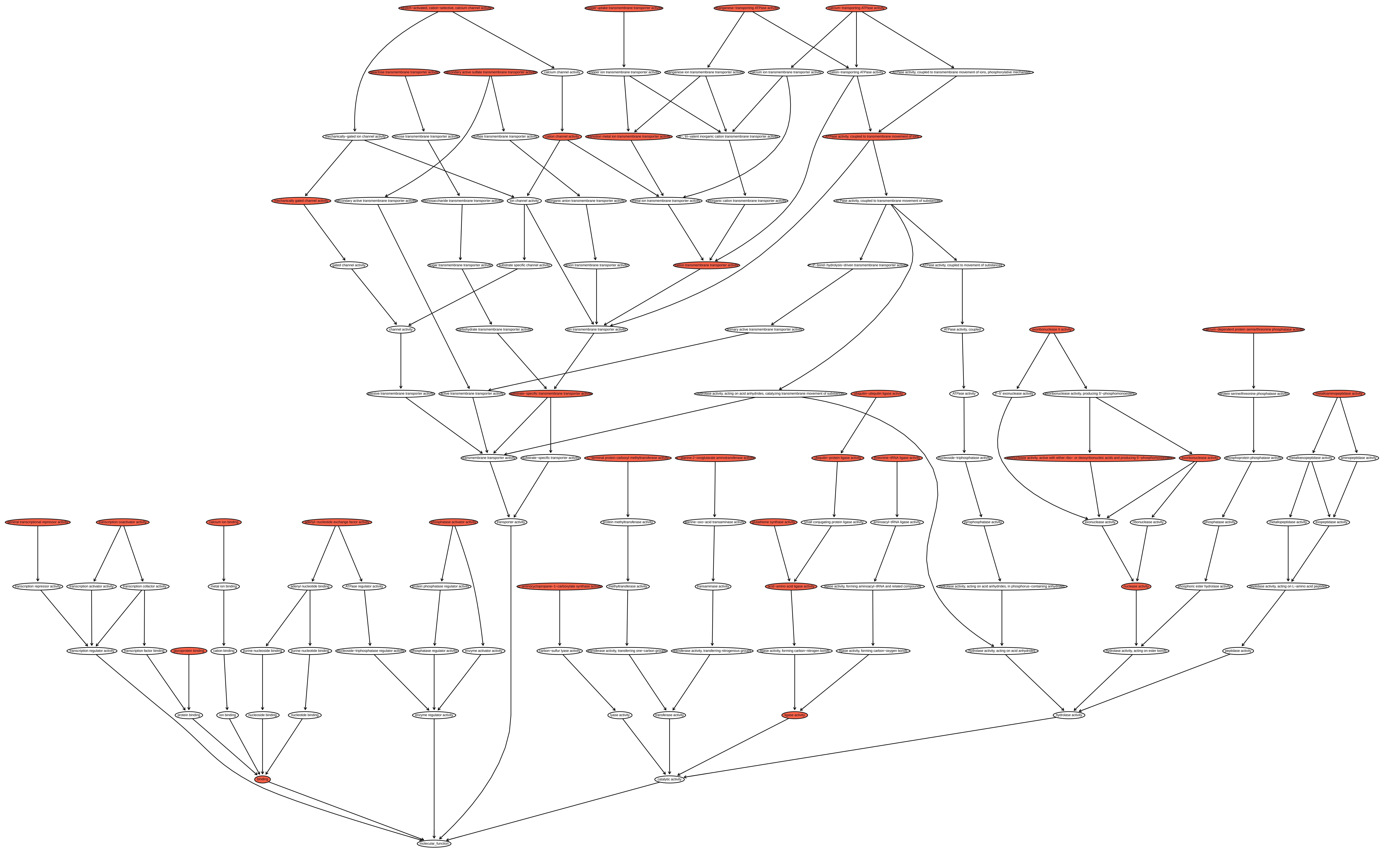

Directed Acyclic Graph of the 48 significant GO terms of the 516 genes in KO screen, Group B, CC

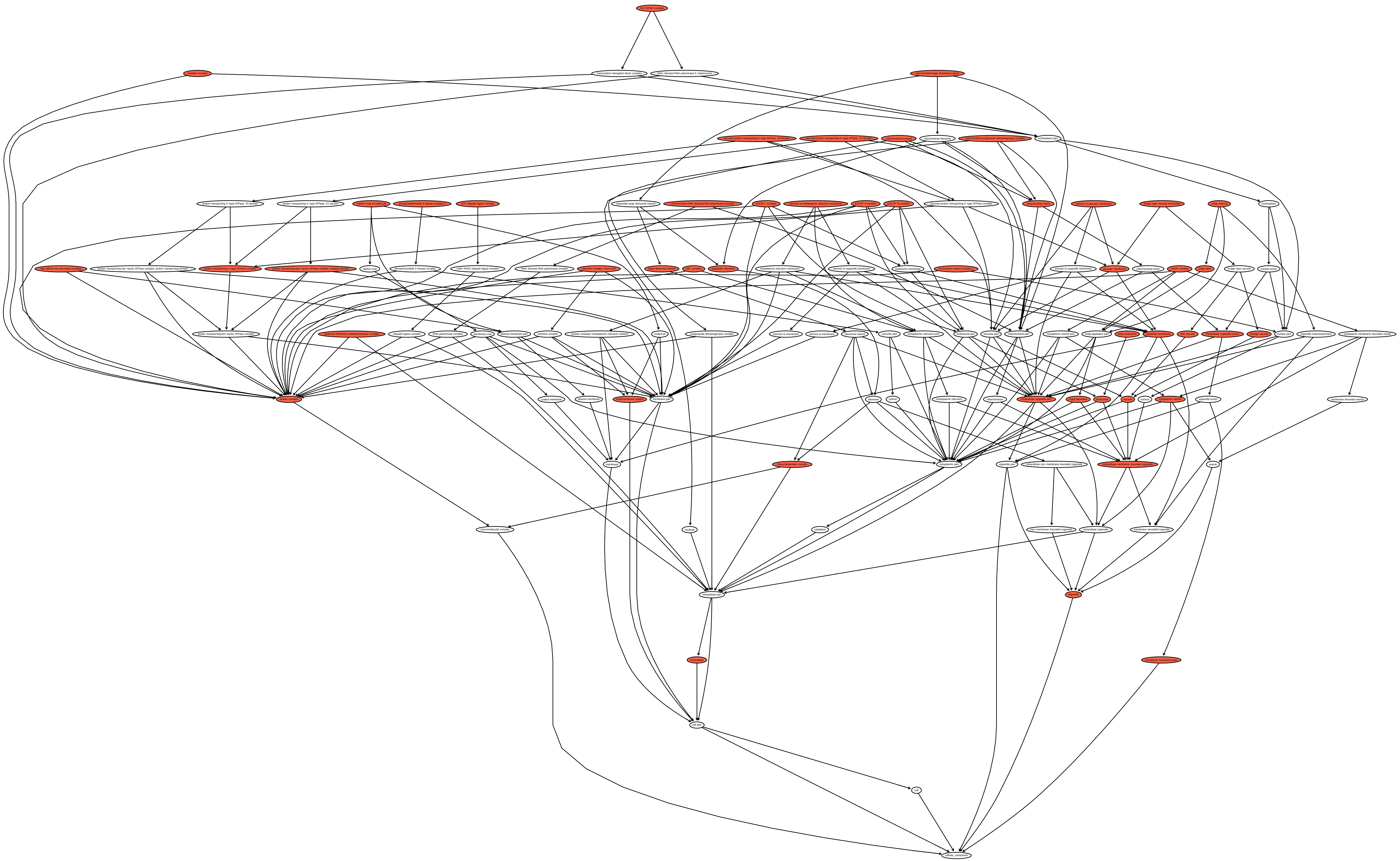

Directed Acyclic Graph of the 132 significant GO terms of the 516 genes in KO screen, Group B, BP

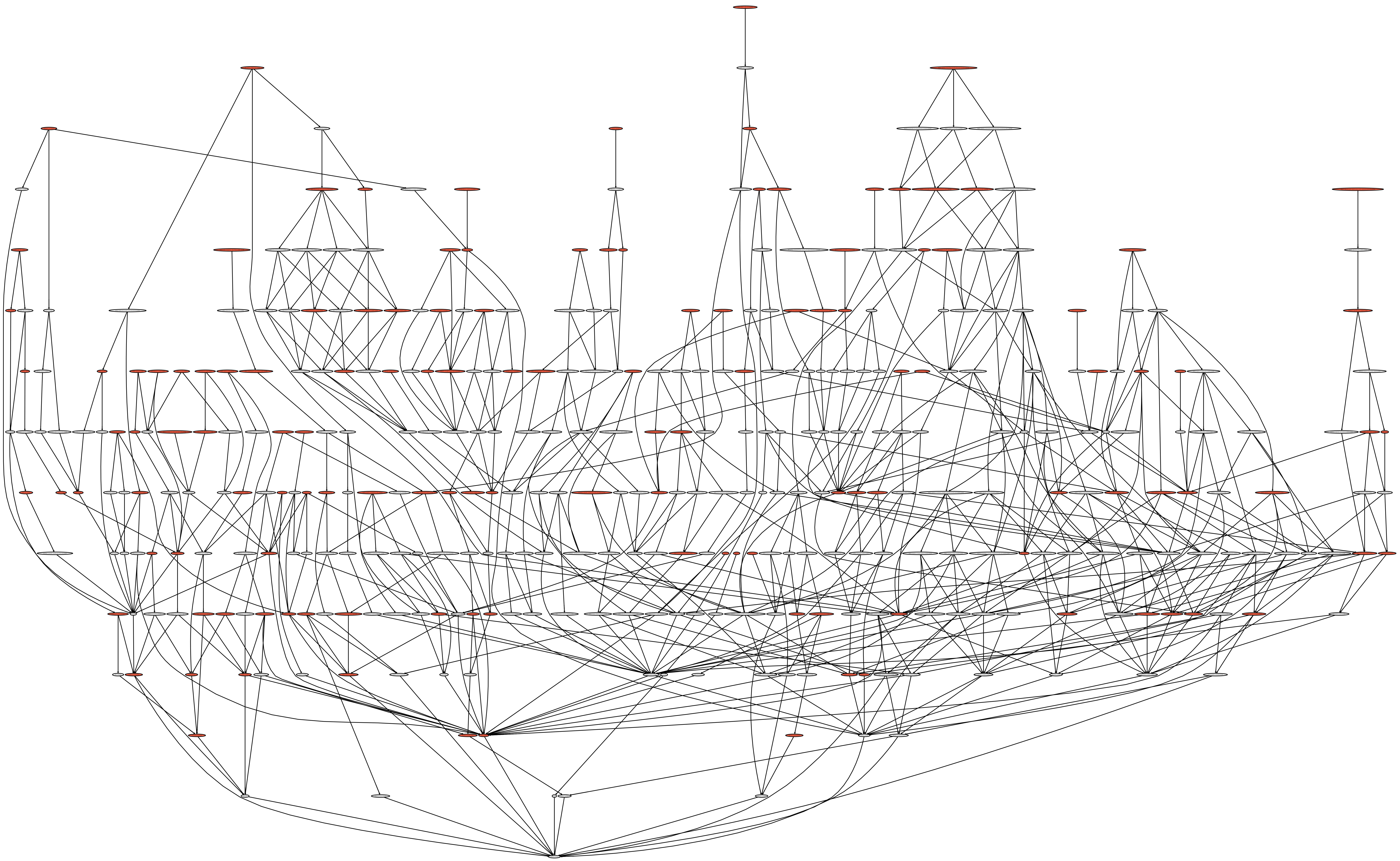

Directed Acyclic Graph of the 35 significant GO terms of the 516 genes in KO screen, Group B, MF

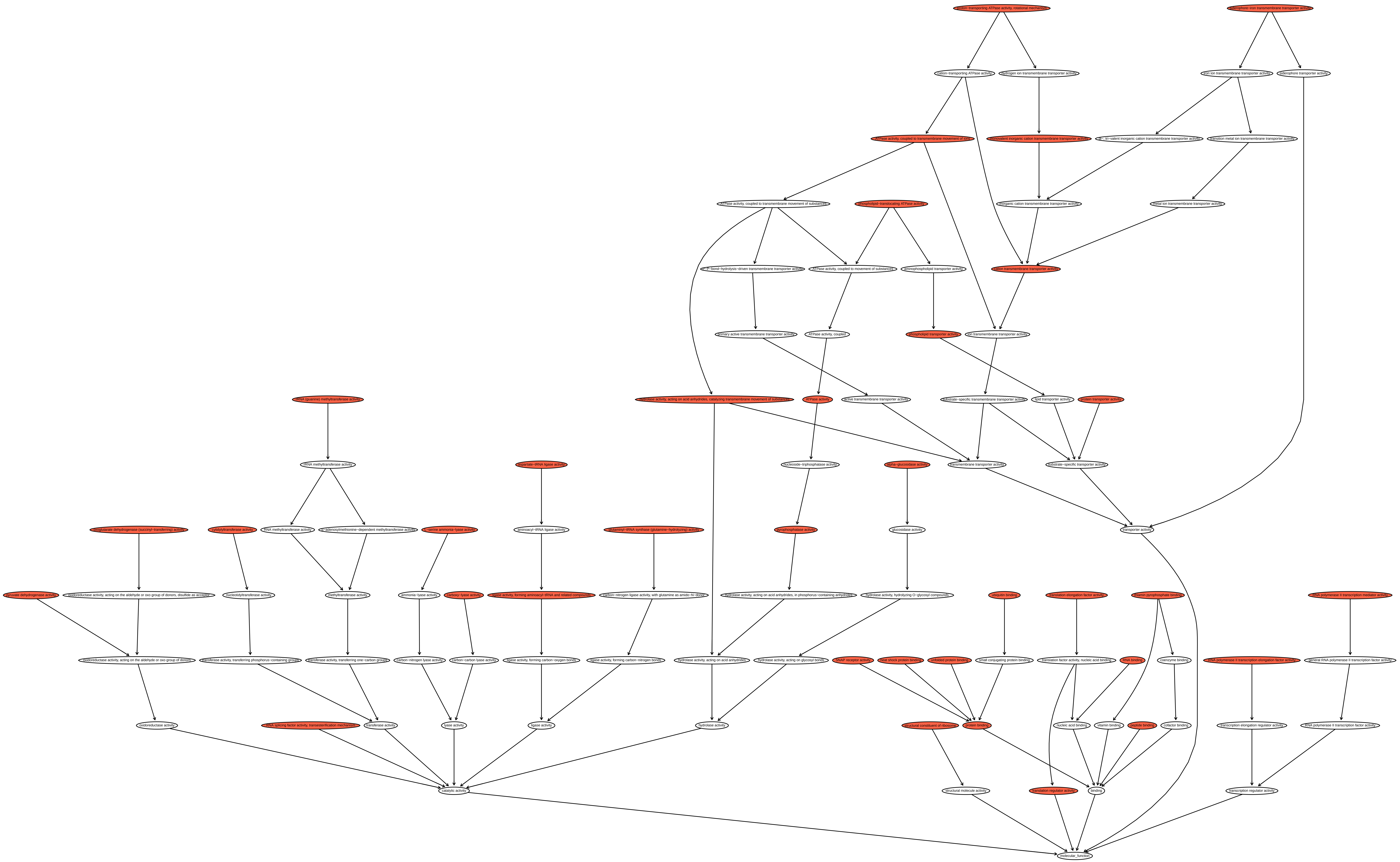

Directed Acyclic Graph of the 6 significant GO terms of the 36 genes in KO screen, Group A, CC

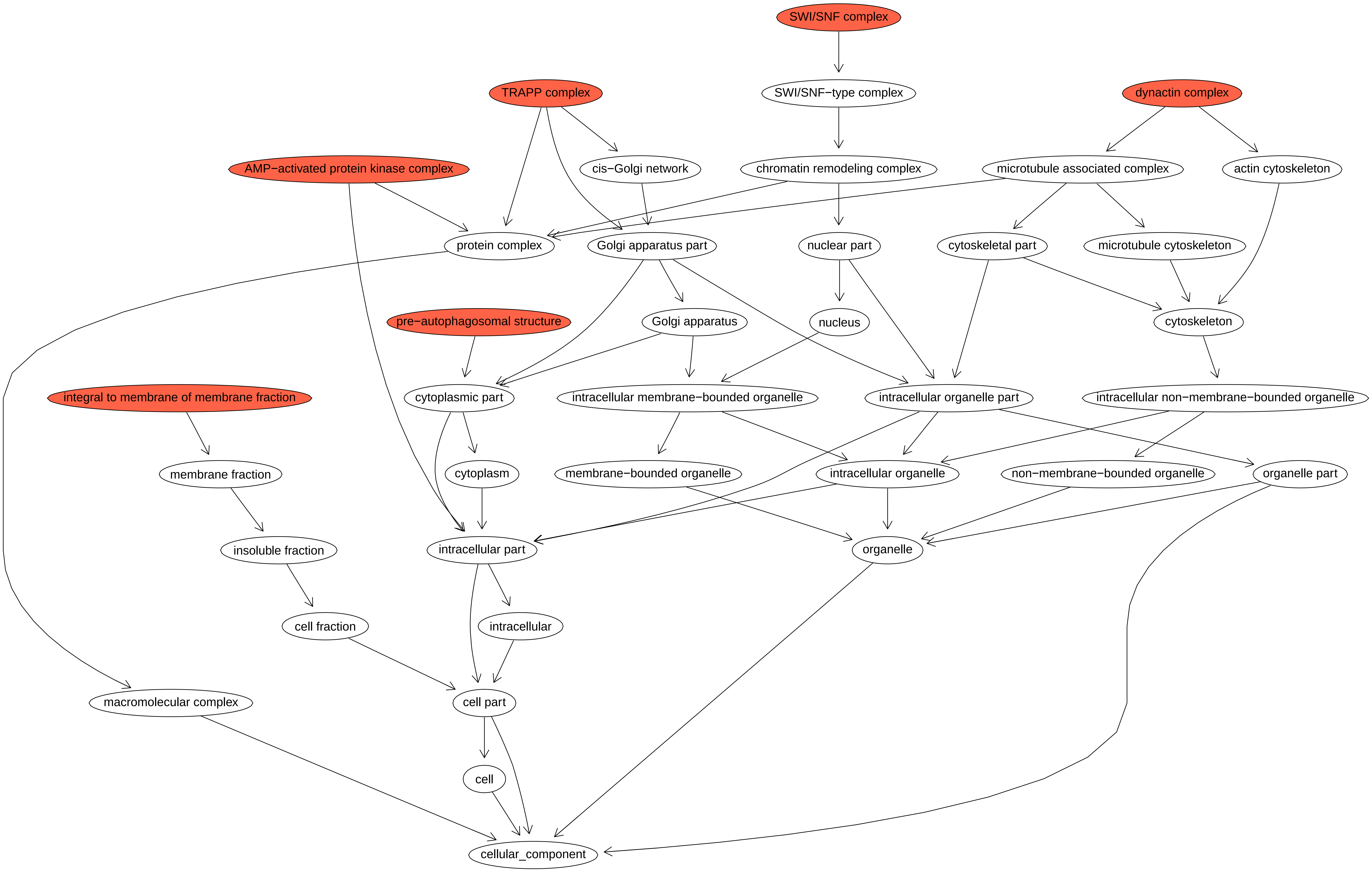

Directed Acyclic Graph of the 24 significant
GO terms of the 36 genes in KO screen, Group A, BP

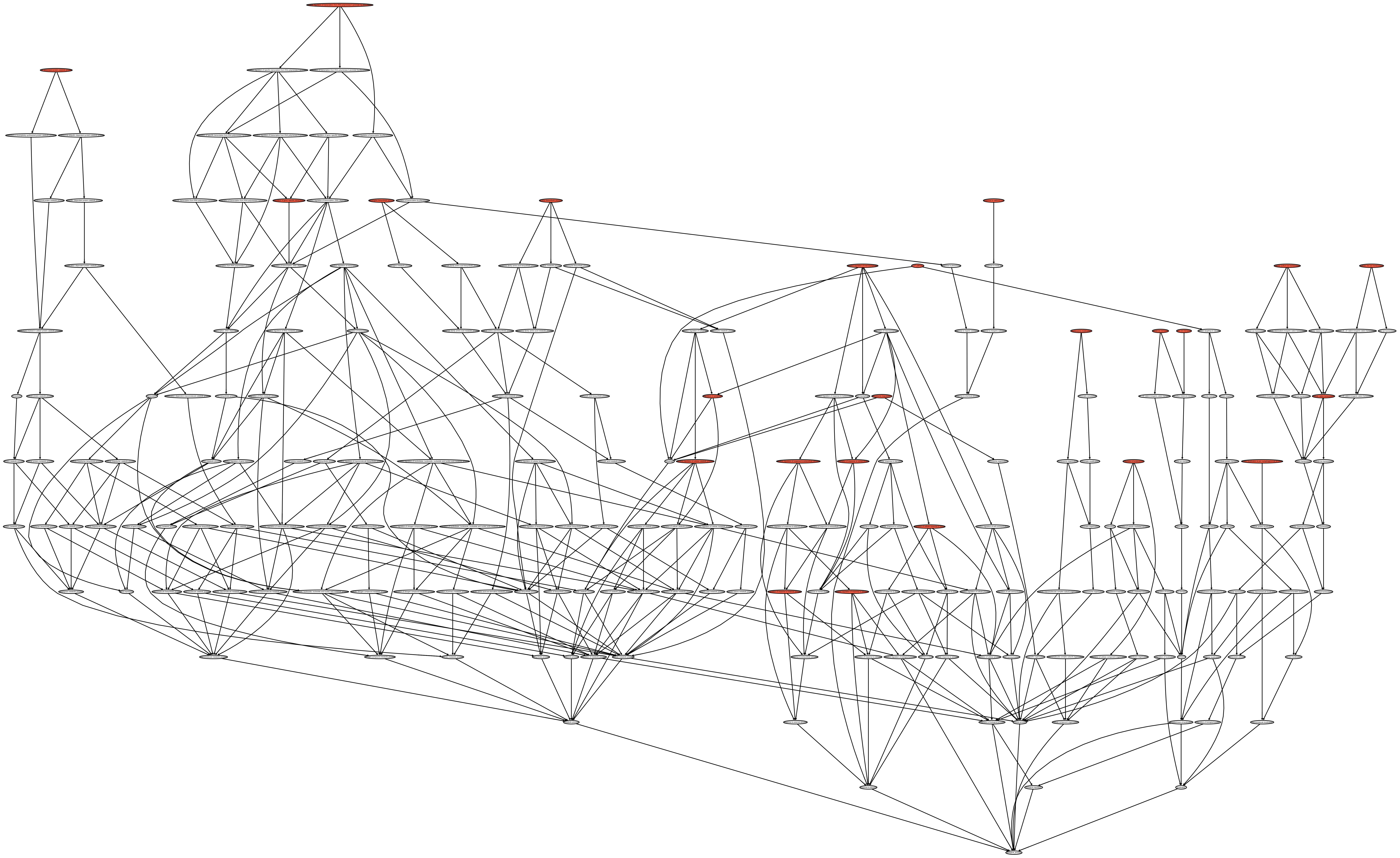

Directed Acyclic Graph of the 17 significant GO terms of the 36 genes in KO screen, Group A, MF

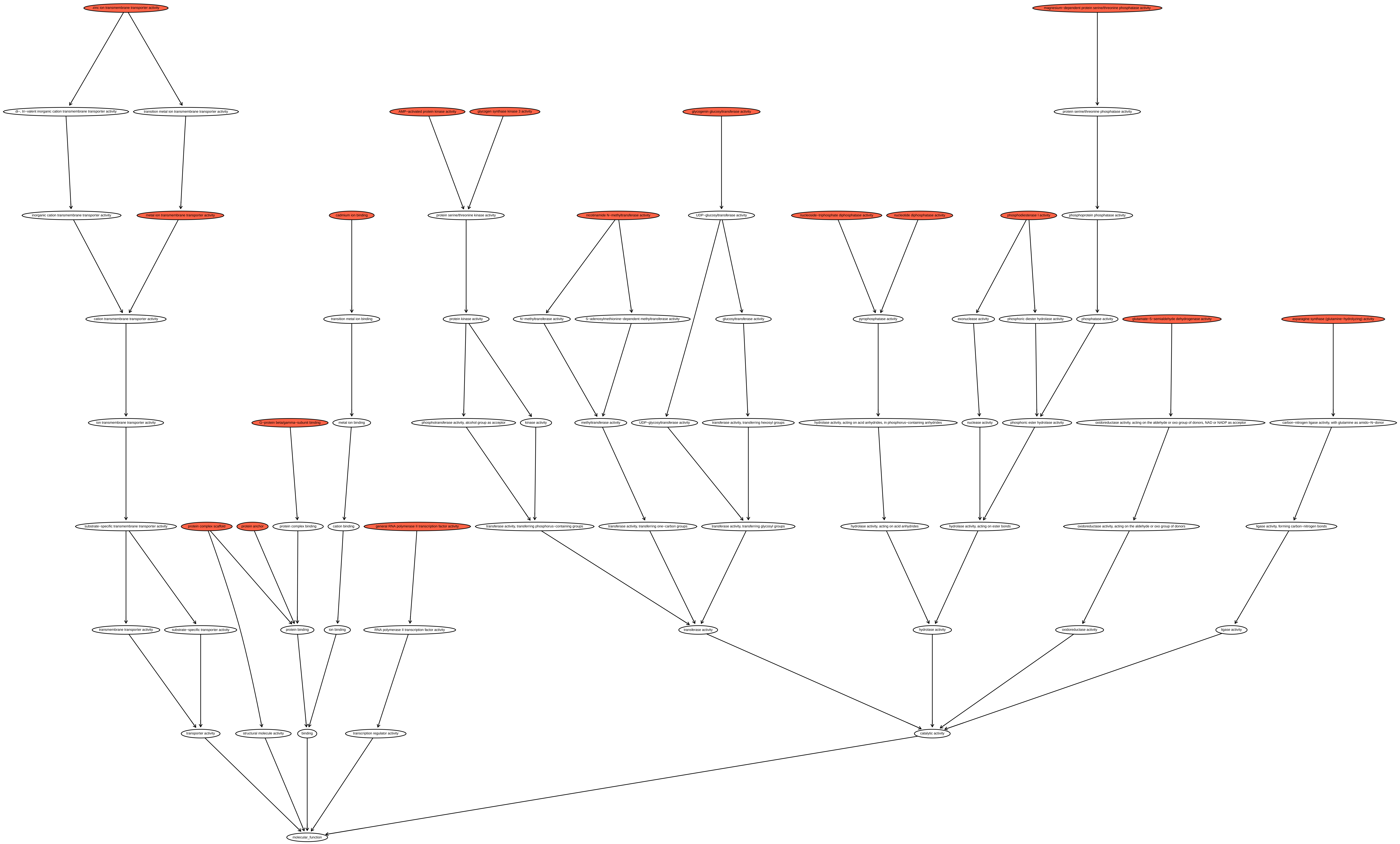

Supplement: Additional file 3: Figure S2 — Directed Acyclic Graph (DAG) and pie charts for Gene Ontology (GO) data for KO (A &B), KOd (C &D) and OE (D &F) gene datasets. The R packages GOstats, Rgraphviz and graphics were utilized to perform GO enrichment, generate the DAG plots and the pie plots. [file 1471-2164-13-623-S3.zip › Figure S2A.pdf]
