# Supplementary material for: High-resolution genome-wide scan of genes, gene-networks and cellular systems impacting the yeast ionome
Source: BMC Genomics. 2012 Nov 14;13:623. doi: 10.1186/1471-2164-13-623 (PMC3652779; doi:10.1186/1471-2164-13-623)

KO group C 48 genes, CC
pieCharts ID, Counts, P−values and GO terms (left)

- 1, 2, 0.00161, Ada2/Gcn5/Ada3 transcription activator complex
- 2, 2, 0.00572, SLIK (SAGA−like) complex
- 3, 2, 0.00928, SAGA complex
- 4, 6, 0.00957, mitochondrial matrix
- 5, 1, 0.02115, transcriptional repressor complex
- 6, 1, 0.03156, calcineurin complex
- 7, 9, 0.03868, intracellular organelle lumen
- 8, 1, 0.04186, ESCRT I complex

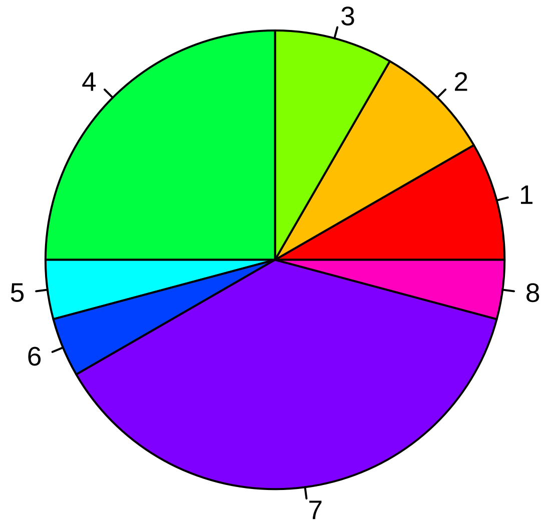

KO group C 48 genes, BP
pieCharts ID, Counts, P−values and GO terms (left)

- 1, 2, 0.00161, calcium ion transport
- 2, 2, 0.00224, manganese ion transport
- 3, 13, 0.00291, biopolymer modification
- 4, 4, 0.00822, metal ion transport
- 5, 1, 0.01063, intron homing
- 6, 1, 0.01063, alanine biosynthetic process
- 7, 1, 0.01063, alanine catabolic process
- 8, 1, 0.01063, pyruvate family amino acid metabolic process
- 9, 1, 0.01063, intein−mediated protein splicing
- 10, 1, 0.01063, mitochondrial threonyl−tRNA aminoacylation
- 11, 6, 0.01272, ion transport
- 12, 7, 0.01316, vesicle−mediated transport
- 13, 4, 0.01362, response to osmotic stress
- 14, 3, 0.01641, actin filament organization
- 15, 1, 0.02115, glutathione biosynthetic process
- 16, 1, 0.02115, galactose transport
- 17, 2, 0.02231, mitotic metaphase/anaphase transition
- 18, 3, 0.02309, protein ubiquitination
- 19, 2, 0.02427, vacuole fusion, non−autophagic
- 20, 20, 0.02938, cellular protein metabolic process
- 21, 2, 0.03983, histone acetylation
- 22, 1, 0.04186, cyclin catabolic process
- 23, 1, 0.04186, wybutosine biosynthetic process
- 24, 1, 0.04186, protein homooligomerization
- 25, 4, 0.04367, ubiquitin−dependent protein catabolic process
- 26, 2, 0.04481, late endosome to vacuole transport
- 27, 2, 0.04738, exocytosis

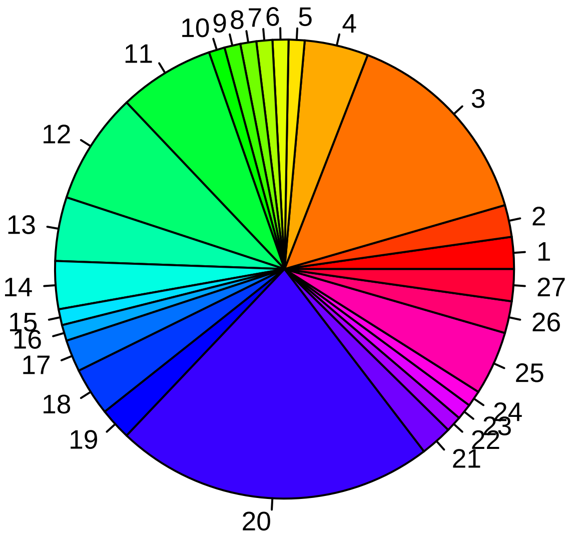

KO group C 48 genes, MF
pieCharts ID, Counts, P−values and GO terms (left)

1, 6, 0.00196, ligase activity
2, 4, 0.00514, acid−amino acid ligase activity
3, 3, 0.0058, calcium ion binding
4, 2, 0.00801, exoribonuclease activity
5, 1, 0.01063, glycoprotein binding
6, 1, 0.01063, glutathione synthase activity
7, 1, 0.01063, threonine−tRNA ligase activity
8, 1, 0.01063, exoribonuclease II activity
9, 1, 0.01063, copper uptake transmembrane transporter activity
10, 1, 0.01063, stretch−activated, cation−selective, calcium channel activity
11, 1, 0.01063, manganese−transporting ATPase activity
12, 1, 0.01063, mechanically gated channel activity
13, 2, 0.01063, transcription coactivator activity
14, 2, 0.01518, exonuclease activity, active with either ribo− or deoxyribonucleic acids and producing 5'−phosphomonoesters
15, 3, 0.02086, ubiquitin−protein ligase activity
16, 1, 0.02115, L−alanine:2−oxoglutarate aminotransferase activity
17, 1, 0.02115, calcium−transporting ATPase activity
18, 1, 0.02115, general transcriptional repressor activity
19, 1, 0.02115, ubiquitin−ubiquitin ligase activity
20, 28, 0.02172, binding
21, 3, 0.02984, nuclease activity
22, 1, 0.03156, C−terminal protein carboxyl methyltransferase activity
23, 1, 0.03156, calcium−dependent protein serine/threonine phosphatase activity
24, 1, 0.03156, 1−aminocyclopropane−1−carboxylate synthase activity
25, 4, 0.03191, cation transmembrane transporter activity
26, 2, 0.03279, transition metal ion transmembrane transporter activity
27, 2, 0.03983, ATPase activity, coupled to transmembrane movement of ions
28, 1, 0.04186, adenyl−nucleotide exchange factor activity
29, 1, 0.04186, cation channel activity
30, 1, 0.04186, galactose transmembrane transporter activity

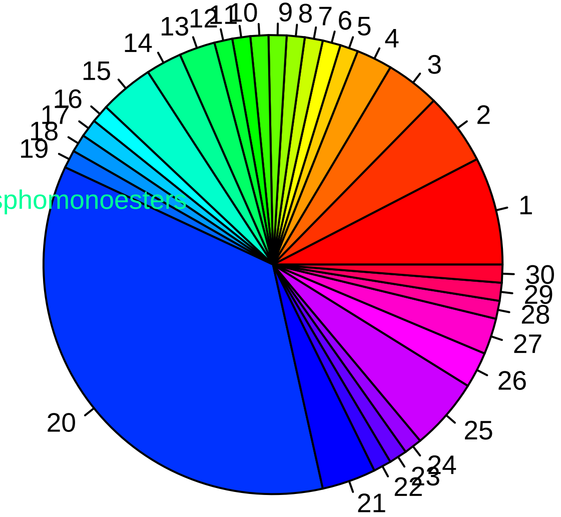

KO group B 516 genes, CC
pieCharts ID, Counts, P−values and GO terms (left)

- 1, 27, 0, endosome
- 2, 9, 0, proton−transporting V−type ATPase complex
- 3, 353, 0, organelle
- 4, 74, 1e−05, intracellular organelle lumen
- 5, 73, 1e−05, membrane−enclosed lumen
- 6, 132, 7e−05, intracellular organelle part
- 7, 4, 0.00012, ESCRT III complex
- 8, 4, 0.00012, GARP complex
- 9, 31, 0.00014, mitochondrial matrix
- 10, 5, 0.00022, Cdc73/Paf1 complex
- 11, 400, 0.00046, intracellular
- 12, 5, 0.00053, vacuolar proton−transporting V−type ATPase, V1 domain
- 13, 54, 0.00112, ribonucleoprotein complex
- 14, 3, 0.00115, ESCRT II complex
- 15, 3, 0.00115, transcription export complex 2
- 16, 4, 0.00151, vacuolar proton−transporting V−type ATPase, V0 domain
- 17, 37, 0.00183, endomembrane system
- 18, 22, 0.0025, large ribosomal subunit
- 19, 11, 0.00255, mitochondrial large ribosomal subunit
- 20, 16, 0.00262, organellar ribosome
- 21, 7, 0.00307, mitochondrial nucleoid
- 22, 3, 0.00424, ESCRT I complex
- 23, 3, 0.00424, phosphatidylinositol 3−kinase complex II
- 24, 5, 0.00527, proton−transporting two−sector ATPase complex, catalytic domain
- 25, 32, 0.00619, Golgi apparatus
- 26, 16, 0.00688, fungal−type vacuole membrane
- 27, 59, 0.00767, organelle membrane
- 28, 2, 0.01099, high affinity iron permease complex
- 29, 2, 0.01099, mitochondrial DNA−directed RNA polymerase complex
- 30, 5, 0.01115, mediator complex

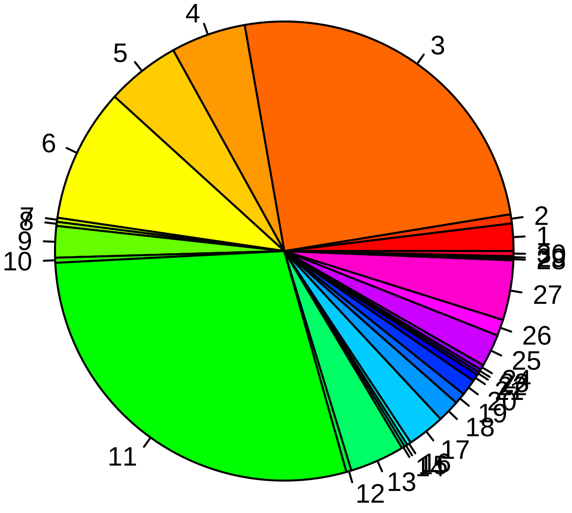

KO group B 516 genes, BP
pieCharts ID, Counts, P−values and GO terms (left)

- 1, 13, 0, vacuolar acidification
- 2, 13, 0, pH reduction
- 3, 13, 0, regulation of intracellular pH
- 4, 31, 0, cellular ion homeostasis
- 5, 29, 0, cation homeostasis
- 6, 55, 0, cellular macromolecule localization
- 7, 36, 0, chemical homeostasis
- 8, 34, 0, cellular homeostasis
- 9, 20, 0, protein targeting to vacuole
- 10, 71, 0, protein localization
- 11, 9, 0, ubiquitin−dependent protein catabolic process via the multivesicular body sorting pathway
- 12, 13, 1e−05, cellular monovalent inorganic cation homeostasis
- 13, 13, 1e−05, late endosome to vacuole transport
- 14, 11, 1e−05, Golgi to vacuole transport
- 15, 5, 1e−05, intralumenal vesicle formation
- 16, 28, 2e−05, response to drug
- 17, 34, 2e−05, vesicle−mediated transport
- 18, 7, 3e−05, protein retention in Golgi apparatus
- 19, 44, 4e−05, cellular localization
- 20, 4, 0.00012, vacuolar proton−transporting V−type ATPase complex assembly
- 21, 60, 0.00021, regulation of biological quality
- 22, 298, 0.00026, primary metabolic process
- 23, 17, 0.00036, intracellular transport
- 24, 20, 0.00036, mitochondrial translation
- 25, 27, 0.00038, autophagy
- 26, 126, 0.00041, cellular protein metabolic process
- 27, 129, 0.00046, establishment of localization
- 28, 9, 0.00063, ATP synthesis coupled proton transport
- 29, 17, 8e−04, nucleic acid transport
- 30, 17, 8e−04, establishment of RNA localization

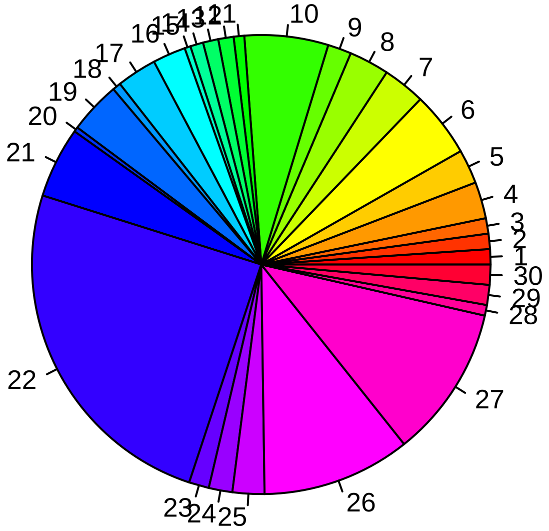

KO group B 516 genes, MF
pieCharts ID, Counts, P−values and GO terms (left)

- 1, 9, 3e−05, proton−transporting ATPase activity, rotational mechanism
- 2, 6, 0.00038, translation elongation factor activity
- 3, 10, 0.00063, ATPase activity, coupled to transmembrane movement of ions
- 4, 34, 0.00067, structural constituent of ribosome
- 5, 5, 0.0011, RNA polymerase II transcription elongation factor activity
- 6, 4, 0.00323, thiamin pyrophosphate binding
- 7, 3, 0.00424, siderophore−iron transmembrane transporter activity
- 8, 21, 0.00678, cation transmembrane transporter activity
- 9, 13, 0.00802, unfolded protein binding
- 10, 3, 0.00976, alpha−glucosidase activity
- 11, 2, 0.01099, oxoglutarate dehydrogenase (succinyl−transferring) activity
- 12, 2, 0.01099, aspartate−tRNA ligase activity
- 13, 2, 0.01099, rRNA (guanine) methyltransferase activity
- 14, 2, 0.01099, cytidylyltransferase activity
- 15, 5, 0.011, carboxy−lyase activity
- 16, 5, 0.01115, peptide binding
- 17, 11, 0.01695, monovalent inorganic cation transmembrane transporter activity
- 18, 7, 0.018, protein transporter activity
- 19, 5, 0.02648, ligase activity, forming aminoacyl−tRNA and related compounds
- 20, 4, 0.02986, ubiquitin binding
- 21, 2, 0.03067, L−serine ammonia−lyase activity
- 22, 2, 0.03067, pyruvate dehydrogenase activity
- 23, 2, 0.03067, glutaminyl−tRNA synthase (glutamine−hydrolyzing) activity
- 24, 36, 0.03137, pyrophosphatase activity
- 25, 5, 0.0336, RNA polymerase II transcription mediator activity
- 26, 5, 0.0336, RNA splicing factor activity, transesterification mechanism
- 27, 25, 0.03619, ATPase activity
- 28, 10, 0.03631, hydrolase activity, acting on acid anhydrides, catalyzing transmembrane movement of substances
- 29, 4, 0.03965, SNAP receptor activity
- 30, 4, 0.03965, phospholipid transporter activity

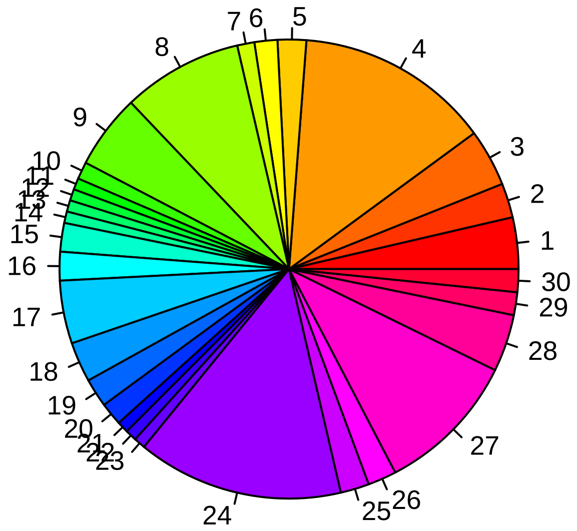

KO group A 36 genes, CC
pieCharts ID, Counts, P−values and GO terms (left)

- 1, 2, 0.00156, SWI/SNF complex
- 2, 2, 0.011, pre−autophagosomal structure
- 3, 1, 0.02289, integral to membrane of membrane fraction
- 4, 1, 0.03041, dynactin complex
- 5, 1, 0.03041, TRAPP complex
- 6, 1, 0.03787, AMP−activated protein kinase complex
- 7, 1, 0.05263, prefoldin complex
- 8, 1, 0.05993, astral microtubule
- 9, 1, 0.05993, RSC complex
- 10, 1, 0.08149, peripheral to membrane of membrane fraction
- 11, 16, 0.08278, membrane
- 12, 4, 0.08281, vacuole

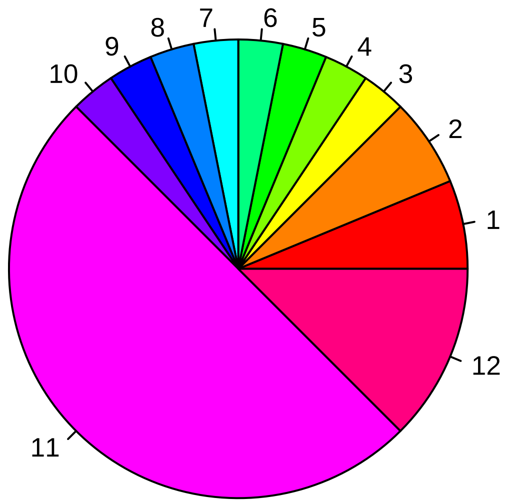

KO group A 36 genes, BP
pieCharts ID, Counts, P−values and GO terms (left)

1, 2, 0.00034, protein localization to pre−autophagosomal structure
2, 2, 0.002, zinc ion transport
3, 2, 0.00248, cellular zinc ion homeostasis
4, 1, 0.00769, regulation of autophagy
5, 1, 0.00769, positive regulation of macroautophagy
6, 1, 0.00769, regulation of response to external stimulus
7, 1, 0.00769, positive regulation of response to extracellular stimulus
8, 1, 0.00769, regulation of response to nutrient levels
9, 1, 0.00769, nucleosome mobilization
10, 2, 0.01, CVT pathway
11, 1, 0.01532, cellular sodium ion homeostasis
12, 1, 0.01532, positive regulation of transcription from RNA polymerase II promoter by pheromones
13, 2, 0.01541, regulation of gene−specific transcription
14, 1, 0.02289, proline biosynthetic process
15, 1, 0.02289, cobalt ion transport
16, 1, 0.02289, regulation of endocytosis
17, 2, 0.02761, ER−associated protein catabolic process
18, 1, 0.03041, asparagine biosynthetic process
19, 1, 0.03041, positive regulation of cellular catabolic process
20, 3, 0.03555, cellular cation homeostasis
21, 1, 0.03787, positive regulation of response to stimulus
22, 1, 0.04528, peroxisome degradation
23, 1, 0.04528, response to cadmium ion
24, 1, 0.04528, regulation of cellular response to stress
25, 3, 0.05016, cellular chemical homeostasis
26, 2, 0.05194, regulation of catabolic process
27, 1, 0.05263, tubulin complex assembly
28, 1, 0.05263, regulation of transcription by pheromones
29, 2, 0.05587, ER to Golgi vesicle−mediated transport
30, 2, 0.05788, glutamine family amino acid metabolic process

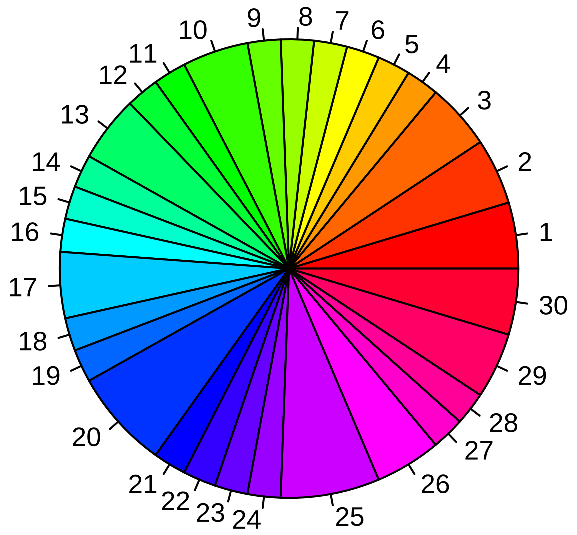

KO group A 36 genes, MF
pieCharts ID, Counts, P−values and GO terms (left)

- 1, 2, 0.002, zinc ion transmembrane transporter activity
- 2, 1, 0.00769, glutamate−5−semialdehyde dehydrogenase activity
- 3, 1, 0.00769, nicotinamide N−methyltransferase activity
- 4, 1, 0.00769, G−protein beta/gamma−subunit binding
- 5, 1, 0.01532, phosphodiesterase I activity
- 6, 1, 0.01532, glycogenin glucosyltransferase activity
- 7, 1, 0.01532, cadmium ion binding
- 8, 2, 0.01662, general RNA polymerase II transcription factor activity
- 9, 1, 0.02289, asparagine synthase (glutamine−hydrolyzing) activity
- 10, 1, 0.02289, magnesium−dependent protein serine/threonine phosphatase activity
- 11, 1, 0.03041, glycogen synthase kinase 3 activity
- 12, 1, 0.03041, protein complex scaffold
- 13, 1, 0.03041, nucleoside−triphosphate diphosphatase activity
- 14, 1, 0.03787, nucleotide diphosphatase activity
- 15, 1, 0.03787, AMP−activated protein kinase activity
- 16, 2, 0.04079, metal ion transmembrane transporter activity
- 17, 1, 0.04528, protein anchor
- 18, 3, 0.0548, protein kinase activity
- 19, 1, 0.08149, NADP or NADPH binding

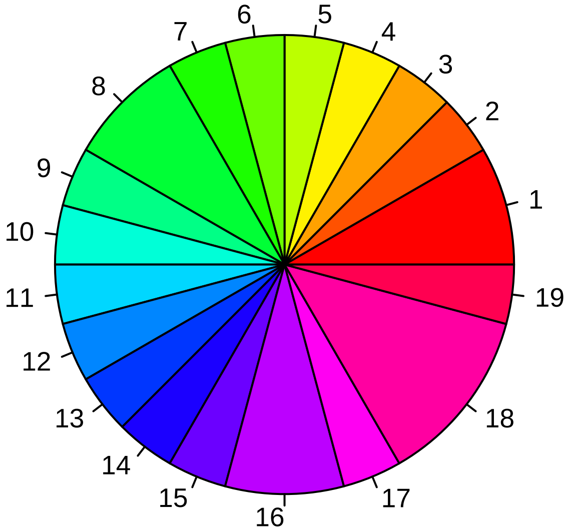

Supplement: Additional file 3: Figure S2 — Directed Acyclic Graph (DAG) and pie charts for Gene Ontology (GO) data for KO (A &B), KOd (C &D) and OE (D &F) gene datasets. The R packages GOstats, Rgraphviz and graphics were utilized to perform GO enrichment, generate the DAG plots and the pie plots. [file 1471-2164-13-623-S3.zip › Figure S2B.pdf]
