# Supplementary material for: High-resolution genome-wide scan of genes, gene-networks and cellular systems impacting the yeast ionome
Source: BMC Genomics. 2012 Nov 14;13:623. doi: 10.1186/1471-2164-13-623 (PMC3652779; doi:10.1186/1471-2164-13-623)

Directed Acyclic Graph of the 1 significant
GO terms of the 3 genes in KOd screen, Group C, CC

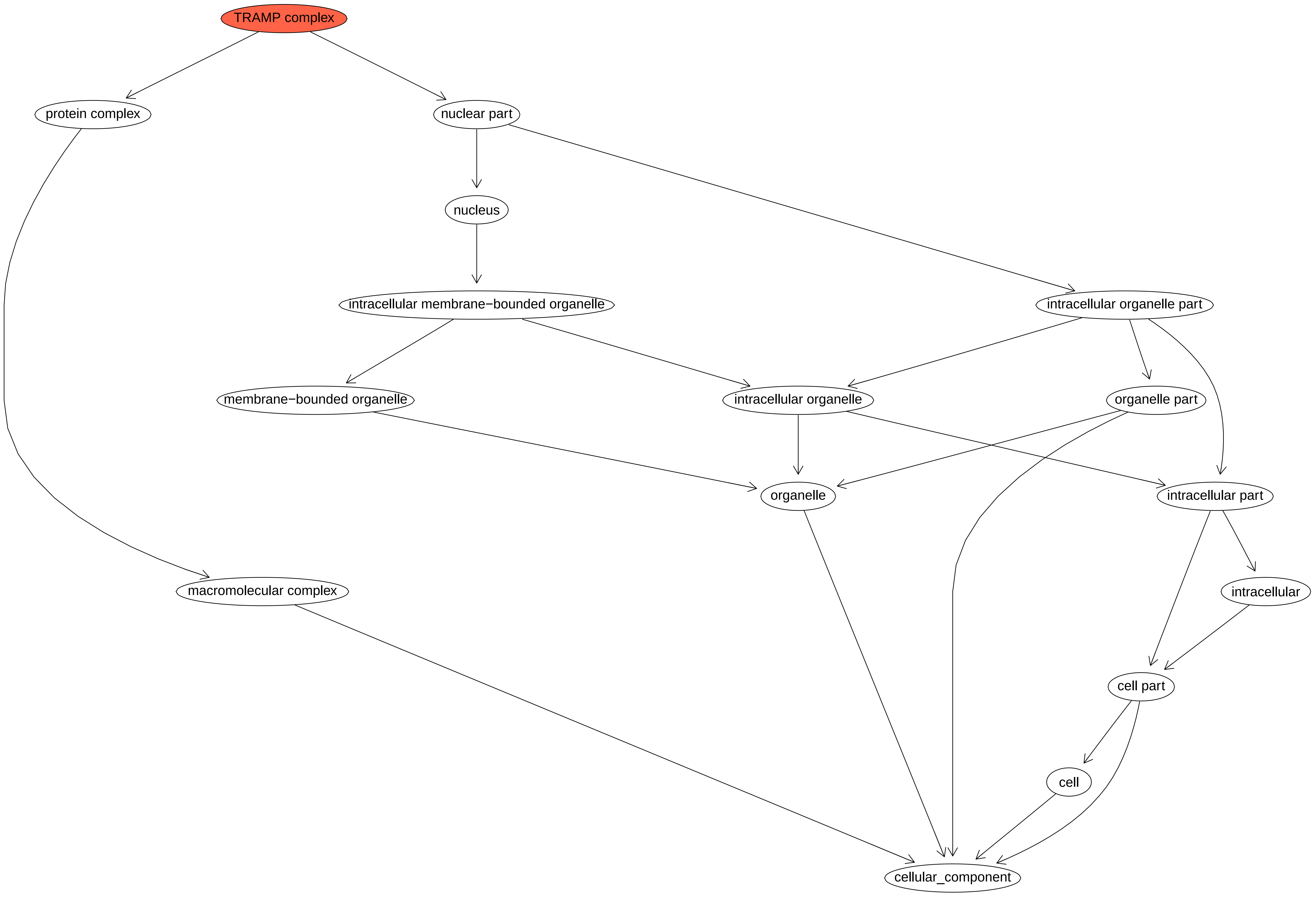

Directed Acyclic Graph of the 25 significant GO terms of the 3 genes in KOd screen, Group C, BP

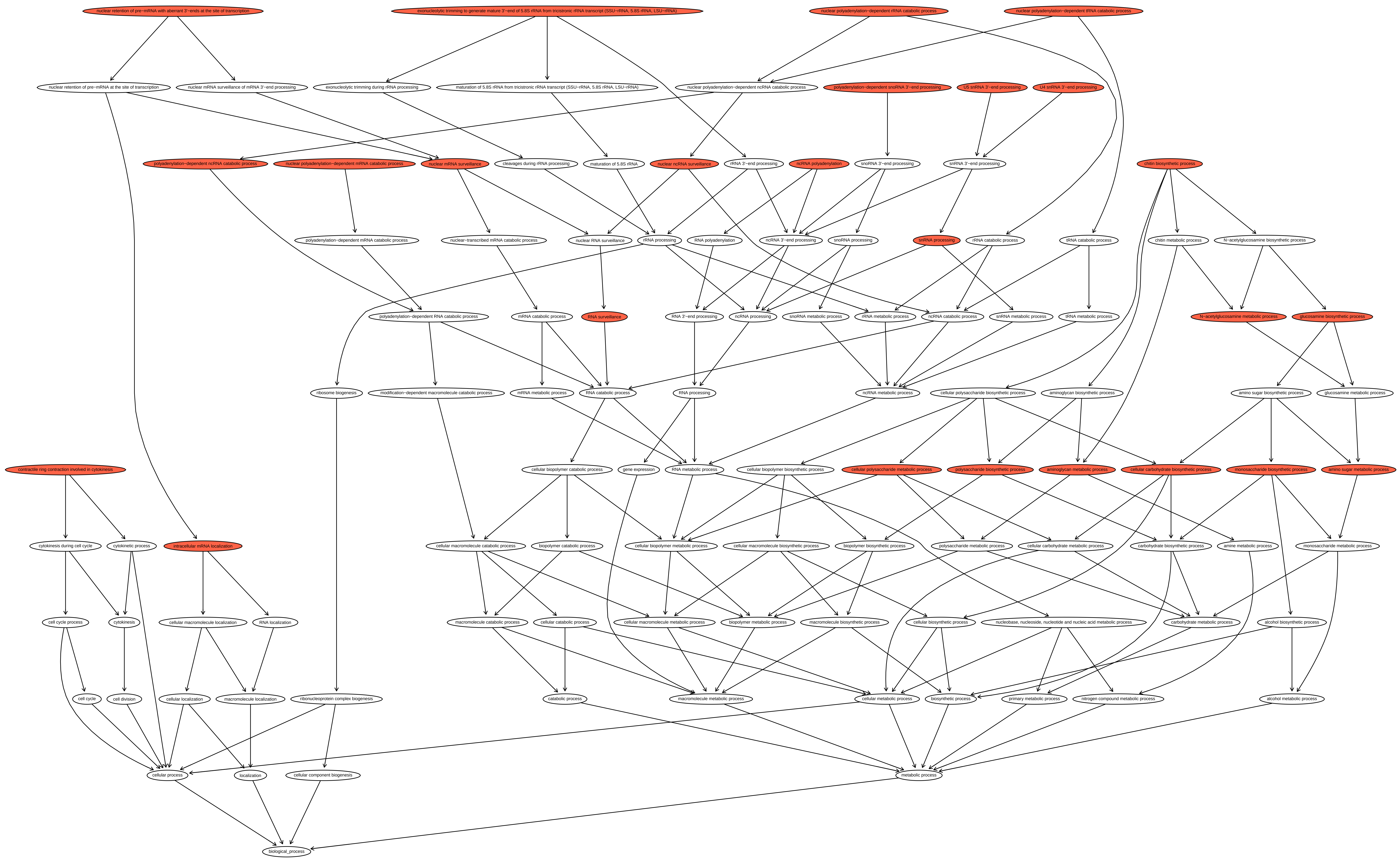

Directed Acyclic Graph of the 10 significant
GO terms of the 3 genes in KOd screen, Group C, MF

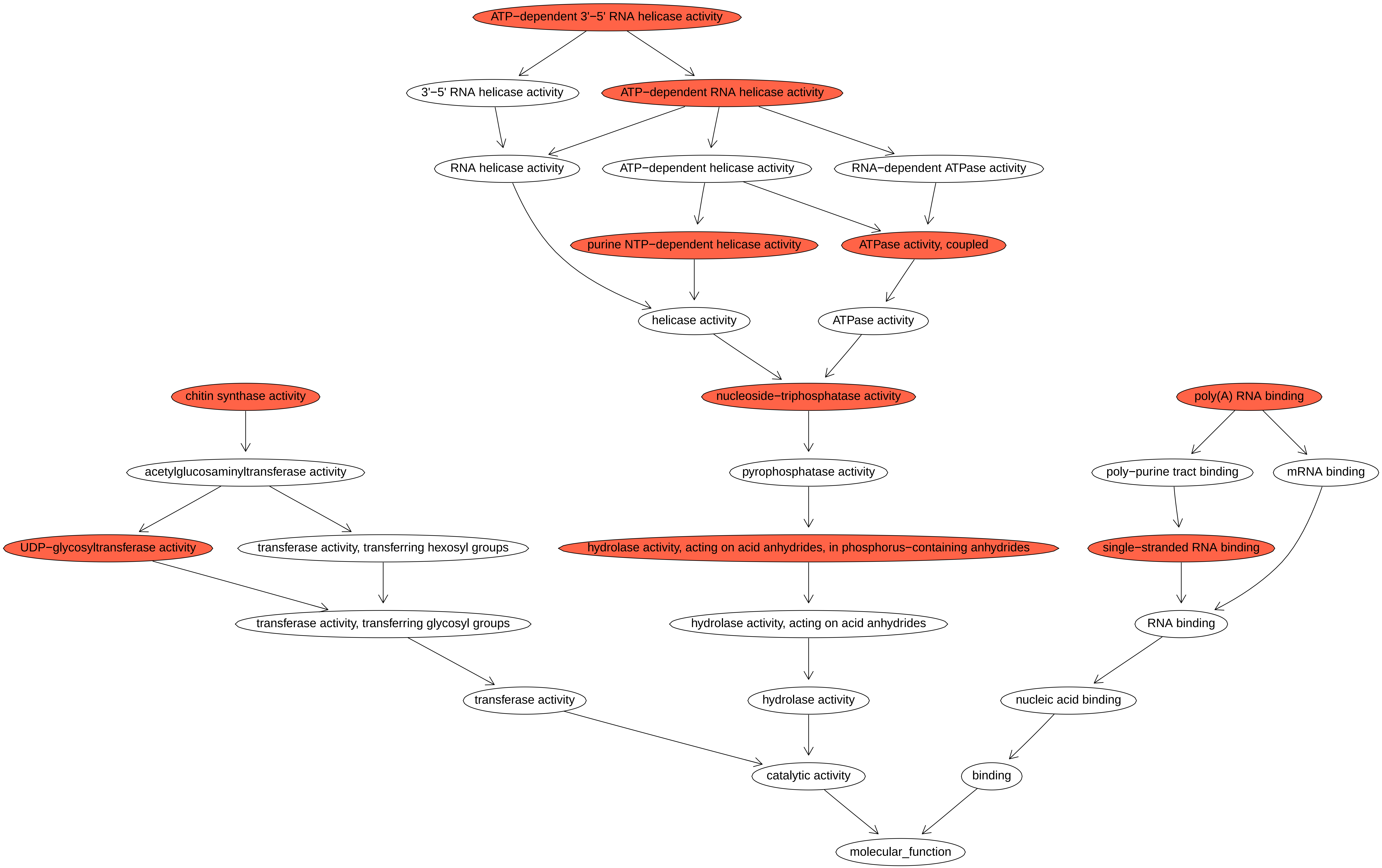

Directed Acyclic Graph of the 16 significant GO terms of the 25 genes in KOd screen, Group B, CC

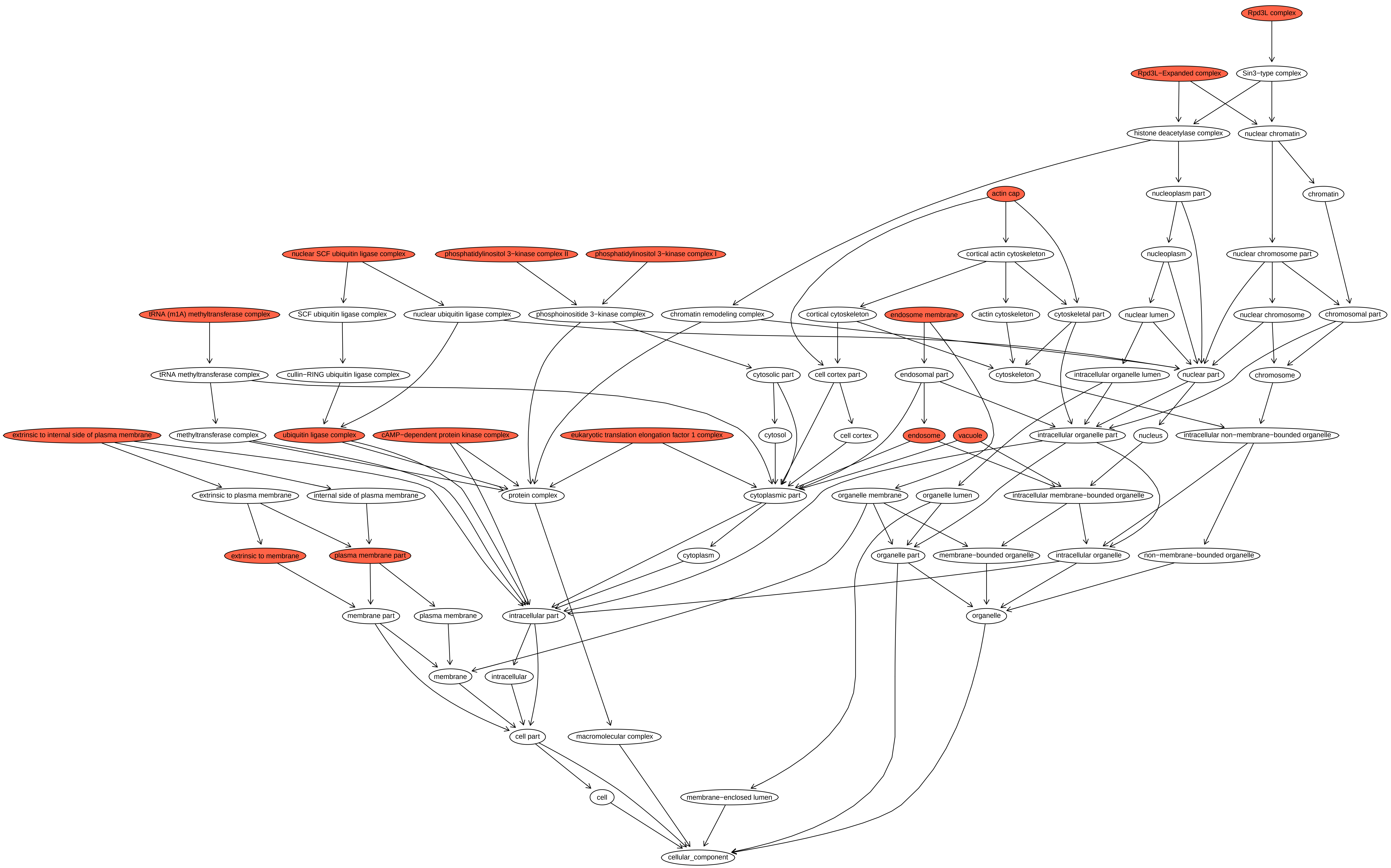

Directed Acyclic Graph of the 81 significant GO terms of the 25 genes in KOd screen, Group B, BP

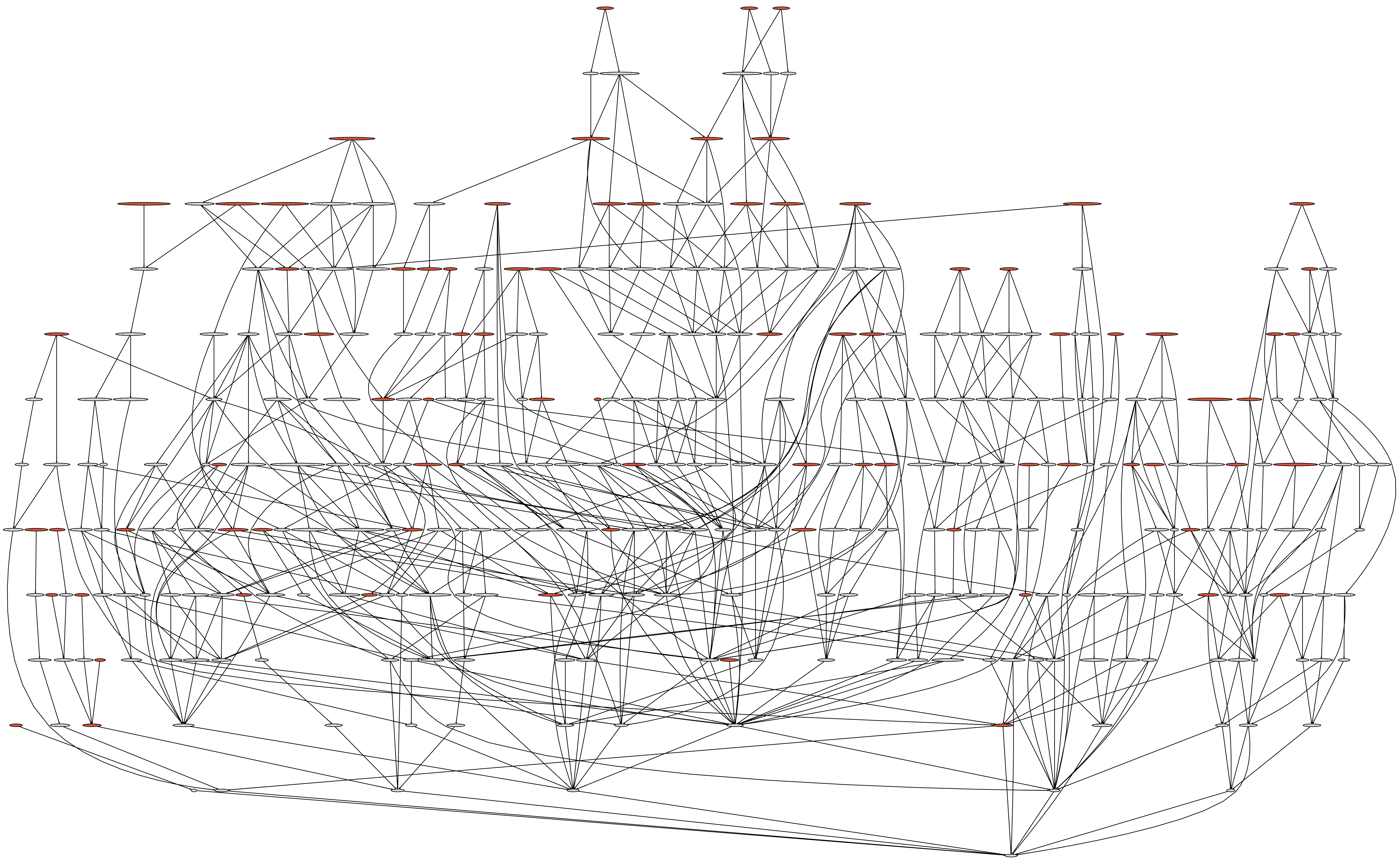

Directed Acyclic Graph of the 34 significant GO terms of the 25 genes in KOd screen, Group B, MF

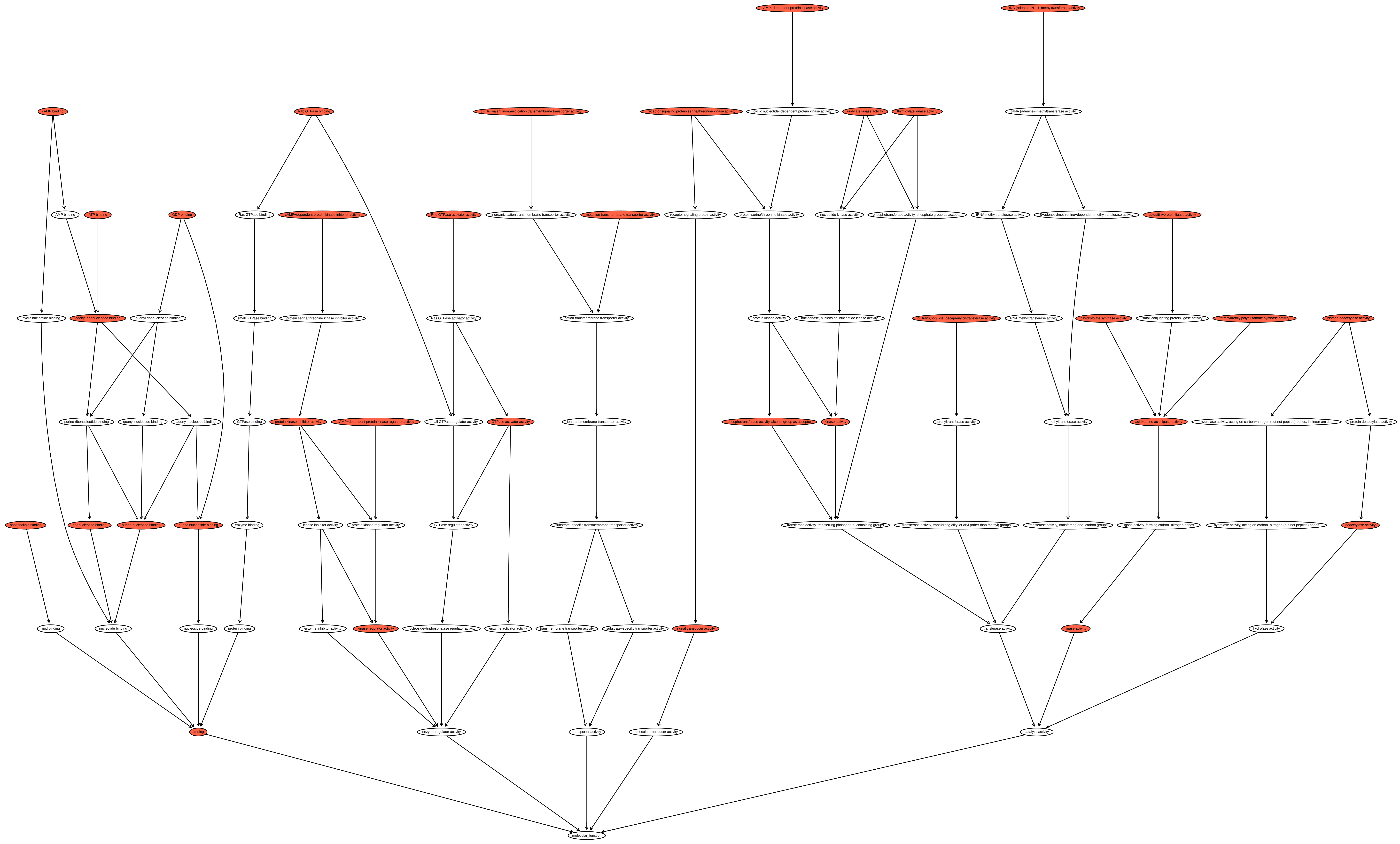

Directed Acyclic Graph of the 12 significant
GO terms of the 7 genes in KOd screen, Group A, CC

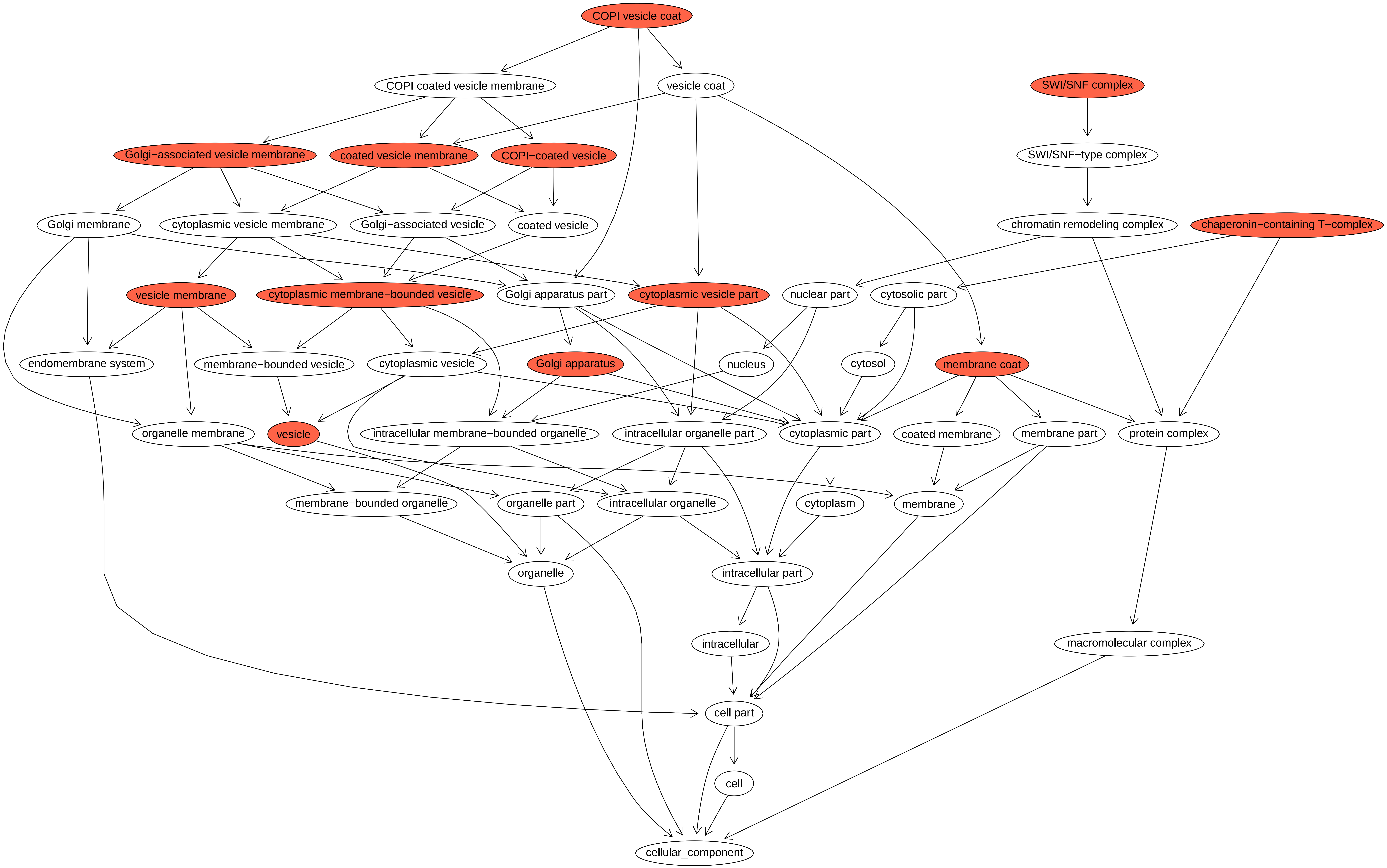

Directed Acyclic Graph of the 10 significant GO terms of the 7 genes in KOd screen, Group A, BP

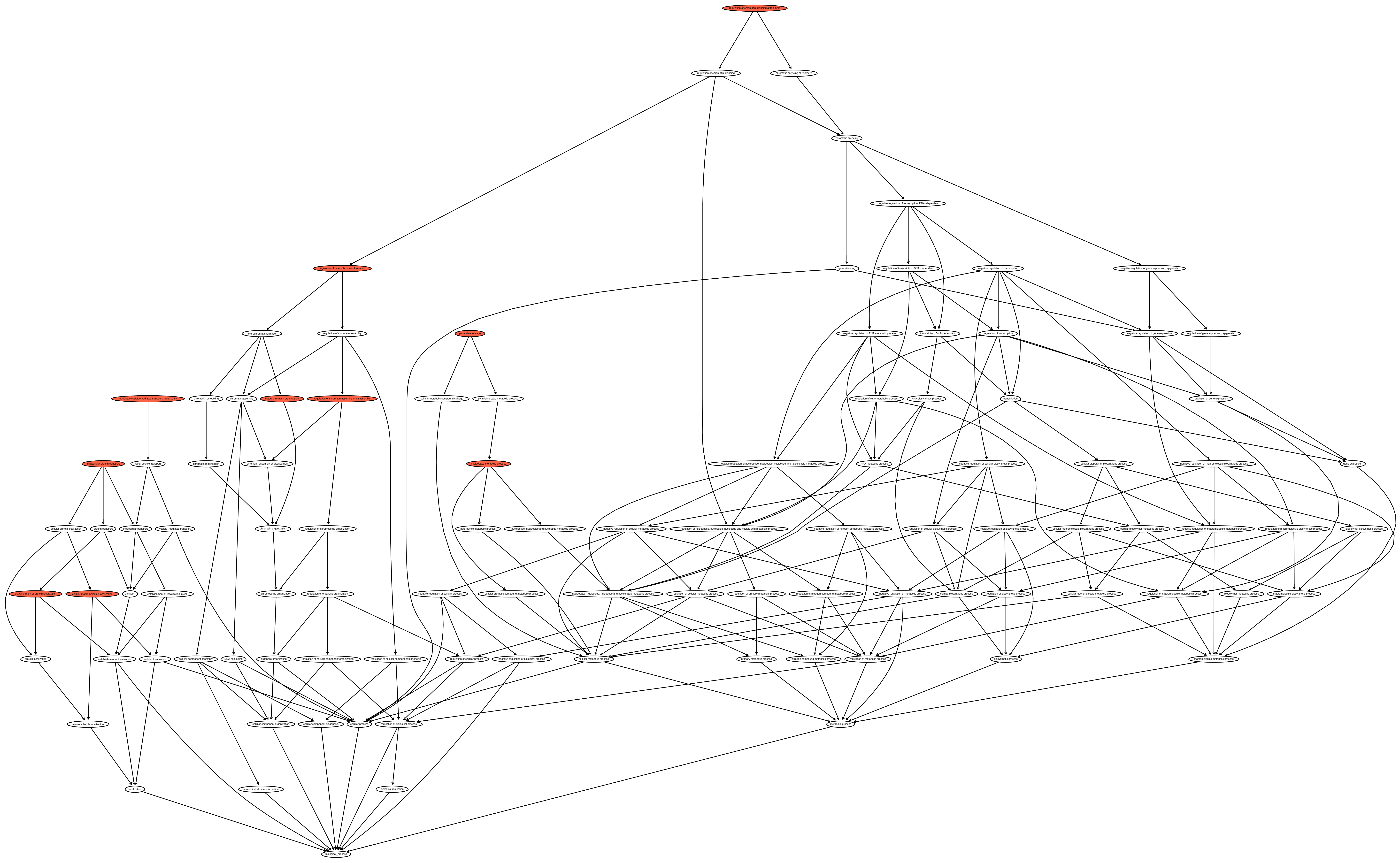

**Directed Acyclic Graph of the 1 significant
GO terms of the 7 genes in KOd screen, Group A, MF**

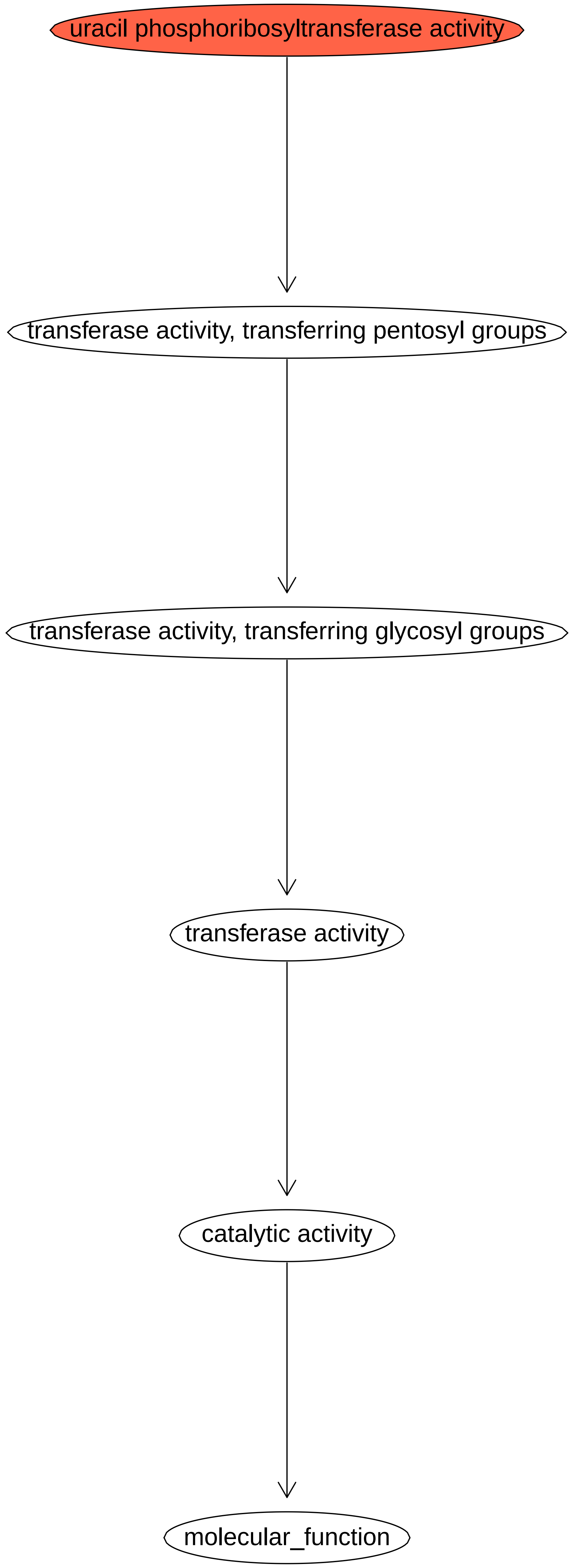

Supplement: Additional file 3: Figure S2 — Directed Acyclic Graph (DAG) and pie charts for Gene Ontology (GO) data for KO (A &B), KOd (C &D) and OE (D &F) gene datasets. The R packages GOstats, Rgraphviz and graphics were utilized to perform GO enrichment, generate the DAG plots and the pie plots. [file 1471-2164-13-623-S3.zip › Figure S2C.pdf]
