# Supplementary material for: High-resolution genome-wide scan of genes, gene-networks and cellular systems impacting the yeast ionome
Source: BMC Genomics. 2012 Nov 14;13:623. doi: 10.1186/1471-2164-13-623 (PMC3652779; doi:10.1186/1471-2164-13-623)

KOd group C 3 genes, CC
pieCharts ID, Counts, P−values and GO terms (left)

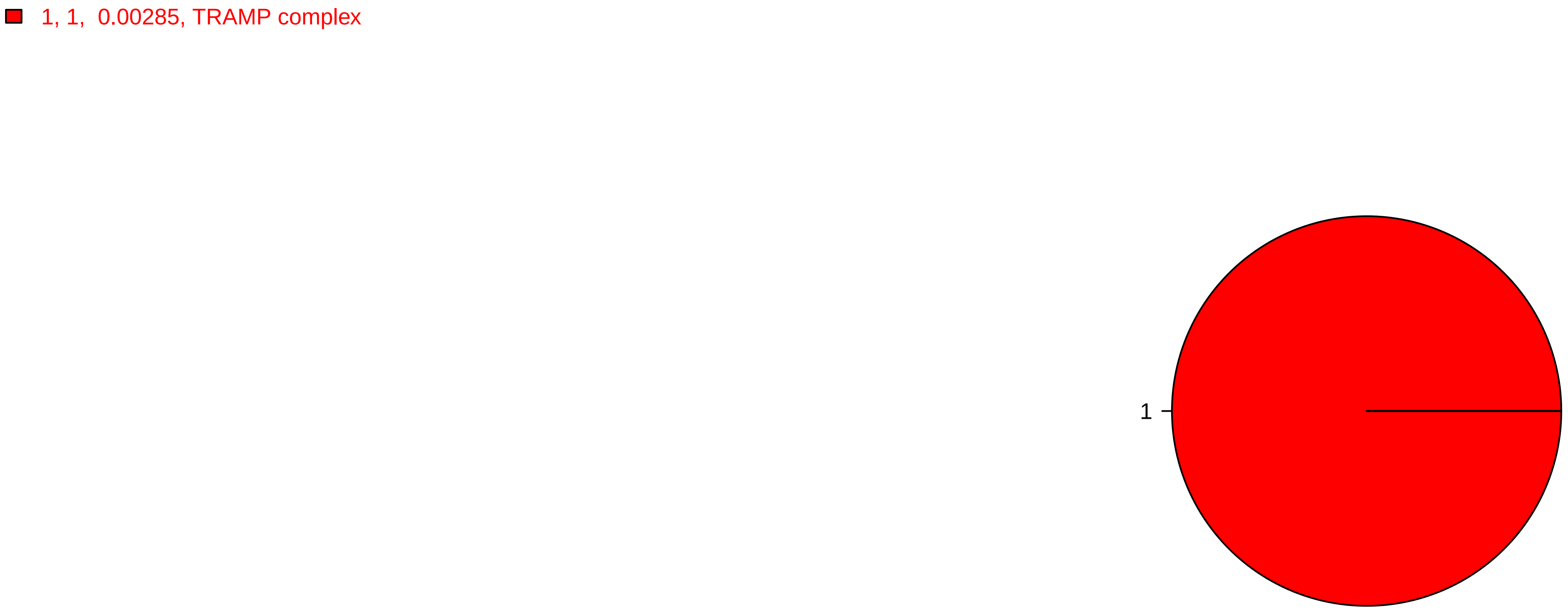

KOd group C 3 genes, BP
pieCharts ID, Counts, P−values and GO terms (left)

- 1, 1, 0.0057, intracellular mRNA localization
- 2, 1, 0.0057, ncRNA polyadenylation
- 3, 1, 0.0057, nuclear retention of pre−mRNA with aberrant 3'−ends at the site of transcription
- 4, 1, 0.00855, contractile ring contraction involved in cytokinesis
- 5, 1, 0.00855, aminoglycan metabolic process
- 6, 1, 0.00855, chitin biosynthetic process
- 7, 1, 0.00855, nuclear polyadenylation−dependent mRNA catabolic process
- 8, 1, 0.01139, U5 snRNA 3'−end processing
- 9, 1, 0.01139, polyadenylation−dependent snoRNA 3'−end processing
- 10, 1, 0.01422, amino sugar metabolic process
- 11, 1, 0.01422, glucosamine biosynthetic process
- 12, 1, 0.01422, N−acetylglucosamine metabolic process
- 13, 1, 0.01705, polysaccharide biosynthetic process
- 14, 1, 0.01705, nuclear mRNA surveillance
- 15, 1, 0.02268, U4 snRNA 3'−end processing
- 16, 1, 0.02268, cellular carbohydrate biosynthetic process
- 17, 1, 0.02549, snRNA processing
- 18, 1, 0.02549, cellular polysaccharide metabolic process
- 19, 1, 0.02549, nuclear polyadenylation−dependent rRNA catabolic process
- 20, 1, 0.0311, polyadenylation−dependent ncRNA catabolic process
- 21, 1, 0.0311, nuclear ncRNA surveillance
- 22, 1, 0.0311, nuclear polyadenylation−dependent tRNA catabolic process
- 23, 1, 0.0339, exonucleolytic trimming to generate mature 3'−end of 5.8S rRNA from tricistronic rRNA transcript (SSU−rRNA, 5.8S rRNA, LSU−rRNA)
- 24, 1, 0.0339, RNA surveillance
- 25, 1, 0.03947, monosaccharide biosynthetic process

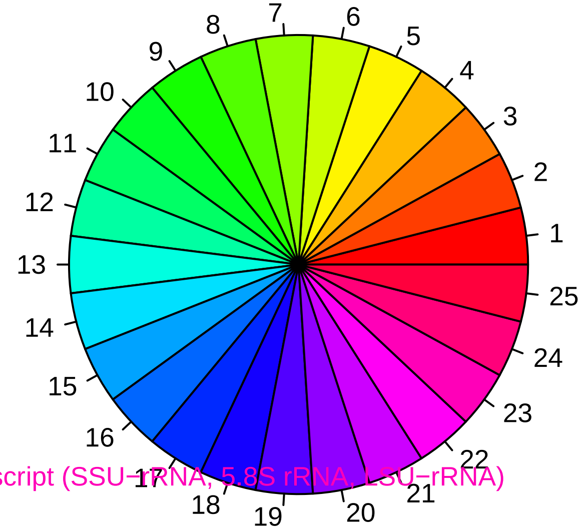

KOd group C 3 genes, MF
pieCharts ID, Counts, P−values and GO terms (left)

1, 1, 0.00285, chitin synthase activity
2, 1, 0.00285, ATP−dependent 3'−5' RNA helicase activity
3, 2, 0.00373, purine NTP−dependent helicase activity
4, 2, 0.00927, ATPase activity, coupled
5, 1, 0.01139, poly(A) RNA binding
6, 1, 0.01705, single−stranded RNA binding
7, 1, 0.02549, UDP−glycosyltransferase activity
8, 2, 0.03308, nucleoside−triphosphatase activity
9, 2, 0.03533, hydrolase activity, acting on acid anhydrides, in phosphorus−containing anhydrides
10, 1, 0.03962, ATP−dependent RNA helicase activity

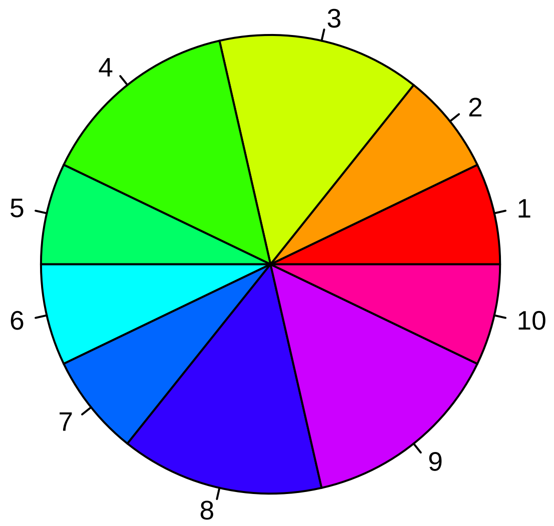

KOd group B 25 genes, CC
pieCharts ID, Counts, P−values and GO terms (left)

- 1, 2, 0.00054, endosome membrane
- 2, 2, 0.00161, extrinsic to internal side of plasma membrane
- 3, 3, 0.00227, endosome
- 4, 3, 0.01038, ubiquitin ligase complex
- 5, 2, 0.01766, plasma membrane part
- 6, 1, 0.02379, cAMP−dependent protein kinase complex
- 7, 1, 0.02379, Rpd3L complex
- 8, 1, 0.02379, phosphatidylinositol 3−kinase complex I
- 9, 1, 0.02379, phosphatidylinositol 3−kinase complex II
- 10, 1, 0.02379, Rpd3L−Expanded complex
- 11, 2, 0.02621, vacuole
- 12, 2, 0.0361, extrinsic to membrane
- 13, 1, 0.04703, eukaryotic translation elongation factor 1 complex
- 14, 1, 0.04703, actin cap
- 15, 1, 0.04703, tRNA (m1A) methyltransferase complex
- 16, 1, 0.04703, nuclear SCF ubiquitin ligase complex
- 17, 1, 0.06974, monolayer−surrounded lipid storage body
- 18, 1, 0.06974, TORC1 complex
- 19, 2, 0.08074, nuclear chromatin
- 20, 2, 0.08658, Golgi membrane
- 21, 1, 0.09193, cohesin complex
- 22, 1, 0.09193, TORC2 complex
- 23, 1, 0.09193, nuclear mitotic cohesin complex
- 24, 1, 0.09193, RNA polymerase I core factor complex

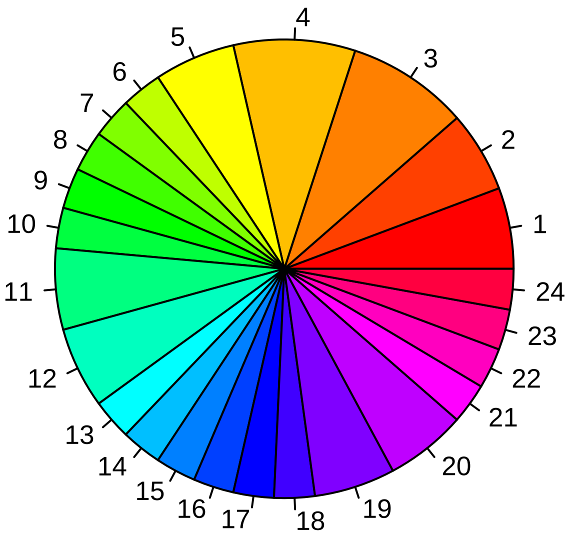

KOd group B 25 genes, BP
pieCharts ID, Counts, P−values and GO terms (left)

- 1, 11, 0.00141, biopolymer modification
- 2, 2, 0.00317, positive regulation of specific transcription from RNA polymerase II promoter
- 3, 2, 0.00317, regulation of gene−specific transcription
- 4, 3, 0.00365, one−carbon metabolic process
- 5, 2, 0.0052, regulation of phosphate metabolic process
- 6, 5, 0.00645, post−translational protein modification
- 7, 2, 0.01061, second−messenger−mediated signaling
- 8, 2, 0.01061, filamentous growth
- 9, 5, 0.01378, signal transduction
- 10, 4, 0.01392, phosphorus metabolic process
- 11, 2, 0.01394, protein ubiquitination during ubiquitin−dependent protein catabolic process
- 12, 11, 0.01445, cellular protein metabolic process
- 13, 1, 0.02379, regulation of transcription during G1 phase of mitotic cell cycle
- 14, 1, 0.02379, regulation of protein amino acid phosphorylation
- 15, 1, 0.02379, dUDP biosynthetic process
- 16, 1, 0.02379, dTDP biosynthetic process
- 17, 1, 0.02379, dTTP biosynthetic process
- 18, 1, 0.02379, ubiquinone metabolic process
- 19, 1, 0.02379, regulation of nitrogen utilization
- 20, 1, 0.02379, pyrimidine nucleoside diphosphate biosynthetic process
- 21, 1, 0.02379, pyrimidine nucleoside triphosphate biosynthetic process
- 22, 1, 0.02379, deoxyribonucleoside diphosphate biosynthetic process
- 23, 1, 0.02379, pyrimidine deoxyribonucleoside diphosphate metabolic process
- 24, 1, 0.02379, response to light stimulus
- 25, 1, 0.02379, regulation of mRNA export from nucleus
- 26, 1, 0.02379, regulation of dolichol biosynthetic process
- 27, 1, 0.02379, regulation of ubiquinone biosynthetic process
- 28, 1, 0.02379, regulation of multivesicular body size
- 29, 1, 0.02379, magnesium ion transport
- 30, 1, 0.02379, macroautophagy

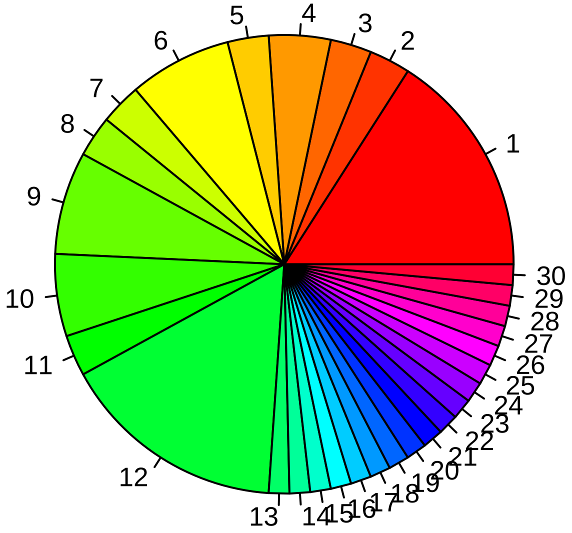

KOd group B 25 genes, MF
pieCharts ID, Counts, P−values and GO terms (left)

1, 4, 0.01277, kinase activity
2, 2, 0.01394, kinase regulator activity
3, 3, 0.01539, ubiquitin−protein ligase activity
4, 9, 0.01563, purine nucleoside binding
5, 10, 0.01715, ribonucleotide binding
6, 3, 0.01735, acid−amino acid ligase activity
7, 1, 0.0195, adenyl ribonucleotide binding
8, 10, 0.02079, purine nucleotide binding
9, 1, 0.02379, tetrahydrofolylpolyglutamate synthase activity
10, 1, 0.02379, histone deacetylase activity
11, 1, 0.02379, cAMP−dependent protein kinase activity
12, 1, 0.02379, thymidylate kinase activity
13, 1, 0.02379, protein kinase inhibitor activity
14, 1, 0.02379, cAMP−dependent protein kinase inhibitor activity
15, 1, 0.02379, Rho GTPase activator activity
16, 1, 0.02379, cAMP−dependent protein kinase regulator activity
17, 1, 0.02379, di−trans,poly−cis−decaprenylcistransferase activity
18, 1, 0.02379, dihydrofolate synthase activity
19, 1, 0.02379, di−, tri−valent inorganic cation transmembrane transporter activity
20, 1, 0.02379, GDP binding
21, 1, 0.02379, cAMP binding
22, 1, 0.02379, metal ion transmembrane transporter activity
23, 2, 0.02621, phospholipid binding
24, 2, 0.031, GTPase activator activity
25, 21, 0.0332, binding
26, 3, 0.03444, phosphotransferase activity, alcohol group as acceptor
27, 2, 0.0361, signal transducer activity
28, 1, 0.04703, receptor signaling protein serine/threonine kinase activity
29, 1, 0.04703, uridylate kinase activity
30, 1, 0.04703, tRNA (adenine−N1−)−methyltransferase activity

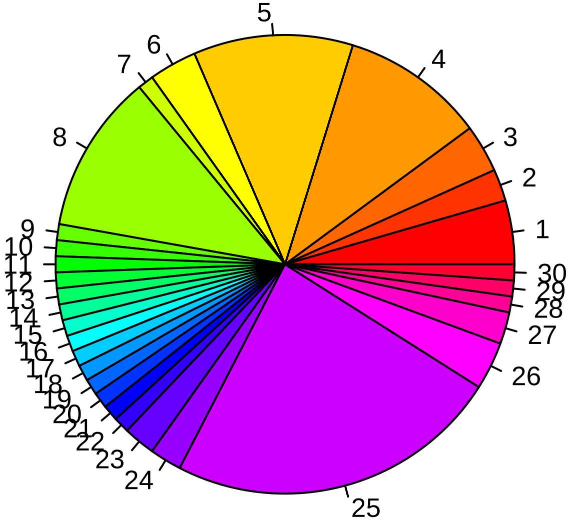

KOd group A 7 genes, CC
pieCharts ID, Counts, P−values and GO terms (left)

- 1, 2, 4e−04, COPI vesicle coat
- 2, 2, 0.00056, COPI−coated vesicle
- 3, 2, 0.00175, vesicle membrane
- 4, 2, 0.00175, Golgi−associated vesicle membrane
- 5, 2, 0.00175, coated vesicle membrane
- 6, 2, 0.00175, cytoplasmic vesicle part
- 7, 2, 0.00206, membrane coat
- 8, 2, 0.00597, cytoplasmic membrane−bounded vesicle
- 9, 2, 0.00769, vesicle
- 10, 1, 0.02267, SWI/SNF complex
- 11, 2, 0.02944, Golgi apparatus
- 12, 1, 0.04492, chaperonin−containing T−complex
- 13, 1, 0.06132, RSC complex

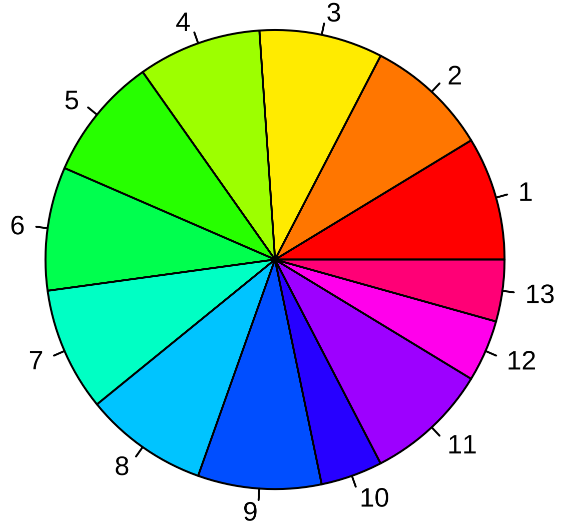

KOd group A 7 genes, BP
pieCharts ID, Counts, P−values and GO terms (left)

- 1, 2, 0.00315, retrograde vesicle−mediated transport, Golgi to ER
- 2, 1, 0.00571, pyrimidine salvage
- 3, 1, 0.01139, nucleobase metabolic process
- 4, 3, 0.01241, intracellular protein transport
- 5, 3, 0.01591, cellular macromolecule localization
- 6, 1, 0.03939, regulation of chromatin silencing at telomere
- 7, 3, 0.0396, establishment of protein localization
- 8, 1, 0.04492, regulation of chromatin assembly or disassembly
- 9, 1, 0.04492, regulation of heterochromatin formation
- 10, 1, 0.04492, heterochromatin organization
- 11, 1, 0.05588, nucleosome disassembly
- 12, 1, 0.06132, ATP−dependent chromatin remodeling
- 13, 1, 0.06577, chromatin assembly or disassembly
- 14, 1, 0.06673, exonucleolytic trimming to generate mature 3'−end of 5.8S rRNA from tricistronic rRNA transcript (SSU−rRNA, 5.8S rRNA, LSU−rRNA)
- 15, 2, 0.07023, vesicle−mediated transport
- 16, 3, 0.07111, establishment of localization in cell
- 17, 1, 0.07212, chromatin assembly
- 18, 1, 0.08375, chromatin remodeling
- 19, 1, 0.09342, regulation of cellular component biogenesis

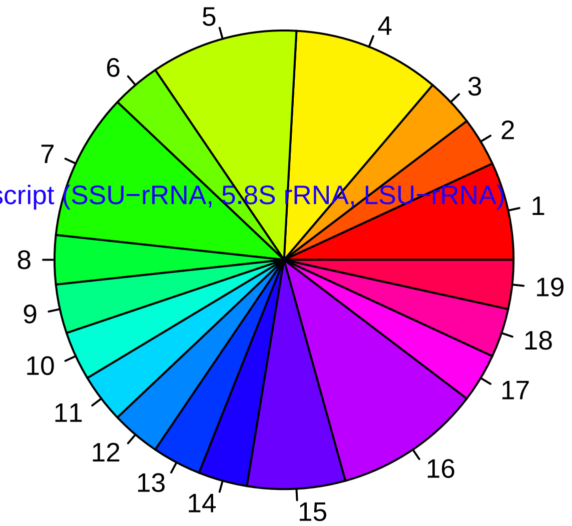

KOd group A 7 genes, MF
pieCharts ID, Counts, P−values and GO terms (left)

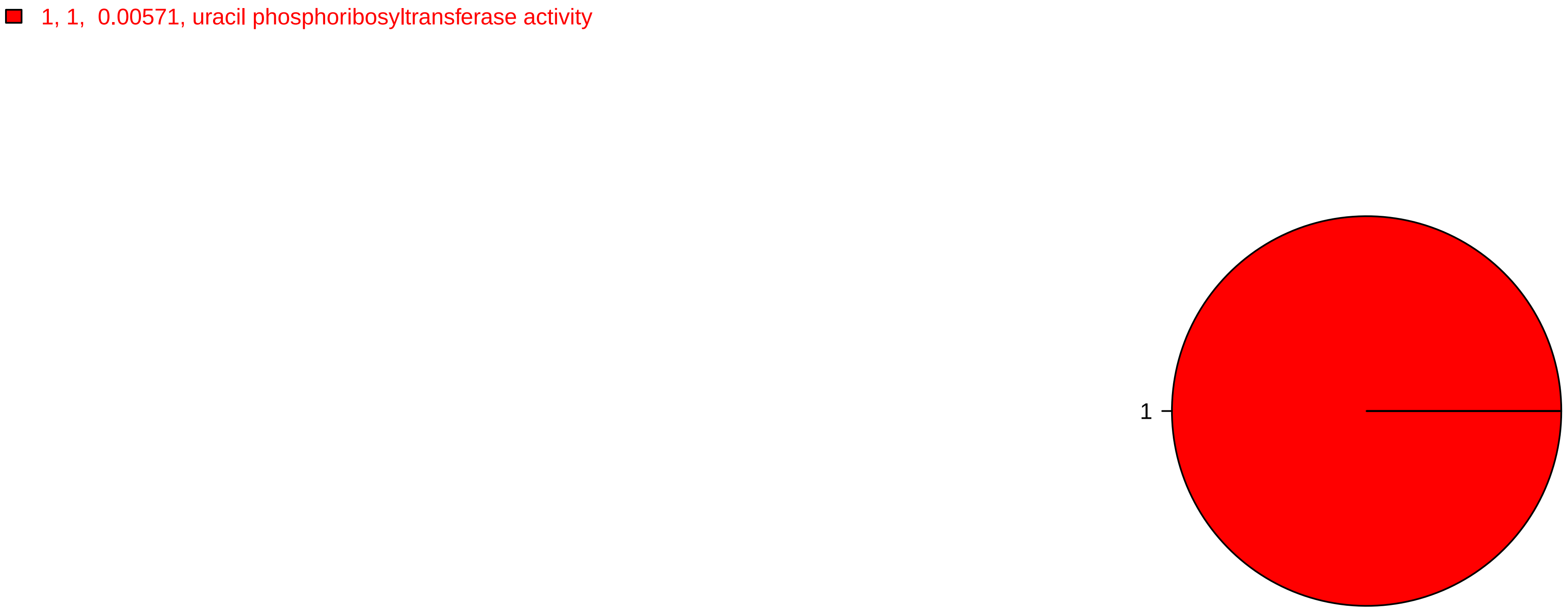

Supplement: Additional file 3: Figure S2 — Directed Acyclic Graph (DAG) and pie charts for Gene Ontology (GO) data for KO (A &B), KOd (C &D) and OE (D &F) gene datasets. The R packages GOstats, Rgraphviz and graphics were utilized to perform GO enrichment, generate the DAG plots and the pie plots. [file 1471-2164-13-623-S3.zip › Figure S2D.pdf]
