# Supplementary material for: High-resolution genome-wide scan of genes, gene-networks and cellular systems impacting the yeast ionome
Source: BMC Genomics. 2012 Nov 14;13:623. doi: 10.1186/1471-2164-13-623 (PMC3652779; doi:10.1186/1471-2164-13-623)

Directed Acyclic Graph of the 20 significant GO terms of the 36 genes in OE screen, Group C, CC

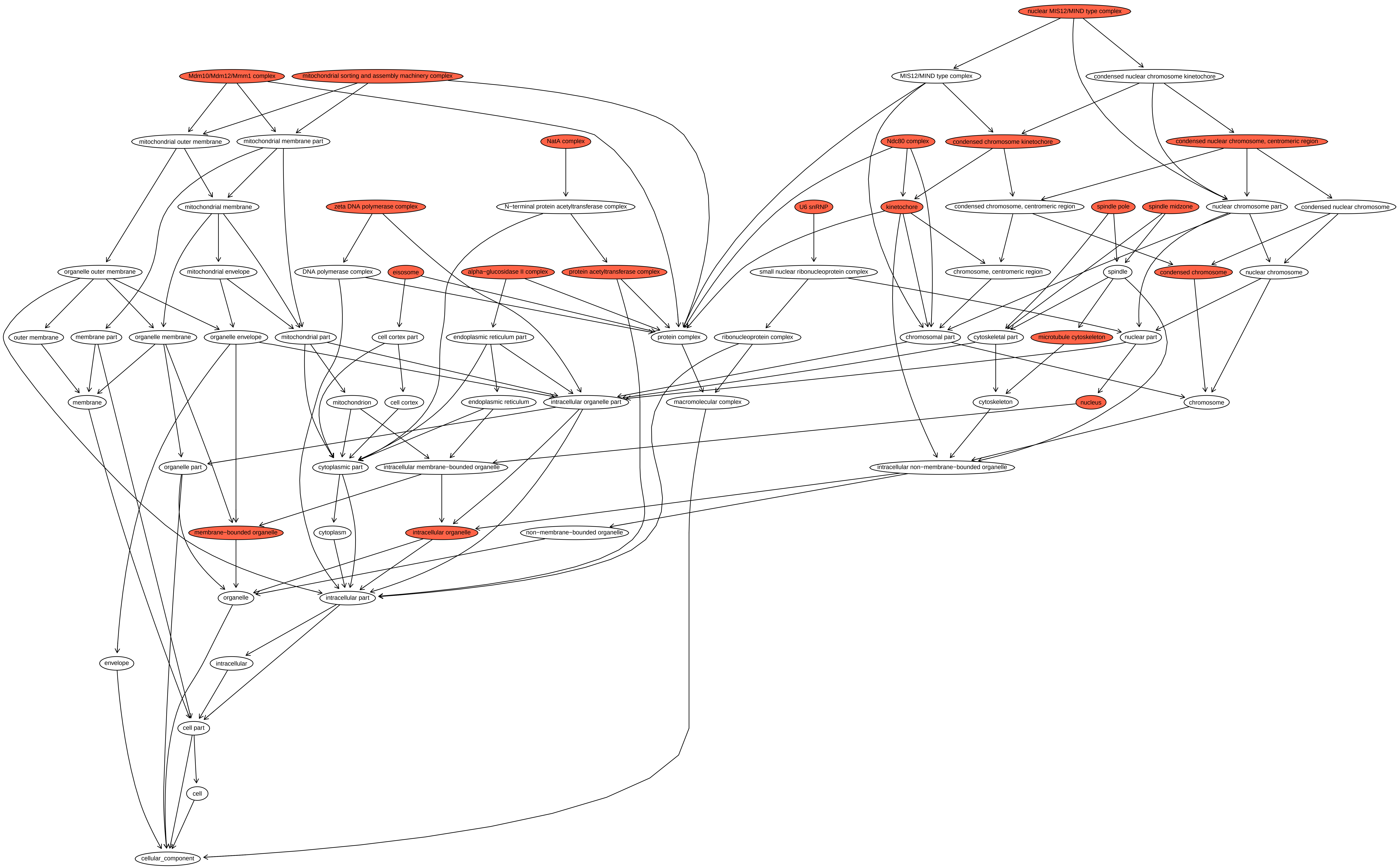

Directed Acyclic Graph of the 48 significant
GO terms of the 36 genes in OE screen, Group C, BP

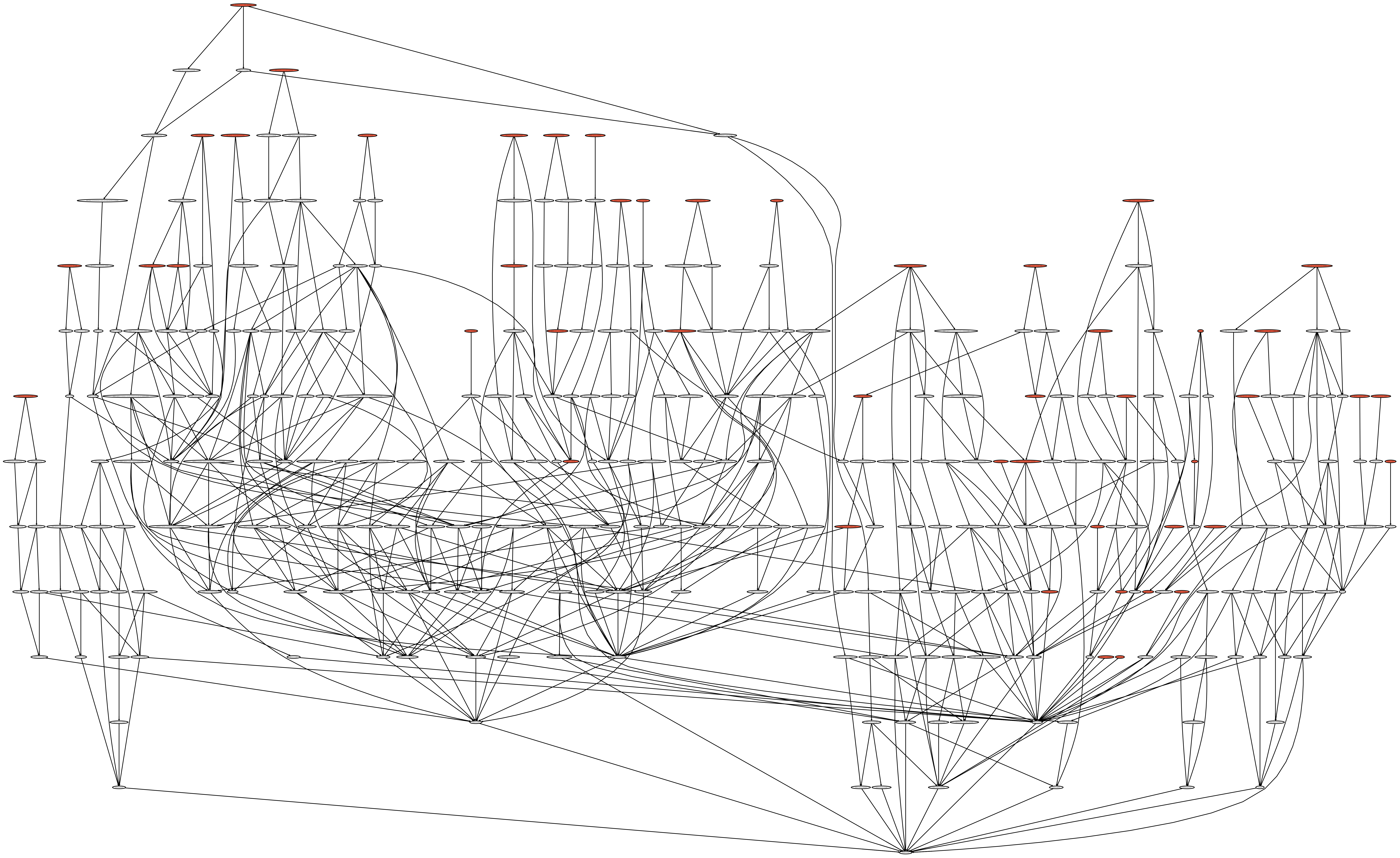

Directed Acyclic Graph of the 20 significant GO terms of the 36 genes in OE screen, Group C, MF

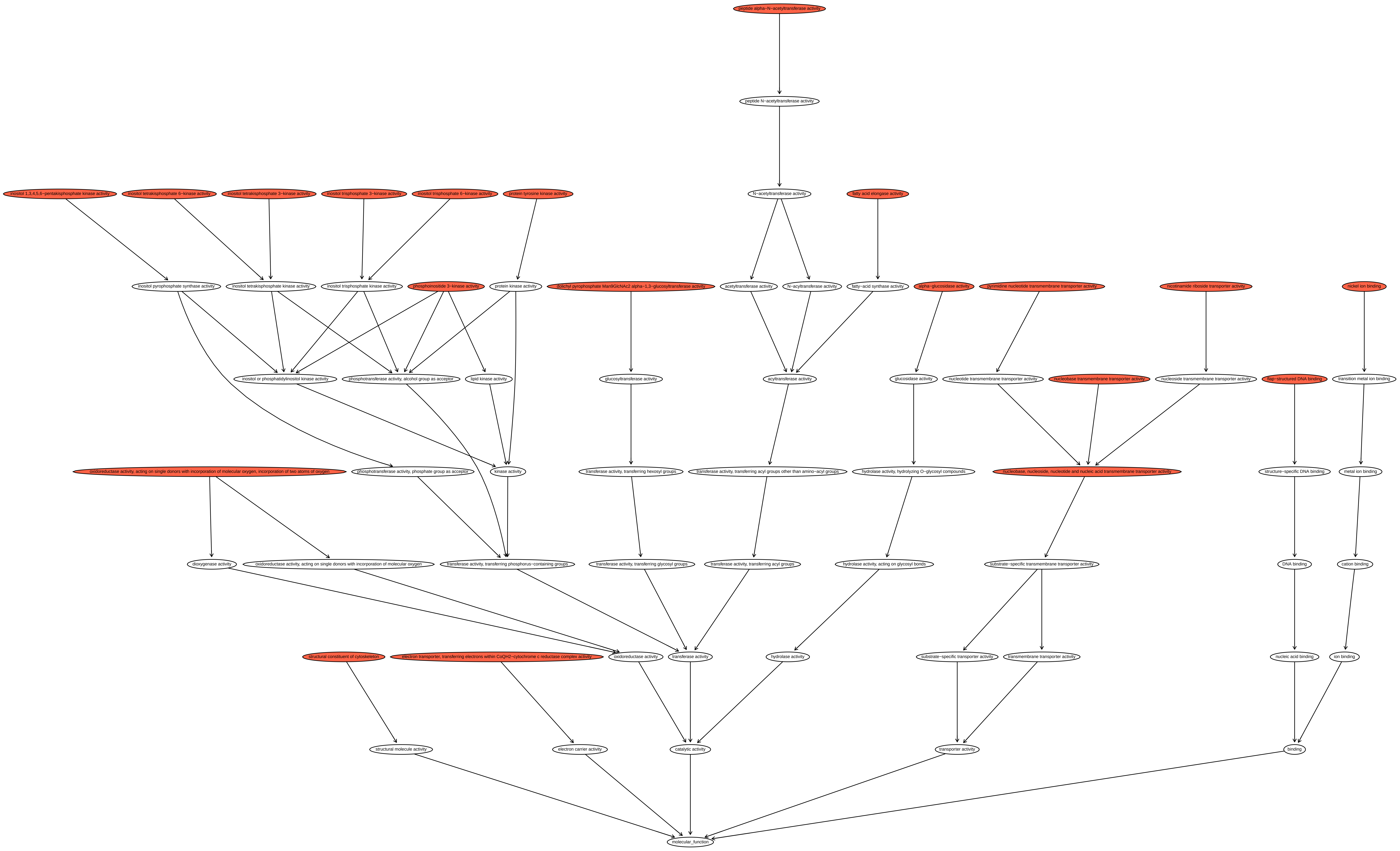

Directed Acyclic Graph of the 13 significant GO terms of the 330 genes in OE screen, Group B, CC

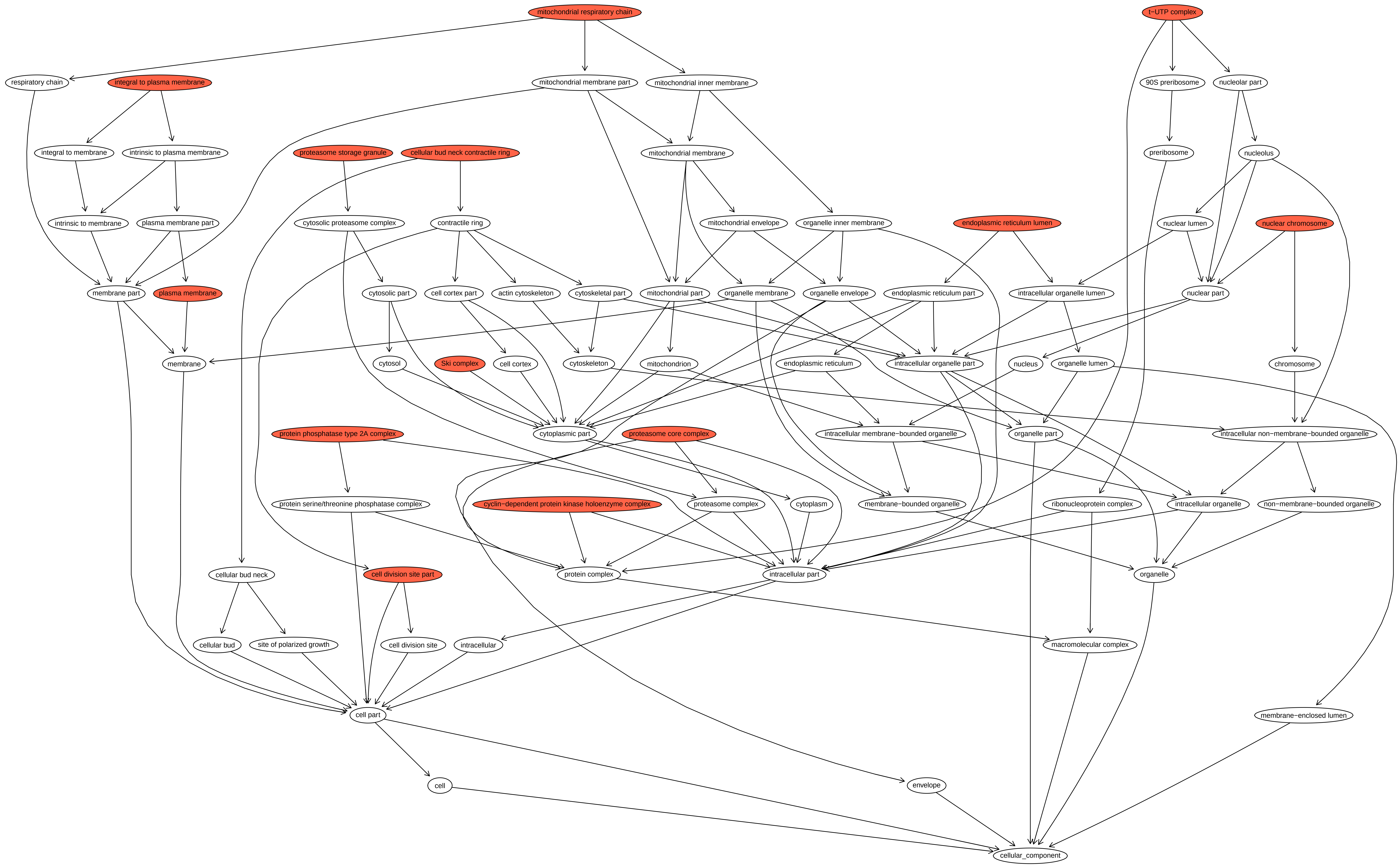

Directed Acyclic Graph of the 46 significant
GO terms of the 330 genes in OE screen, Group B, BP

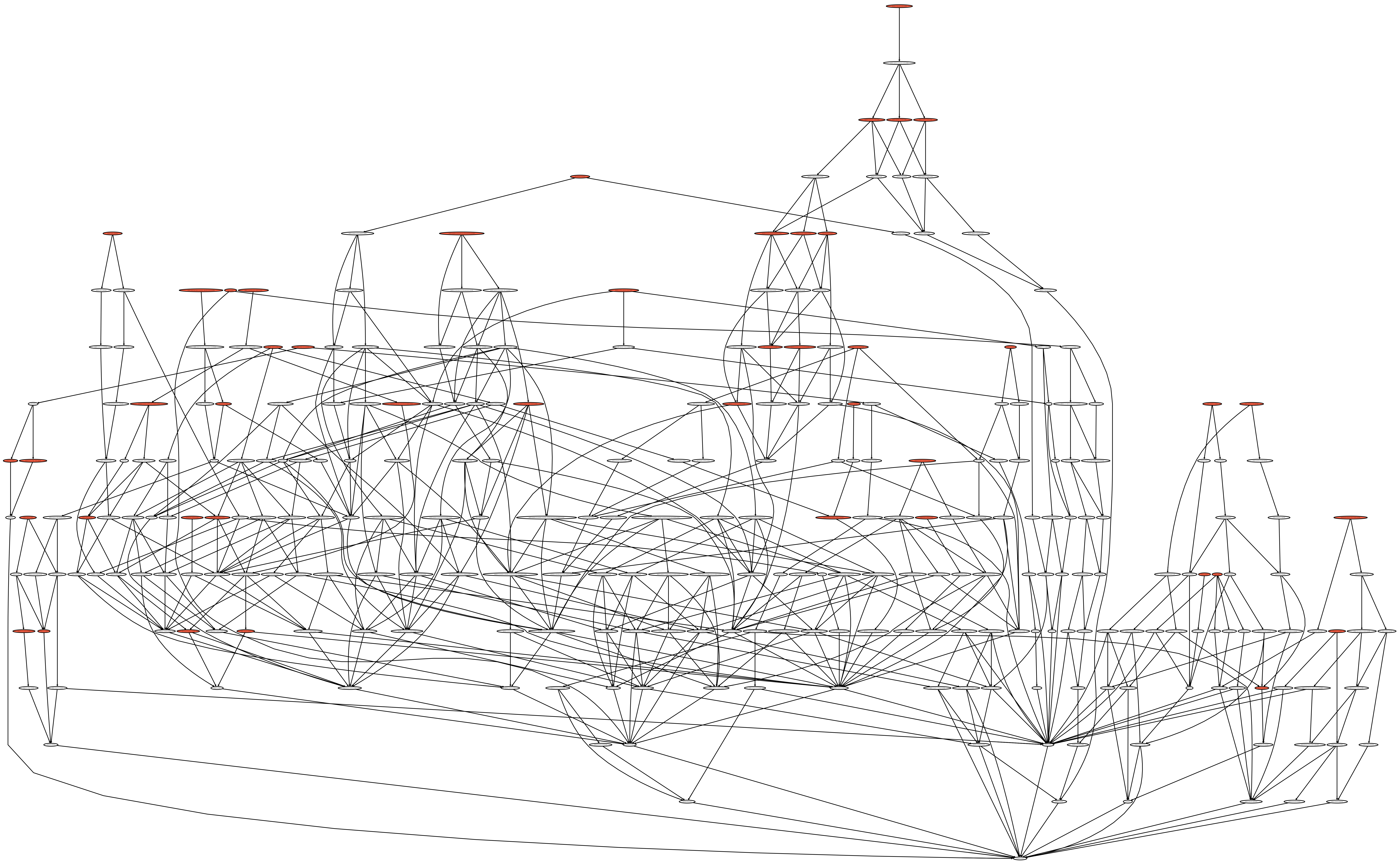

Directed Acyclic Graph of the 14 significant GO terms of the 330 genes in OE screen, Group B, MF

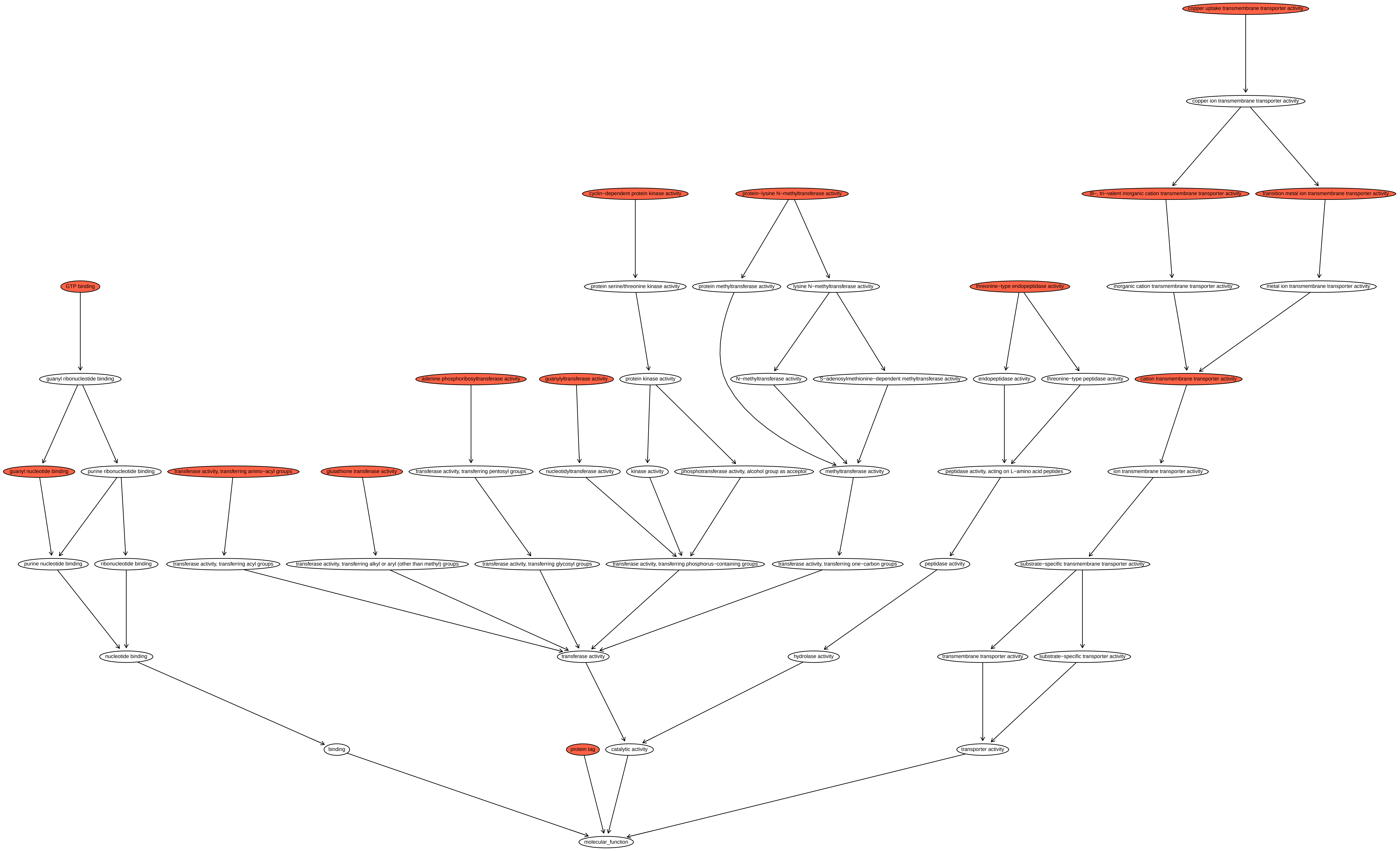

Directed Acyclic Graph of the 12 significant
GO terms of the 80 genes in OE screen, Group A, CC

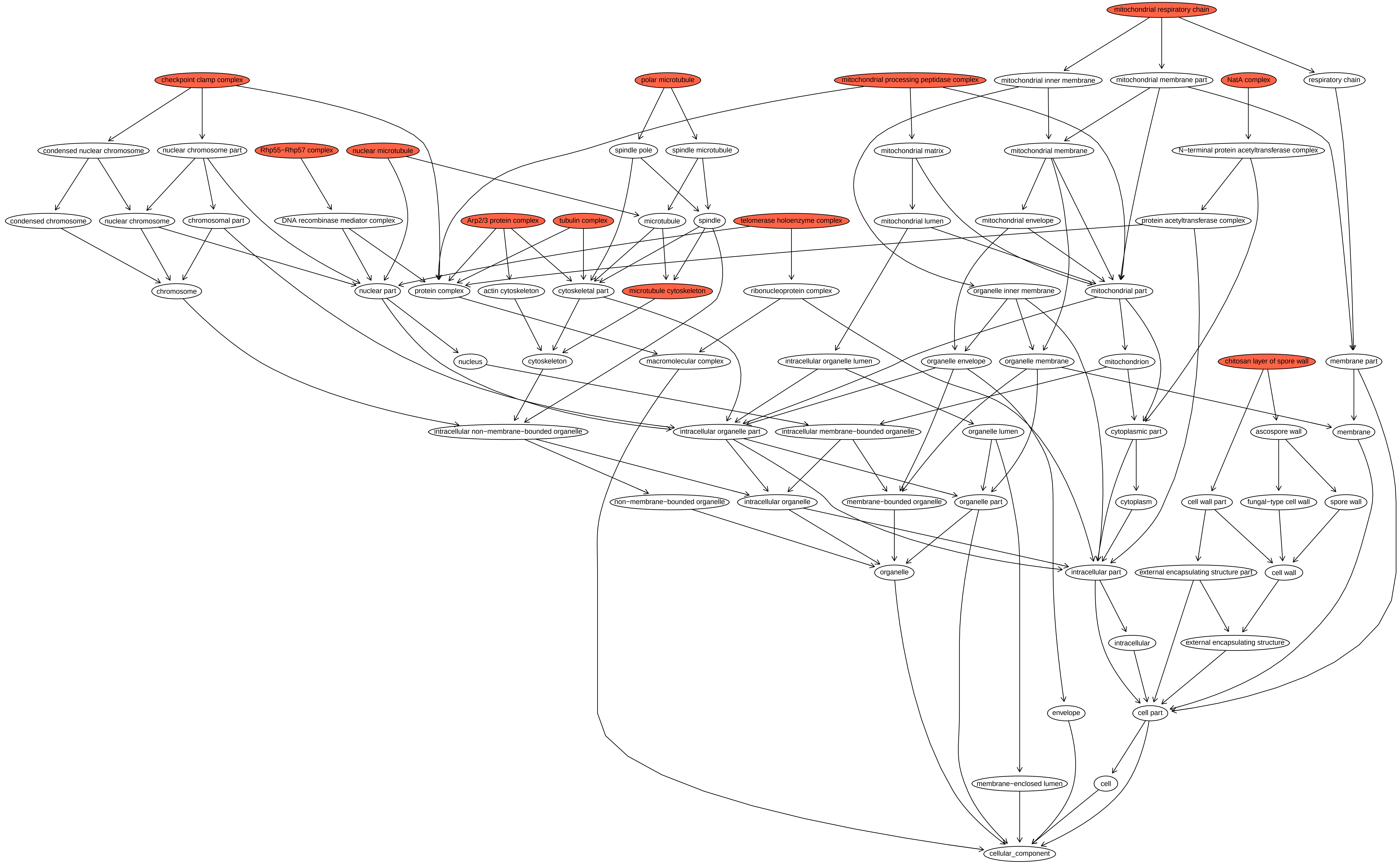

Directed Acyclic Graph of the 34 significant GO terms of the 80 genes in OE screen, Group A, BP

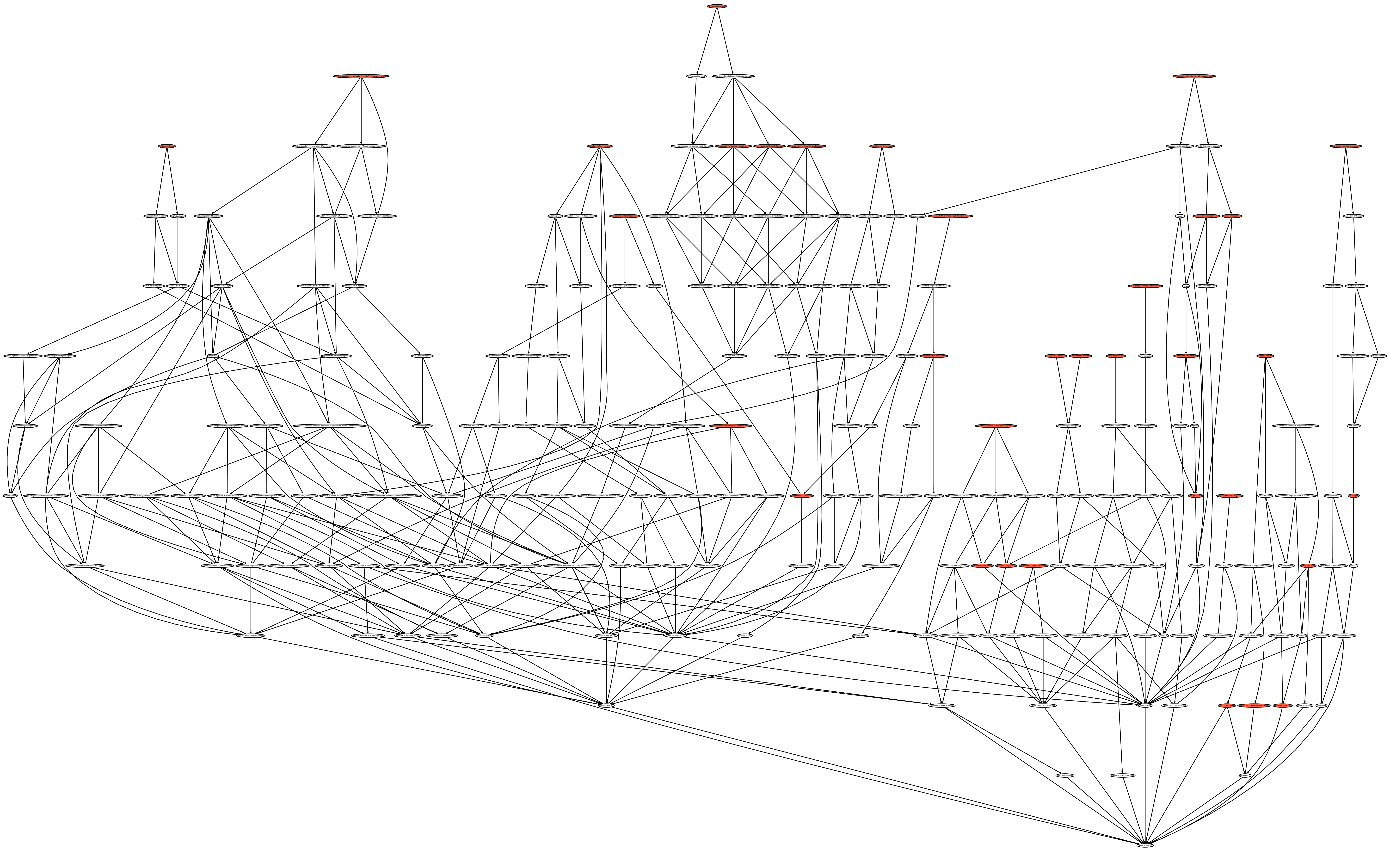

Directed Acyclic Graph of the 21 significant GO terms of the 80 genes in OE screen, Group A, MF

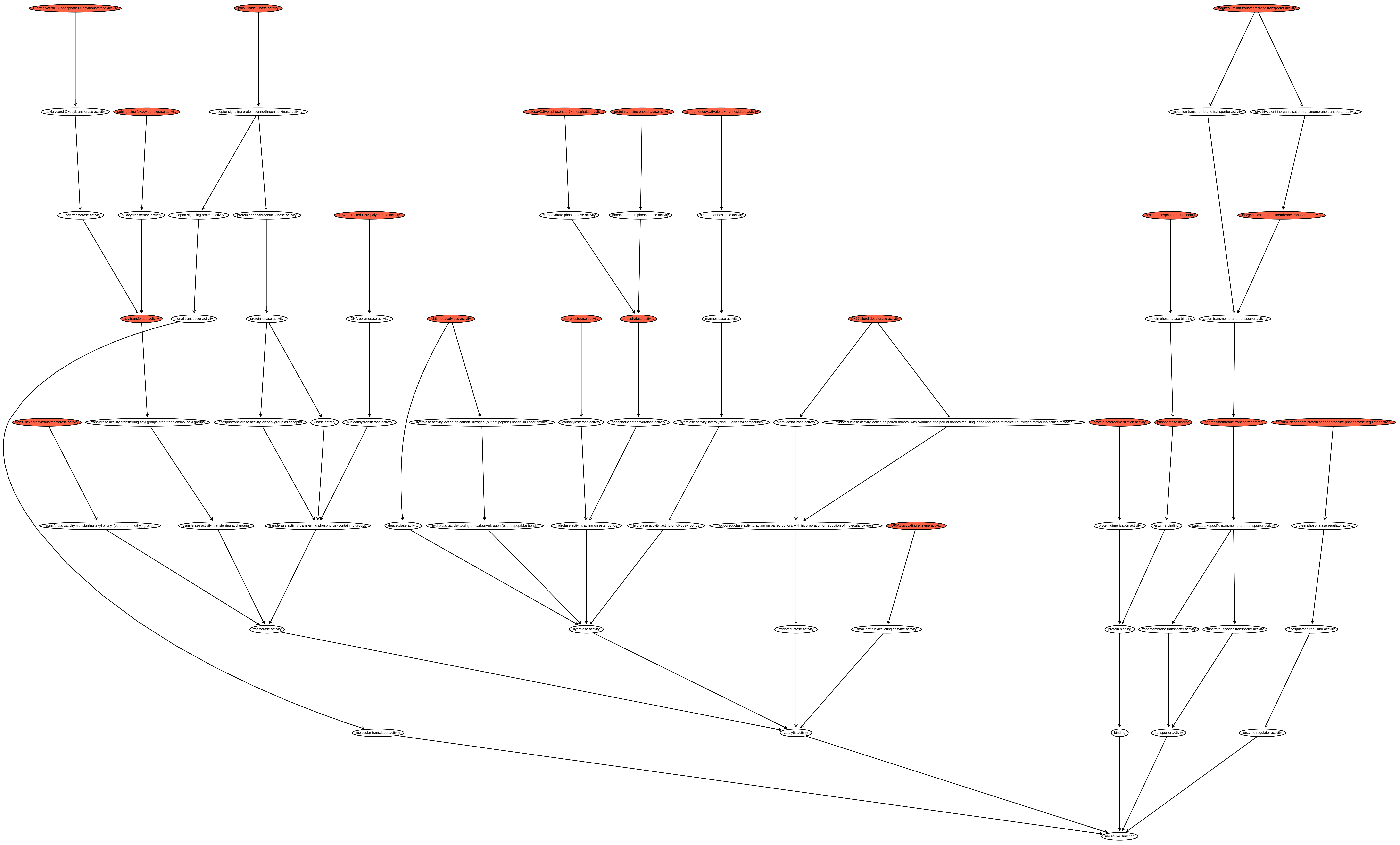

Supplement: Additional file 3: Figure S2 — Directed Acyclic Graph (DAG) and pie charts for Gene Ontology (GO) data for KO (A &B), KOd (C &D) and OE (D &F) gene datasets. The R packages GOstats, Rgraphviz and graphics were utilized to perform GO enrichment, generate the DAG plots and the pie plots. [file 1471-2164-13-623-S3.zip › Figure S2E.pdf]
