# Supplementary material for: High-resolution genome-wide scan of genes, gene-networks and cellular systems impacting the yeast ionome
Source: BMC Genomics. 2012 Nov 14;13:623. doi: 10.1186/1471-2164-13-623 (PMC3652779; doi:10.1186/1471-2164-13-623)

OE group C 36 genes, CC
pieCharts ID, Counts, P−values and GO terms (left)

- 1, 1, 0.00625, alpha−glucosidase II complex
- 2, 29, 0.00746, intracellular organelle
- 3, 1, 0.01247, zeta DNA polymerase complex
- 4, 1, 0.01247, NatA complex
- 5, 3, 0.01551, spindle pole
- 6, 18, 0.01572, nucleus
- 7, 27, 0.01725, membrane−bounded organelle
- 8, 1, 0.01865, eisosome
- 9, 1, 0.01865, Mdm10/Mdm12/Mmm1 complex
- 10, 1, 0.02479, nuclear MIS12/MIND type complex
- 11, 1, 0.02479, mitochondrial sorting and assembly machinery complex
- 12, 1, 0.02479, Ndc80 complex
- 13, 3, 0.0298, condensed chromosome
- 14, 1, 0.03507, kinetochore
- 15, 2, 0.03852, condensed nuclear chromosome, centromeric region
- 16, 3, 0.04134, microtubule cytoskeleton
- 17, 1, 0.04299, protein acetyltransferase complex
- 18, 2, 0.04886, condensed chromosome kinetochore
- 19, 1, 0.04898, U6 snRNP
- 20, 1, 0.04898, spindle midzone

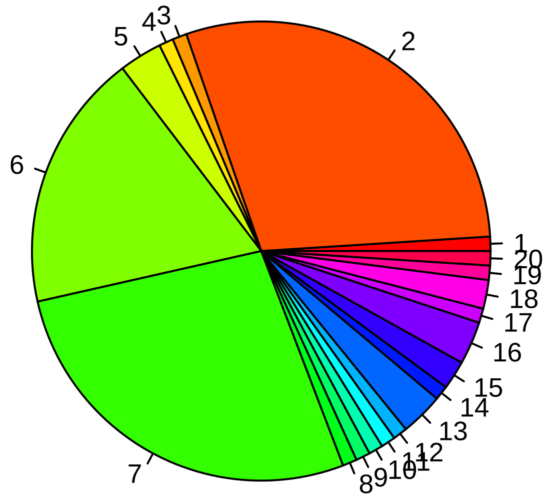

OE group C 36 genes, BP
pieCharts ID, Counts, P−values and GO terms (left)

1, 6, 0.00109, mitosis
2, 6, 0.00157, organelle fission
3, 1, 0.00625, cell morphogenesis checkpoint
4, 1, 0.00625, regulation of arginine metabolic process
5, 1, 0.00625, pyrimidine nucleotide transport
6, 1, 0.00625, G2/M transition size control checkpoint
7, 1, 0.00625, nicotinamide riboside transport
8, 1, 0.00625, maintenance of protein location in nucleus
9, 1, 0.00625, negative regulation of strand invasion
10, 2, 0.00671, microtubule nucleation
11, 3, 0.00916, post−Golgi vesicle−mediated transport
12, 2, 0.01039, regulation of microtubule cytoskeleton organization
13, 6, 0.01101, mitotic cell cycle
14, 4, 0.01118, chromosome segregation
15, 1, 0.01247, cis assembly of pre−catalytic spliceosome
16, 1, 0.01247, regulation of cellular amino acid metabolic process
17, 1, 0.01247, negative regulation of spindle pole body separation
18, 1, 0.01247, peptidyl−glutamine methylation
19, 2, 0.01385, mitochondrial genome maintenance
20, 2, 0.01773, regulation of transferase activity
21, 6, 0.0178, M phase
22, 1, 0.01865, thiamin transport
23, 1, 0.01865, error−prone postreplication DNA repair
24, 2, 0.02091, regulation of phosphorylation
25, 3, 0.02322, peptidyl−amino acid modification
26, 1, 0.02479, fatty acid elongation
27, 1, 0.02479, protein stabilization
28, 2, 0.0267, regulation of phosphorus metabolic process
29, 2, 0.03044, protein amino acid N−linked glycosylation
30, 1, 0.03089, positive regulation of transcription by galactose

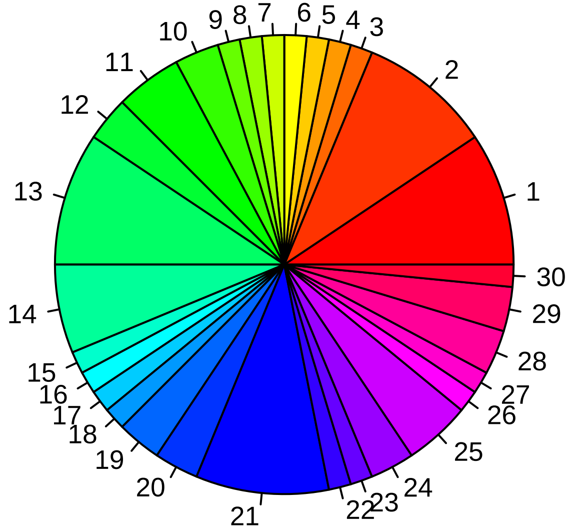

OE group C 36 genes, MF
pieCharts ID, Counts, P−values and GO terms (left)

- 1, 1, 0.00625, inositol trisphosphate 6−kinase activity
- 2, 1, 0.00625, inositol tetrakisphosphate 3−kinase activity
- 3, 1, 0.00625, inositol tetrakisphosphate 6−kinase activity
- 4, 1, 0.00625, pyrimidine nucleotide transmembrane transporter activity
- 5, 1, 0.00625, nickel ion binding
- 6, 1, 0.00625, nicotinamide riboside transporter activity
- 7, 1, 0.00625, dolichyl pyrophosphate Man9GlcNAc2 alpha−1,3−glucosyltransferase activity
- 8, 1, 0.00625, electron transporter, transferring electrons within CoQH2−cytochrome c reductase complex activity
- 9, 1, 0.00625, flap−structured DNA binding
- 10, 1, 0.01247, inositol 1,3,4,5,6−pentakisphosphate kinase activity
- 11, 1, 0.01247, inositol trisphosphate 3−kinase activity
- 12, 1, 0.01865, fatty acid elongase activity
- 13, 1, 0.02406, nucleobase, nucleoside, nucleotide and nucleic acid transmembrane transporter activity
- 14, 1, 0.02479, alpha−glucosidase activity
- 15, 1, 0.02479, phosphoinositide 3−kinase activity
- 16, 2, 0.03712, structural constituent of cytoskeleton
- 17, 1, 0.04299, protein tyrosine kinase activity
- 18, 1, 0.04898, peptide alpha−N−acetyltransferase activity
- 19, 1, 0.04898, nucleobase transmembrane transporter activity
- 20, 1, 0.04898, oxidoreductase activity, acting on single donors with incorporation of molecular oxygen, incorporation of two atoms of oxygen

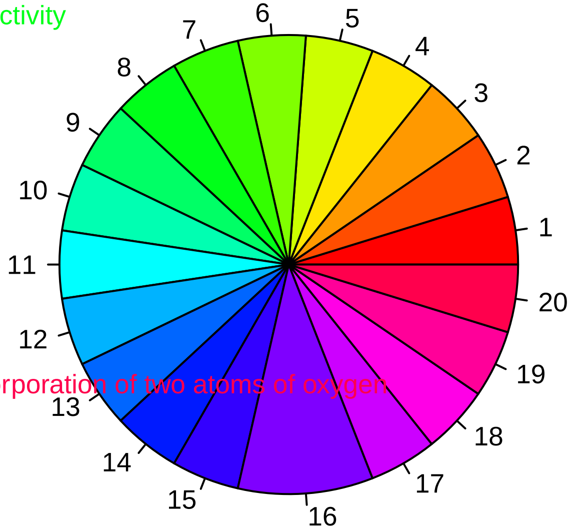

OE group B 330 genes, CC
pieCharts ID, Counts, P−values and GO terms (left)

1, 4, 0.00303, cellular bud neck contractile ring
2, 28, 0.01471, plasma membrane
3, 2, 0.01998, Ski complex
4, 5, 0.02394, proteasome storage granule
5, 3, 0.03165, endoplasmic reticulum lumen
6, 23, 0.0359, nuclear chromosome
7, 3, 0.03936, proteasome core complex
8, 4, 0.03983, cell division site part
9, 4, 0.04601, mitochondrial respiratory chain
10, 4, 0.04601, integral to plasma membrane
11, 2, 0.0461, protein phosphatase type 2A complex
12, 2, 0.0461, t−UTP complex
13, 3, 0.04792, cyclin−dependent protein kinase holoenzyme complex

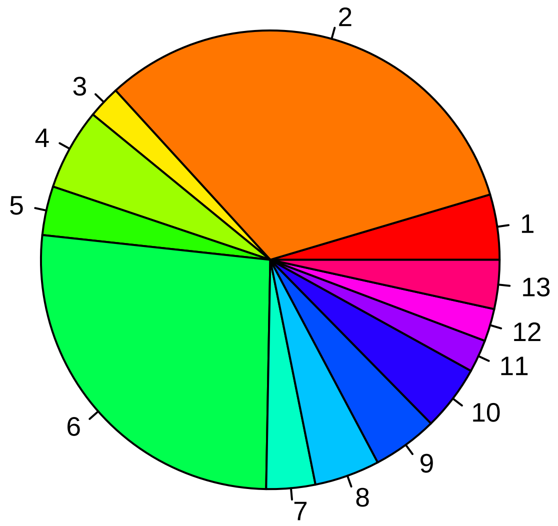

OE group B 330 genes, BP
pieCharts ID, Counts, P−values and GO terms (left)

- 1, 2, 0.00361, adenine salvage
- 2, 2, 0.00361, tetrahydrobiopterin biosynthetic process
- 3, 56, 0.00635, cellular catabolic process
- 4, 18, 0.00713, cellular response to heat
- 5, 23, 0.00773, proteolysis involved in cellular protein catabolic process
- 6, 2, 0.0104, activation of adenylate cyclase activity
- 7, 2, 0.0104, thiamin transport
- 8, 2, 0.0104, positive regulation of cyclase activity
- 9, 2, 0.0104, regulation of DNA replication during S phase
- 10, 2, 0.0104, positive regulation of lyase activity
- 11, 38, 0.01156, cellular biopolymer catabolic process
- 12, 23, 0.01161, modification−dependent macromolecule catabolic process
- 13, 10, 0.01246, asexual reproduction
- 14, 11, 0.01336, aerobic respiration
- 15, 6, 0.01456, vesicle fusion
- 16, 2, 0.01998, glycogen catabolic process
- 17, 2, 0.01998, cAMP biosynthetic process
- 18, 2, 0.01998, polyphosphate metabolic process
- 19, 2, 0.01998, regulation of cyclic nucleotide biosynthetic process
- 20, 2, 0.01998, regulation of cAMP metabolic process
- 21, 2, 0.01998, regulation of adenylate cyclase activity
- 22, 5, 0.02065, bipolar cellular bud site selection
- 23, 19, 0.02119, response to temperature stimulus
- 24, 8, 0.02381, protein targeting to vacuole
- 25, 3, 0.02482, positive regulation of transcription from RNA polymerase I promoter
- 26, 56, 0.02671, response to stress
- 27, 34, 0.02746, protein catabolic process
- 28, 5, 0.02754, nucleoside monophosphate biosynthetic process
- 29, 13, 0.0308, positive regulation of RNA metabolic process
- 30, 3, 0.03165, protein retention in Golgi apparatus

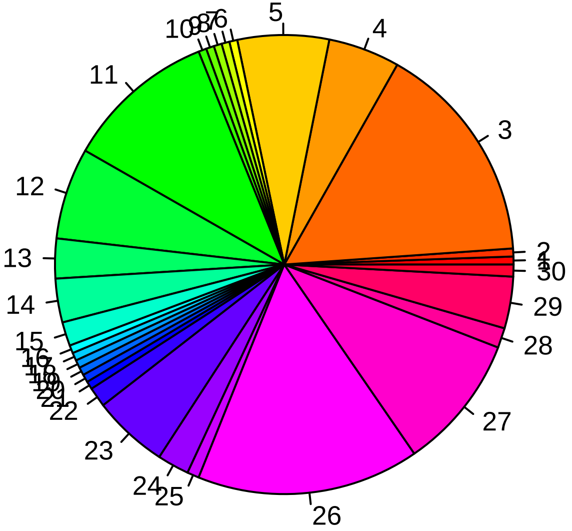

OE group B 330 genes, MF
pieCharts ID, Counts, P−values and GO terms (left)

1, 14, 0.00147, GTP binding
2, 14, 0.00147, guanyl nucleotide binding
3, 7, 0.00163, di−, tri−valent inorganic cation transmembrane transporter activity
4, 4, 0.00202, protein tag
5, 2, 0.00361, adenine phosphoribosyltransferase activity
6, 3, 0.0063, glutathione transferase activity
7, 2, 0.0104, copper uptake transmembrane transporter activity
8, 2, 0.0104, guanylyltransferase activity
9, 5, 0.01048, transition metal ion transmembrane transporter activity
10, 3, 0.01382, cyclin−dependent protein kinase activity
11, 13, 0.0152, cation transmembrane transporter activity
12, 2, 0.03199, transferase activity, transferring amino−acyl groups
13, 3, 0.03936, threonine−type endopeptidase activity
14, 3, 0.04792, protein−lysine N−methyltransferase activity

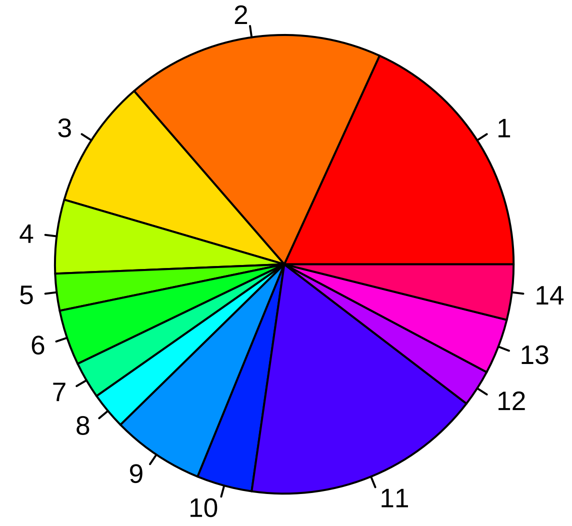

OE group A 80 genes, CC
pieCharts ID, Counts, P−values and GO terms (left)

1, 2, 0.00381, nuclear microtubule
2, 2, 0.00504, Arp2/3 protein complex
3, 1, 0.01388, telomerase holoenzyme complex
4, 1, 0.02756, chitosan layer of spore wall
5, 1, 0.02756, mitochondrial processing peptidase complex
6, 1, 0.02756, NatA complex
7, 1, 0.02756, Rhp55−Rhp57 complex
8, 5, 0.02812, microtubule cytoskeleton
9, 2, 0.03981, mitochondrial respiratory chain
10, 1, 0.04106, polar microtubule
11, 1, 0.04106, checkpoint clamp complex
12, 1, 0.04106, tubulin complex

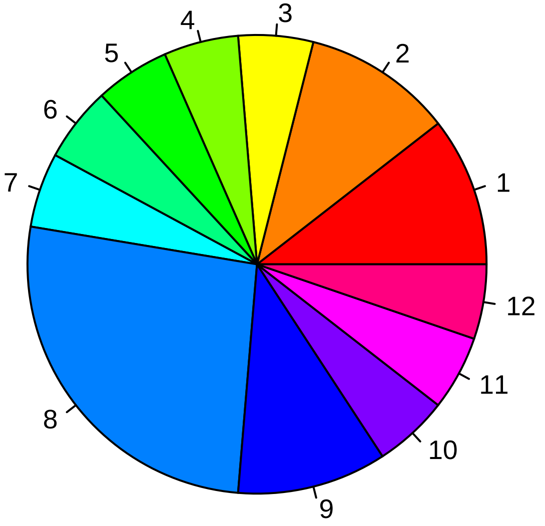

OE group A 80 genes, BP
pieCharts ID, Counts, P−values and GO terms (left)

- 1, 3, 0.0059, regulation of cellular carbohydrate metabolic process
- 2, 3, 0.00952, protein amino acid dephosphorylation
- 3, 5, 0.01164, ascospore formation
- 4, 5, 0.01164, sexual sporulation
- 5, 1, 0.01388, ATP catabolic process
- 6, 1, 0.01388, purine nucleoside triphosphate catabolic process
- 7, 1, 0.01388, purine ribonucleotide catabolic process
- 8, 1, 0.01388, ribonucleoside triphosphate catabolic process
- 9, 1, 0.01388, negative regulation of transcription from RNA polymerase III promoter
- 10, 1, 0.01388, synaptonemal complex disassembly
- 11, 7, 0.01515, reproduction of a single−celled organism
- 12, 2, 0.01551, regulation of gluconeogenesis
- 13, 2, 0.01551, protein polymerization
- 14, 6, 0.02163, reproductive process
- 15, 5, 0.02253, microtubule−based process
- 16, 7, 0.02377, cytoskeleton organization
- 17, 11, 0.02648, cell cycle phase
- 18, 3, 0.02686, fungal−type cell wall biogenesis
- 19, 1, 0.02756, G1/S transition checkpoint
- 20, 1, 0.02756, mitochondrial magnesium ion transport
- 21, 1, 0.02756, mRNA stabilization
- 22, 6, 0.03277, M phase of meiotic cell cycle
- 23, 6, 0.03605, ion transport
- 24, 8, 0.03738, phosphate metabolic process
- 25, 1, 0.04106, resolution of meiotic joint molecules as recombinants
- 26, 1, 0.04106, G2/M transition checkpoint
- 27, 1, 0.04106, Arp2/3 complex−mediated actin nucleation
- 28, 1, 0.04106, ceramide biosynthetic process
- 29, 1, 0.04106, chromosome separation
- 30, 2, 0.04304, mitochondrial ATP synthesis coupled electron transport

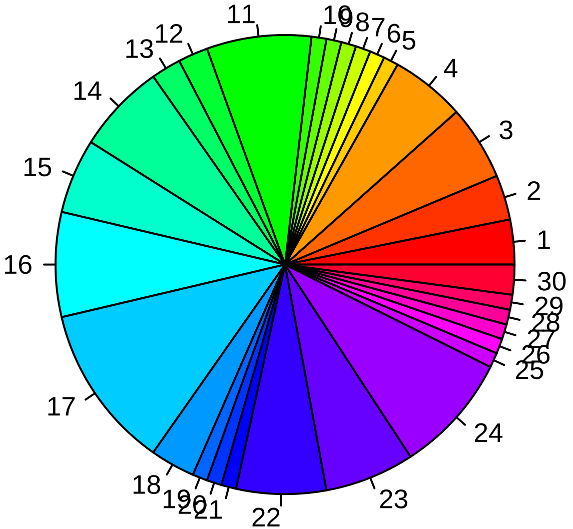

OE group A 80 genes, MF
pieCharts ID, Counts, P−values and GO terms (left)

- 1, 2, 0.00381, protein heterodimerization activity
- 2, 1, 0.01388, trans−hexaprenyltranstransferase activity
- 3, 1, 0.01388, C−22 sterol desaturase activity
- 4, 1, 0.01388, calcium−dependent protein serine/threonine phosphatase regulator activity
- 5, 1, 0.01388, protein phosphatase 2B binding
- 6, 1, 0.01388, URM1 activating enzyme activity
- 7, 1, 0.01388, polo kinase kinase activity
- 8, 2, 0.02009, protein tyrosine phosphatase activity
- 9, 1, 0.02756, chitin deacetylase activity
- 10, 1, 0.02756, fructose−2,6−bisphosphate 2−phosphatase activity
- 11, 1, 0.02756, sterol esterase activity
- 12, 1, 0.02756, mannan endo−1,6−alpha−mannosidase activity
- 13, 1, 0.02756, sphingosine N−acyltransferase activity
- 14, 5, 0.03752, ion transmembrane transporter activity
- 15, 1, 0.04106, 1−acylglycerol−3−phosphate O−acyltransferase activity
- 16, 1, 0.04106, RNA−directed DNA polymerase activity
- 17, 1, 0.04106, magnesium ion transmembrane transporter activity
- 18, 1, 0.04106, phosphatase binding
- 19, 4, 0.04213, phosphatase activity
- 20, 4, 0.04213, inorganic cation transmembrane transporter activity
- 21, 4, 0.04636, acyltransferase activity

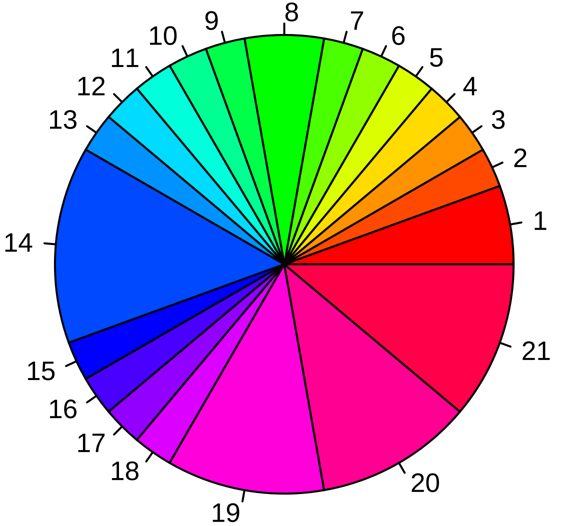

Supplement: Additional file 3: Figure S2 — Directed Acyclic Graph (DAG) and pie charts for Gene Ontology (GO) data for KO (A &B), KOd (C &D) and OE (D &F) gene datasets. The R packages GOstats, Rgraphviz and graphics were utilized to perform GO enrichment, generate the DAG plots and the pie plots. [file 1471-2164-13-623-S3.zip › Figure S2F.pdf]
