# Supplementary material for: High-resolution genome-wide scan of genes, gene-networks and cellular systems impacting the yeast ionome
Source: BMC Genomics. 2012 Nov 14;13:623. doi: 10.1186/1471-2164-13-623 (PMC3652779; doi:10.1186/1471-2164-13-623)
Supplement: Additional file 4: Figure S3 — Clusters of ionomic profiles using the exhaustive significance clustering (ESC) method for the KO (A), KOd (B) and OE (C) data sets of genes that have a significant impact on the ionome. The X-axis represents the elements used in the clustering and the Y-axis represents the moderated Z values used for each element. Only the genes that significantly affect at least one element and pass the annealing process are included, and only the clusters that include at least 3 genes are shown. [file 1471-2164-13-623-S4.zip › Figure S3A.pdf]

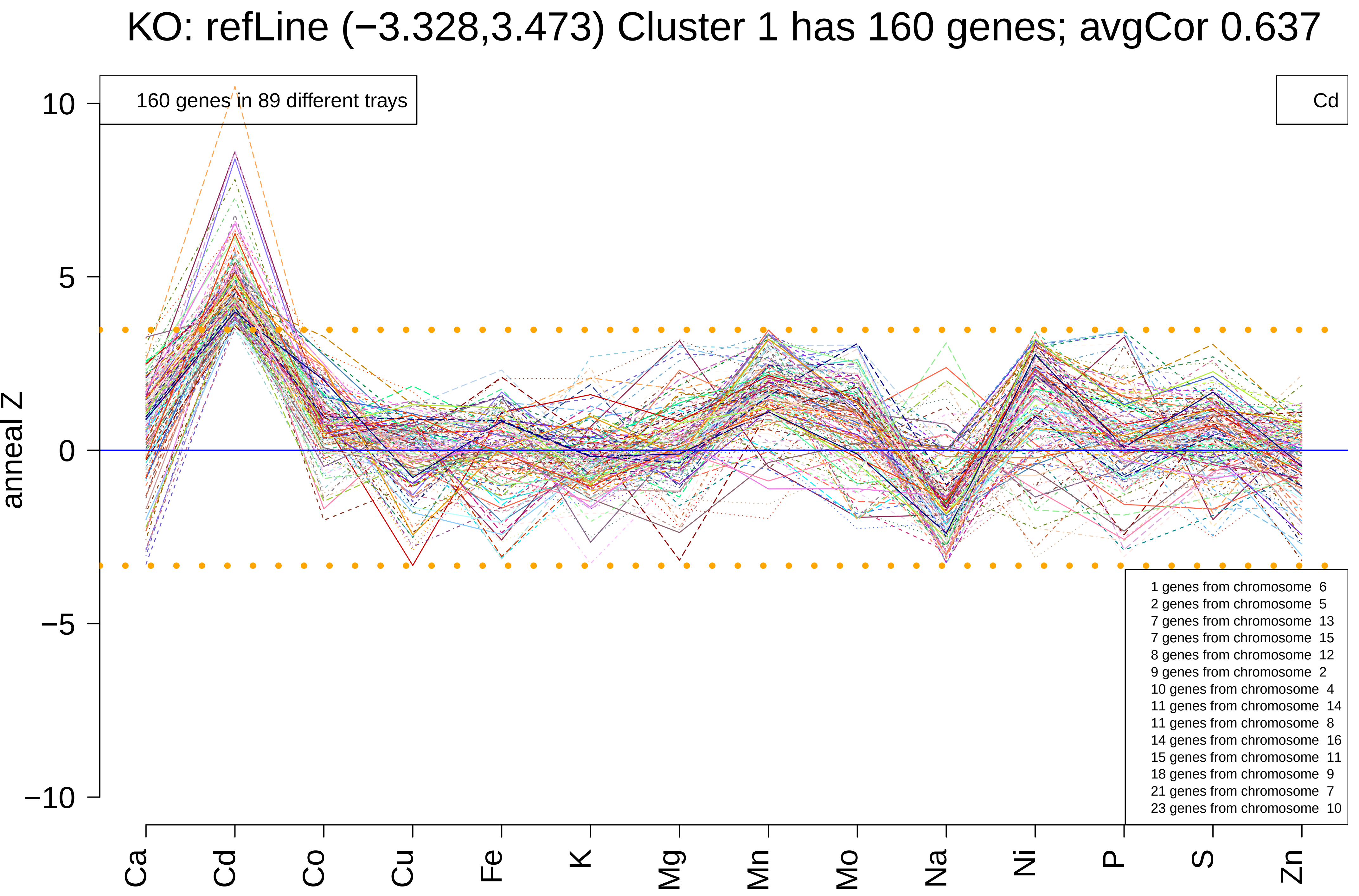

KO: refLine (−3.328,3.473) Cluster 1 has 160 genes; avgCor 0.637
160 genes in 89 different trays
Cd
anneal Z
10
5
0
−5
−10
Ca
Cd
Co
Cu
Fe
K
Mg
Mn
Mo
Na
Ni
P
S
Zn
1 genes from chromosome 6
2 genes from chromosome 5
7 genes from chromosome 13
7 genes from chromosome 15
8 genes from chromosome 12
9 genes from chromosome 2
10 genes from chromosome 4
11 genes from chromosome 14
11 genes from chromosome 8
14 genes from chromosome 16
15 genes from chromosome 11
18 genes from chromosome 9
21 genes from chromosome 7
23 genes from chromosome 10

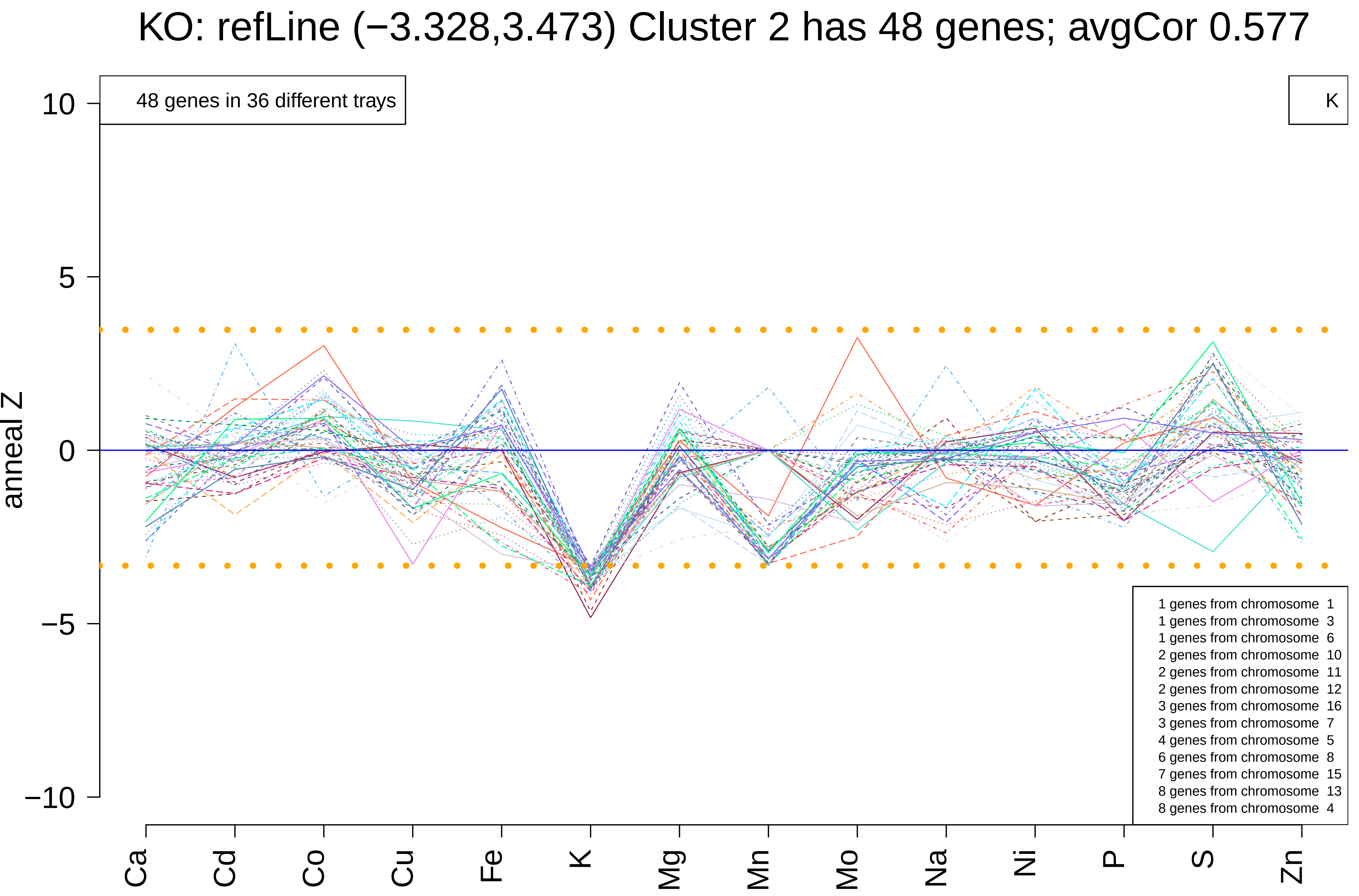
KO: refLine (−3.328,3.473) Cluster 2 has 48 genes; avgCor 0.577
48 genes in 36 different trays
K
anneal Z
10
5
0
−5
−10
Ca
Cd
Co
Cu
Fe
K
Mg
Mn
Mo
Na
Ni
P
S
Zn
1 genes from chromosome 1
1 genes from chromosome 3
1 genes from chromosome 6
2 genes from chromosome 10
2 genes from chromosome 11
2 genes from chromosome 12
3 genes from chromosome 16
3 genes from chromosome 7
4 genes from chromosome 5
6 genes from chromosome 8
7 genes from chromosome 15
8 genes from chromosome 13
8 genes from chromosome 4

KO: refLine (−3.328,3.473) Cluster 3 has 41 genes; avgCor 0.533

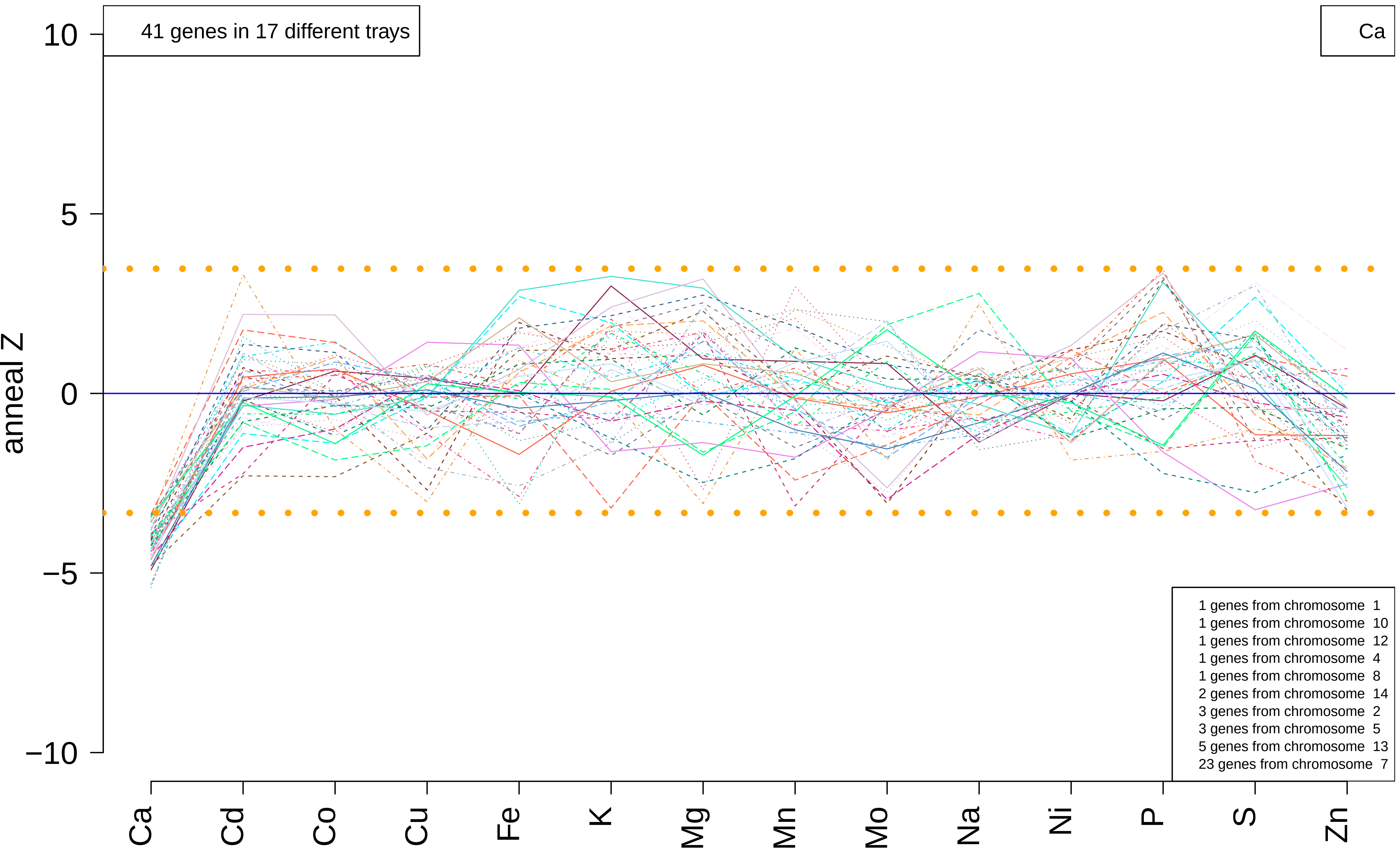

KO: refLine (−3.328,3.473) Cluster 4 has 31 genes; avgCor 0.495

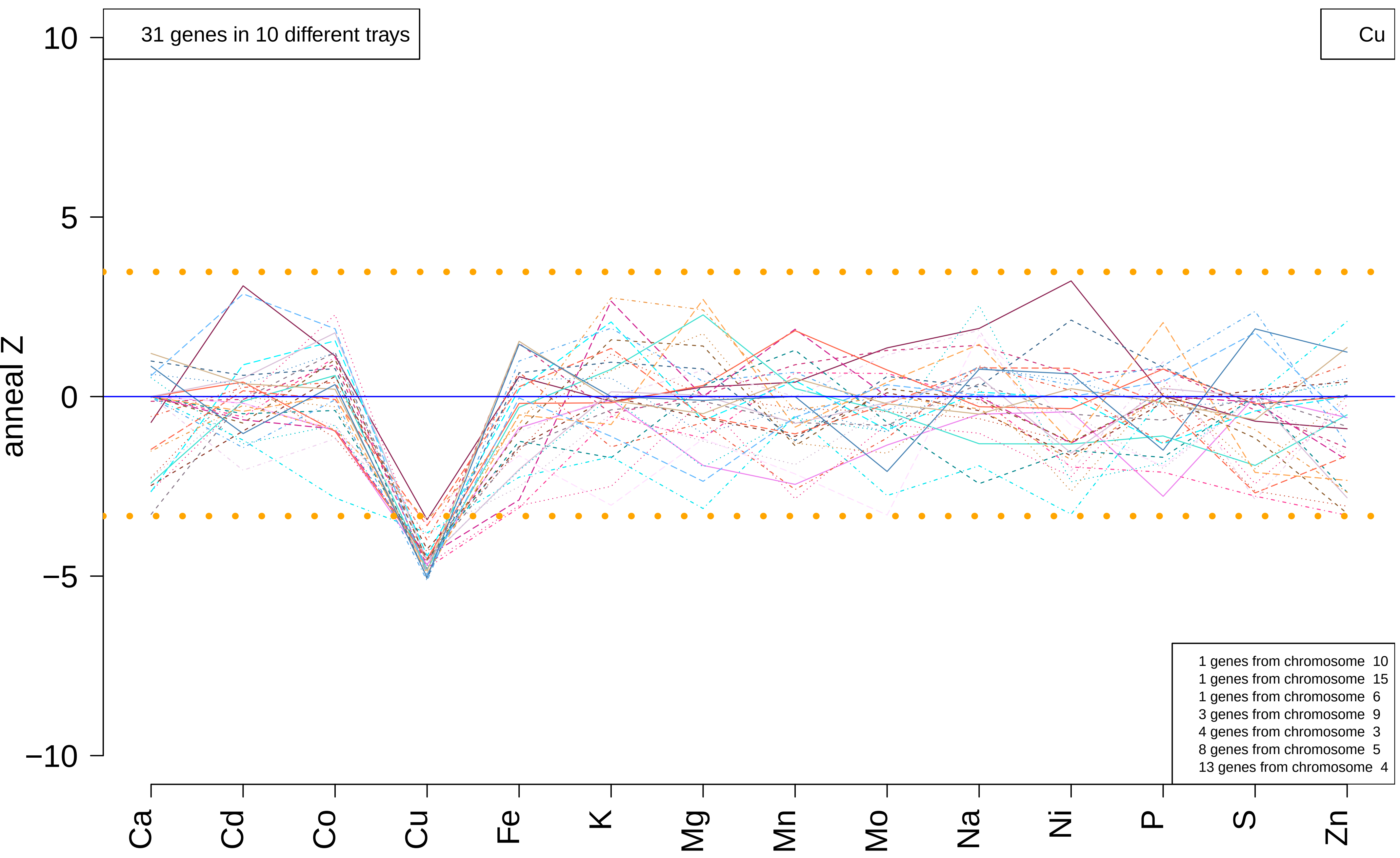

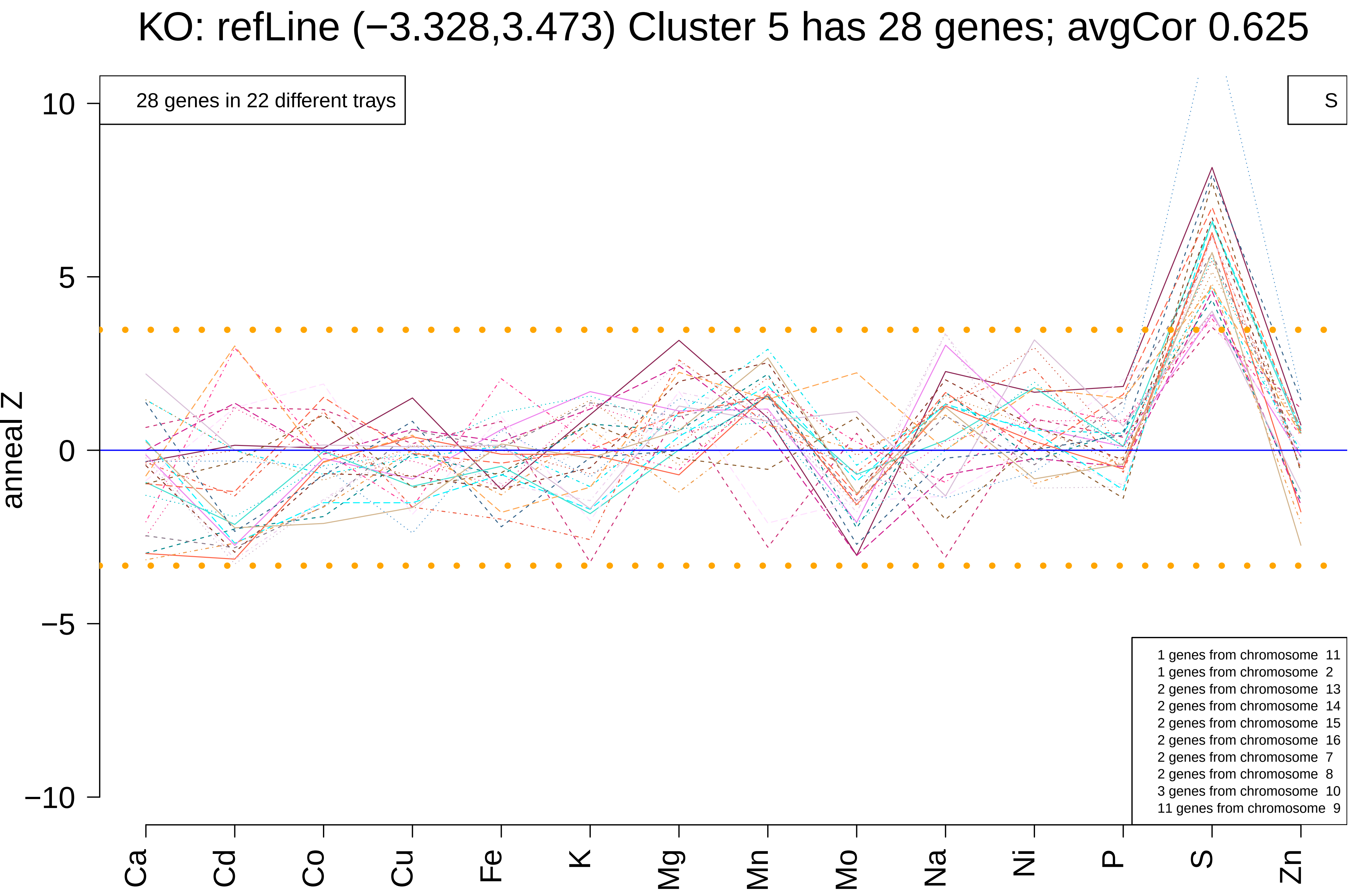
KO: refLine (−3.328,3.473) Cluster 5 has 28 genes; avgCor 0.625
28 genes in 22 different trays
S
anneal Z
10
5
0
−5
−10
Ca
Cd
Co
Cu
Fe
K
Mg
Mn
Mo
Na
Ni
P
S
Zn
1 genes from chromosome 11
1 genes from chromosome 2
2 genes from chromosome 13
2 genes from chromosome 14
2 genes from chromosome 15
2 genes from chromosome 16
2 genes from chromosome 7
2 genes from chromosome 8
3 genes from chromosome 10
11 genes from chromosome 9

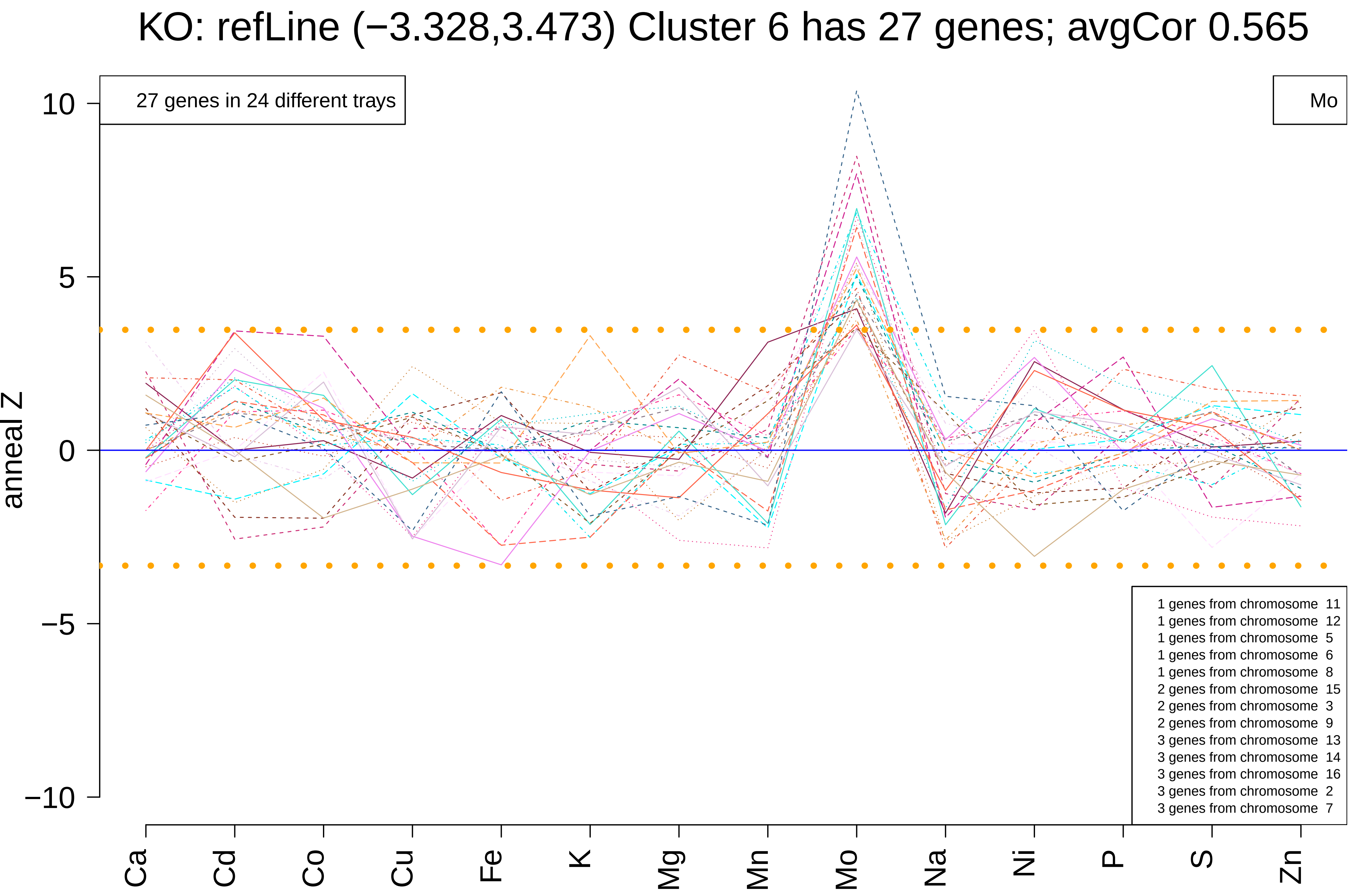
KO: refLine (−3.328,3.473) Cluster 6 has 27 genes; avgCor 0.565
27 genes in 24 different trays
Mo
anneal Z
10
5
0
−5
−10
Ca
Cd
Co
Cu
Fe
K
Mg
Mn
Mo
Na
Ni
P
S
Zn
1 genes from chromosome 11
1 genes from chromosome 12
1 genes from chromosome 5
1 genes from chromosome 6
1 genes from chromosome 8
2 genes from chromosome 15
2 genes from chromosome 3
2 genes from chromosome 9
3 genes from chromosome 13
3 genes from chromosome 14
3 genes from chromosome 16
3 genes from chromosome 2
3 genes from chromosome 7

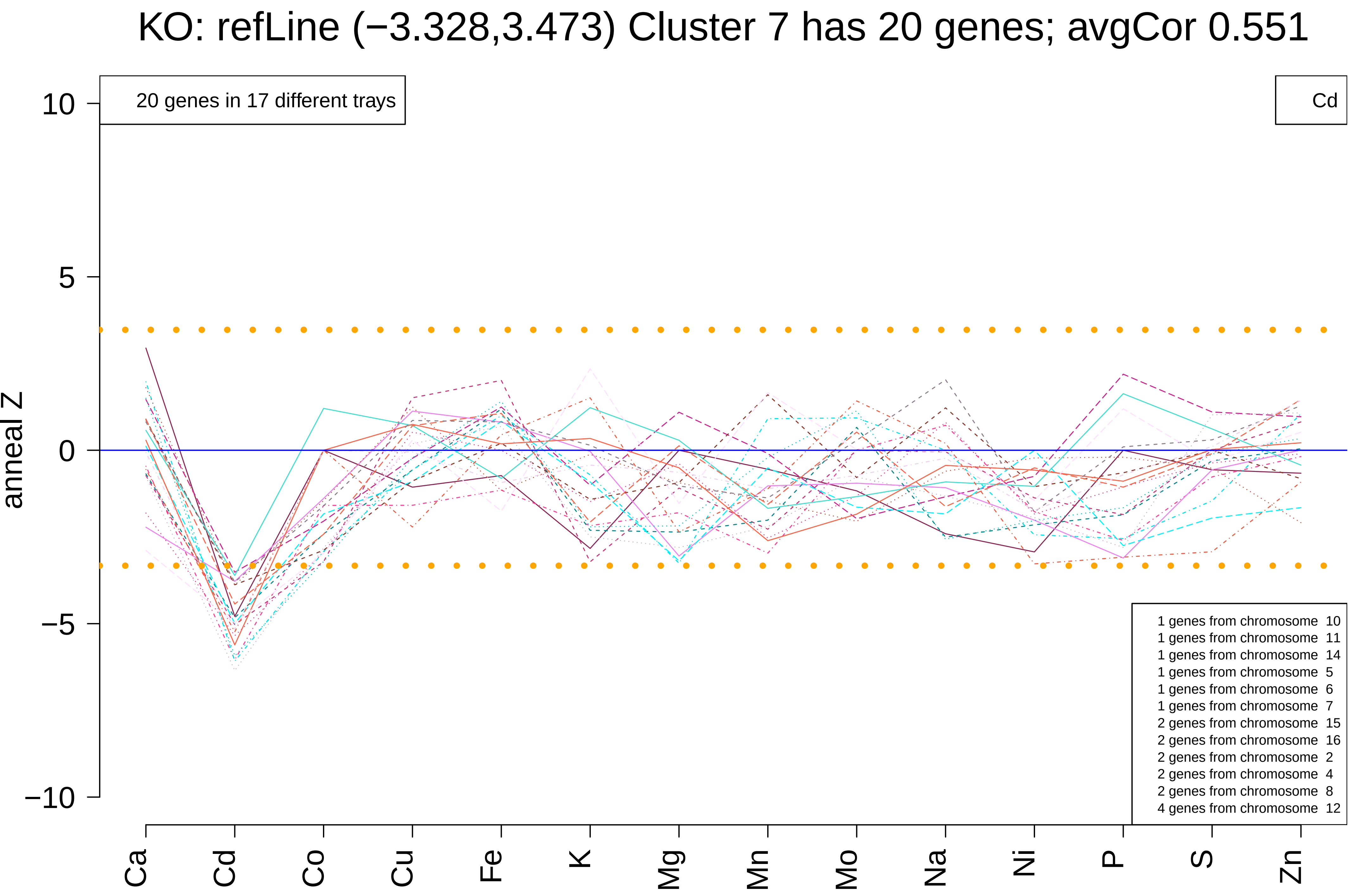
KO: refLine (−3.328,3.473) Cluster 7 has 20 genes; avgCor 0.551
20 genes in 17 different trays
Cd
anneal Z
10
5
0
−5
−10
Ca
Cd
Co
Cu
Fe
K
Mg
Mn
Mo
Na
Ni
P
S
Zn
1 genes from chromosome 10
1 genes from chromosome 11
1 genes from chromosome 14
1 genes from chromosome 5
1 genes from chromosome 6
1 genes from chromosome 7
2 genes from chromosome 15
2 genes from chromosome 16
2 genes from chromosome 2
2 genes from chromosome 4
2 genes from chromosome 8
4 genes from chromosome 12

KO: refLine (−3.328,3.473) Cluster 8 has 15 genes; avgCor 0.707

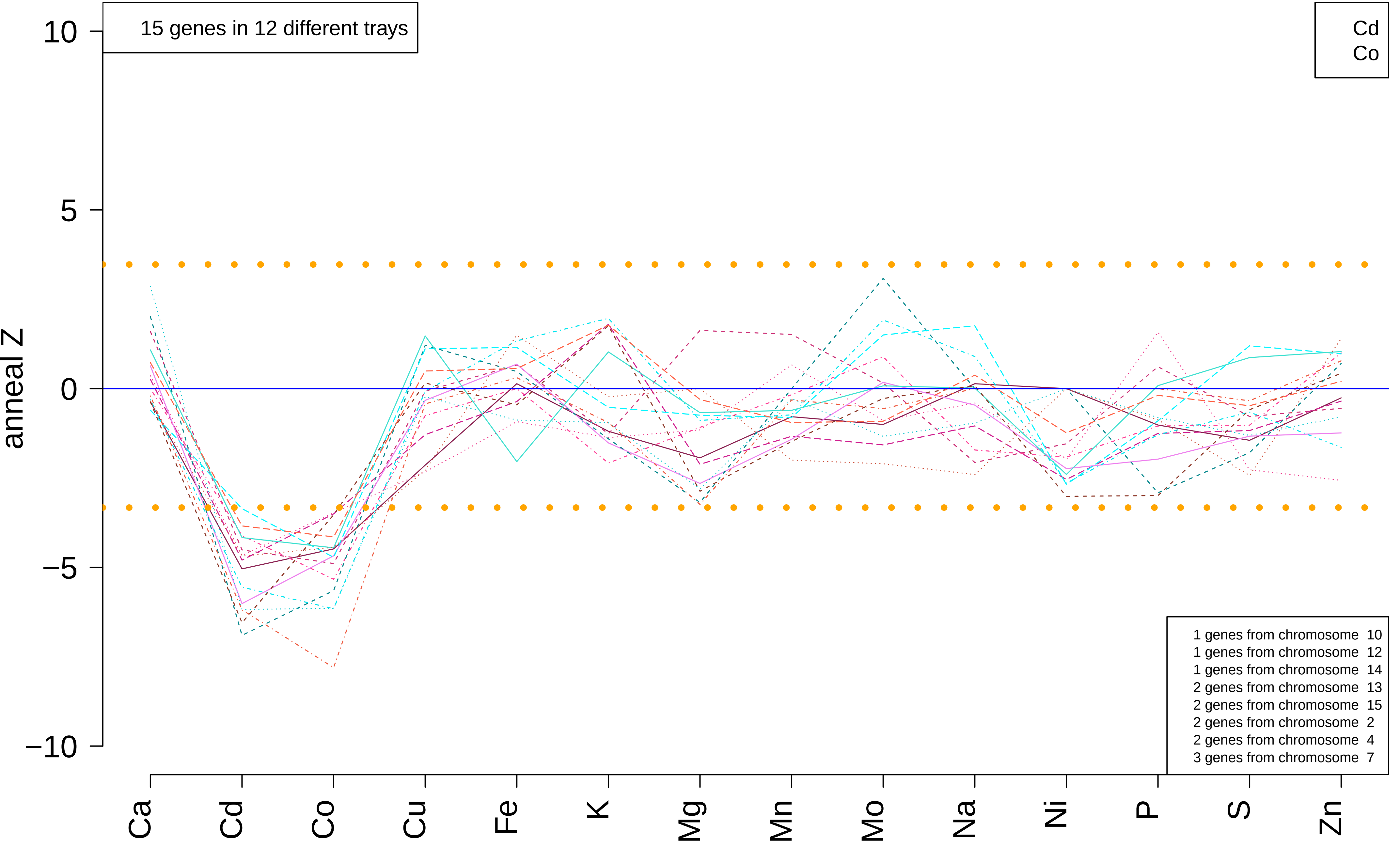

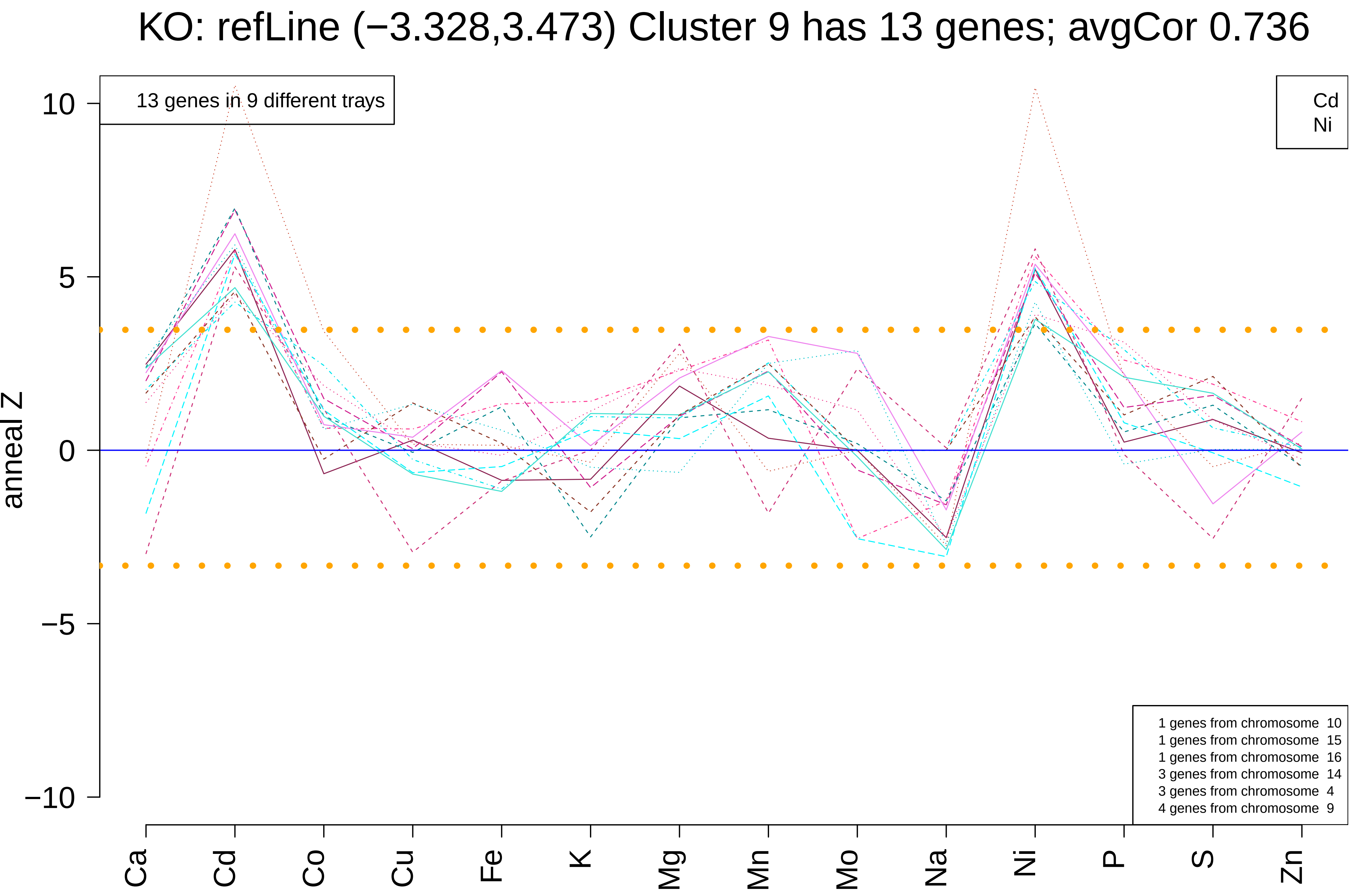

KO: refLine (−3.328,3.473) Cluster 9 has 13 genes; avgCor 0.736
13 genes in 9 different trays
Cd
Ni
anneal Z
10
5
0
−5
−10
Ca
Cd
Co
Cu
Fe
K
Mg
Mn
Mo
Na
Ni
P
S
Zn
1 genes from chromosome 10
1 genes from chromosome 15
1 genes from chromosome 16
3 genes from chromosome 14
3 genes from chromosome 4
4 genes from chromosome 9

KO: refLine (−3.328,3.473) Cluster 10 has 12 genes; avgCor 0.474

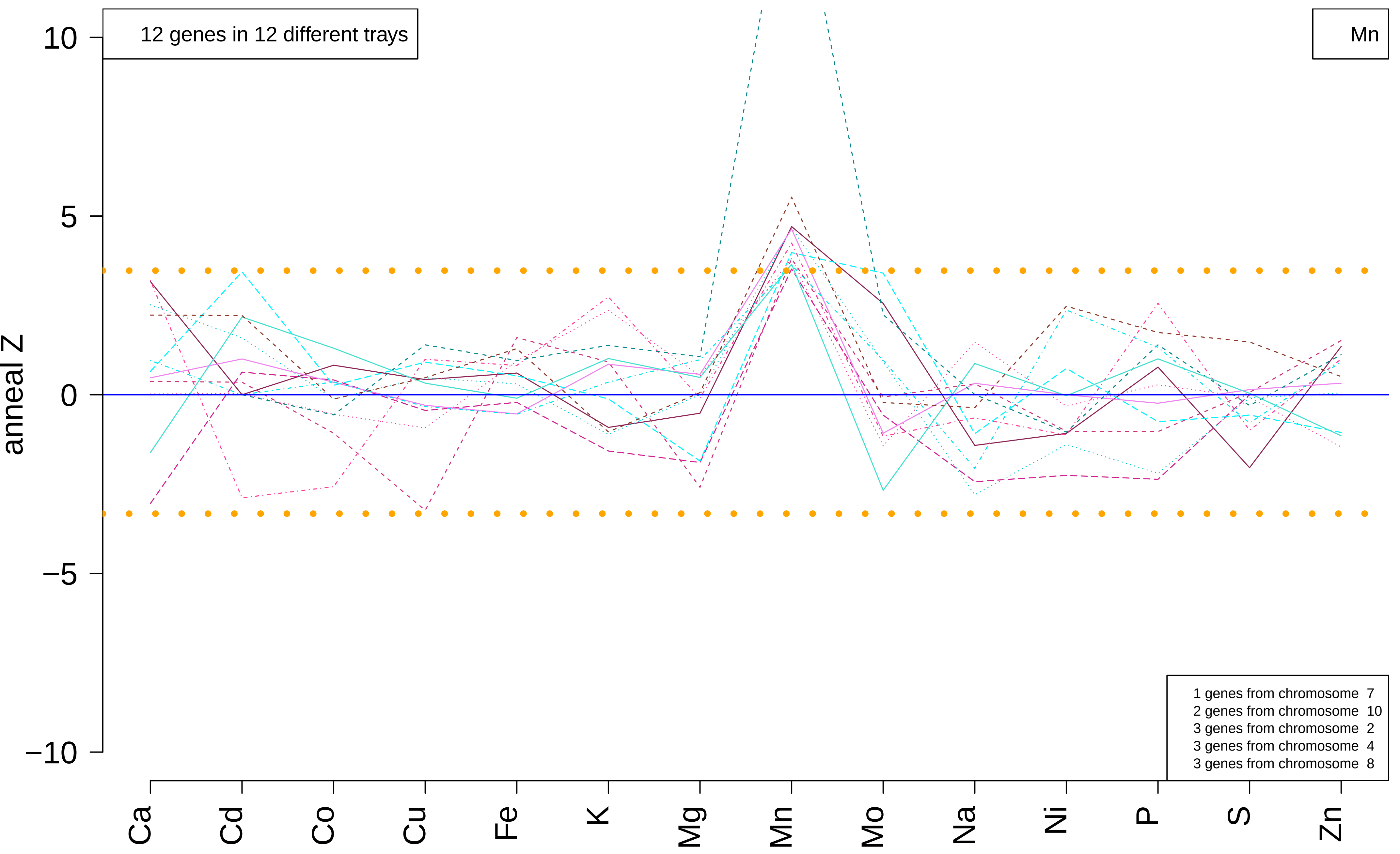

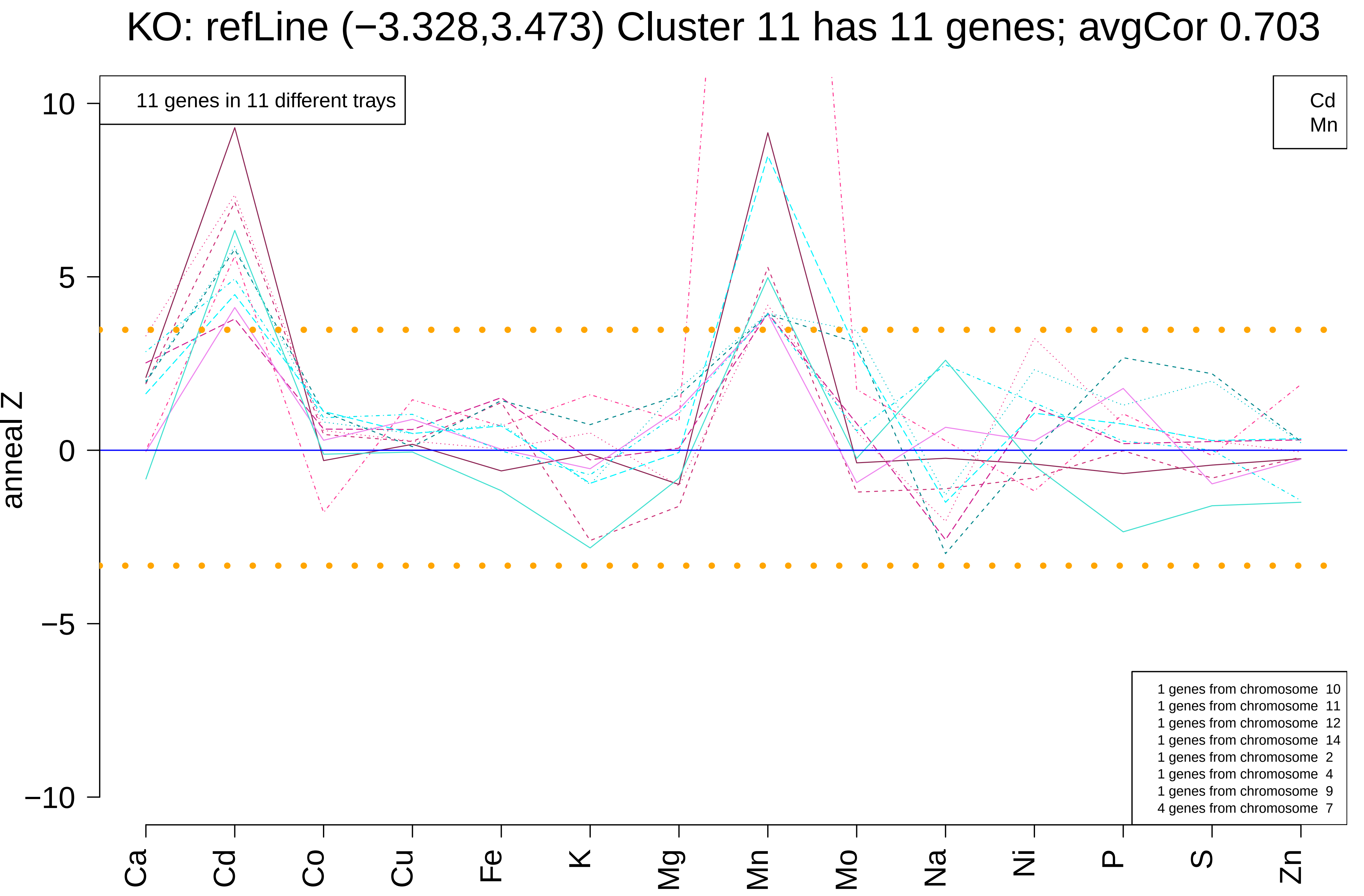
KO: refLine (−3.328,3.473) Cluster 11 has 11 genes; avgCor 0.703
11 genes in 11 different trays
Cd
Mn
anneal Z
10
5
0
−5
−10
Ca
Cd
Co
Cu
Fe
K
Mg
Mn
Mo
Na
Ni
P
S
Zn
1 genes from chromosome 10
1 genes from chromosome 11
1 genes from chromosome 12
1 genes from chromosome 14
1 genes from chromosome 2
1 genes from chromosome 4
1 genes from chromosome 9
4 genes from chromosome 7

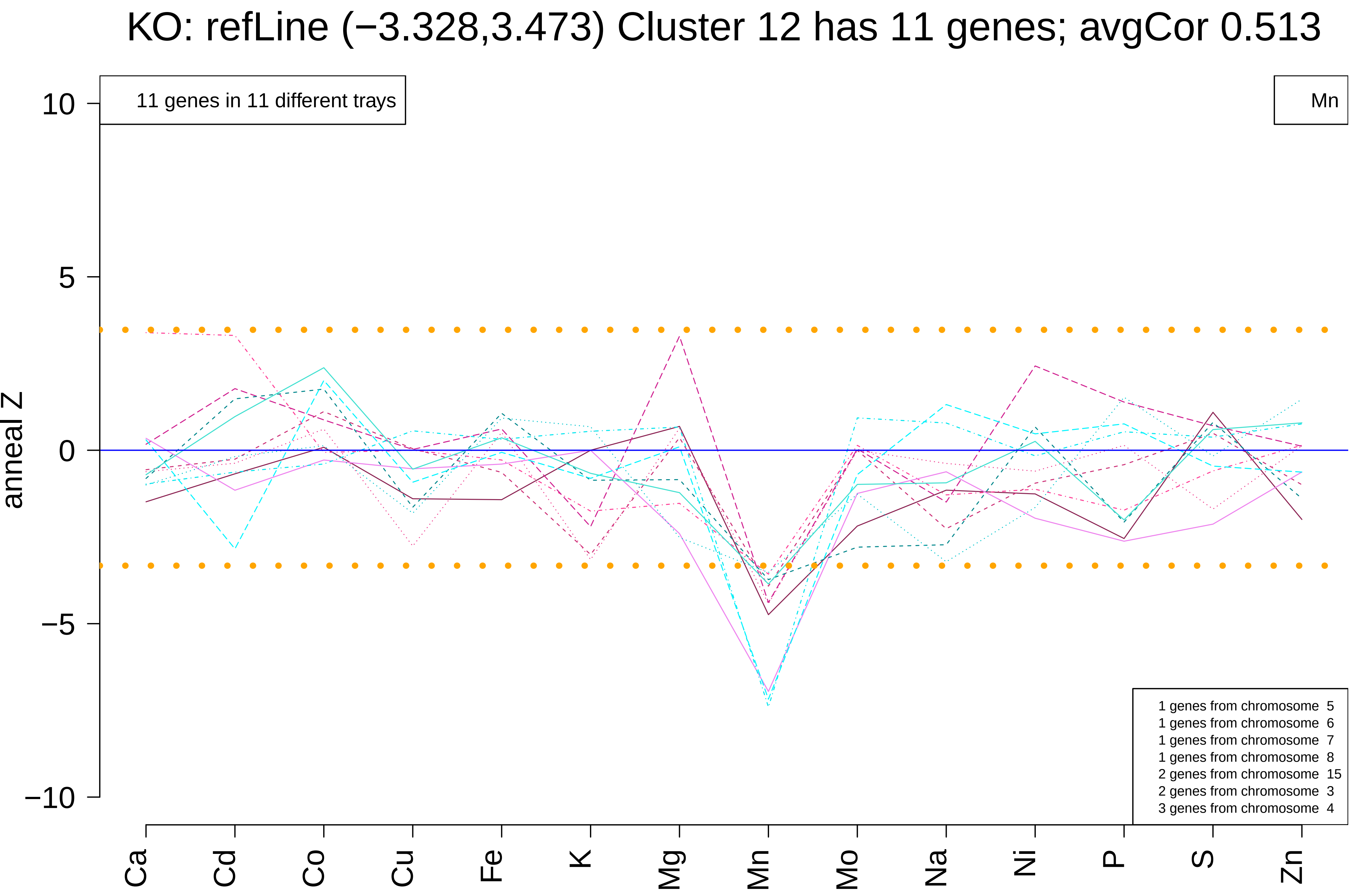
KO: refLine (−3.328,3.473) Cluster 12 has 11 genes; avgCor 0.513
11 genes in 11 different trays
Mn
anneal Z
10
5
0
−5
−10
Ca
Cd
Co
Cu
Fe
K
Mg
Mn
Mo
Na
Ni
P
S
Zn
1 genes from chromosome 5
1 genes from chromosome 6
1 genes from chromosome 7
1 genes from chromosome 8
2 genes from chromosome 15
2 genes from chromosome 3
3 genes from chromosome 4

KO: refLine (−3.328,3.473) Cluster 13 has 11 genes; avgCor 0.579

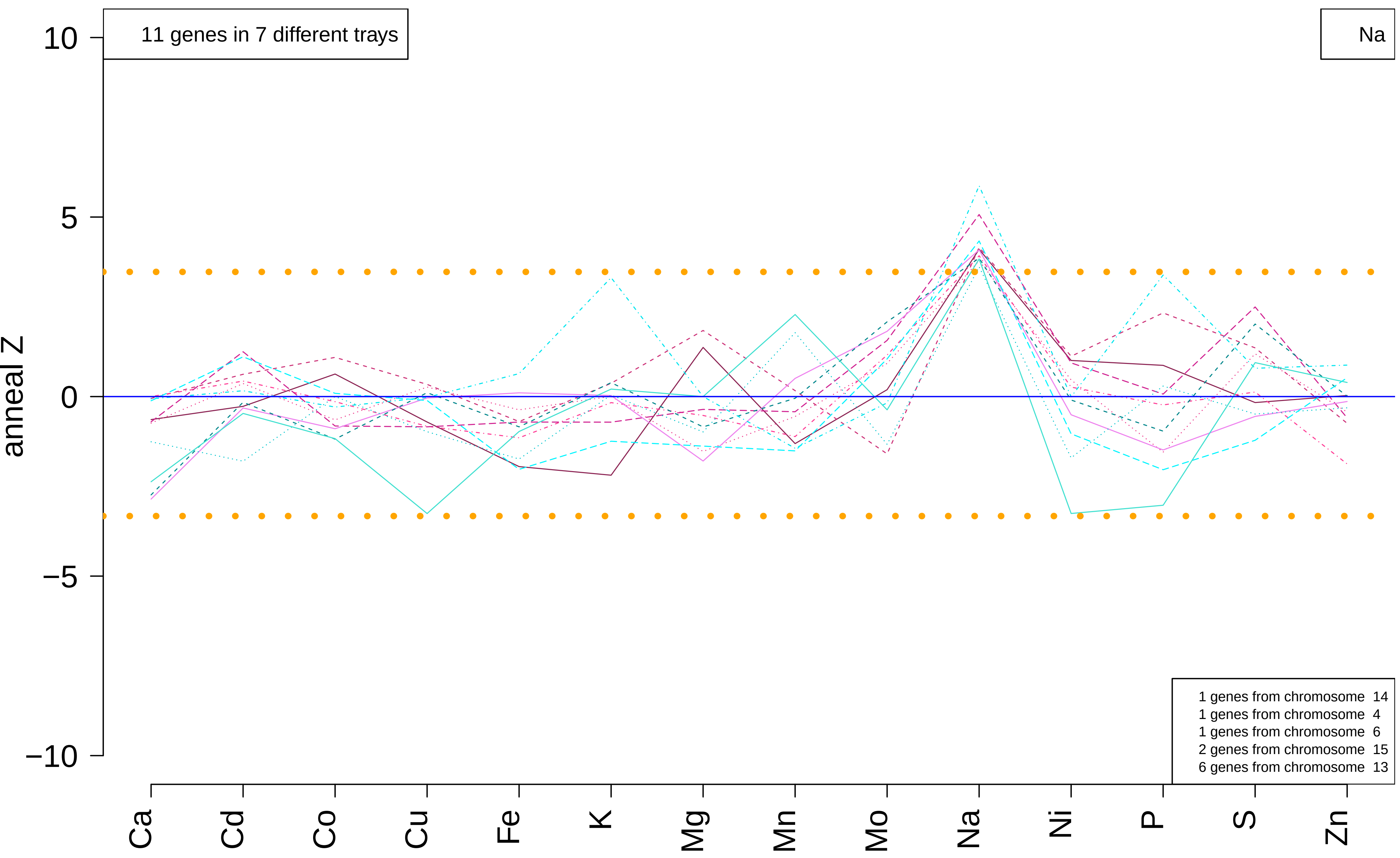

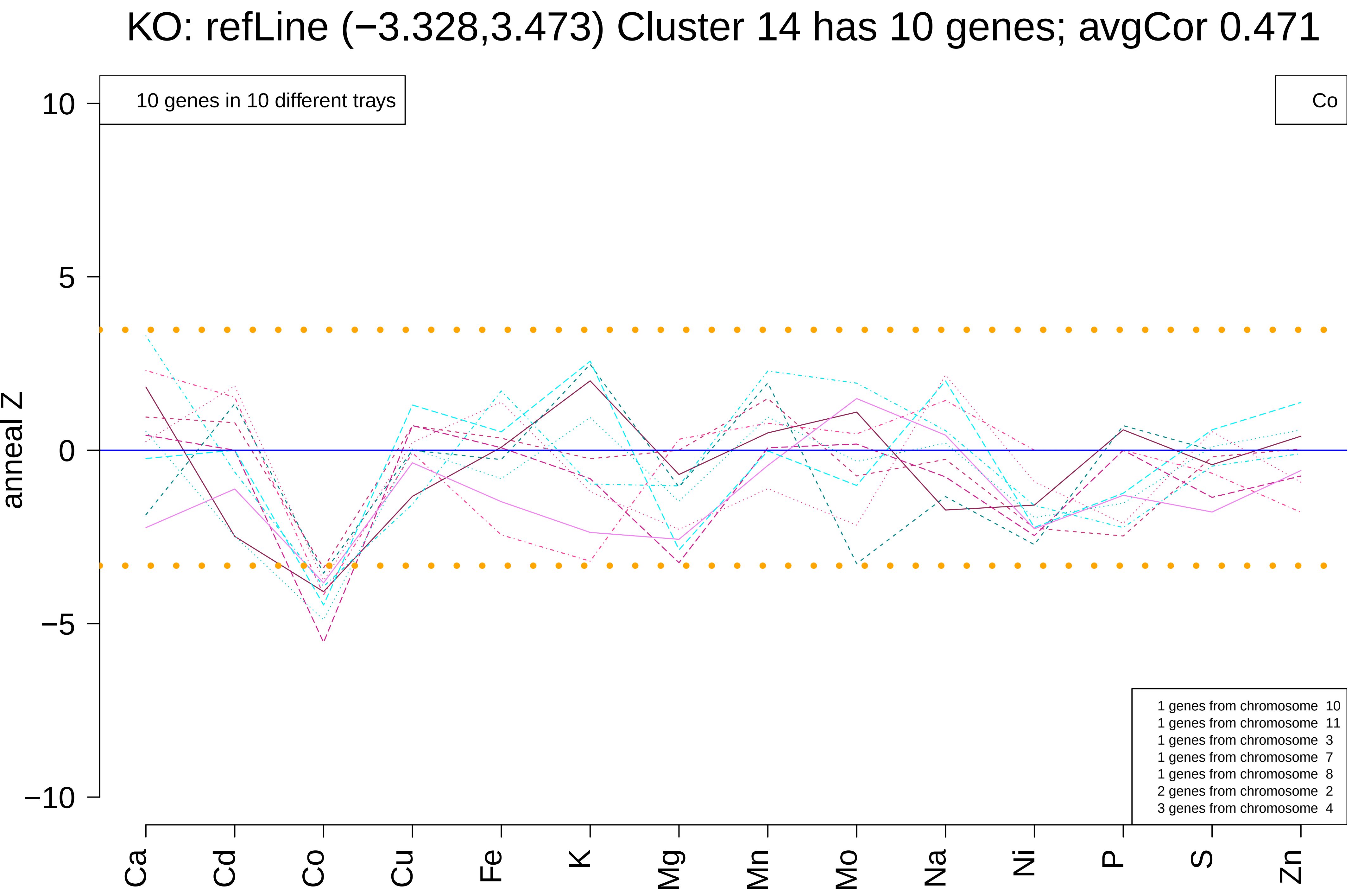
KO: refLine (−3.328,3.473) Cluster 14 has 10 genes; avgCor 0.471
10 genes in 10 different trays
Co
anneal Z
10
5
0
−5
−10
Ca
Cd
Co
Cu
Fe
K
Mg
Mn
Mo
Na
Ni
P
S
Zn
1 genes from chromosome 10
1 genes from chromosome 11
1 genes from chromosome 3
1 genes from chromosome 7
1 genes from chromosome 8
2 genes from chromosome 2
3 genes from chromosome 4

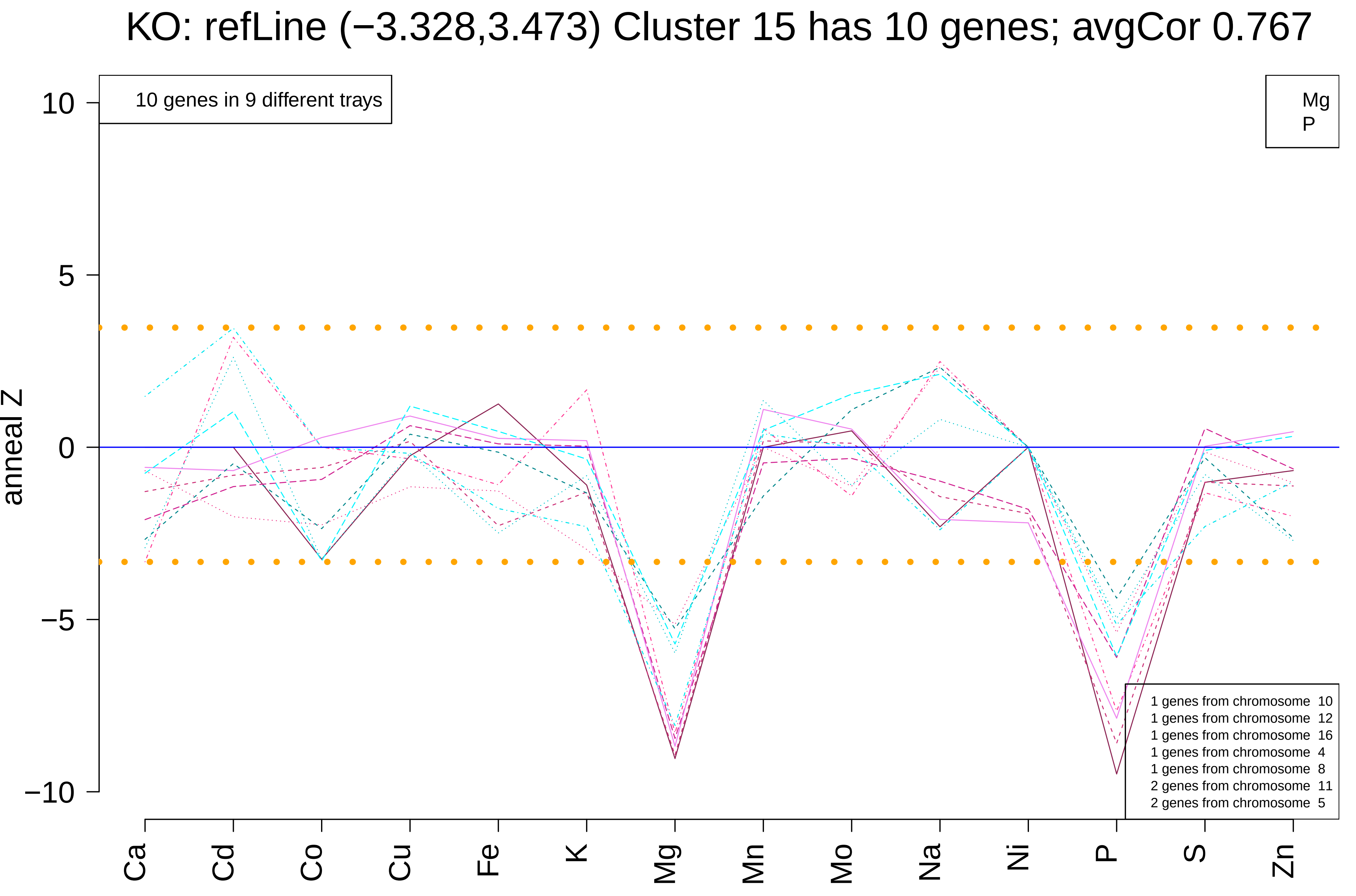

KO: refLine (−3.328,3.473) Cluster 15 has 10 genes; avgCor 0.767
10 genes in 9 different trays
Mg
P
anneal Z
10
5
0
−5
−10
Ca
Cd
Co
Cu
Fe
K
Mg
Mn
Mo
Na
Ni
P
S
Zn
1 genes from chromosome 10
1 genes from chromosome 12
1 genes from chromosome 16
1 genes from chromosome 4
1 genes from chromosome 8
2 genes from chromosome 11
2 genes from chromosome 5

KO: refLine (−3.328,3.473) Cluster 16 has 8 genes; avgCor 0.486

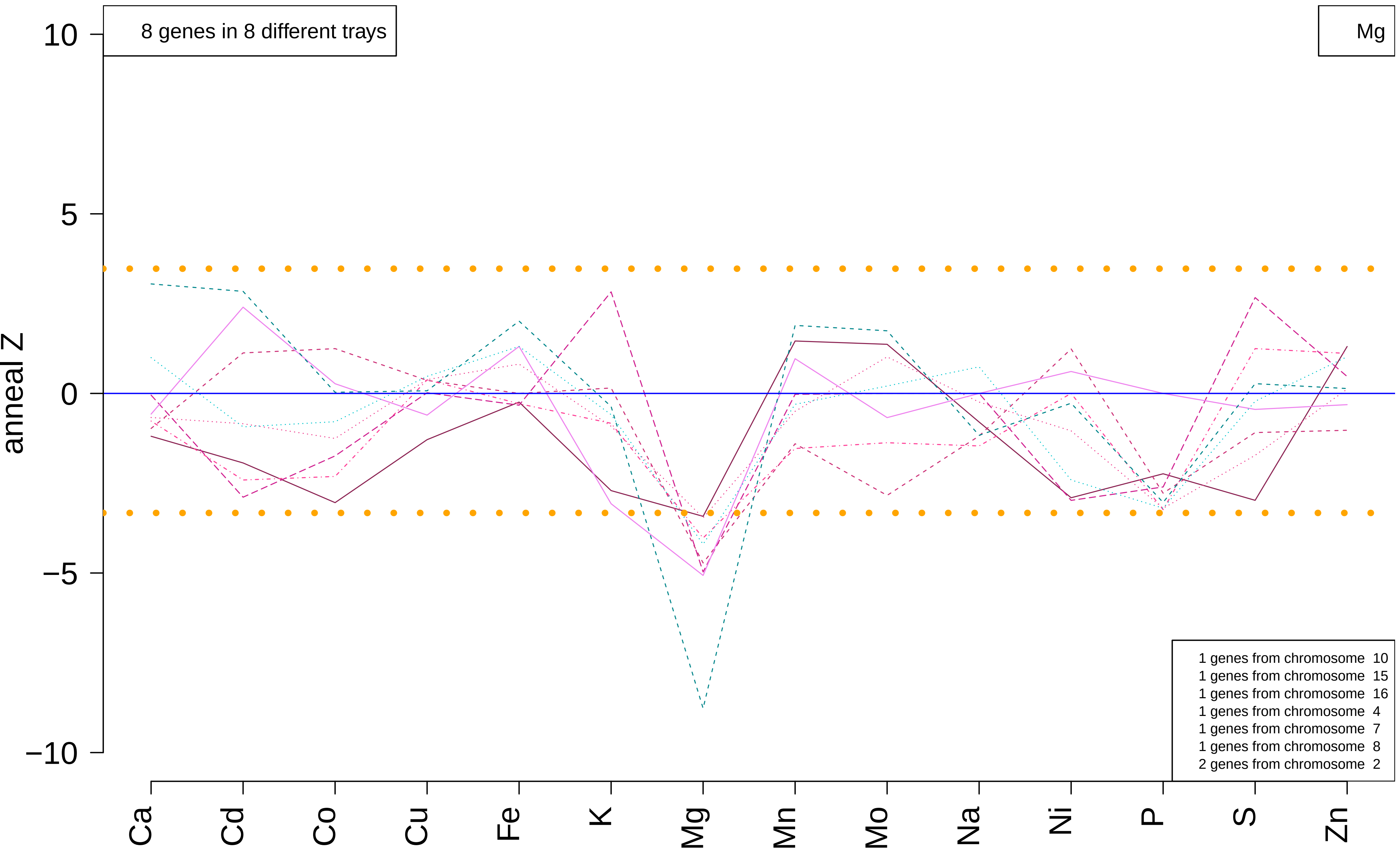

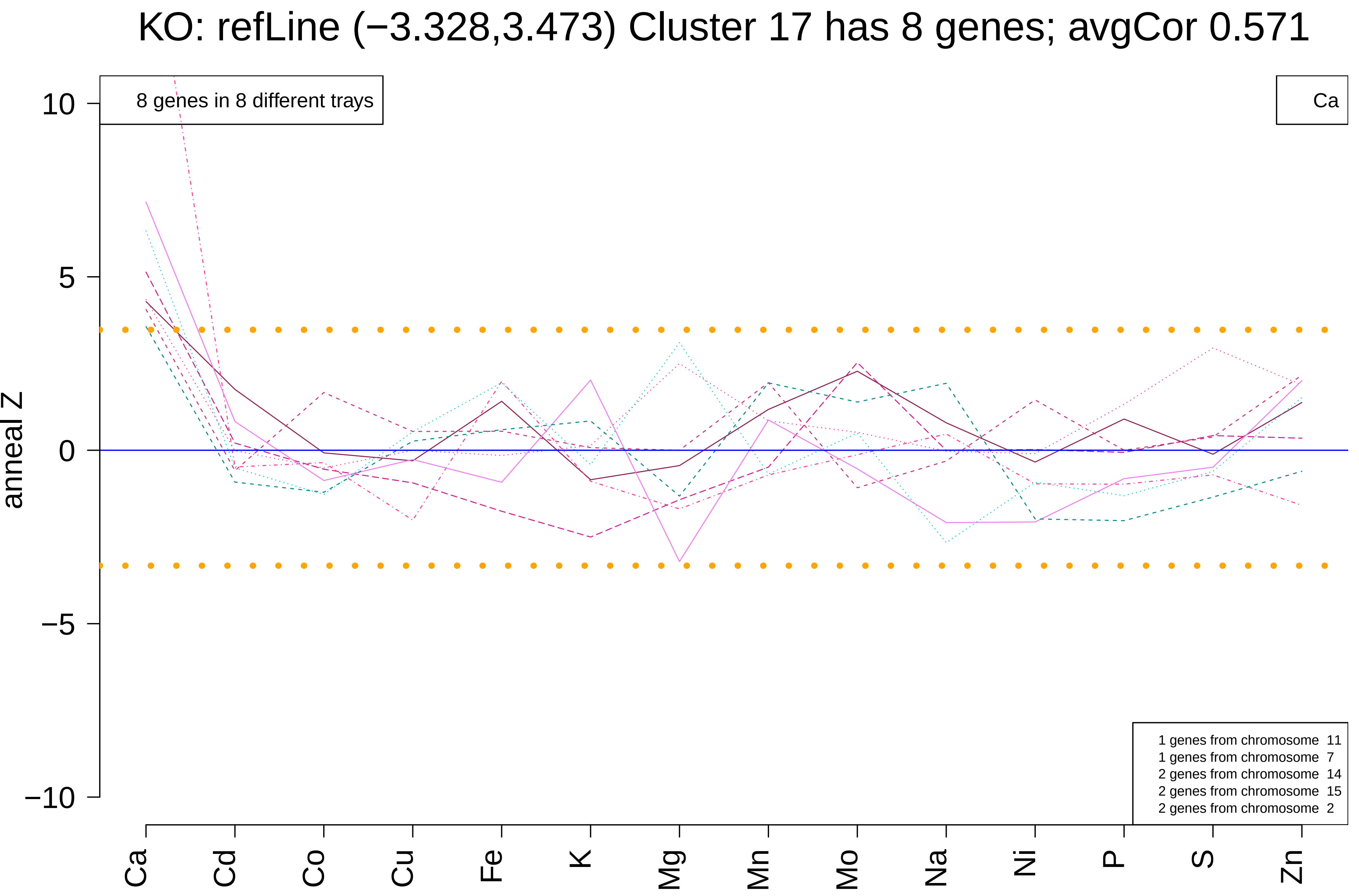

KO: refLine (−3.328,3.473) Cluster 17 has 8 genes; avgCor 0.571
8 genes in 8 different trays
Ca
anneal Z
10
5
0
−5
−10
1 genes from chromosome 11
1 genes from chromosome 7
2 genes from chromosome 14
2 genes from chromosome 15
2 genes from chromosome 2
Ca
Cd
Co
Cu
Fe
K
Mg
Mn
Mo
Na
Ni
P
S
Zn

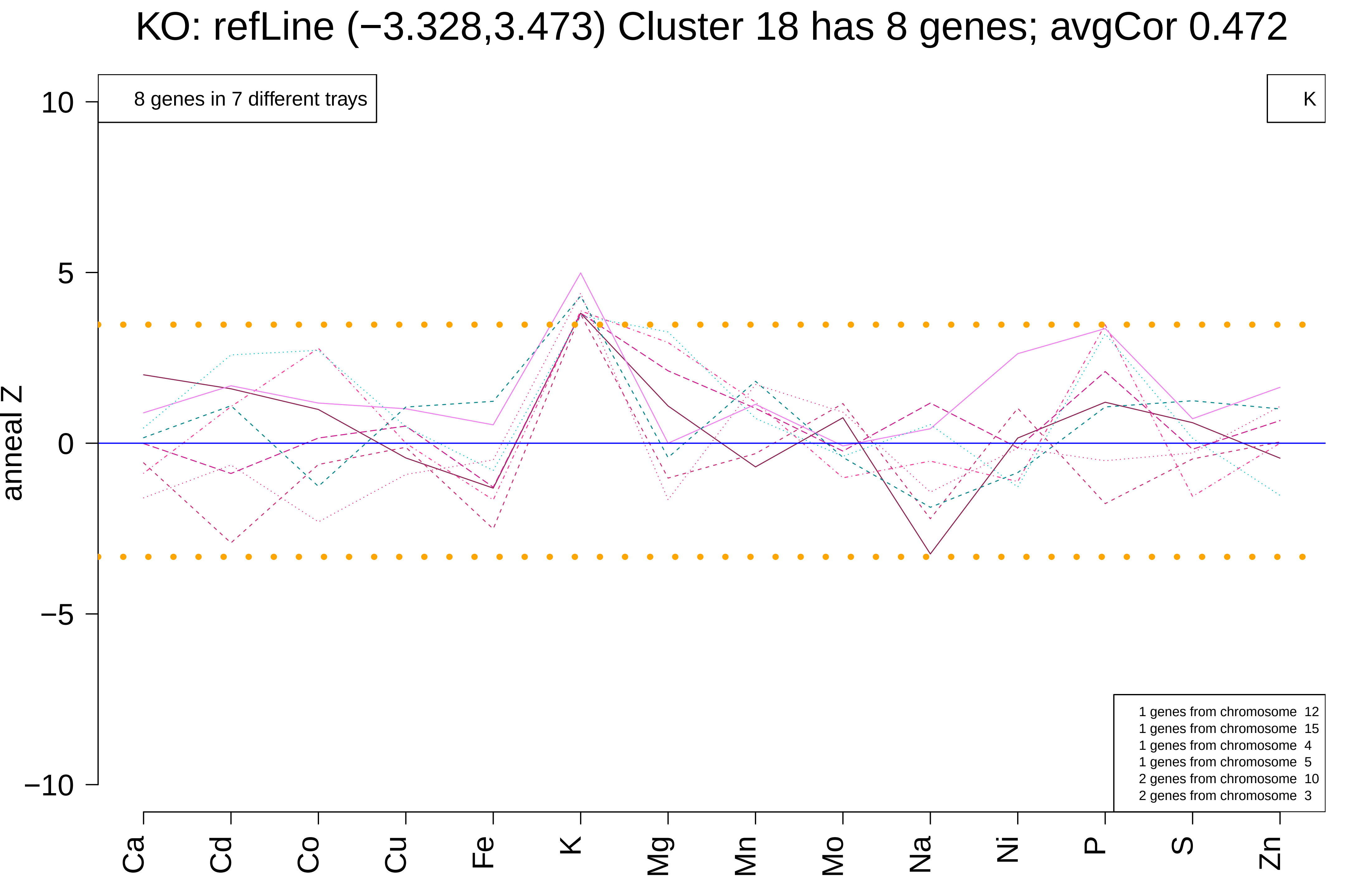
KO: refLine (−3.328,3.473) Cluster 18 has 8 genes; avgCor 0.472
8 genes in 7 different trays
K
anneal Z
10
5
0
−5
−10
Ca
Cd
Co
Cu
Fe
K
Mg
Mn
Mo
Na
Ni
P
S
Zn
1 genes from chromosome 12
1 genes from chromosome 15
1 genes from chromosome 4
1 genes from chromosome 5
2 genes from chromosome 10
2 genes from chromosome 3

KO: refLine (−3.328,3.473) Cluster 19 has 7 genes; avgCor 0.782

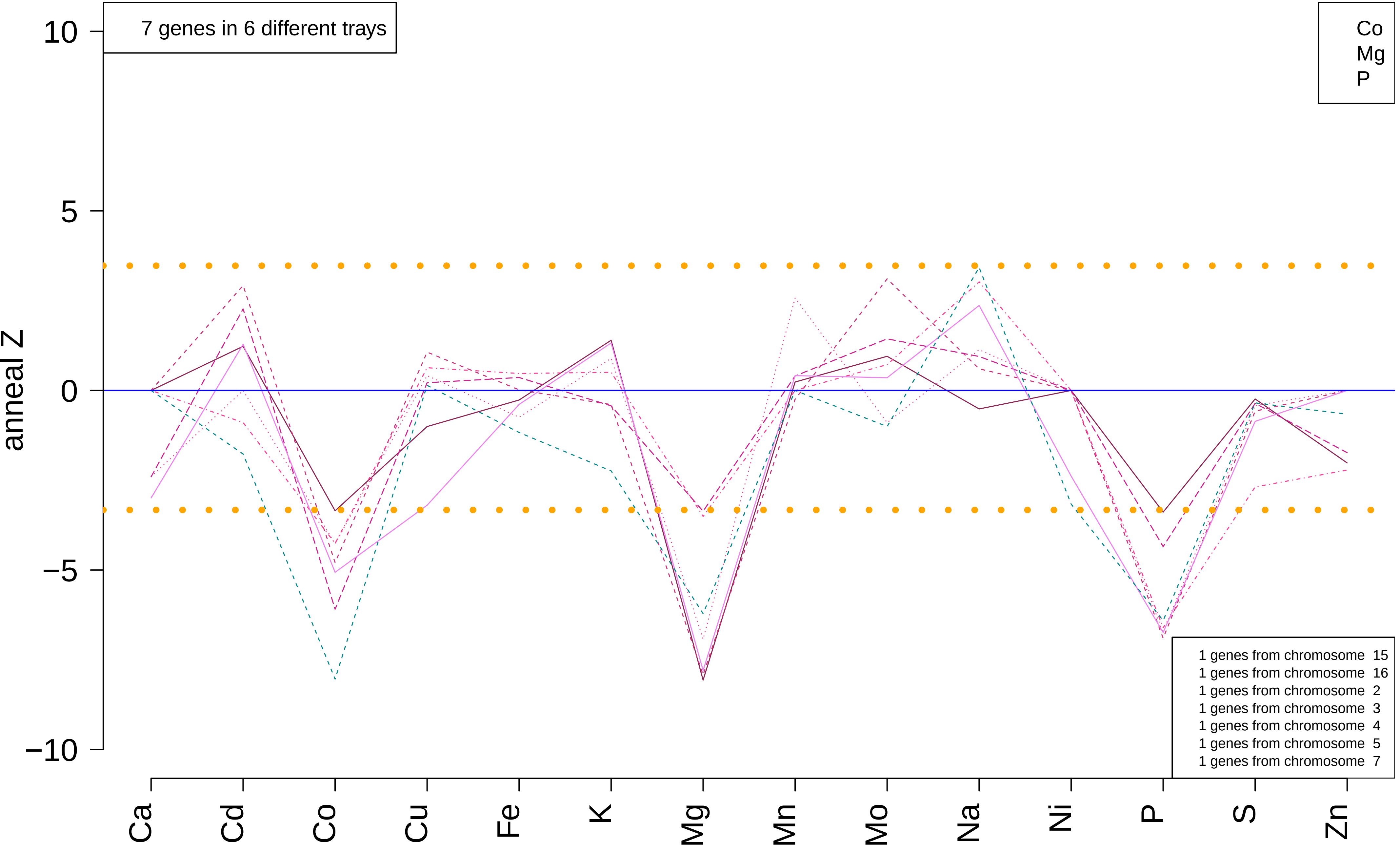

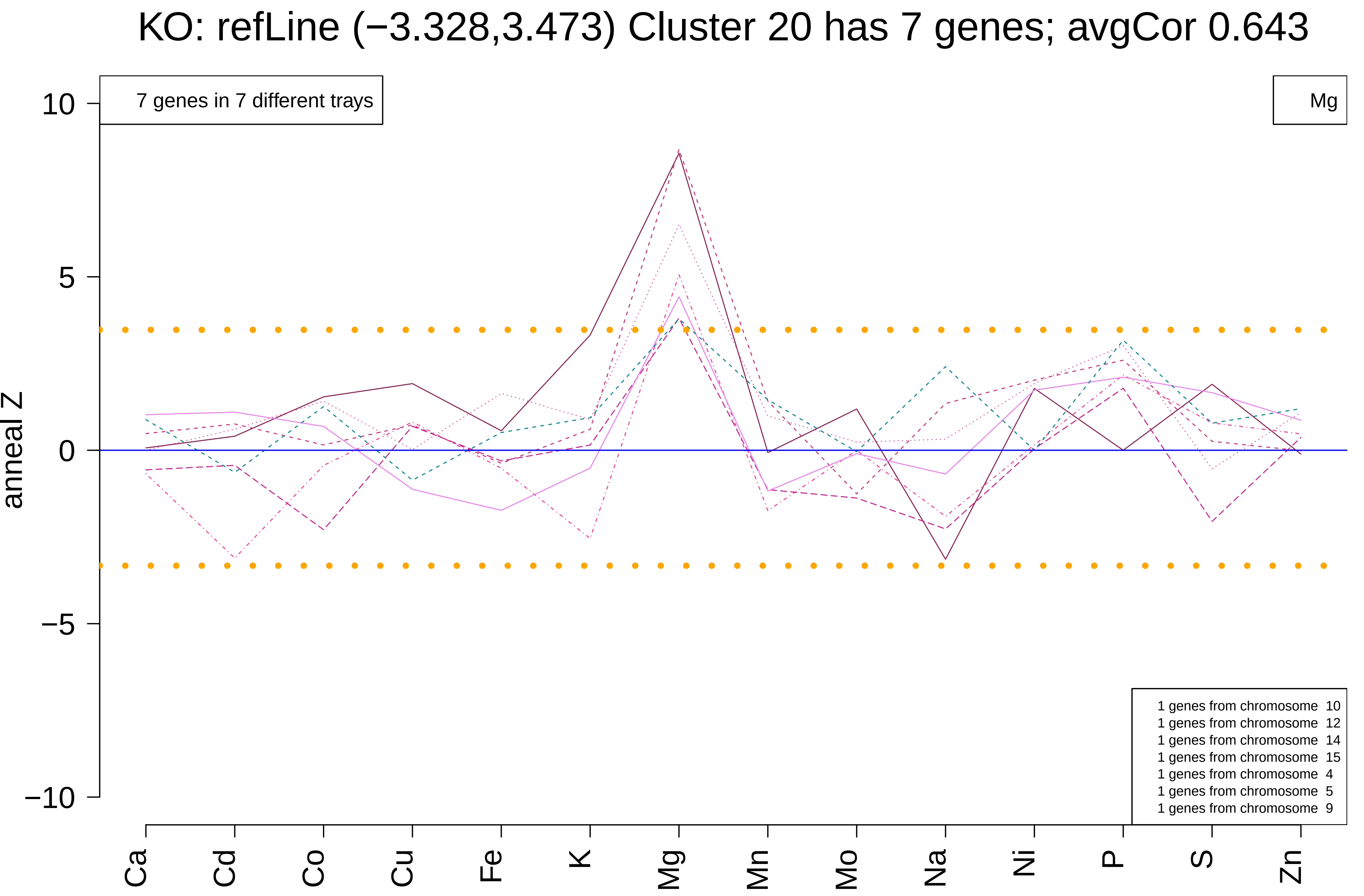
KO: refLine (−3.328,3.473) Cluster 20 has 7 genes; avgCor 0.643
7 genes in 7 different trays
Mg
anneal Z
10
5
0
−5
−10
Ca
Cd
Co
Cu
Fe
K
Mg
Mn
Mo
Na
Ni
P
S
Zn
1 genes from chromosome 10
1 genes from chromosome 12
1 genes from chromosome 14
1 genes from chromosome 15
1 genes from chromosome 4
1 genes from chromosome 5
1 genes from chromosome 9

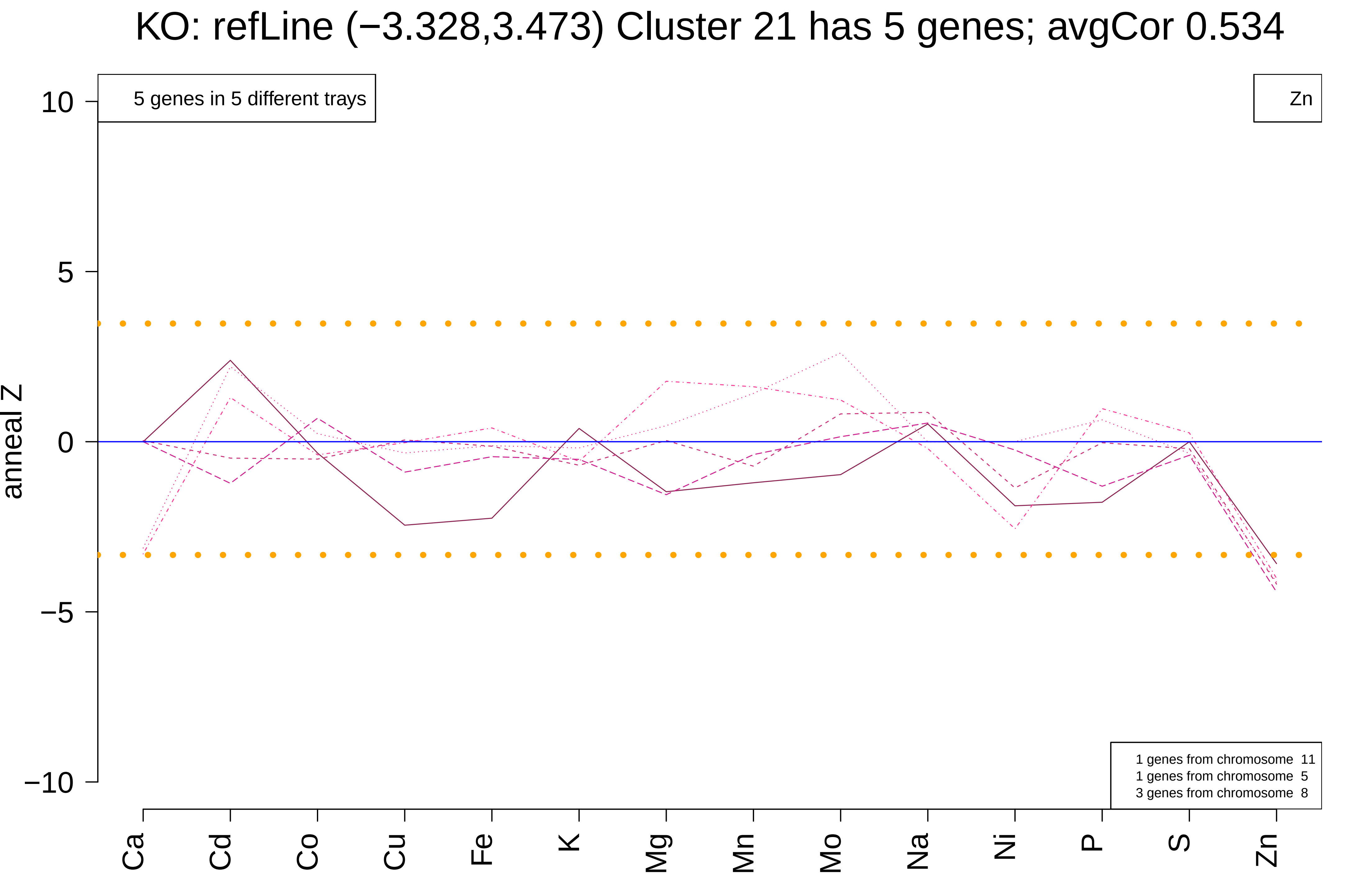
KO: refLine (−3.328,3.473) Cluster 21 has 5 genes; avgCor 0.534
5 genes in 5 different trays
Zn
anneal Z
10
5
0
−5
−10
Ca
Cd
Co
Cu
Fe
K
Mg
Mn
Mo
Na
Ni
P
S
Zn
1 genes from chromosome 11
1 genes from chromosome 5
3 genes from chromosome 8

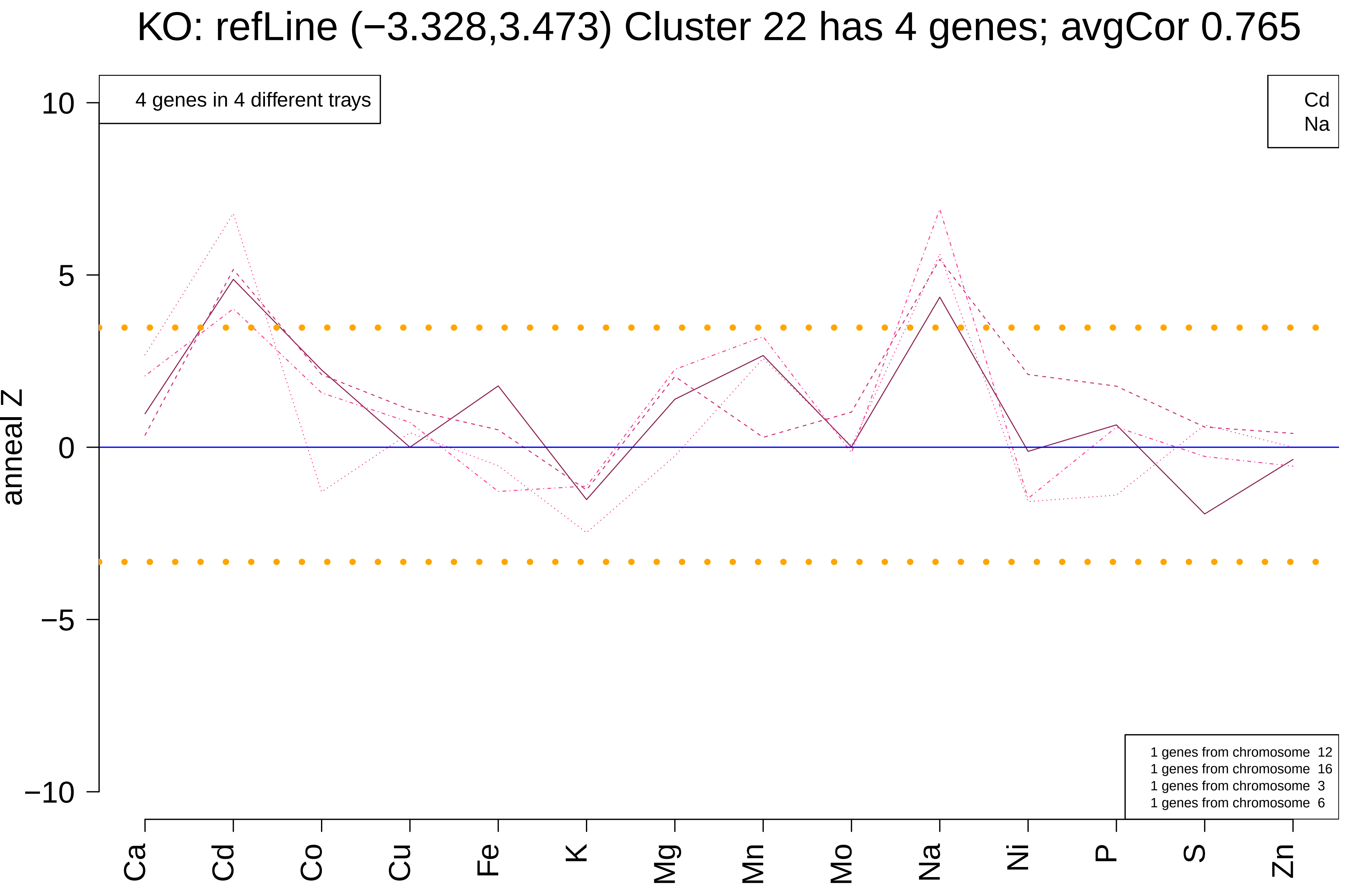
KO: refLine (−3.328,3.473) Cluster 22 has 4 genes; avgCor 0.765
4 genes in 4 different trays
Cd
Na
anneal Z
10
5
0
−5
−10
1 genes from chromosome 12
1 genes from chromosome 16
1 genes from chromosome 3
1 genes from chromosome 6
Ca
Cd
Co
Cu
Fe
K
Mg
Mn
Mo
Na
Ni
P
S
Zn

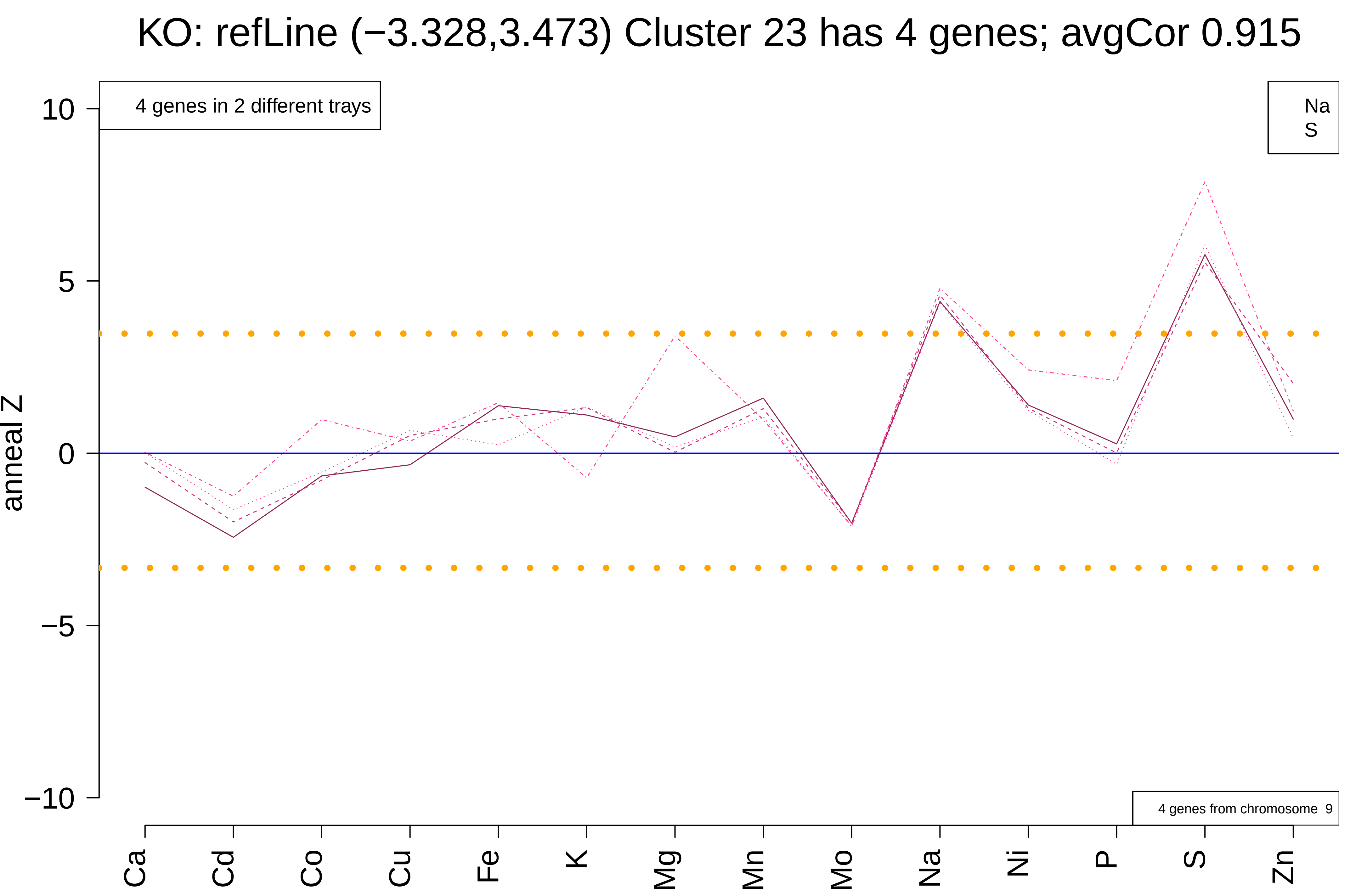
KO: refLine (−3.328,3.473) Cluster 23 has 4 genes; avgCor 0.915
4 genes in 2 different trays
Na
S
anneal Z
10
5
0
−5
−10
4 genes from chromosome 9
Ca
Cd
Co
Cu
Fe
K
Mg
Mn
Mo
Na
Ni
P
S
Zn

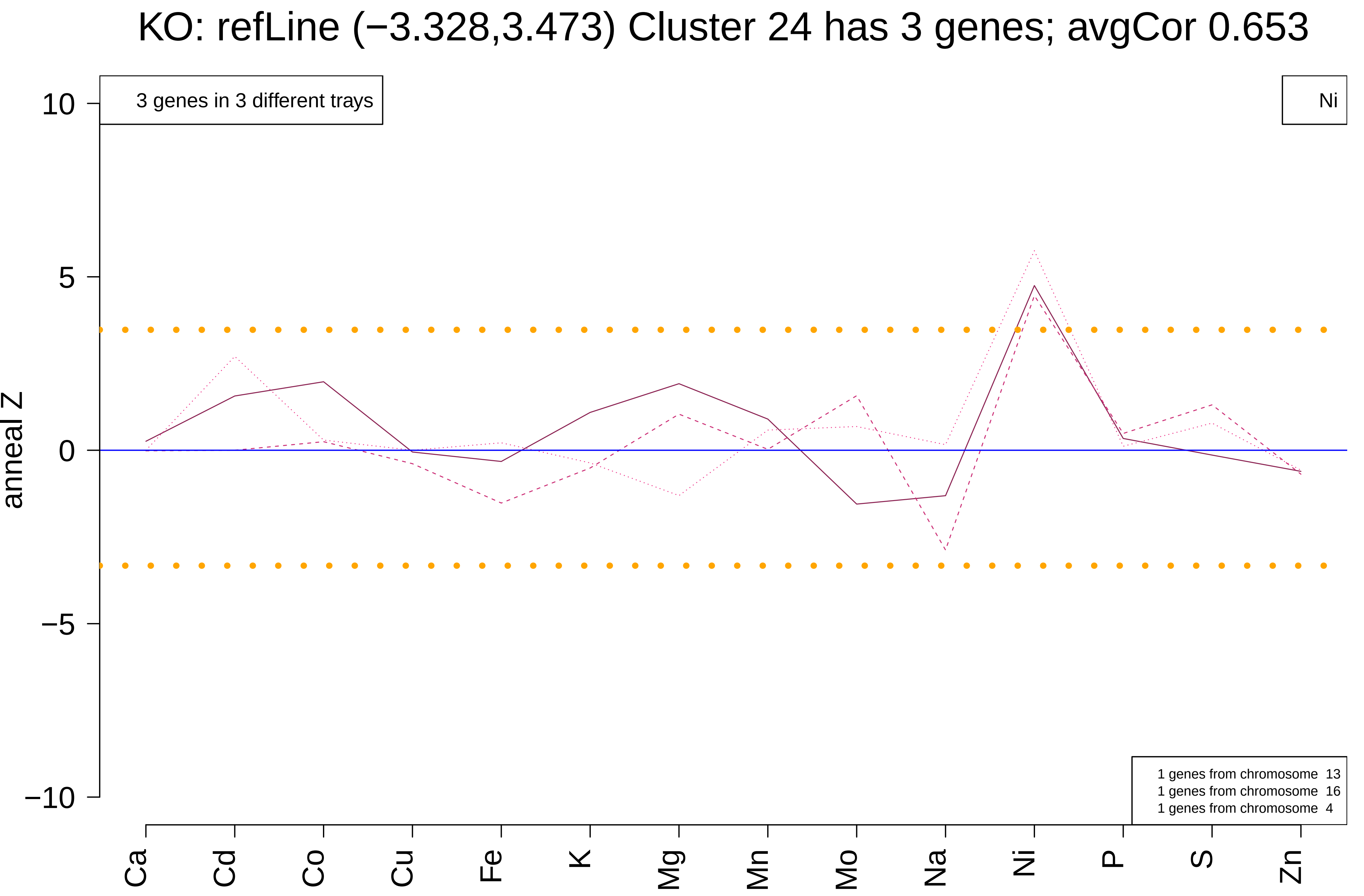
KO: refLine (−3.328,3.473) Cluster 24 has 3 genes; avgCor 0.653
3 genes in 3 different trays
Ni
anneal Z
10
5
0
−5
−10
1 genes from chromosome 13
1 genes from chromosome 16
1 genes from chromosome 4
Ca
Cd
Co
Cu
Fe
K
Mg
Mn
Mo
Na
Ni
P
S
Zn

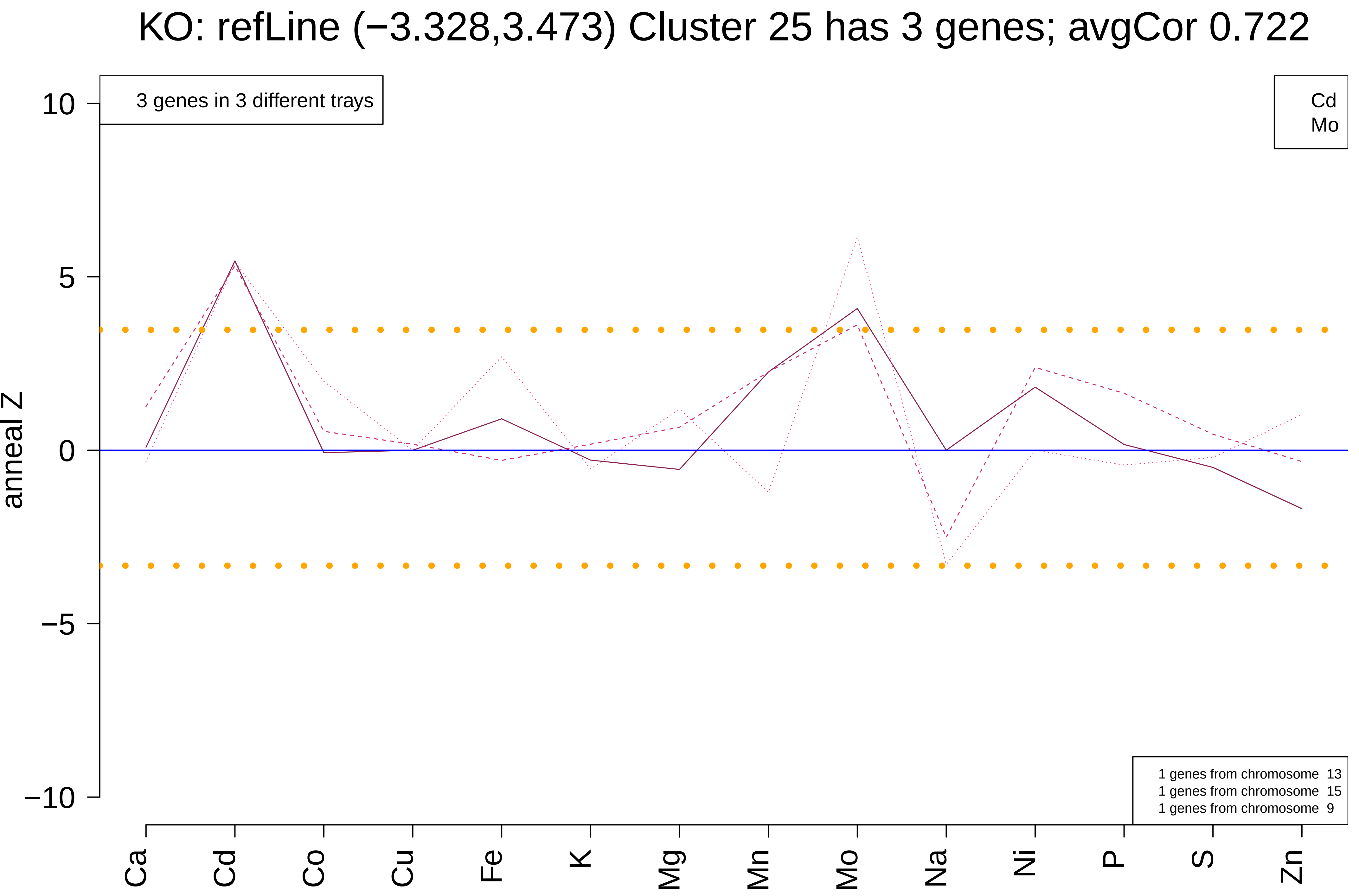
KO: refLine (−3.328,3.473) Cluster 25 has 3 genes; avgCor 0.722
3 genes in 3 different trays
Cd
Mo
anneal Z
10
5
0
−5
−10
1 genes from chromosome 13
1 genes from chromosome 15
1 genes from chromosome 9
Ca
Cd
Co
Cu
Fe
K
Mg
Mn
Mo
Na
Ni
P
S
Zn

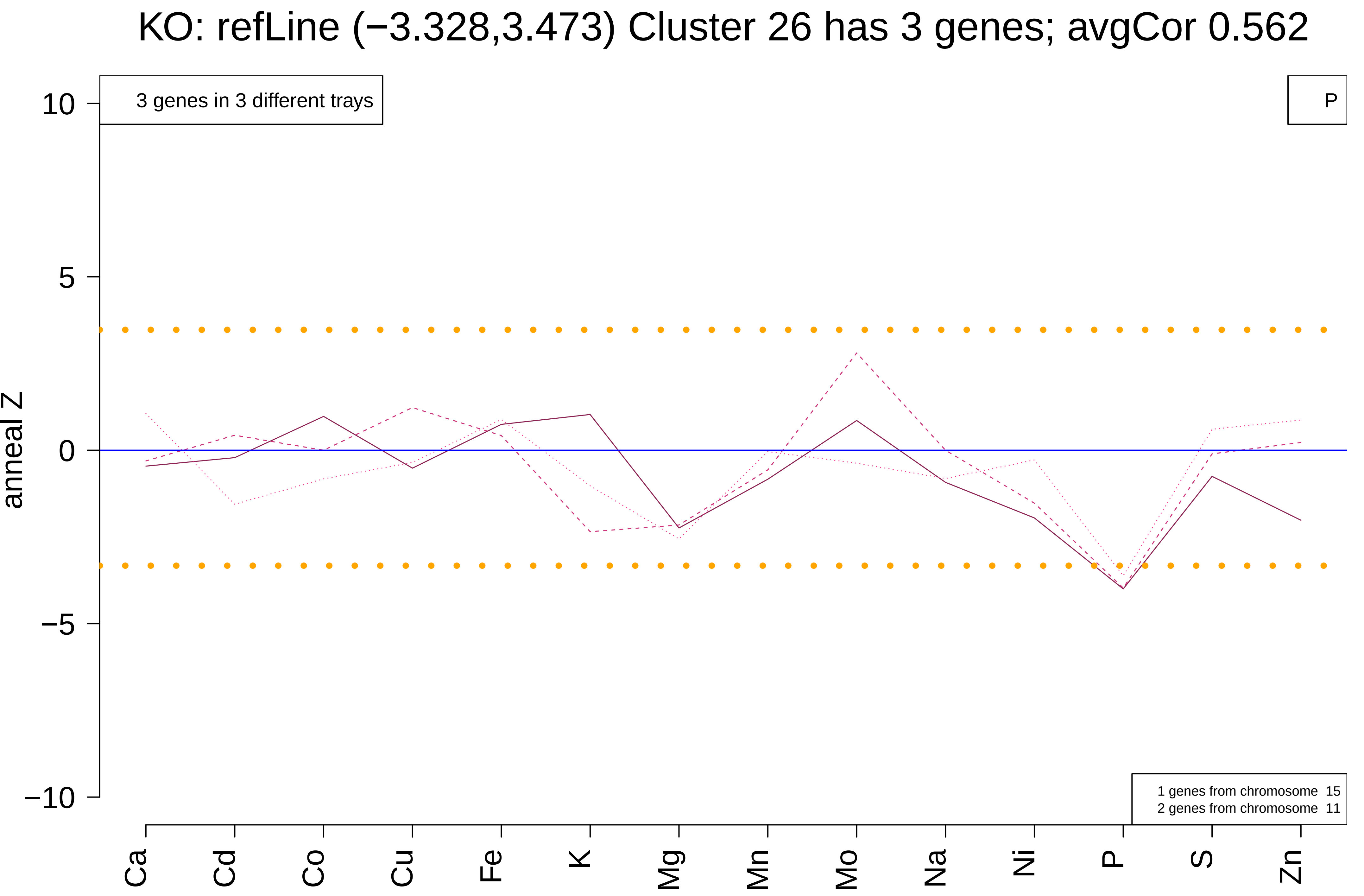
KO: refLine (−3.328,3.473) Cluster 26 has 3 genes; avgCor 0.562
3 genes in 3 different trays
P
anneal Z
10
5
0
−5
−10
Ca
Cd
Co
Cu
Fe
K
Mg
Mn
Mo
Na
Ni
P
S
Zn
1 genes from chromosome 15
2 genes from chromosome 11
