# Supplementary material for: High-resolution genome-wide scan of genes, gene-networks and cellular systems impacting the yeast ionome
Source: BMC Genomics. 2012 Nov 14;13:623. doi: 10.1186/1471-2164-13-623 (PMC3652779; doi:10.1186/1471-2164-13-623)
Supplement: Additional file 4: Figure S3 — Clusters of ionomic profiles using the exhaustive significance clustering (ESC) method for the KO (A), KOd (B) and OE (C) data sets of genes that have a significant impact on the ionome. The X-axis represents the elements used in the clustering and the Y-axis represents the moderated Z values used for each element. Only the genes that significantly affect at least one element and pass the annealing process are included, and only the clusters that include at least 3 genes are shown. [file 1471-2164-13-623-S4.zip › Figure S3B.pdf]

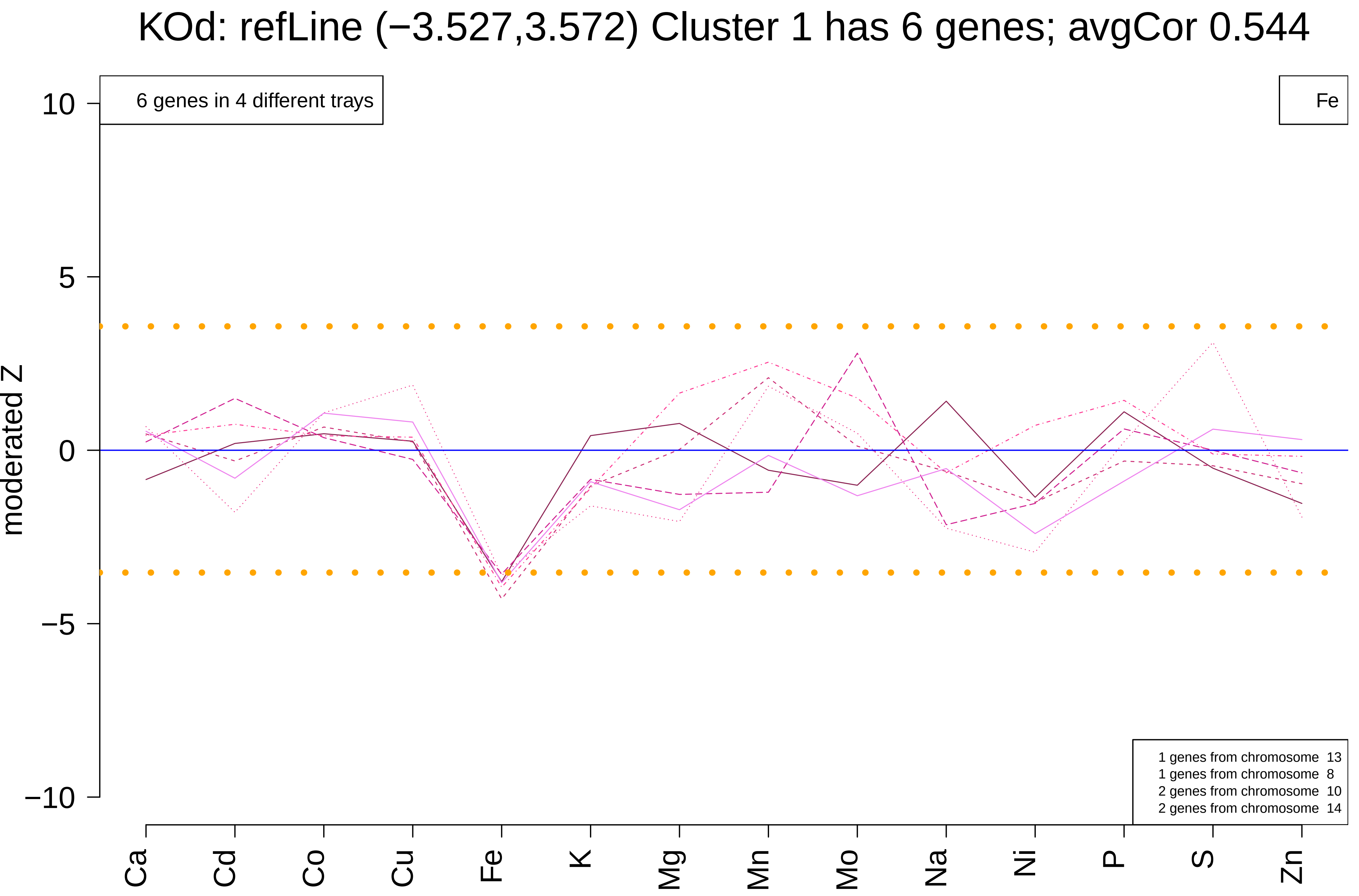
KOd: refLine (−3.527,3.572) Cluster 1 has 6 genes; avgCor 0.544
6 genes in 4 different trays
Fe
moderated Z
10
5
0
−5
−10
Ca
Cd
Co
Cu
Fe
K
Mg
Mn
Mo
Na
Ni
P
S
Zn
1 genes from chromosome 13
1 genes from chromosome 8
2 genes from chromosome 10
2 genes from chromosome 14

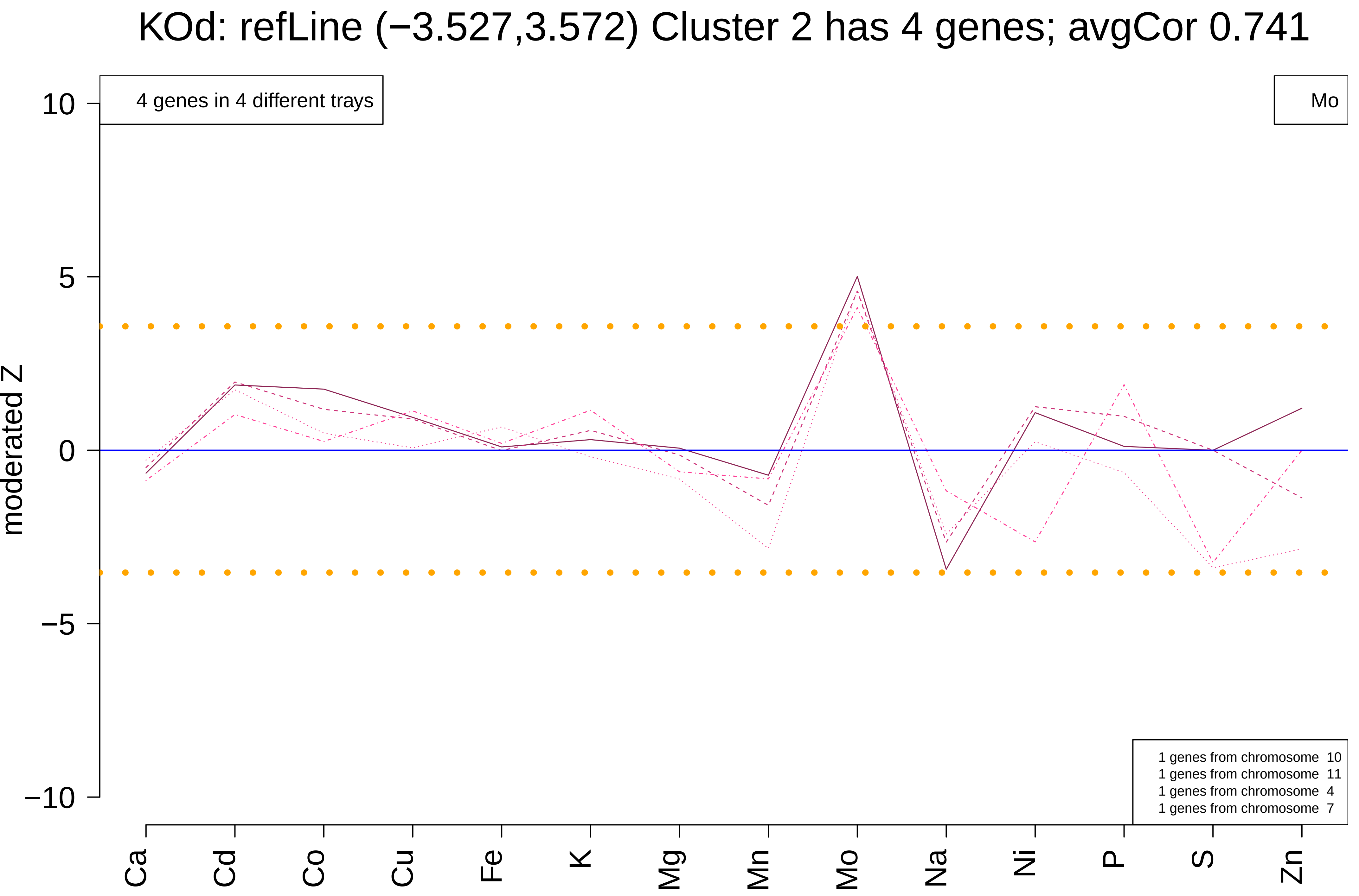
KOd: refLine (−3.527,3.572) Cluster 2 has 4 genes; avgCor 0.741
4 genes in 4 different trays
Mo
moderated Z
10
5
0
−5
−10
Ca
Cd
Co
Cu
Fe
K
Mg
Mn
Mo
Na
Ni
P
S
Zn
1 genes from chromosome 10
1 genes from chromosome 11
1 genes from chromosome 4
1 genes from chromosome 7

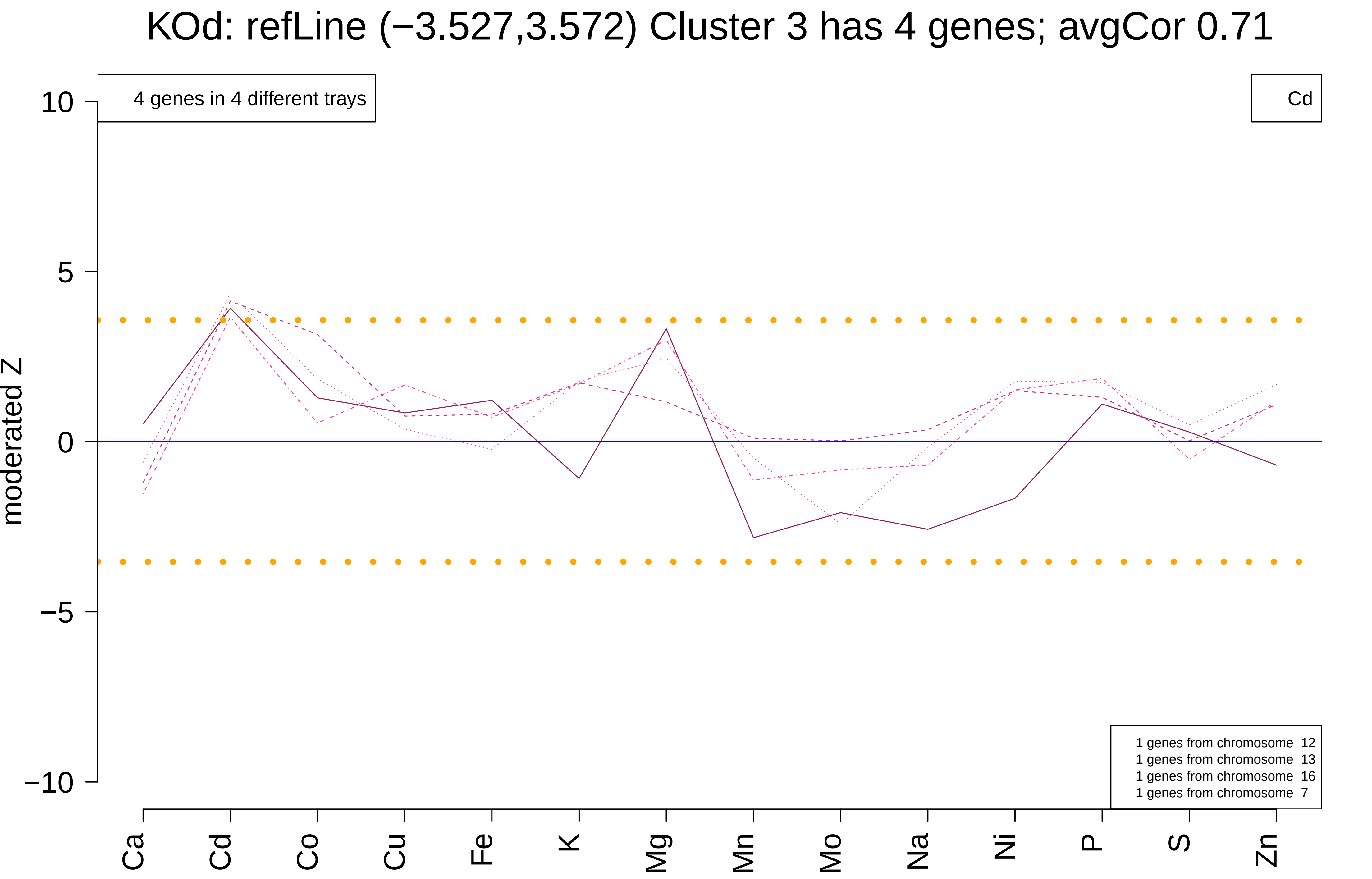
KOd: refLine (−3.527,3.572) Cluster 3 has 4 genes; avgCor 0.71
4 genes in 4 different trays
Cd
moderated Z
10
5
0
−5
−10
1 genes from chromosome 12
1 genes from chromosome 13
1 genes from chromosome 16
1 genes from chromosome 7
Ca
Cd
Co
Cu
Fe
K
Mg
Mn
Mo
Na
Ni
P
S
Zn

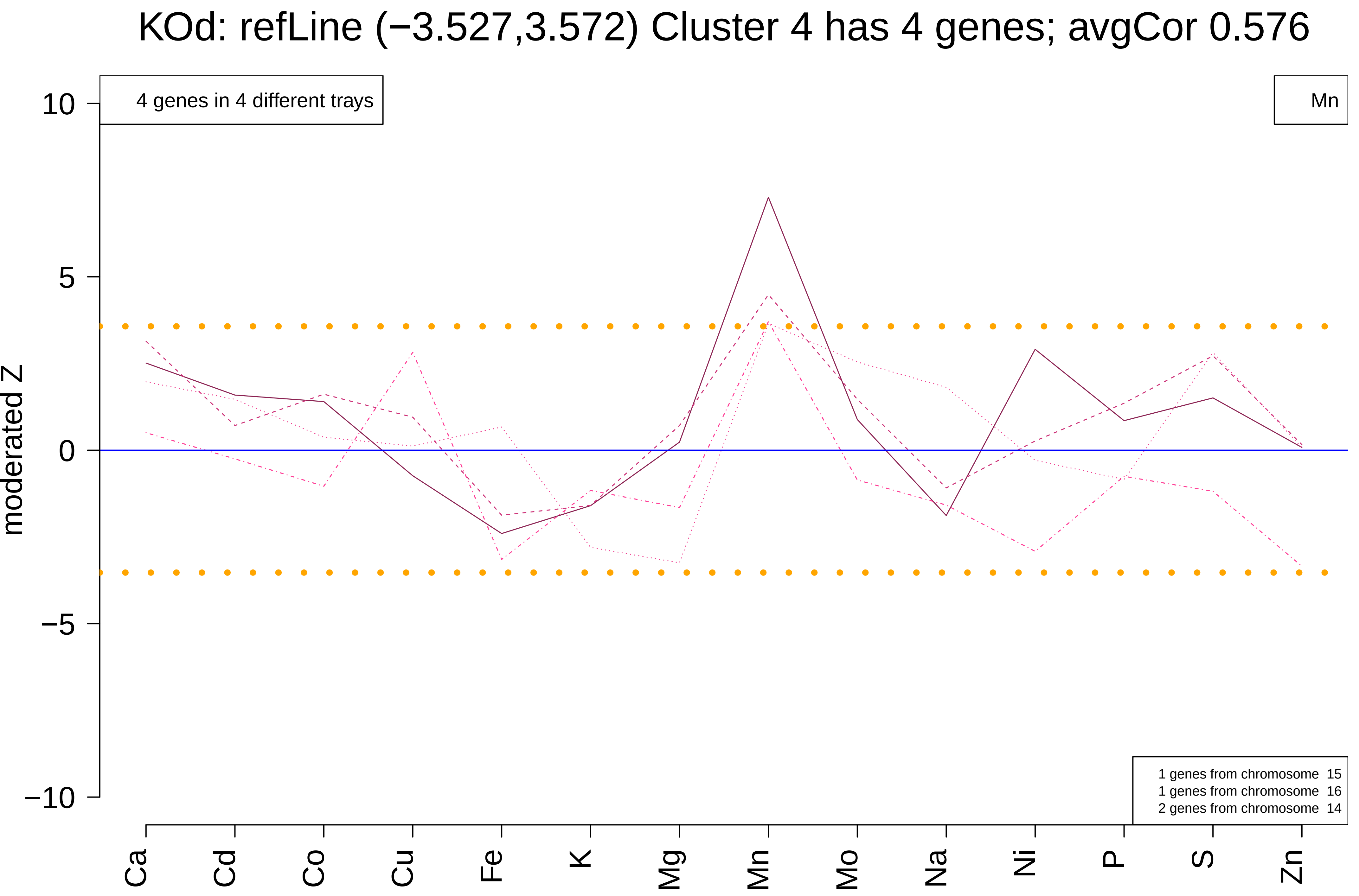
KOd: refLine (−3.527,3.572) Cluster 4 has 4 genes; avgCor 0.576
4 genes in 4 different trays
Mn
moderated Z
10
5
0
−5
−10
1 genes from chromosome 15
1 genes from chromosome 16
2 genes from chromosome 14
Ca
Cd
Co
Cu
Fe
K
Mg
Mn
Mo
Na
Ni
P
S
Zn

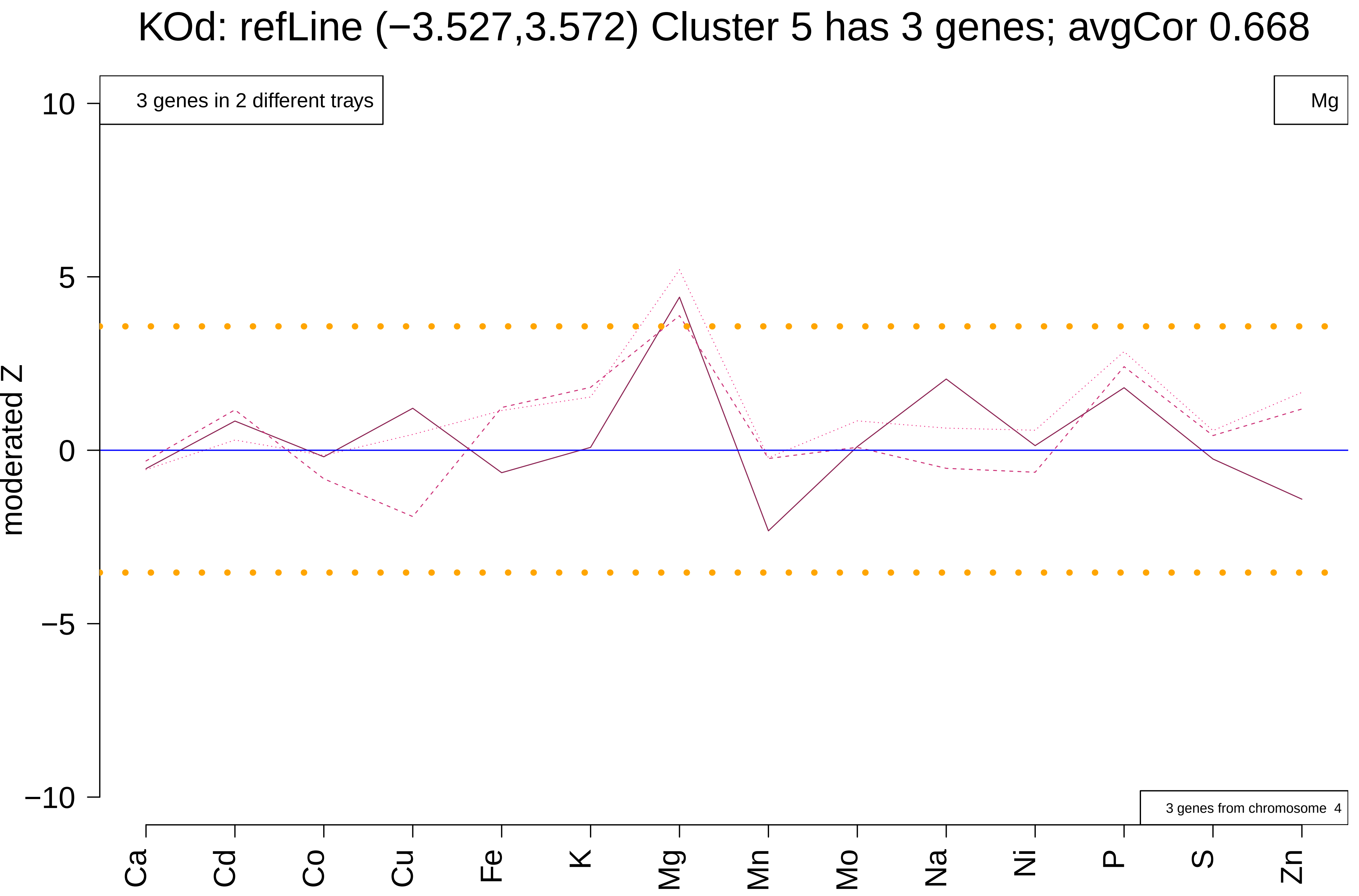

KOd: refLine (−3.527,3.572) Cluster 5 has 3 genes; avgCor 0.668
3 genes in 2 different trays
Mg
moderated Z
10
5
0
−5
−10
3 genes from chromosome 4
Ca
Cd
Co
Cu
Fe
K
Mg
Mn
Mo
Na
Ni
P
S
Zn
