# Supplementary material for: High-resolution genome-wide scan of genes, gene-networks and cellular systems impacting the yeast ionome
Source: BMC Genomics. 2012 Nov 14;13:623. doi: 10.1186/1471-2164-13-623 (PMC3652779; doi:10.1186/1471-2164-13-623)
Supplement: Additional file 4: Figure S3 — Clusters of ionomic profiles using the exhaustive significance clustering (ESC) method for the KO (A), KOd (B) and OE (C) data sets of genes that have a significant impact on the ionome. The X-axis represents the elements used in the clustering and the Y-axis represents the moderated Z values used for each element. Only the genes that significantly affect at least one element and pass the annealing process are included, and only the clusters that include at least 3 genes are shown. [file 1471-2164-13-623-S4.zip › Figure S3C.pdf]

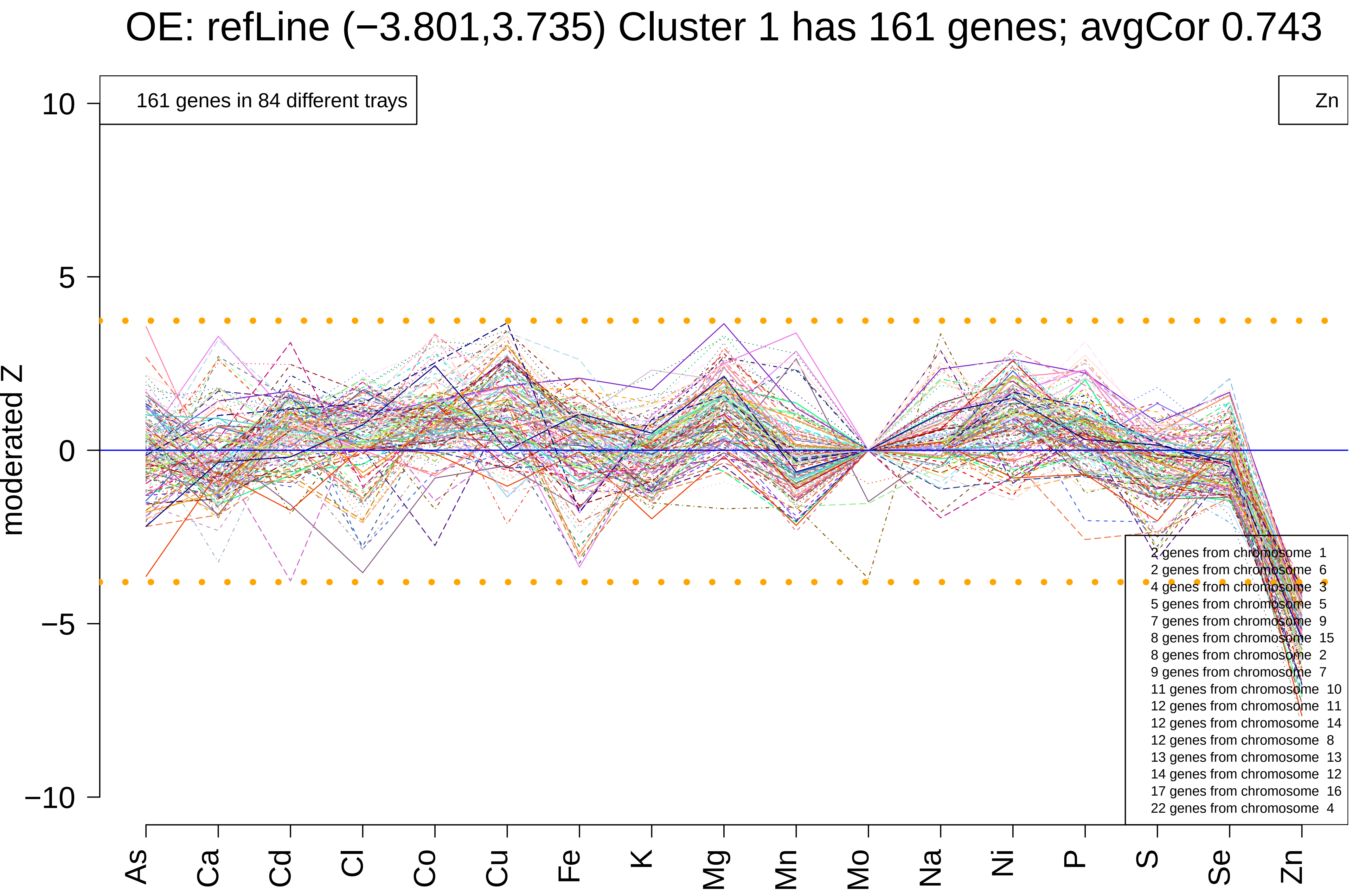

OE: refLine (−3.801,3.735) Cluster 1 has 161 genes; avgCor 0.743
161 genes in 84 different trays
Zn
moderated Z
10
5
0
−5
−10
As
Ca
Cd
Cl
Co
Cu
Fe
K
Mg
Mn
Mo
Na
Ni
P
S
Se
Zn
2 genes from chromosome 1
2 genes from chromosome 6
4 genes from chromosome 3
5 genes from chromosome 5
7 genes from chromosome 9
8 genes from chromosome 15
8 genes from chromosome 2
9 genes from chromosome 7
11 genes from chromosome 10
12 genes from chromosome 11
12 genes from chromosome 14
12 genes from chromosome 8
13 genes from chromosome 13
14 genes from chromosome 12
17 genes from chromosome 16
22 genes from chromosome 4

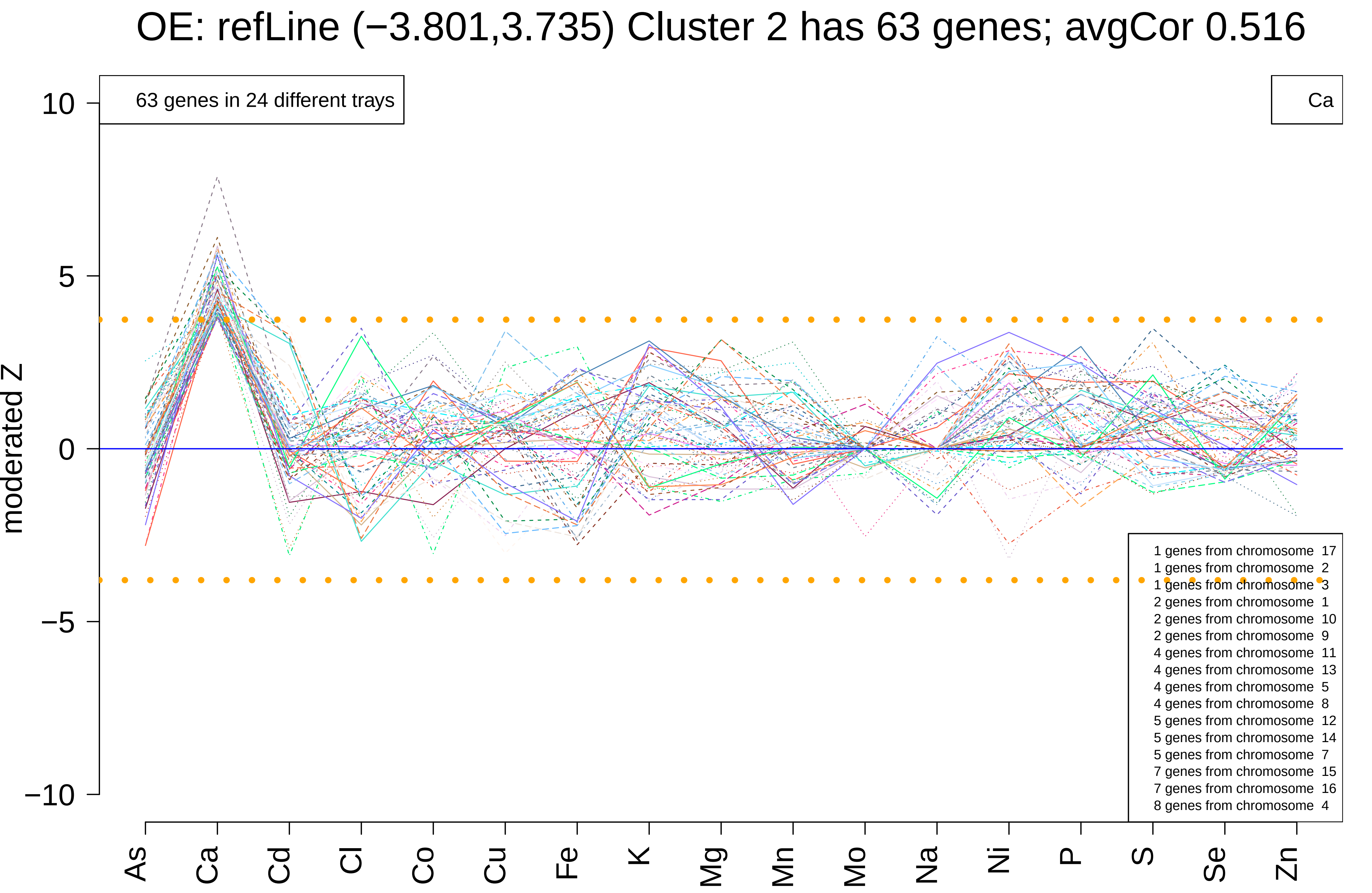

OE: refLine (−3.801,3.735) Cluster 2 has 63 genes; avgCor 0.516
63 genes in 24 different trays
Ca
moderated Z
10
5
0
−5
−10
As
Ca
Cd
Cl
Co
Cu
Fe
K
Mg
Mn
Mo
Na
Ni
P
S
Se
Zn
1 genes from chromosome 17
1 genes from chromosome 2
1 genes from chromosome 3
2 genes from chromosome 1
2 genes from chromosome 10
2 genes from chromosome 9
4 genes from chromosome 11
4 genes from chromosome 13
4 genes from chromosome 5
4 genes from chromosome 8
5 genes from chromosome 12
5 genes from chromosome 14
5 genes from chromosome 7
7 genes from chromosome 15
7 genes from chromosome 16
8 genes from chromosome 4

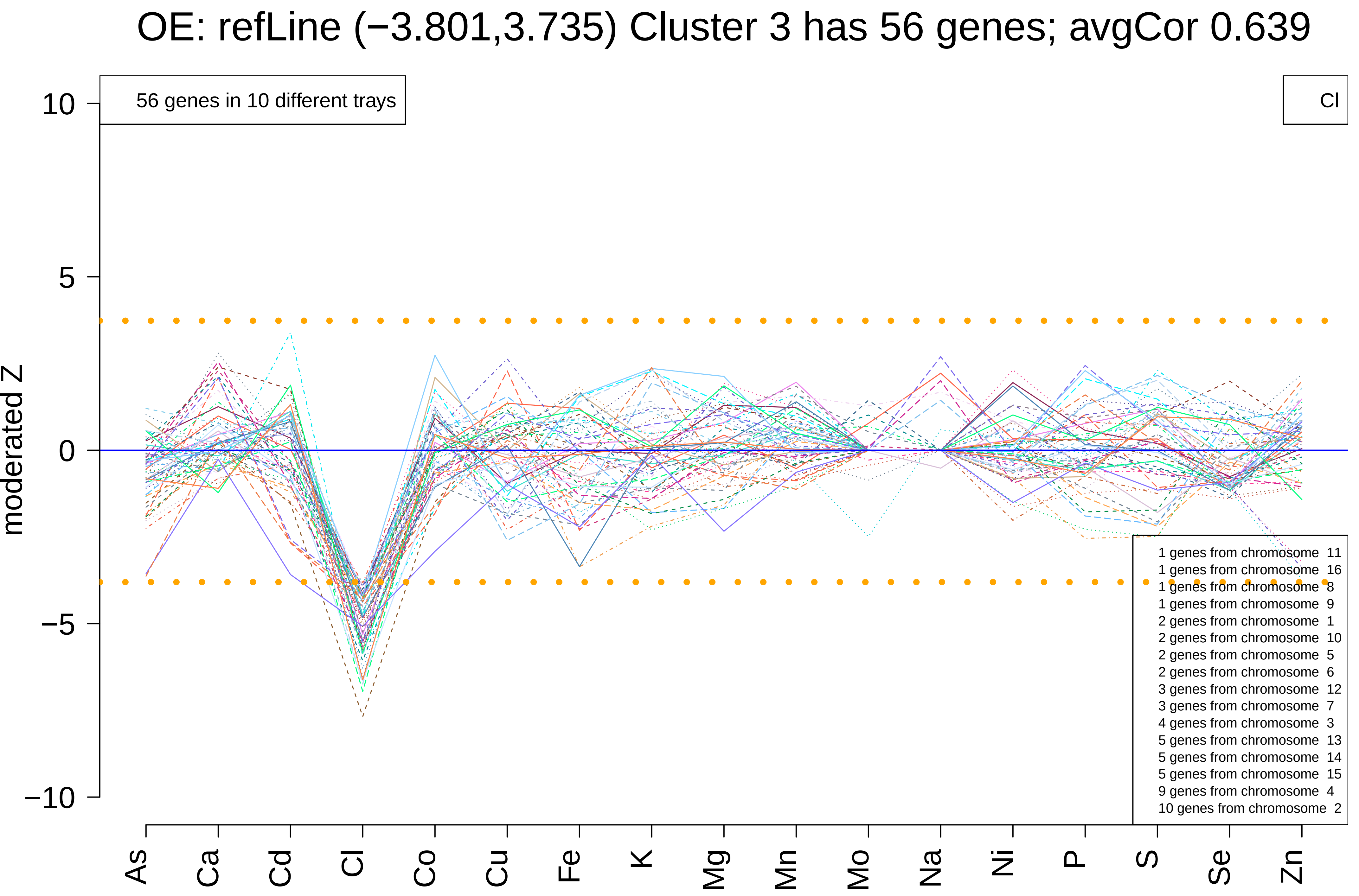

OE: refLine (−3.801,3.735) Cluster 3 has 56 genes; avgCor 0.639
56 genes in 10 different trays
Cl
moderated Z
10
5
0
−5
−10
As
Ca
Cd
Cl
Co
Cu
Fe
K
Mg
Mn
Mo
Na
Ni
P
S
Se
Zn
1 genes from chromosome 11
1 genes from chromosome 16
1 genes from chromosome 8
1 genes from chromosome 9
2 genes from chromosome 1
2 genes from chromosome 10
2 genes from chromosome 5
2 genes from chromosome 6
3 genes from chromosome 12
3 genes from chromosome 7
4 genes from chromosome 3
5 genes from chromosome 13
5 genes from chromosome 14
5 genes from chromosome 15
9 genes from chromosome 4
10 genes from chromosome 2

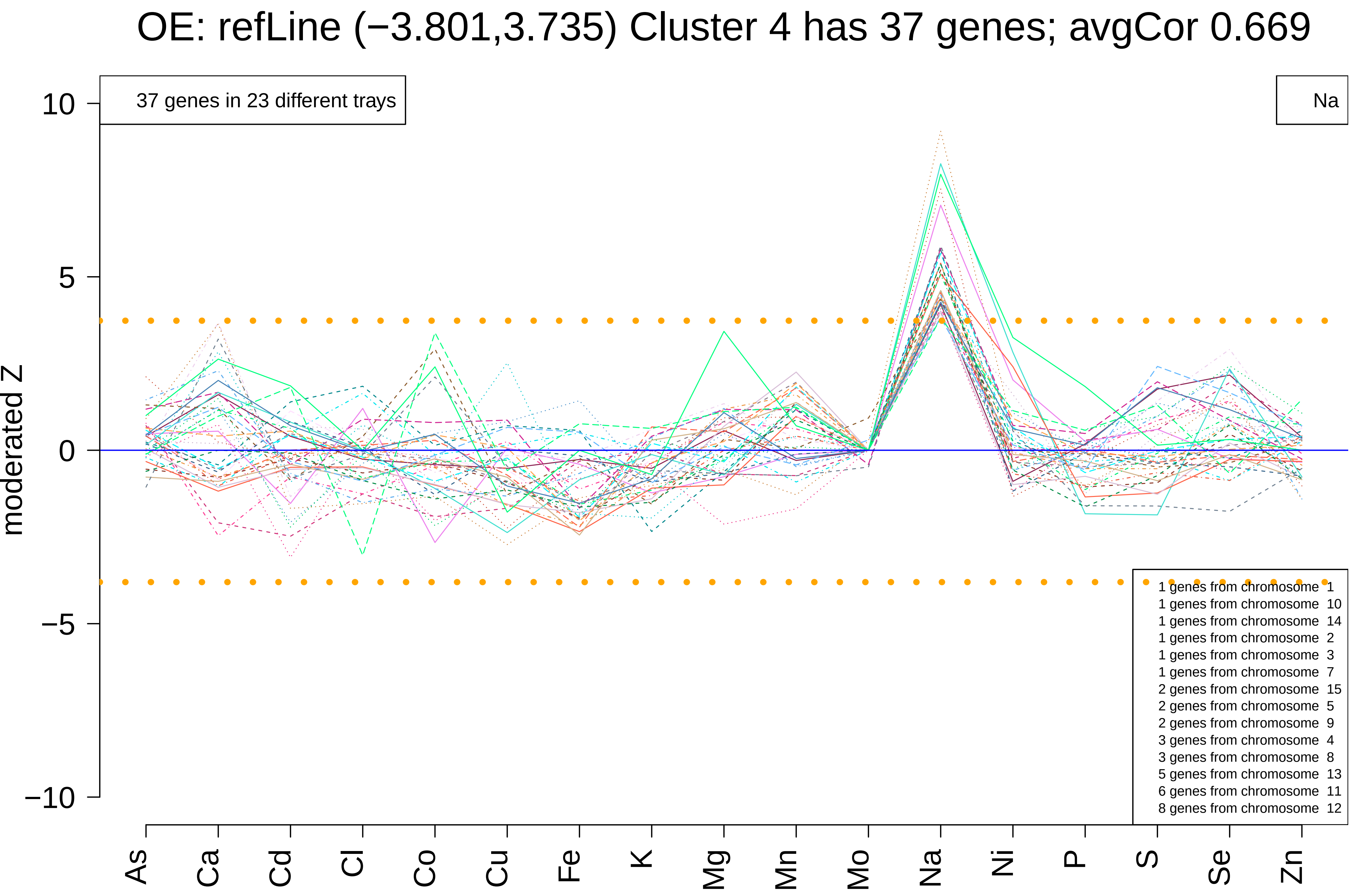
OE: refLine (−3.801,3.735) Cluster 4 has 37 genes; avgCor 0.669
37 genes in 23 different trays
Na
moderated Z
10
5
0
−5
−10
As
Ca
Cd
Cl
Co
Cu
Fe
K
Mg
Mn
Mo
Na
Ni
P
S
Se
Zn
1 genes from chromosome 1
1 genes from chromosome 10
1 genes from chromosome 14
1 genes from chromosome 2
1 genes from chromosome 3
1 genes from chromosome 7
2 genes from chromosome 15
2 genes from chromosome 5
2 genes from chromosome 9
3 genes from chromosome 4
3 genes from chromosome 8
5 genes from chromosome 13
6 genes from chromosome 11
8 genes from chromosome 12

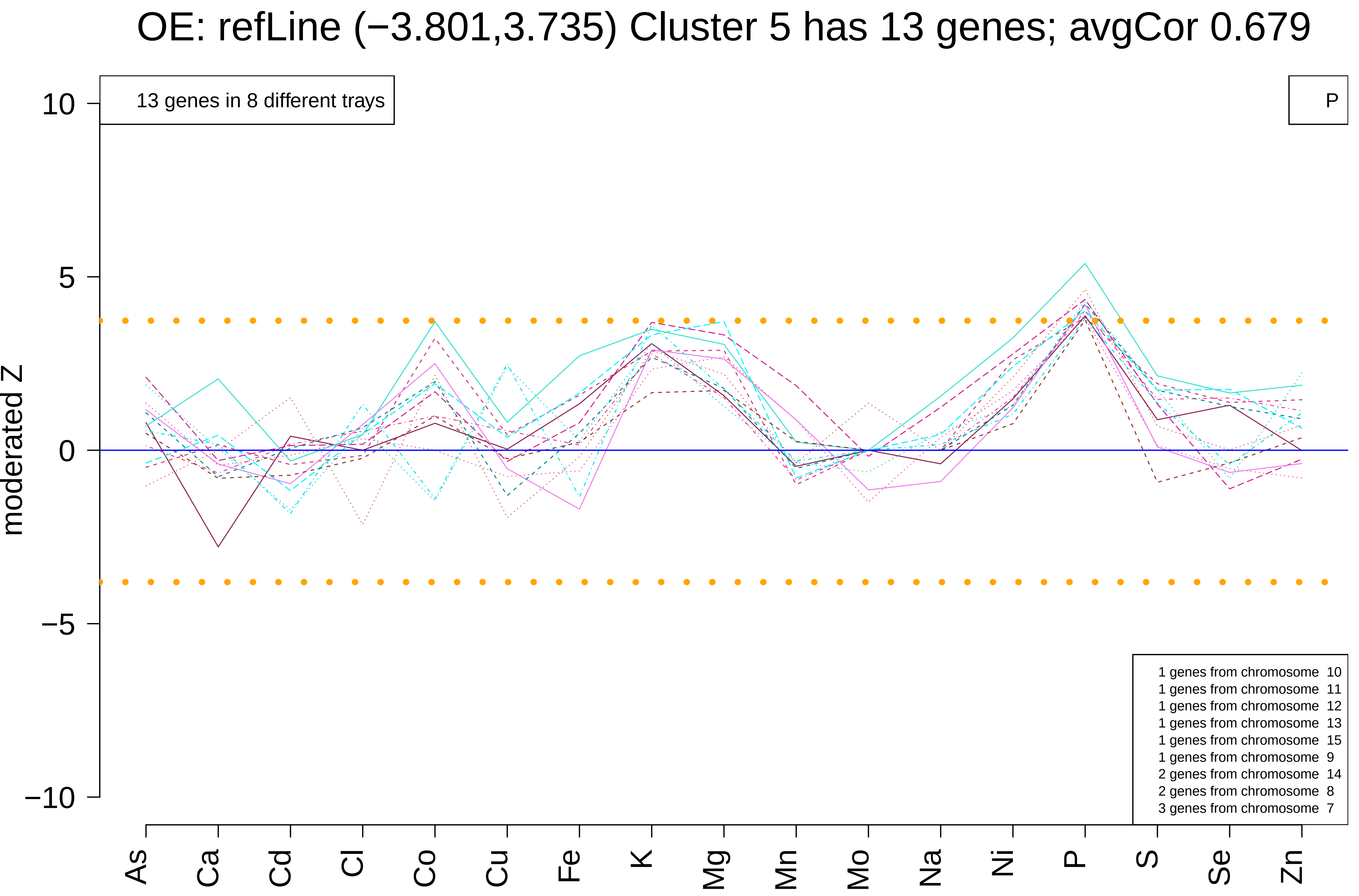

OE: refLine (−3.801,3.735) Cluster 5 has 13 genes; avgCor 0.679
13 genes in 8 different trays
P
moderated Z
10
5
0
−5
−10
As
Ca
Cd
Cl
Co
Cu
Fe
K
Mg
Mn
Mo
Na
Ni
P
S
Se
Zn
1 genes from chromosome 10
1 genes from chromosome 11
1 genes from chromosome 12
1 genes from chromosome 13
1 genes from chromosome 15
1 genes from chromosome 9
2 genes from chromosome 14
2 genes from chromosome 8
3 genes from chromosome 7

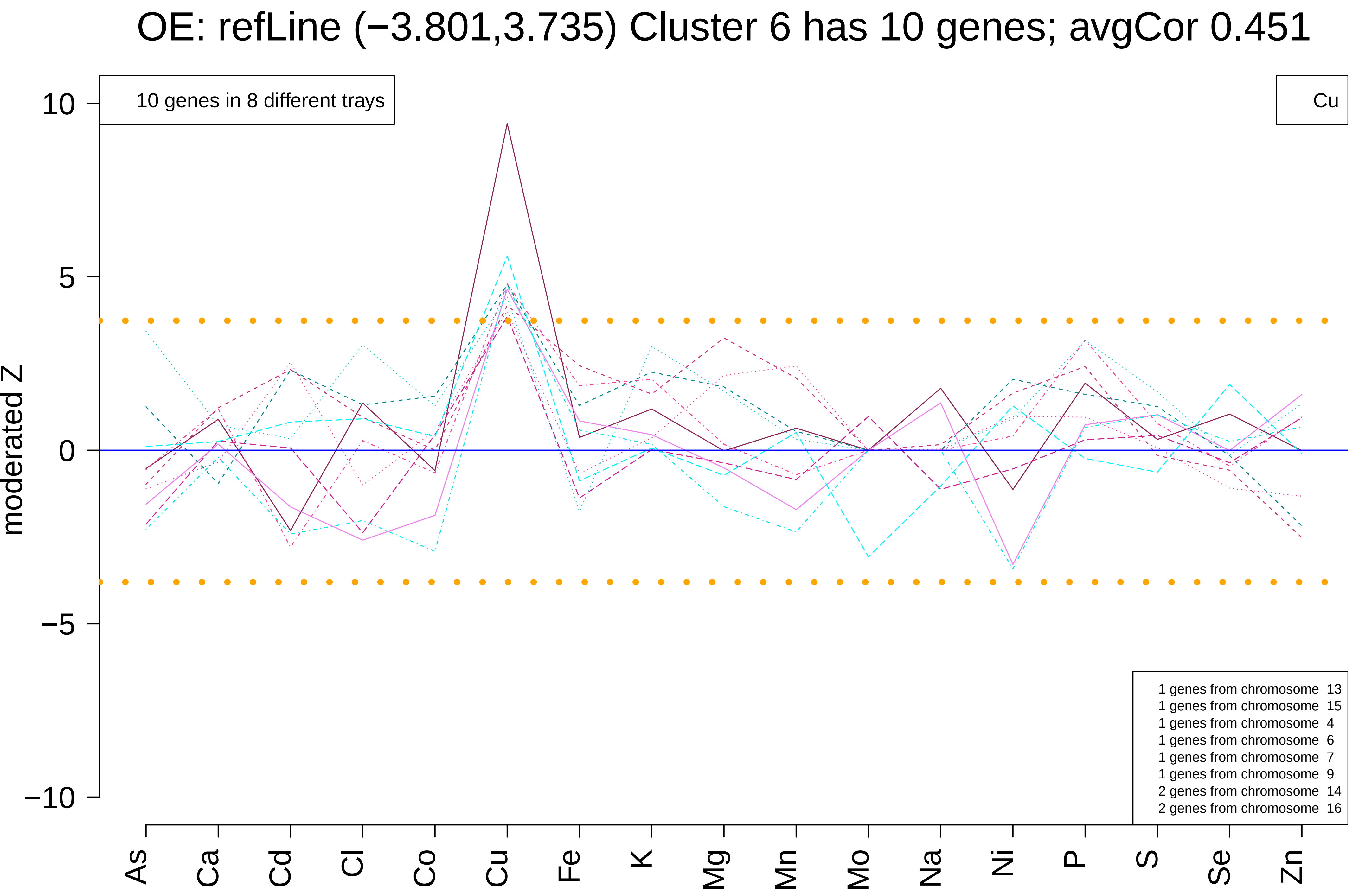
OE: refLine (−3.801,3.735) Cluster 6 has 10 genes; avgCor 0.451
10 genes in 8 different trays
Cu
moderated Z
10
5
0
−5
−10
As
Ca
Cd
Cl
Co
Cu
Fe
K
Mg
Mn
Mo
Na
Ni
P
S
Se
Zn
1 genes from chromosome 13
1 genes from chromosome 15
1 genes from chromosome 4
1 genes from chromosome 6
1 genes from chromosome 7
1 genes from chromosome 9
2 genes from chromosome 14
2 genes from chromosome 16

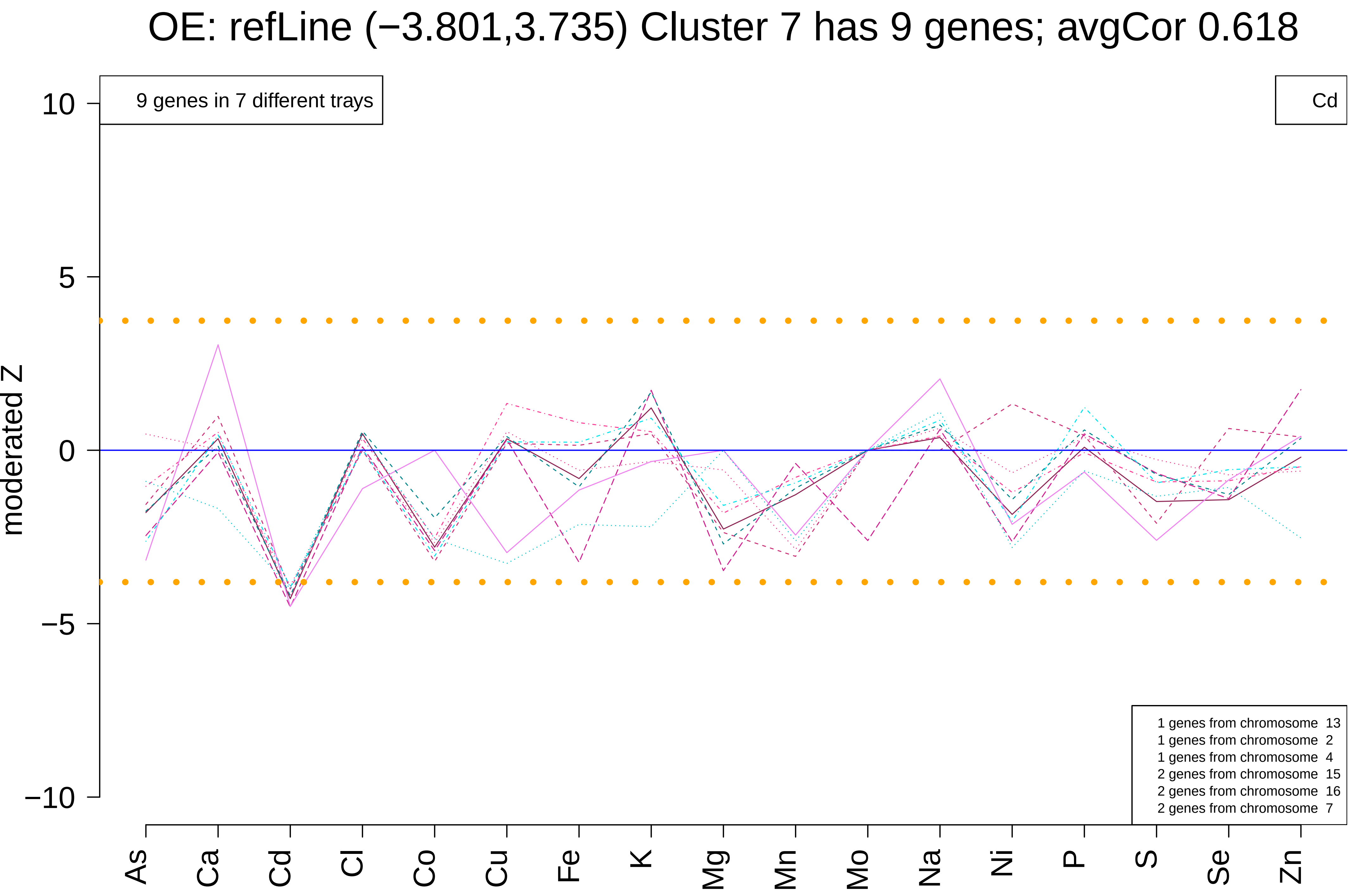

OE: refLine (−3.801,3.735) Cluster 7 has 9 genes; avgCor 0.618
9 genes in 7 different trays
Cd
moderated Z
10
5
0
−5
−10
As
Ca
Cd
Cl
Co
Cu
Fe
K
Mg
Mn
Mo
Na
Ni
P
S
Se
Zn
1 genes from chromosome 13
1 genes from chromosome 2
1 genes from chromosome 4
2 genes from chromosome 15
2 genes from chromosome 16
2 genes from chromosome 7

OE: refLine (−3.801,3.735) Cluster 8 has 9 genes; avgCor 0.534

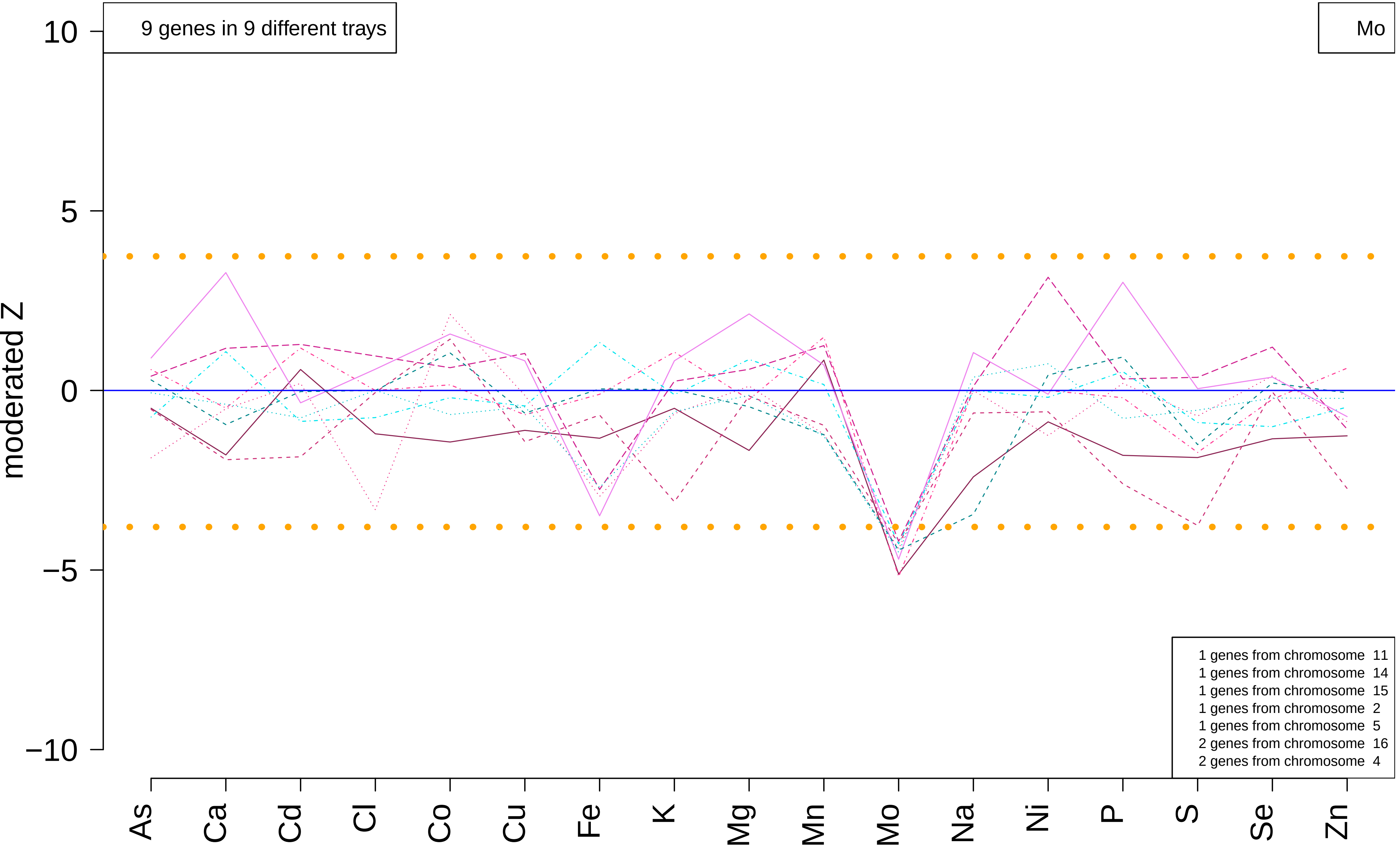

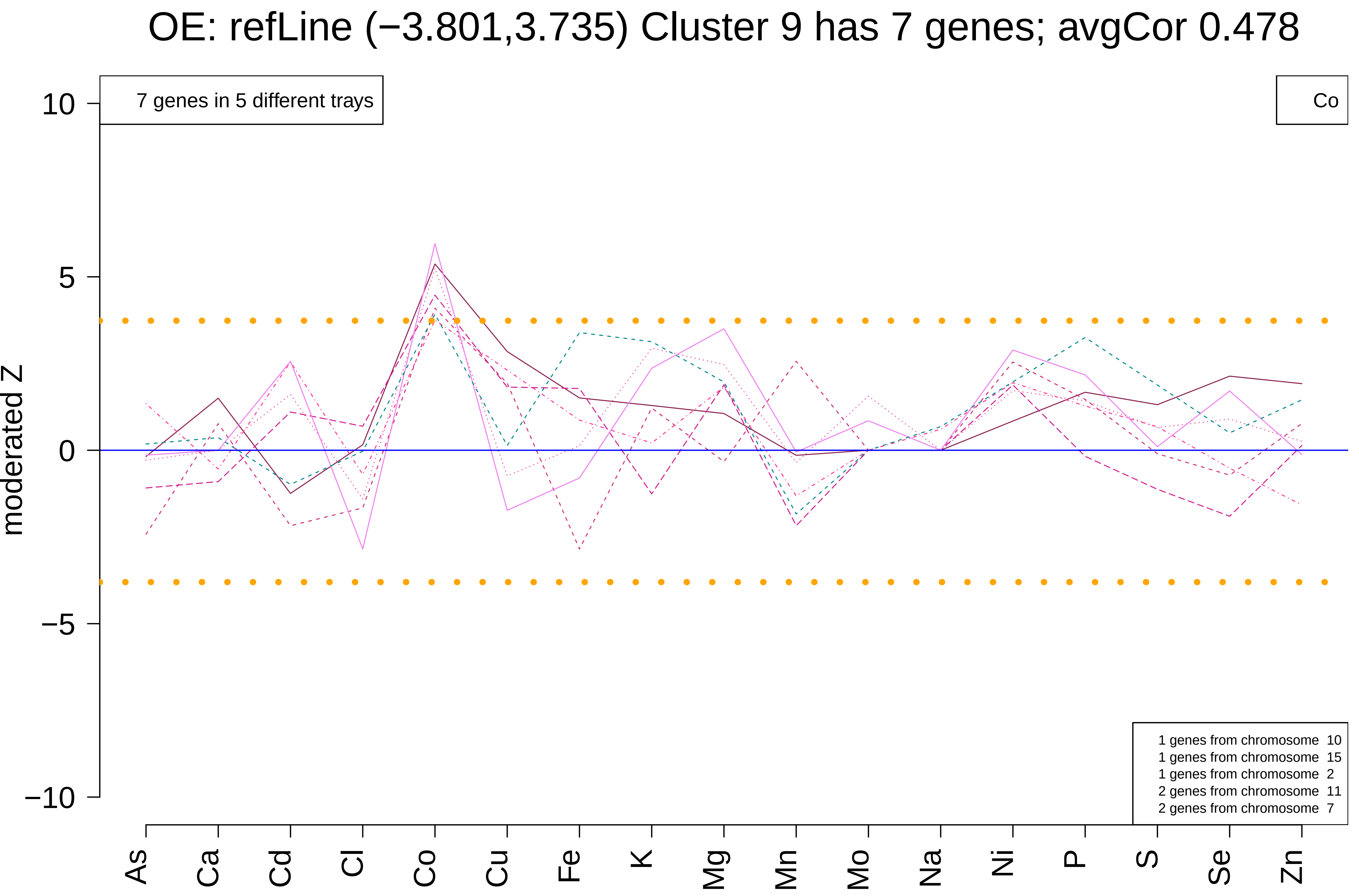
OE: refLine (−3.801,3.735) Cluster 9 has 7 genes; avgCor 0.478
7 genes in 5 different trays
Co
moderated Z
10
5
0
−5
−10
As
Ca
Cd
Cl
Co
Cu
Fe
K
Mg
Mn
Mo
Na
Ni
P
S
Se
Zn
1 genes from chromosome 10
1 genes from chromosome 15
1 genes from chromosome 2
2 genes from chromosome 11
2 genes from chromosome 7

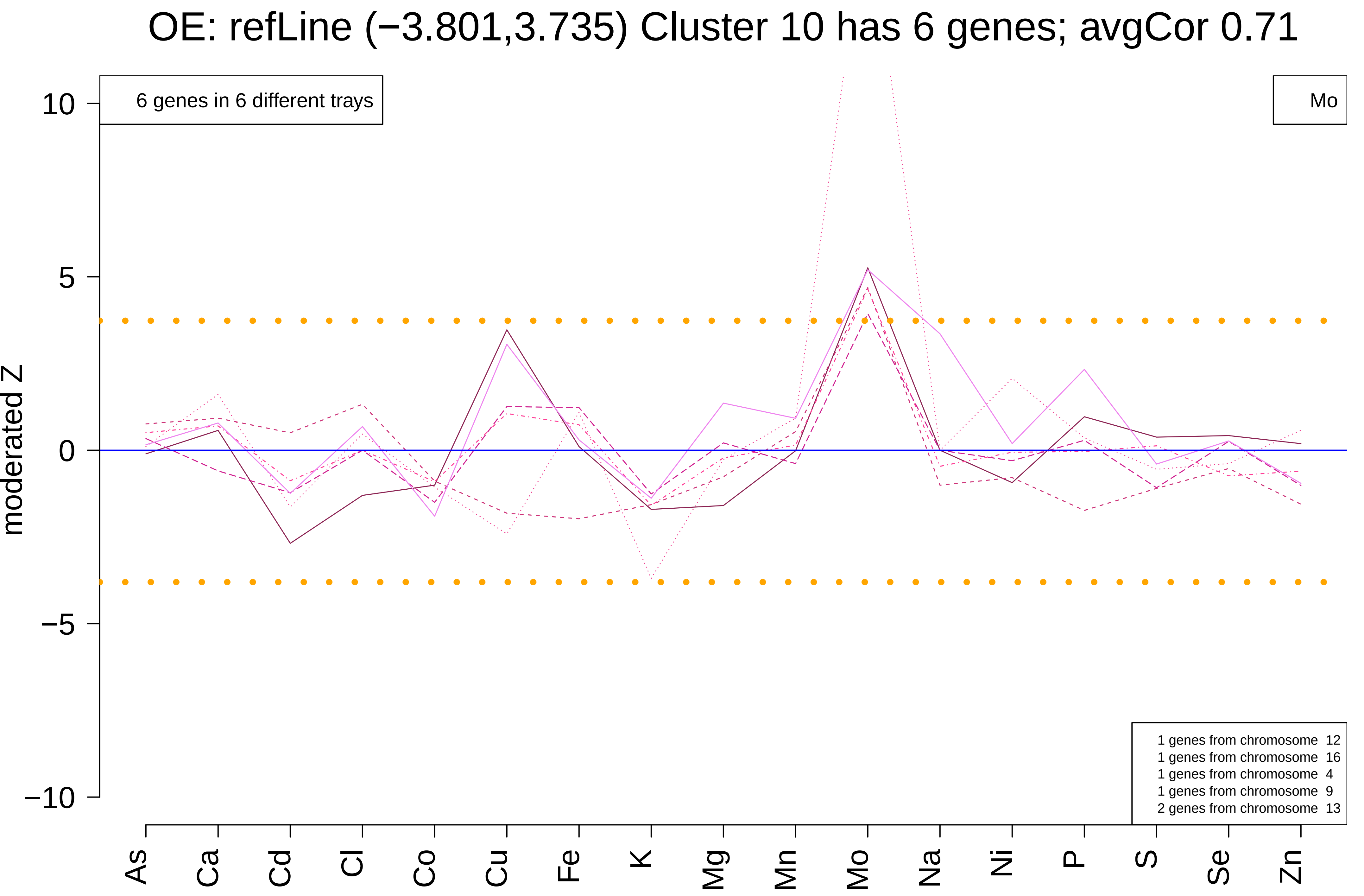
OE: refLine (−3.801,3.735) Cluster 10 has 6 genes; avgCor 0.71
6 genes in 6 different trays
Mo
moderated Z
10
5
0
−5
−10
1 genes from chromosome 12
1 genes from chromosome 16
1 genes from chromosome 4
1 genes from chromosome 9
2 genes from chromosome 13
As
Ca
Cd
Cl
Co
Cu
Fe
K
Mg
Mn
Mo
Na
Ni
P
S
Se
Zn

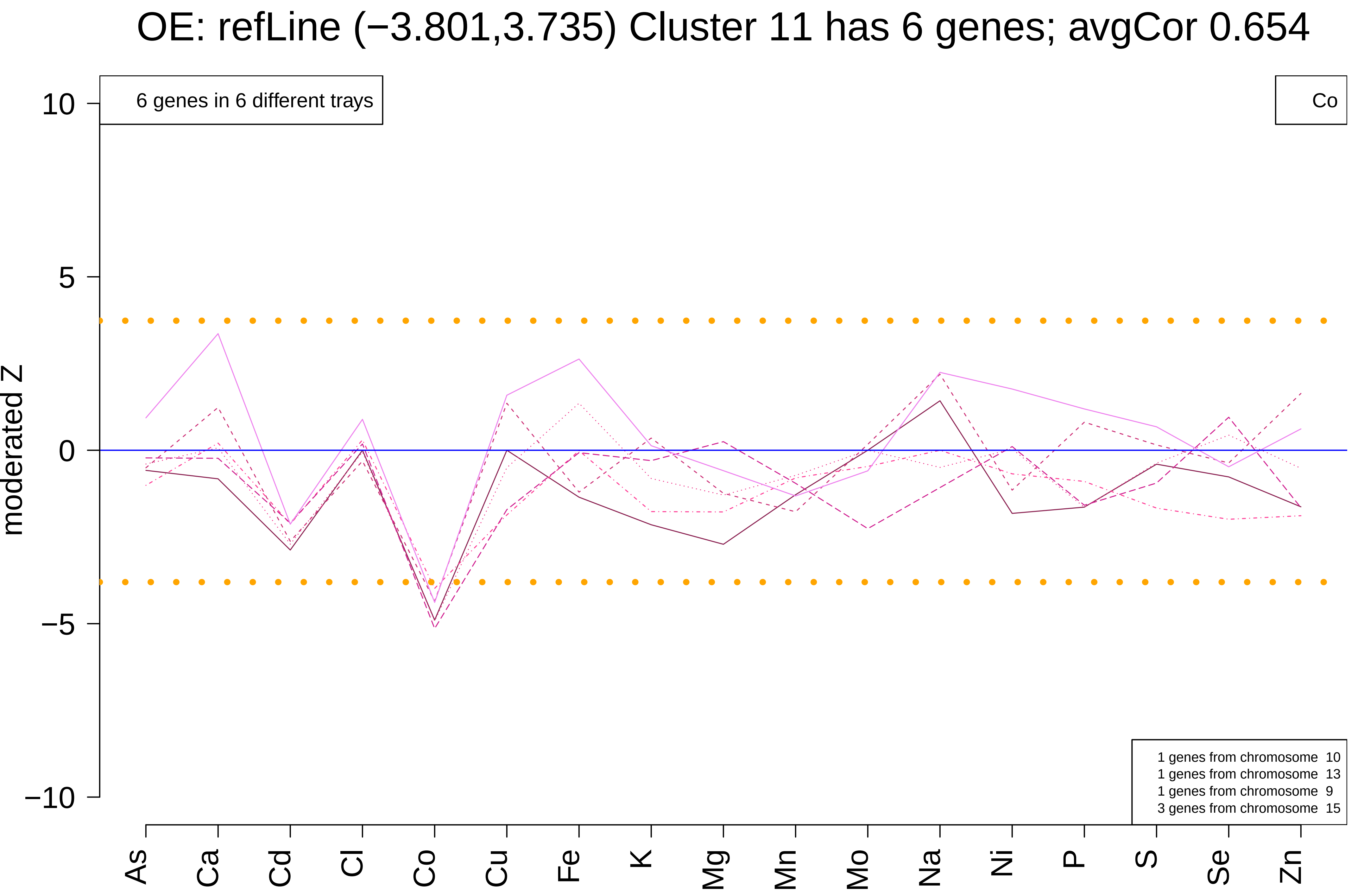

OE: refLine (−3.801,3.735) Cluster 11 has 6 genes; avgCor 0.654
6 genes in 6 different trays
Co
10
5
0
−5
−10
moderated Z
1 genes from chromosome 10
1 genes from chromosome 13
1 genes from chromosome 9
3 genes from chromosome 15
As
Ca
Cd
Cl
Co
Cu
Fe
K
Mg
Mn
Mo
Na
Ni
P
S
Se
Zn

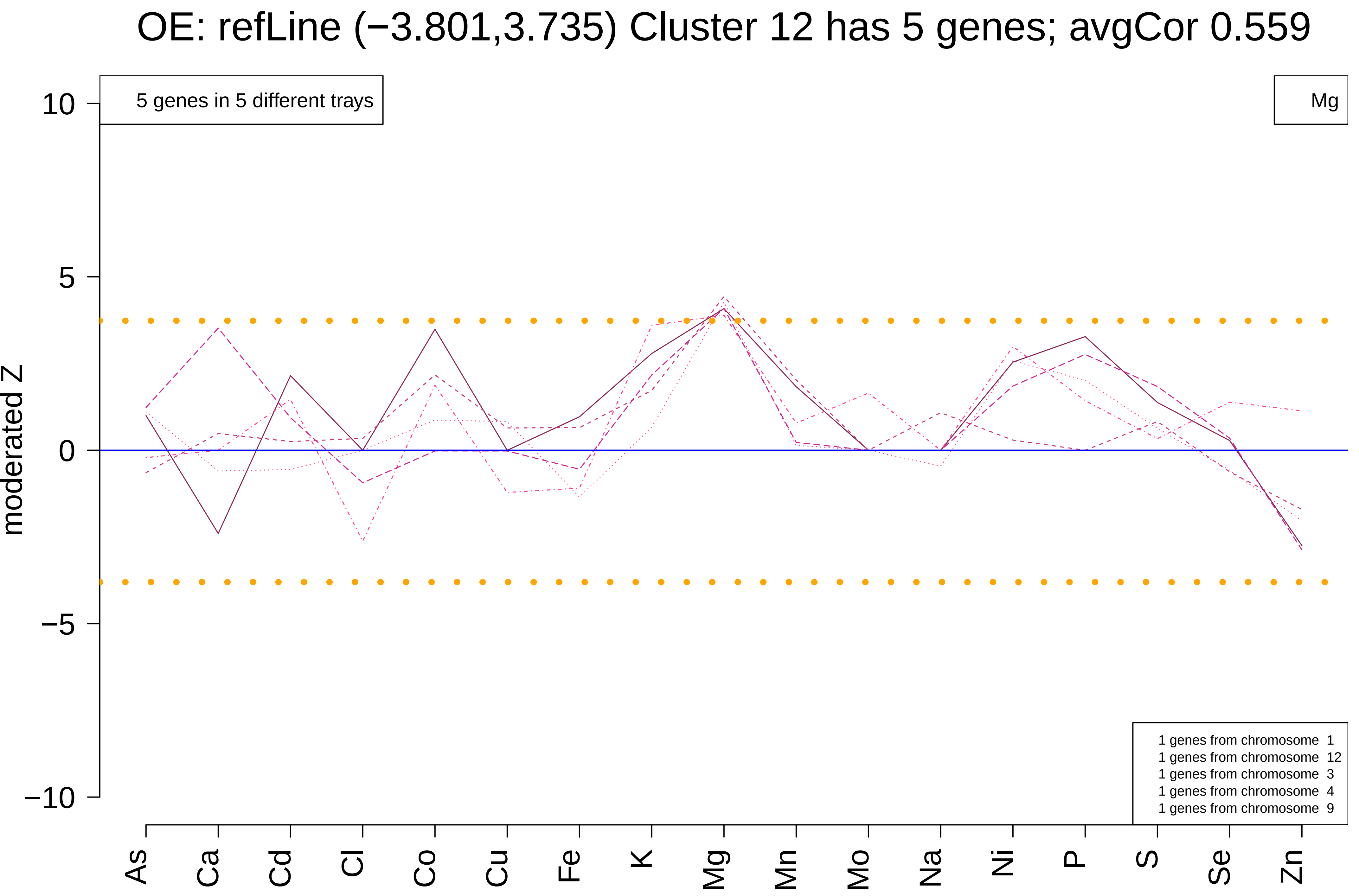

OE: refLine (−3.801,3.735) Cluster 12 has 5 genes; avgCor 0.559
5 genes in 5 different trays
Mg
moderated Z
10
5
0
−5
−10
As
Ca
Cd
Cl
Co
Cu
Fe
K
Mg
Mn
Mo
Na
Ni
P
S
Se
Zn
1 genes from chromosome 1
1 genes from chromosome 12
1 genes from chromosome 3
1 genes from chromosome 4
1 genes from chromosome 9

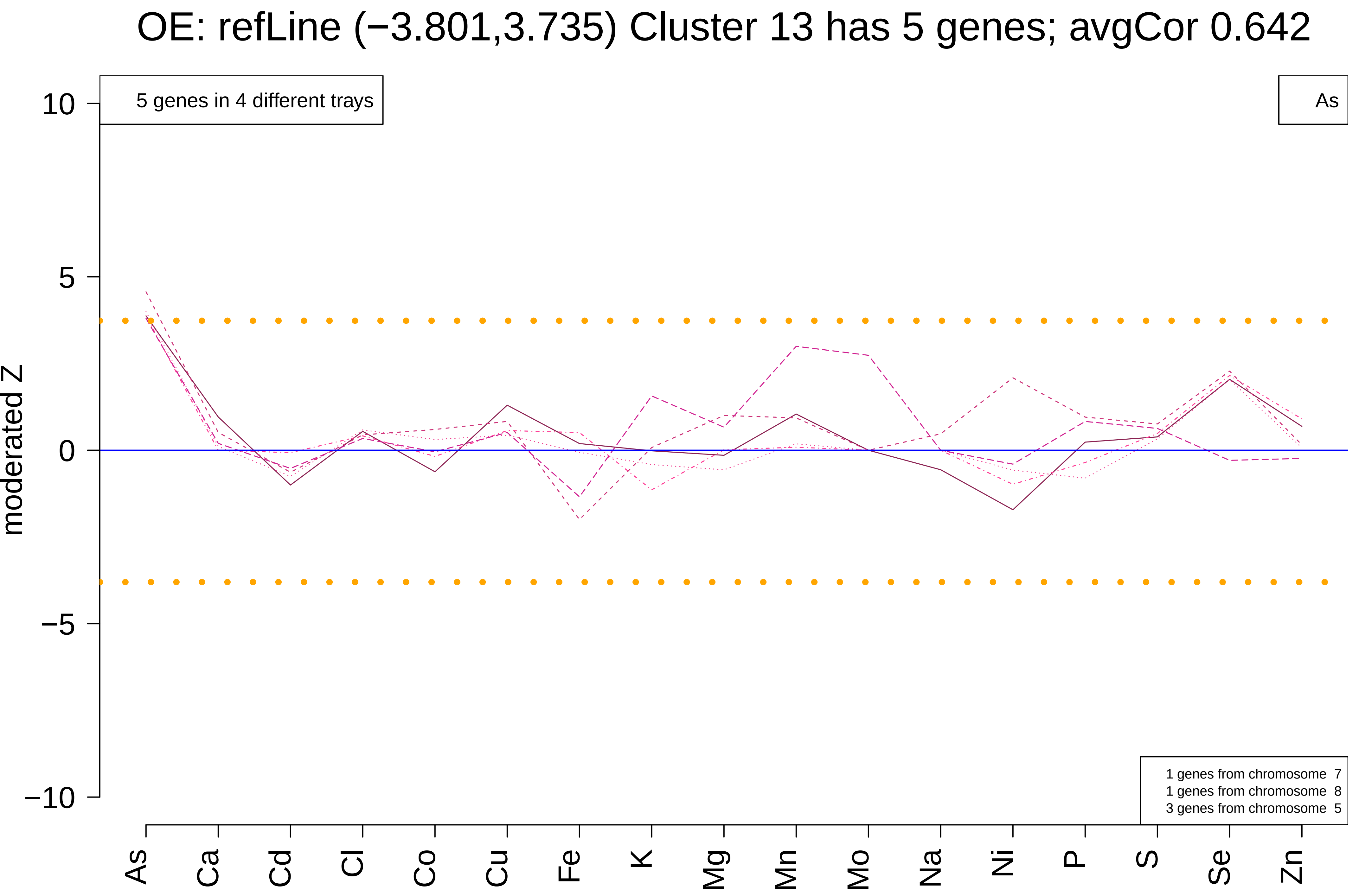

OE: refLine (−3.801,3.735) Cluster 13 has 5 genes; avgCor 0.642
5 genes in 4 different trays
As
moderated Z
10
5
0
−5
−10
1 genes from chromosome 7
1 genes from chromosome 8
3 genes from chromosome 5
As
Ca
Cd
Cl
Co
Cu
Fe
K
Mg
Mn
Mo
Na
Ni
P
S
Se
Zn

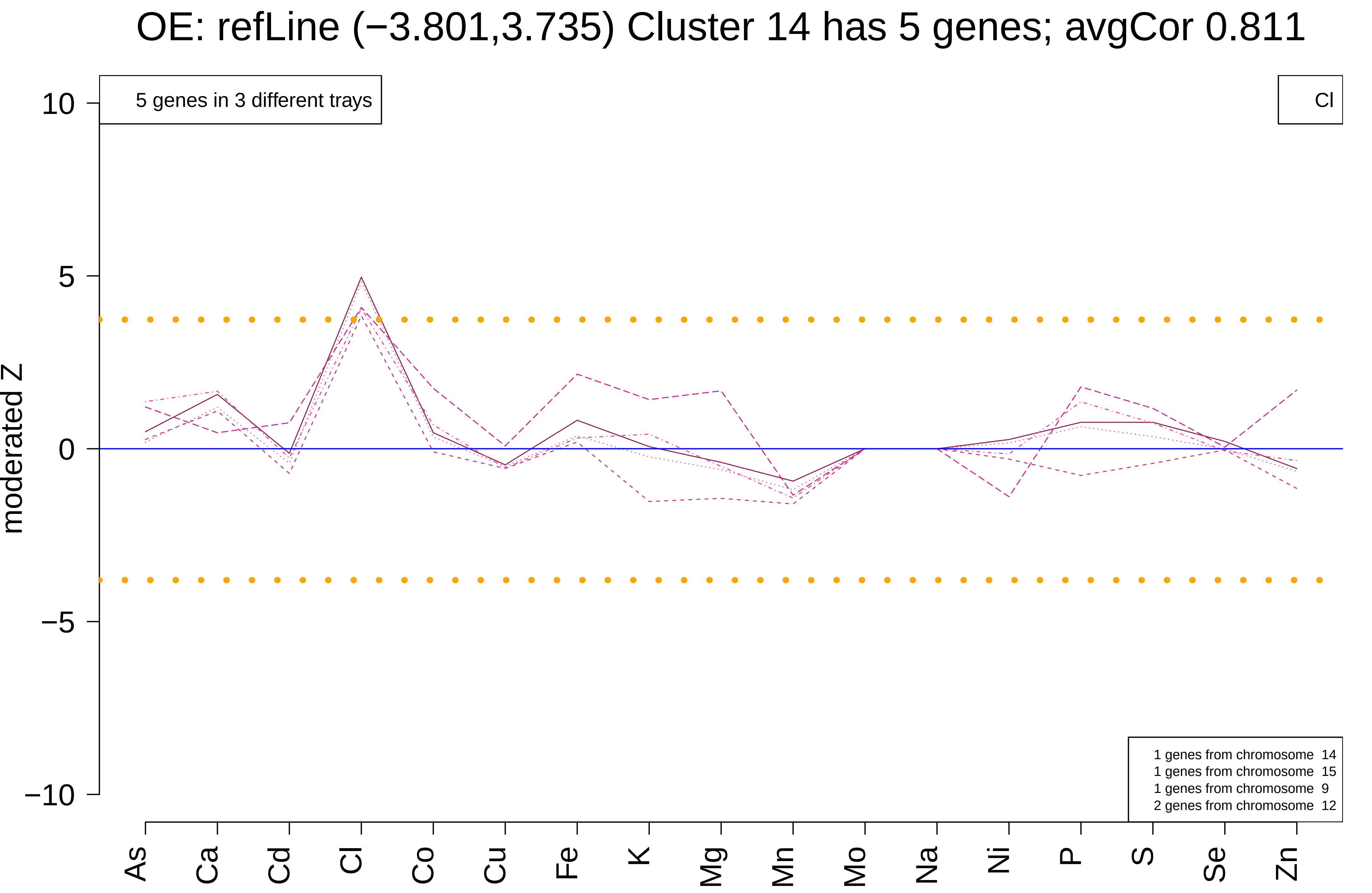

OE: refLine (−3.801,3.735) Cluster 14 has 5 genes; avgCor 0.811
5 genes in 3 different trays
Cl
moderated Z
10
5
0
−5
−10
1 genes from chromosome 14
1 genes from chromosome 15
1 genes from chromosome 9
2 genes from chromosome 12
As
Ca
Cd
Cl
Co
Cu
Fe
K
Mg
Mn
Mo
Na
Ni
P
S
Se
Zn

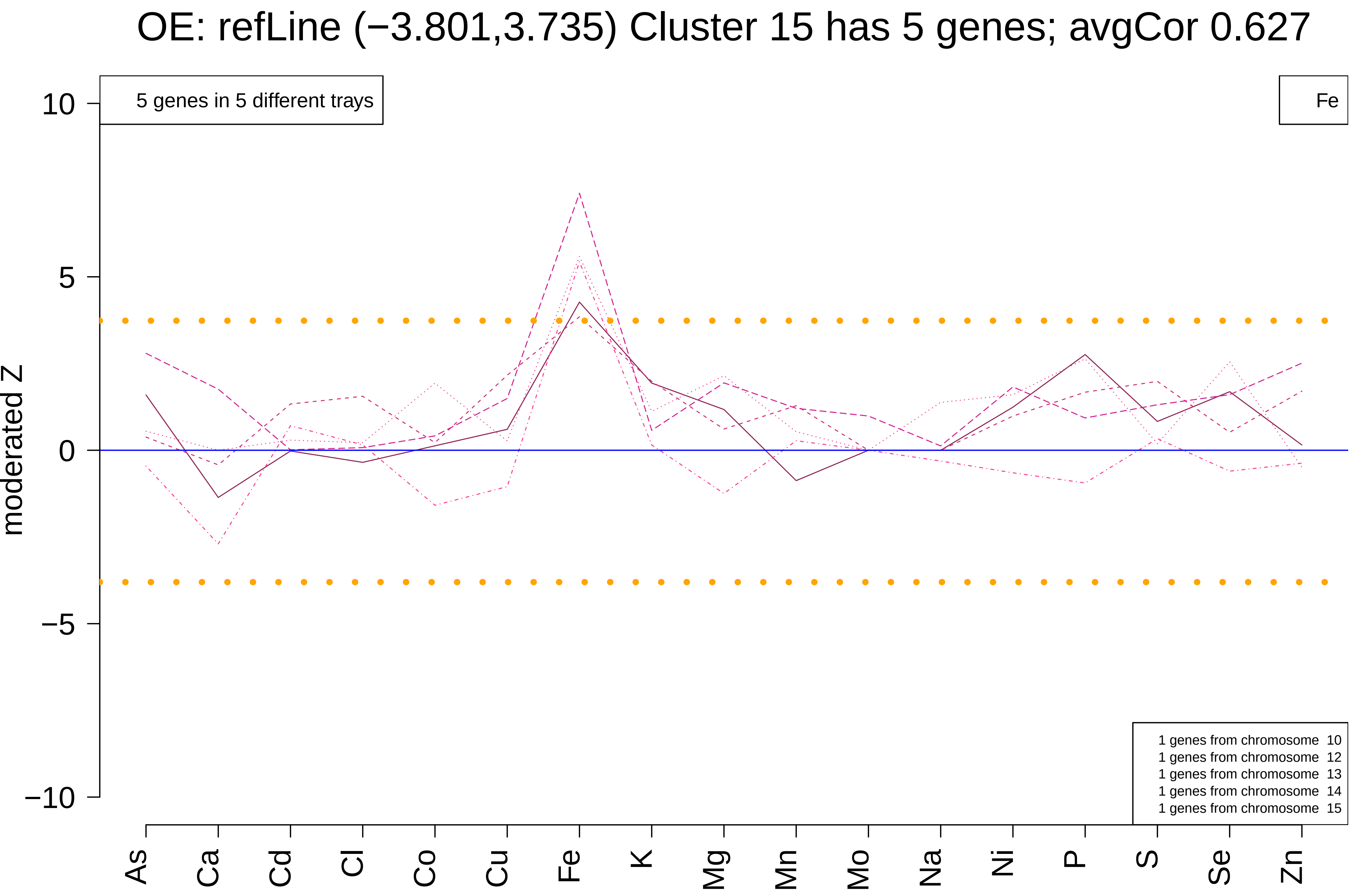

OE: refLine (−3.801,3.735) Cluster 15 has 5 genes; avgCor 0.627
5 genes in 5 different trays
Fe
moderated Z
10
5
0
−5
−10
As
Ca
Cd
Cl
Co
Cu
Fe
K
Mg
Mn
Mo
Na
Ni
P
S
Se
Zn
1 genes from chromosome 10
1 genes from chromosome 12
1 genes from chromosome 13
1 genes from chromosome 14
1 genes from chromosome 15

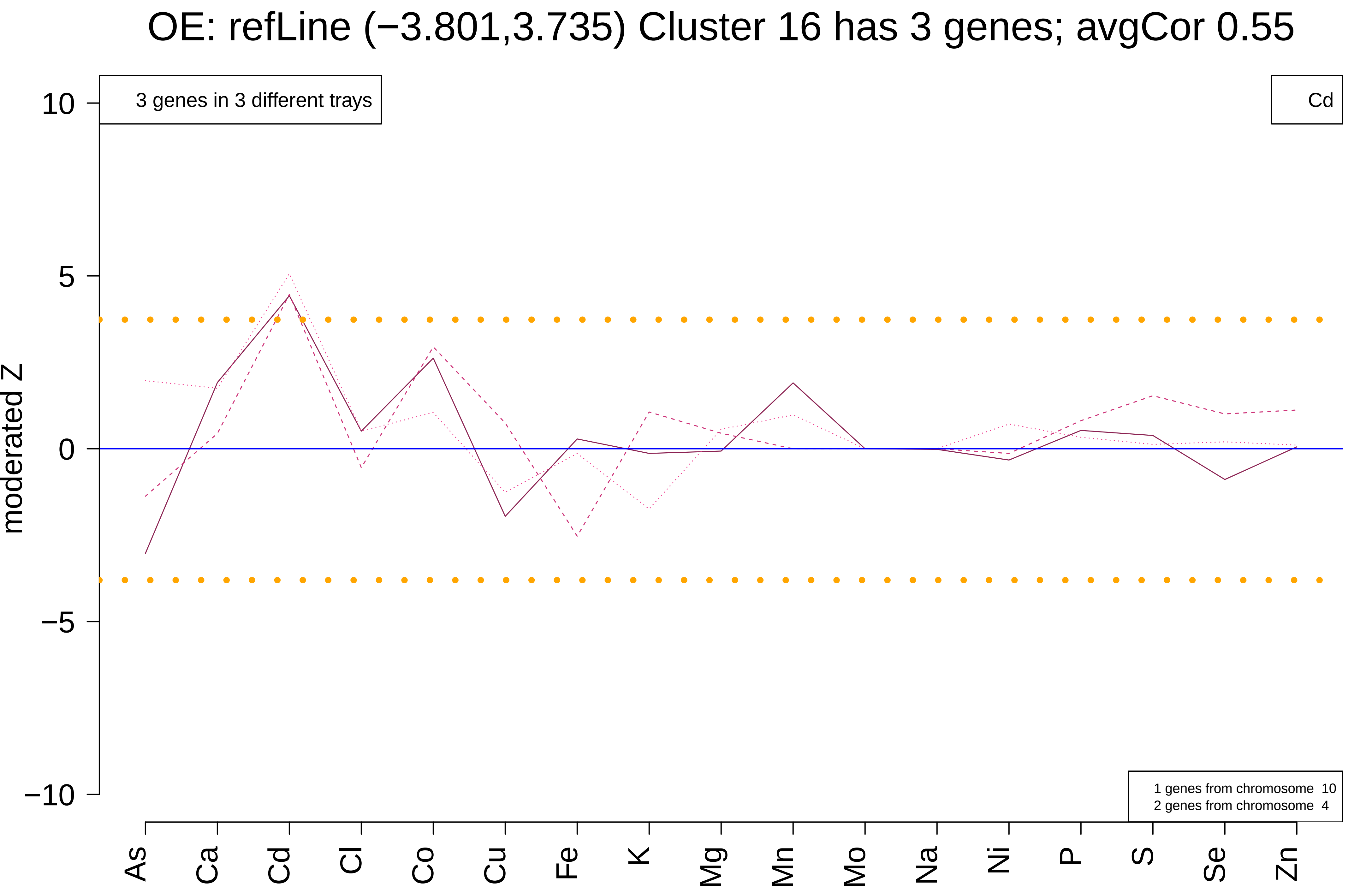

OE: refLine (−3.801,3.735) Cluster 16 has 3 genes; avgCor 0.55
3 genes in 3 different trays
Cd
moderated Z
10
5
0
−5
−10
As
Ca
Cd
Cl
Co
Cu
Fe
K
Mg
Mn
Mo
Na
Ni
P
S
Se
Zn
1 genes from chromosome 10
2 genes from chromosome 4

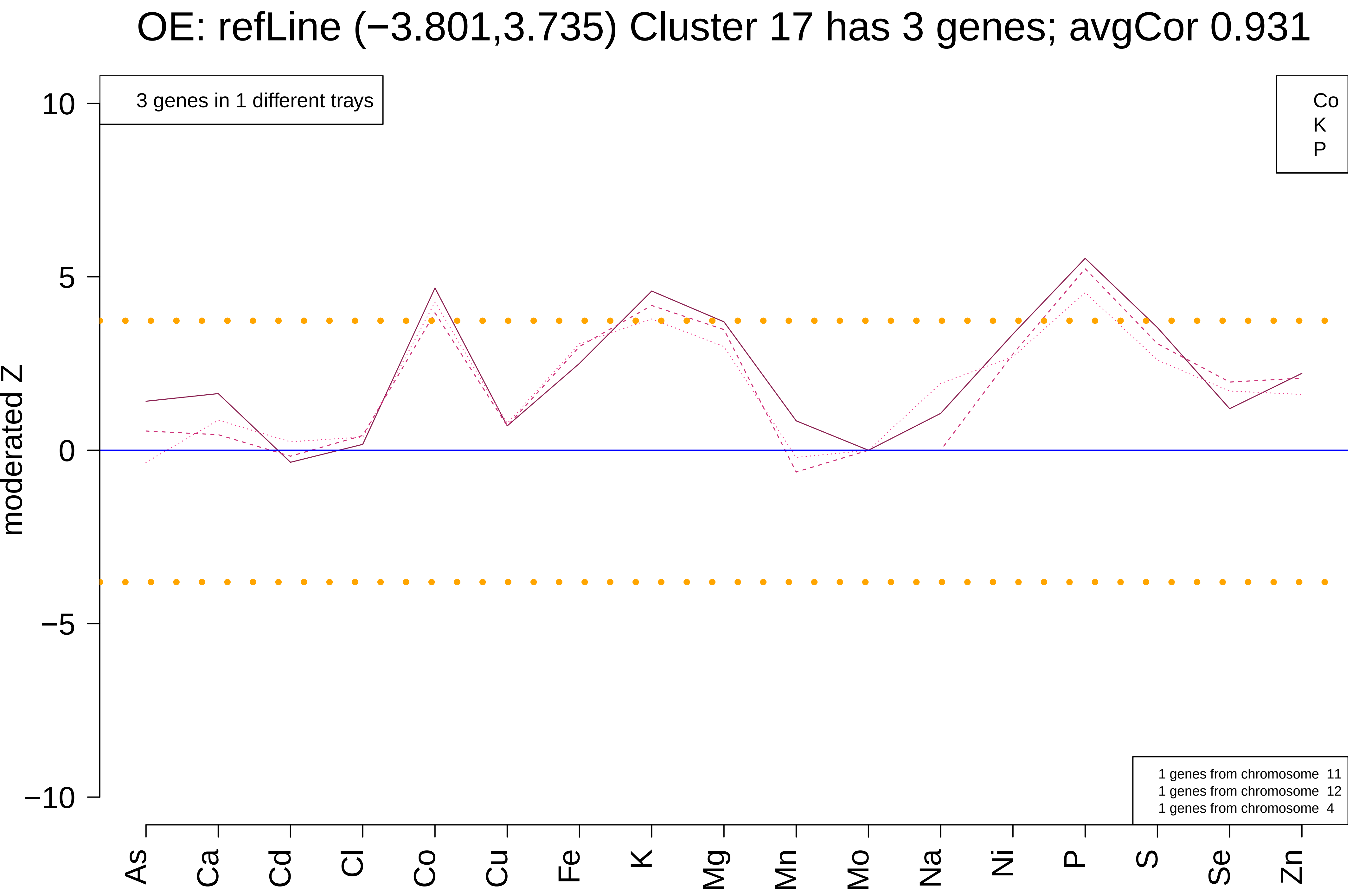

OE: refLine (−3.801,3.735) Cluster 17 has 3 genes; avgCor 0.931
3 genes in 1 different trays
Co
K
P
moderated Z
10
5
0
−5
−10
1 genes from chromosome 11
1 genes from chromosome 12
1 genes from chromosome 4
As
Ca
Cd
Cl
Co
Cu
Fe
K
Mg
Mn
Mo
Na
Ni
P
S
Se
Zn

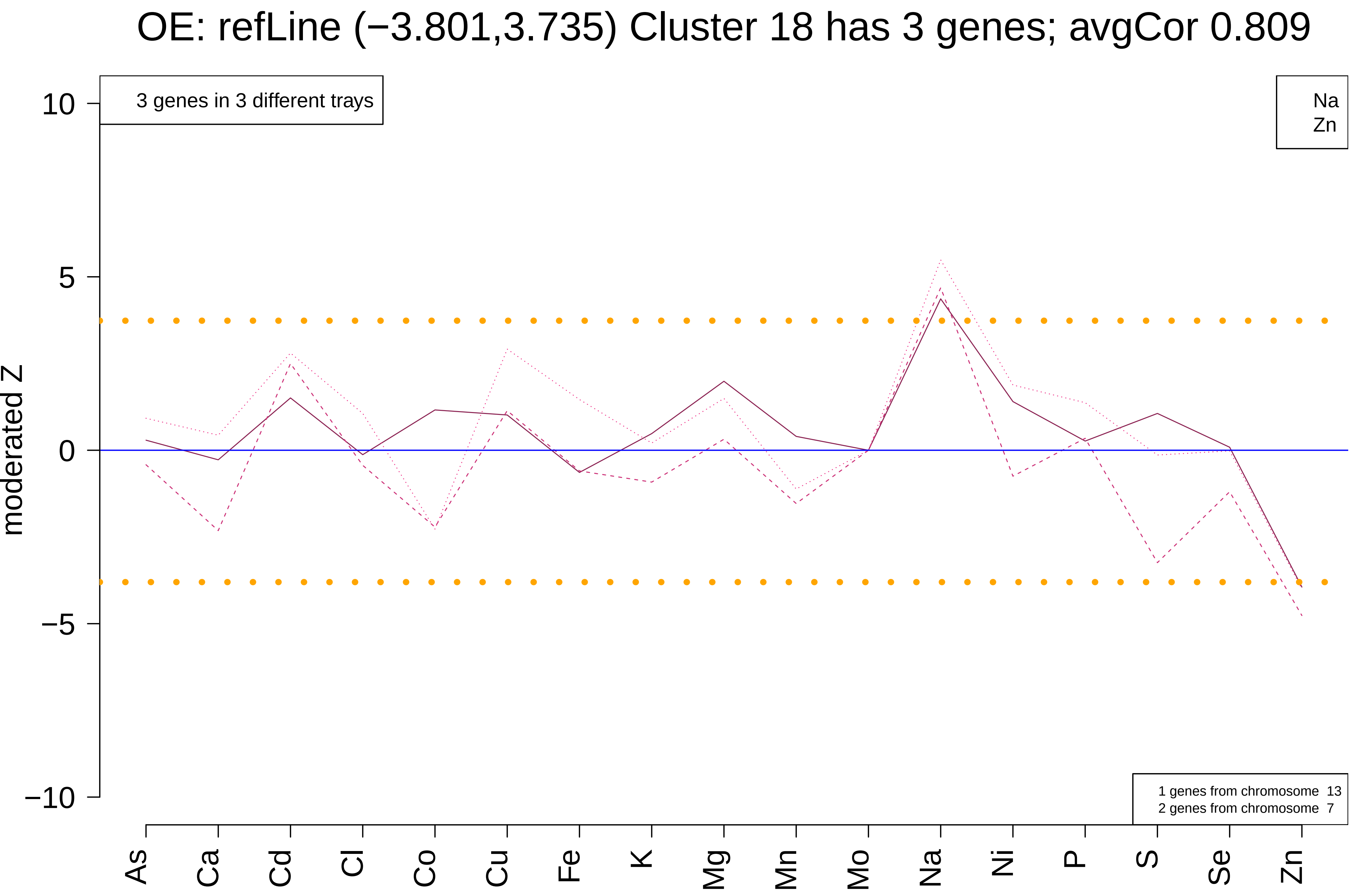
OE: refLine (−3.801,3.735) Cluster 18 has 3 genes; avgCor 0.809
3 genes in 3 different trays
Na
Zn
10
5
0
−5
−10
moderated Z
1 genes from chromosome 13
2 genes from chromosome 7
As
Ca
Cd
Cl
Co
Cu
Fe
K
Mg
Mn
Mo
Na
Ni
P
S
Se
Zn
