# Supplementary material for: High-resolution genome-wide scan of genes, gene-networks and cellular systems impacting the yeast ionome
Source: BMC Genomics. 2012 Nov 14;13:623. doi: 10.1186/1471-2164-13-623 (PMC3652779; doi:10.1186/1471-2164-13-623)

KO Ca : 96 significant mutants, 49 nodes, 88 edges

Number of significant elements in a mutant is between 1 and 5 .
Edge value is the correlation between the all elements profile of two mutants.

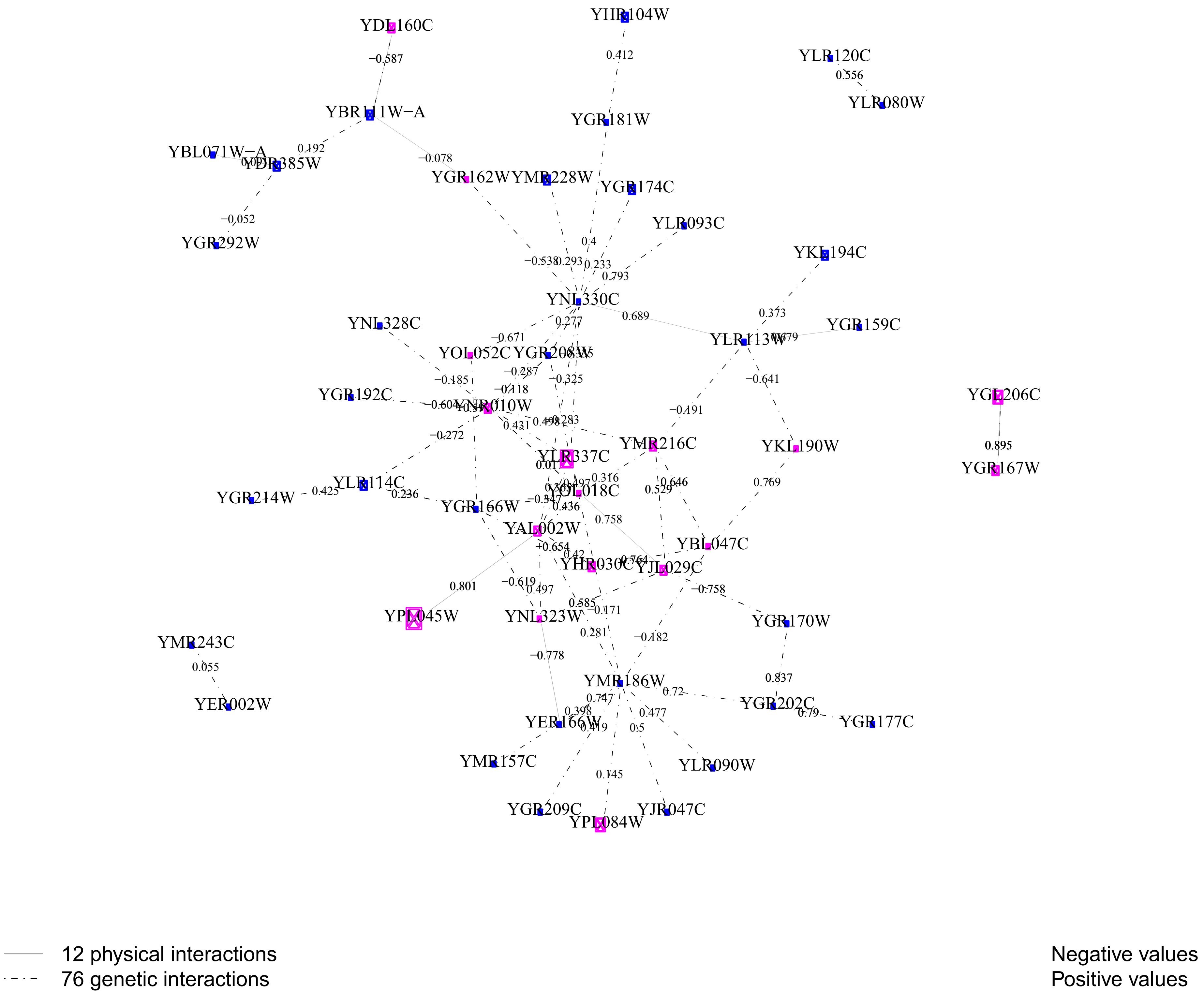

KO Cd : 294 significant mutants, 159 nodes, 550 edges

Number of significant elements in a mutant is between 1 and 5 .
Edge value is the correlation between the all elements profile of two mutants.

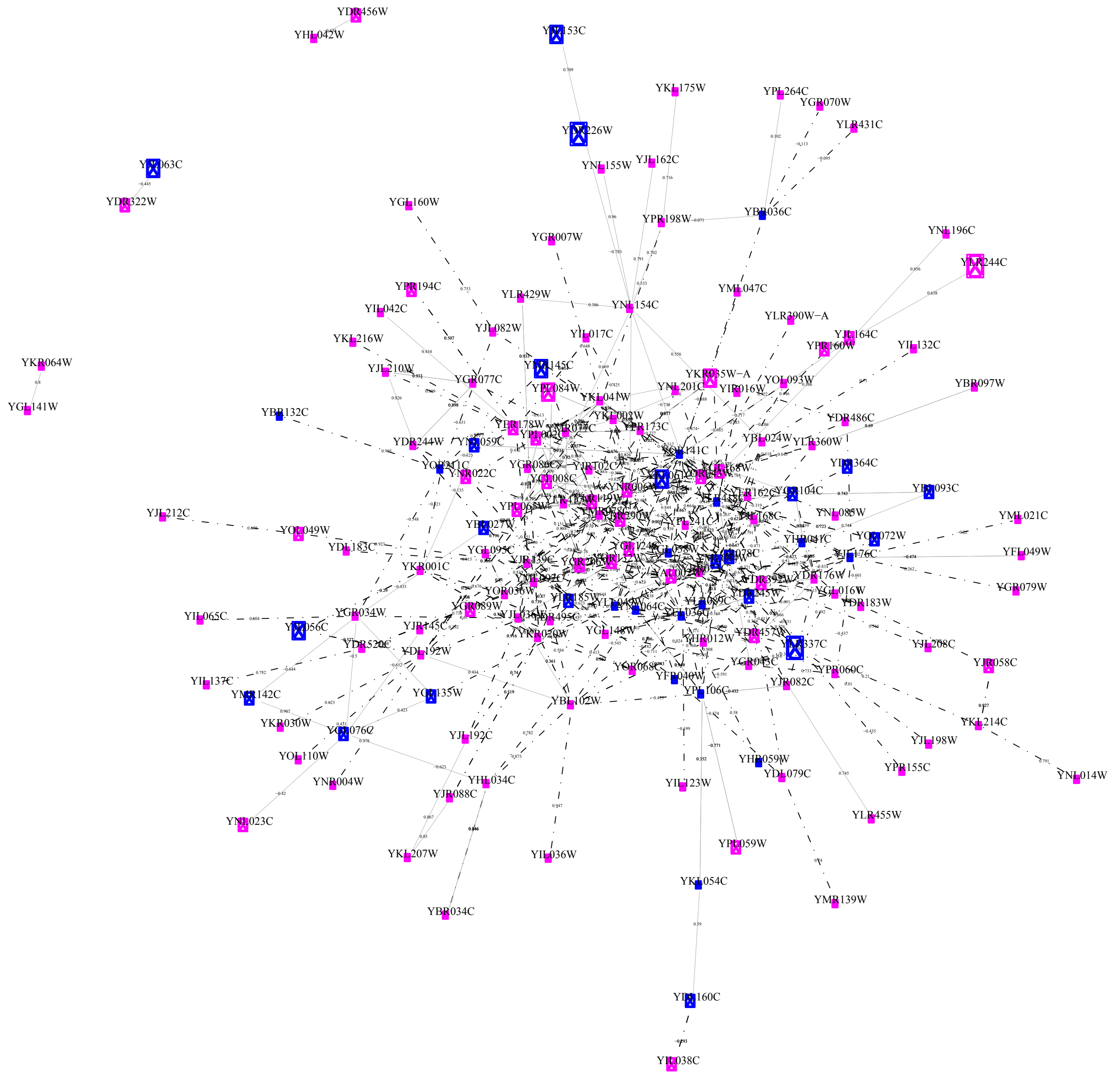

135 physical interactions
415 genetic interactions

Negative values
Positive values

KO Co : 80 significant mutants, 39 nodes, 145 edges

Number of significant elements in a mutant is between 1 and 6 .
Edge value is the correlation between the all elements profile of two mutants.

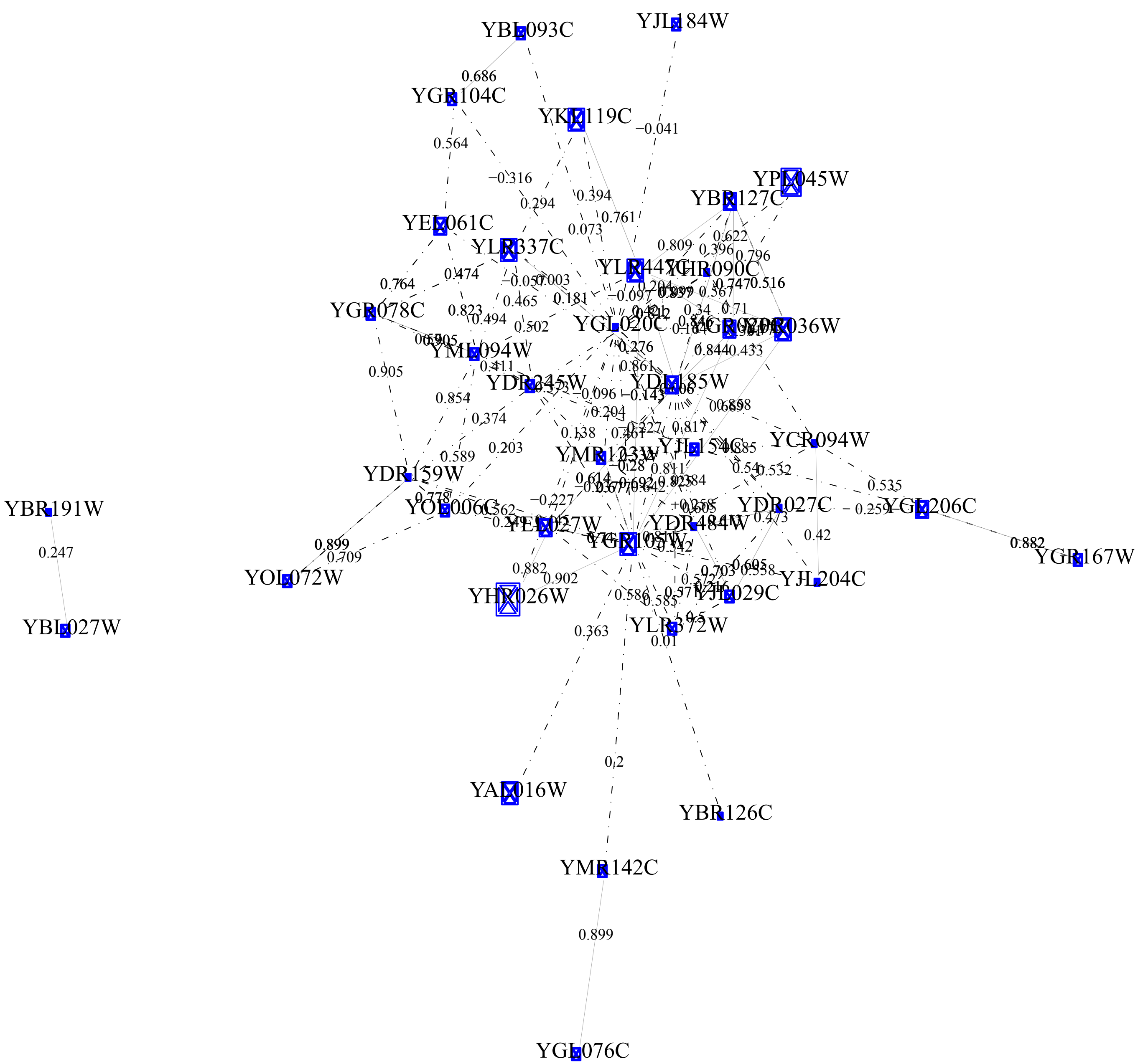

45 physical interactions
100 genetic interactions

Negative values
Positive values

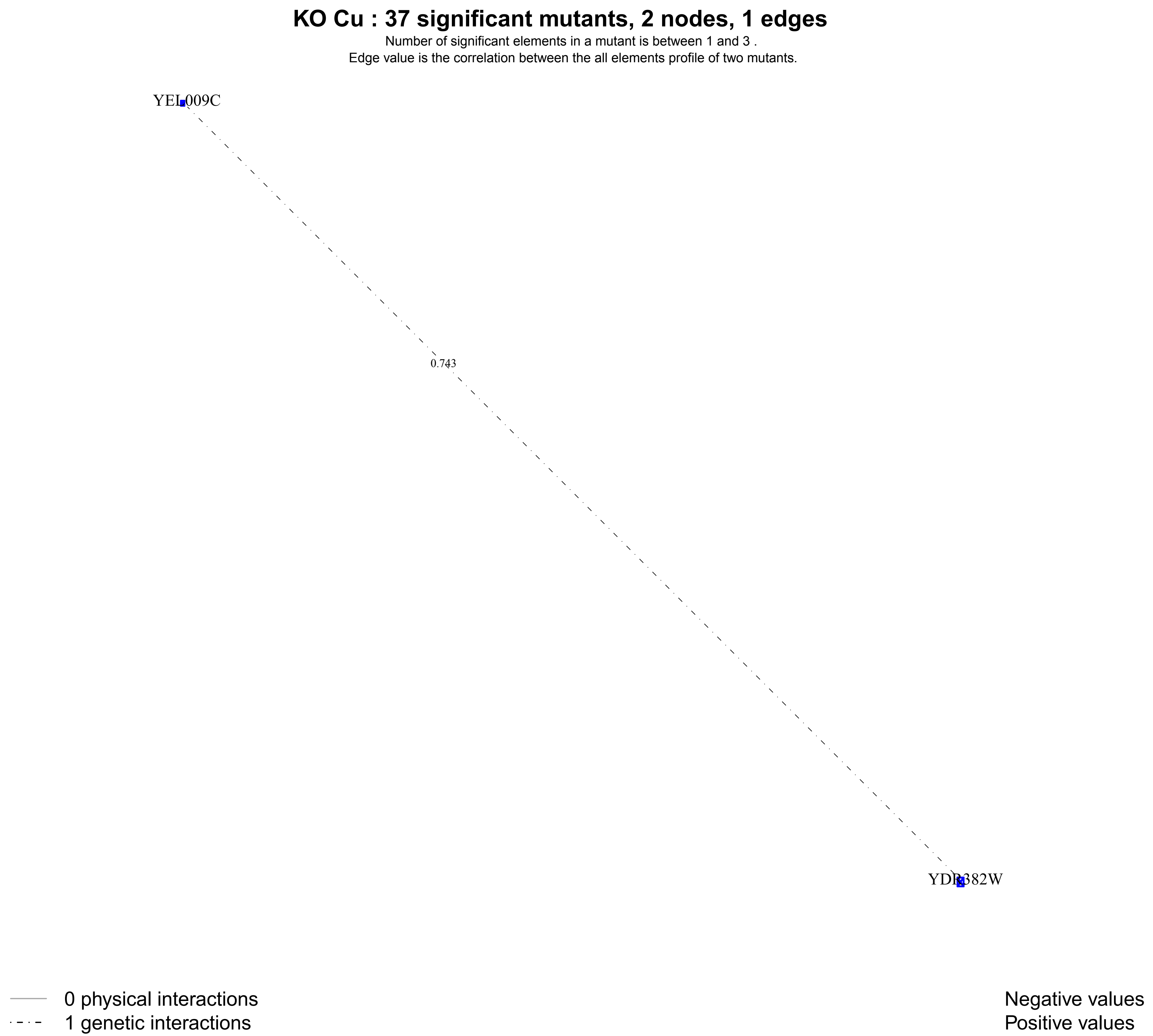
KO Cu : 37 significant mutants, 2 nodes, 1 edges
Number of significant elements in a mutant is between 1 and 3 .
Edge value is the correlation between the all elements profile of two mutants.
YEL009C
0.743
YDR382W
0 physical interactions
1 genetic interactions
Negative values
Positive values

KO K : 81 significant mutants, 30 nodes, 29 edges

Number of significant elements in a mutant is between 1 and 5 .

Edge value is the correlation between the all elements profile of two mutants.

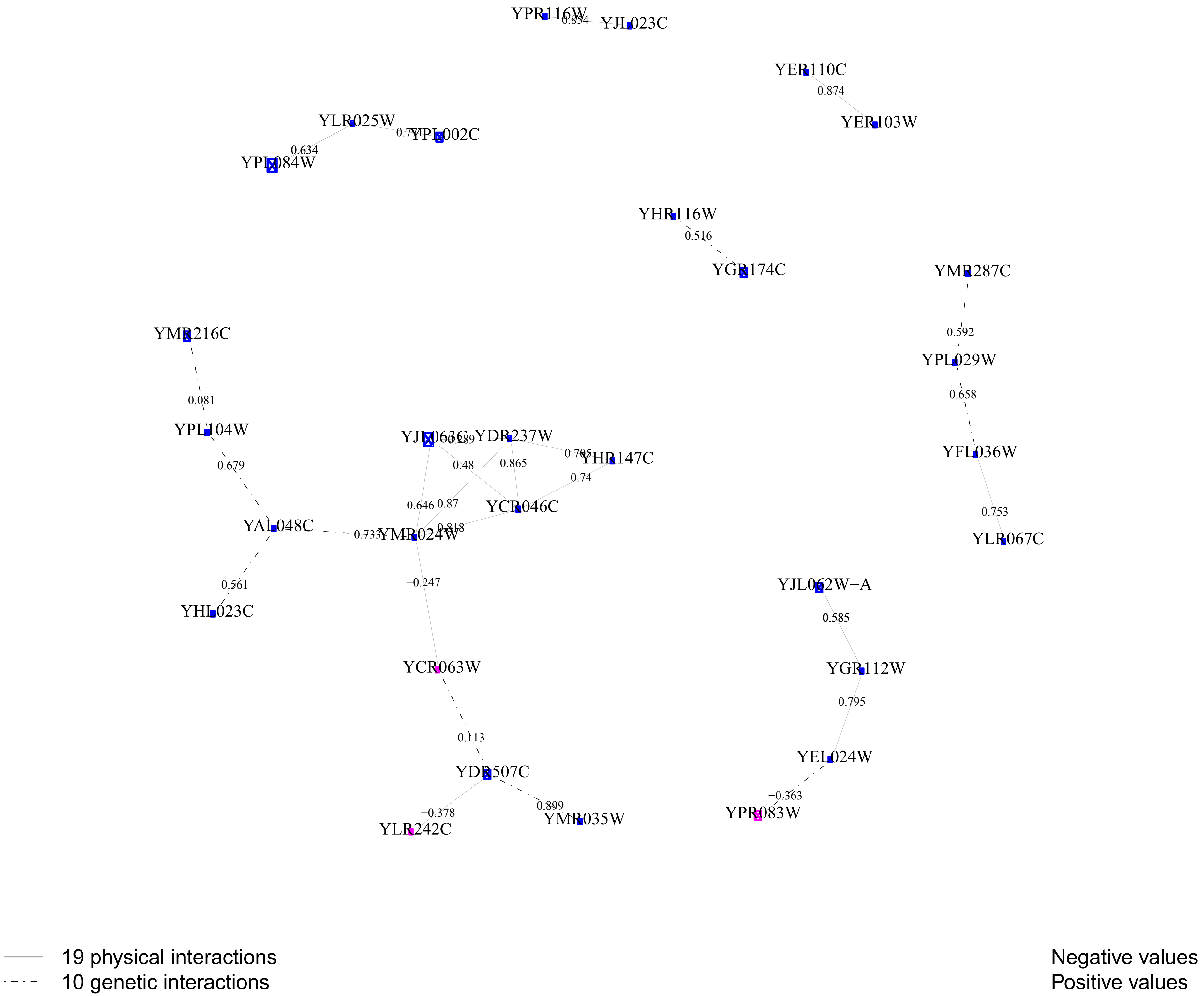

KO Mg : 84 significant mutants, 36 nodes, 96 edges

Number of significant elements in a mutant is between 1 and 6 .

Edge value is the correlation between the all elements profile of two mutants.

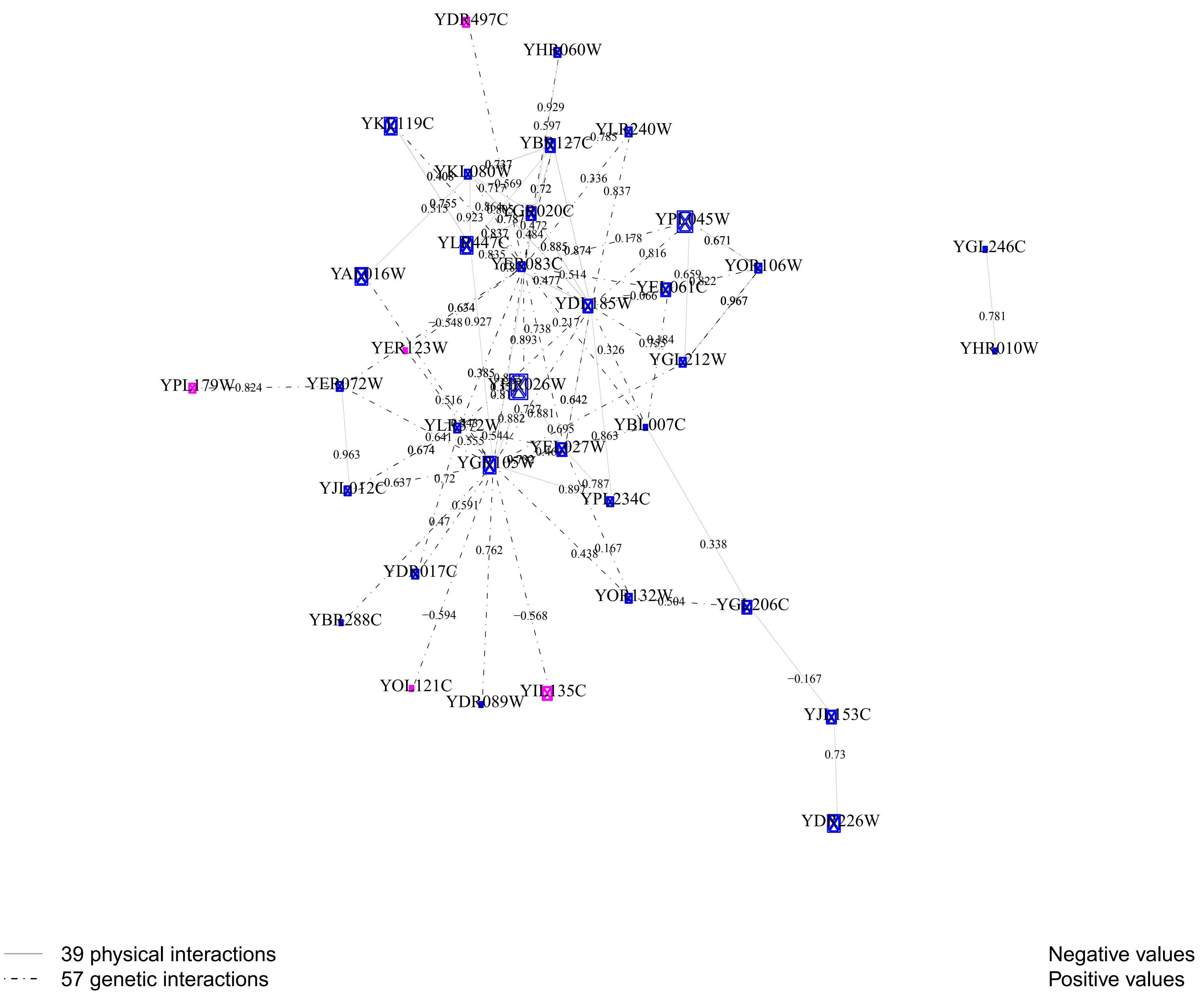

KO Mn : 66 significant mutants, 24 nodes, 38 edges

Number of significant elements in a mutant is between 1 and 6 .
Edge value is the correlation between the all elements profile of two mutants.

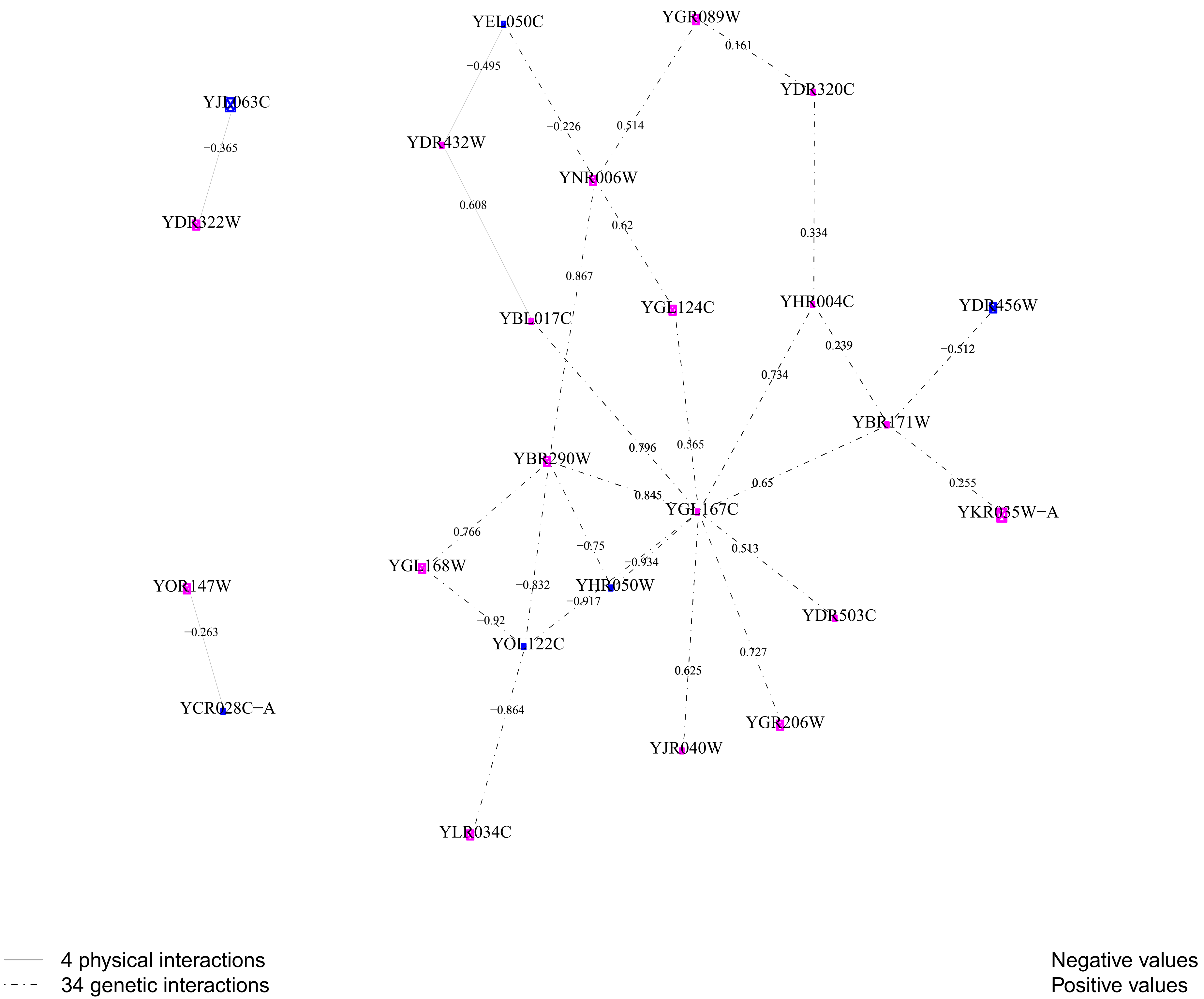

KO Mo : 47 significant mutants, 22 nodes, 39 edges

Number of significant elements in a mutant is between 1 and 6 .
Edge value is the correlation between the all elements profile of two mutants.

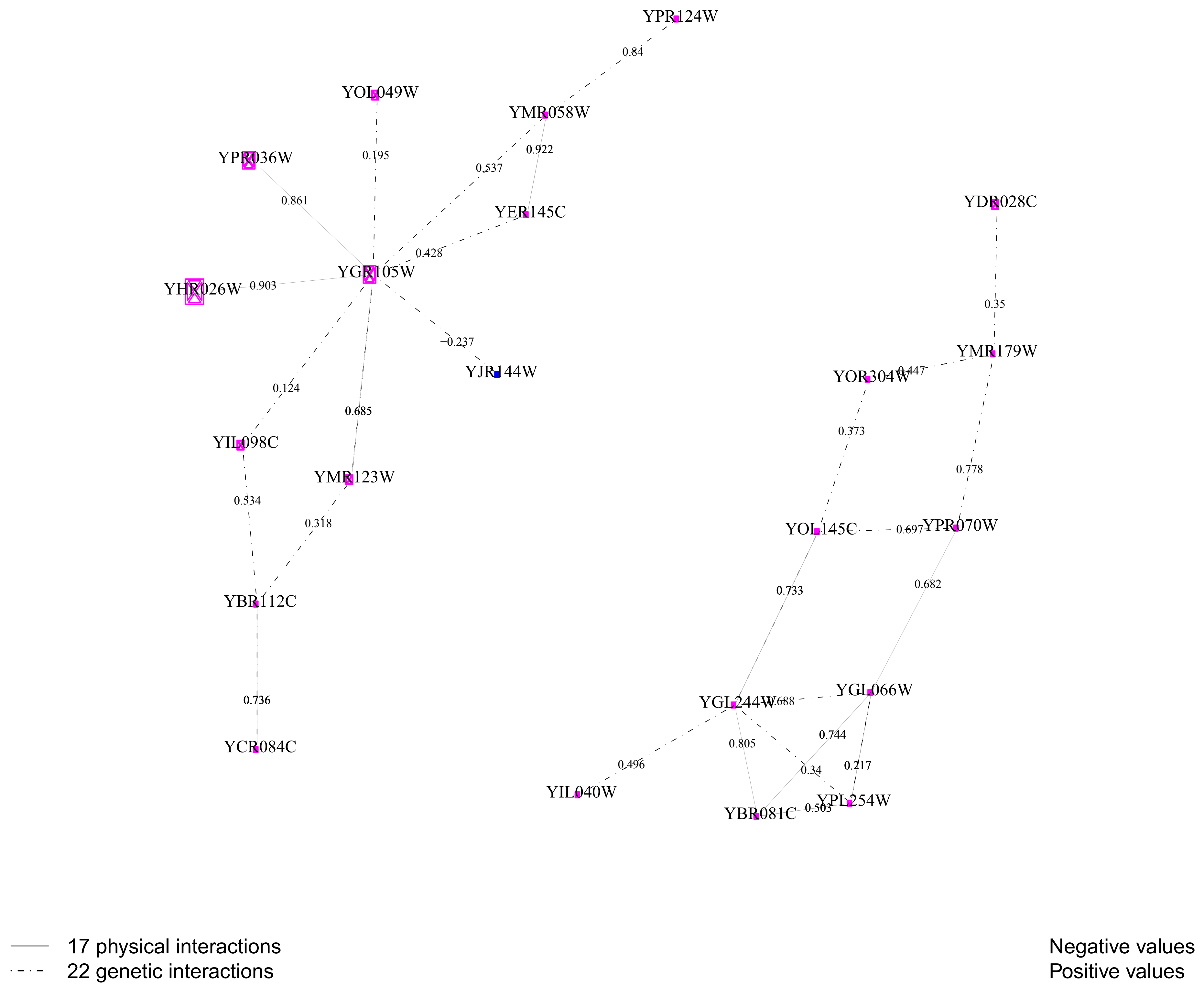

KO Na : 38 significant mutants, 16 nodes, 32 edges

Number of significant elements in a mutant is between 1 and 6 .

Edge value is the correlation between the all elements profile of two mutants.

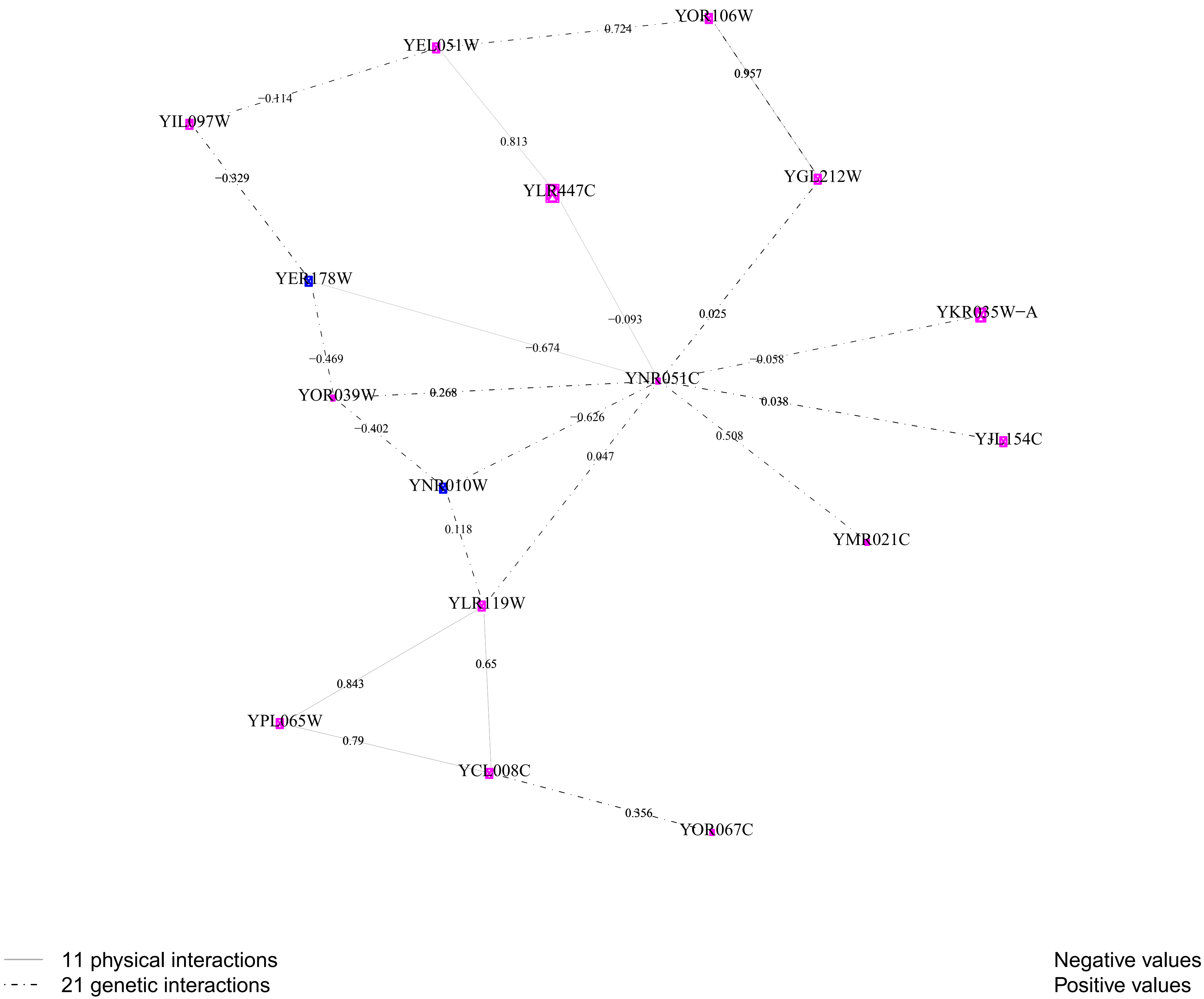

KO Ni : 22 significant mutants, 4 nodes, 3 edges

Number of significant elements in a mutant is between 1 and 3 .
Edge value is the correlation between the all elements profile of two mutants.

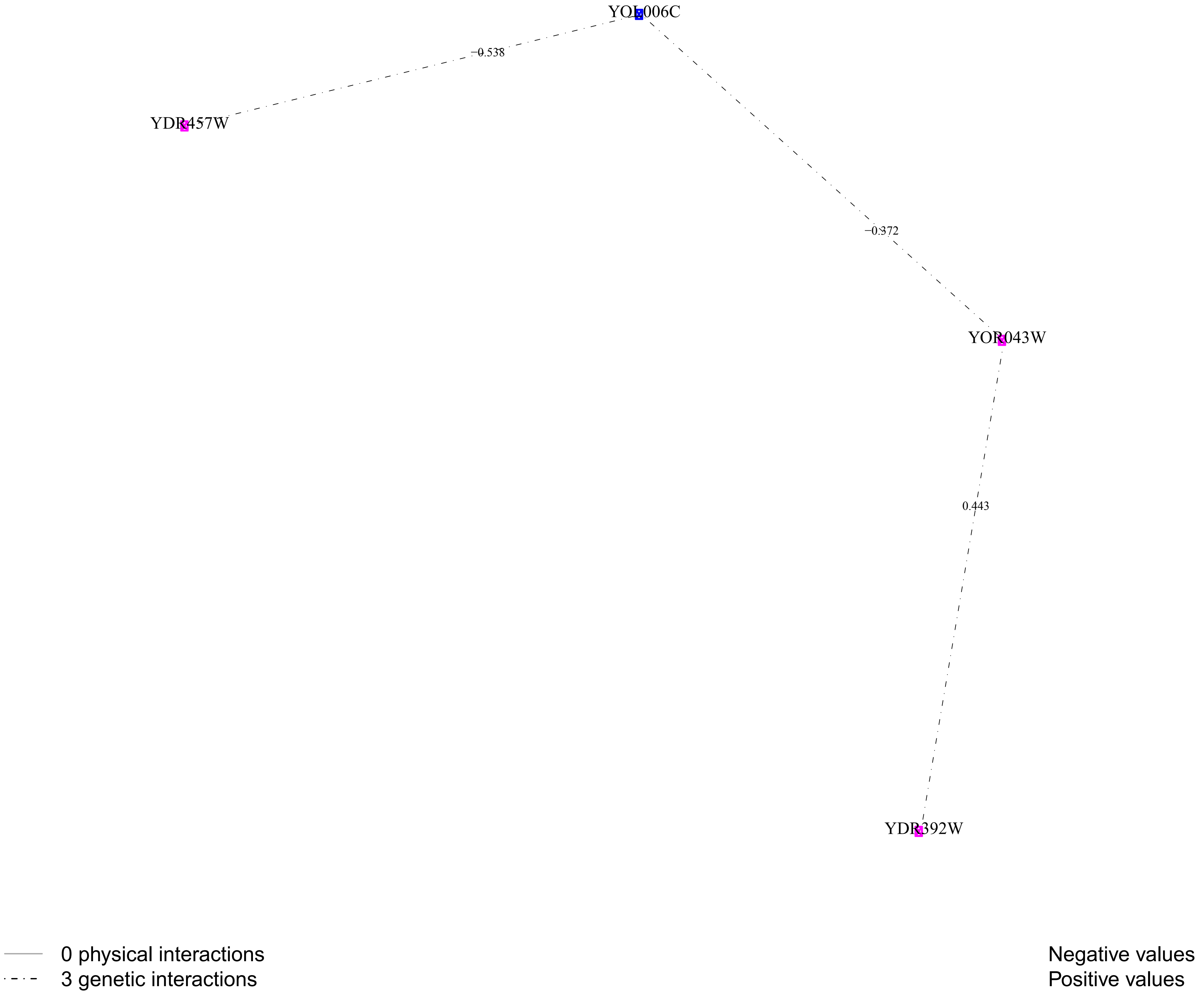

KO P : 58 significant mutants, 26 nodes, 87 edges

Number of significant elements in a mutant is between 1 and 6 .

Edge value is the correlation between the all elements profile of two mutants.

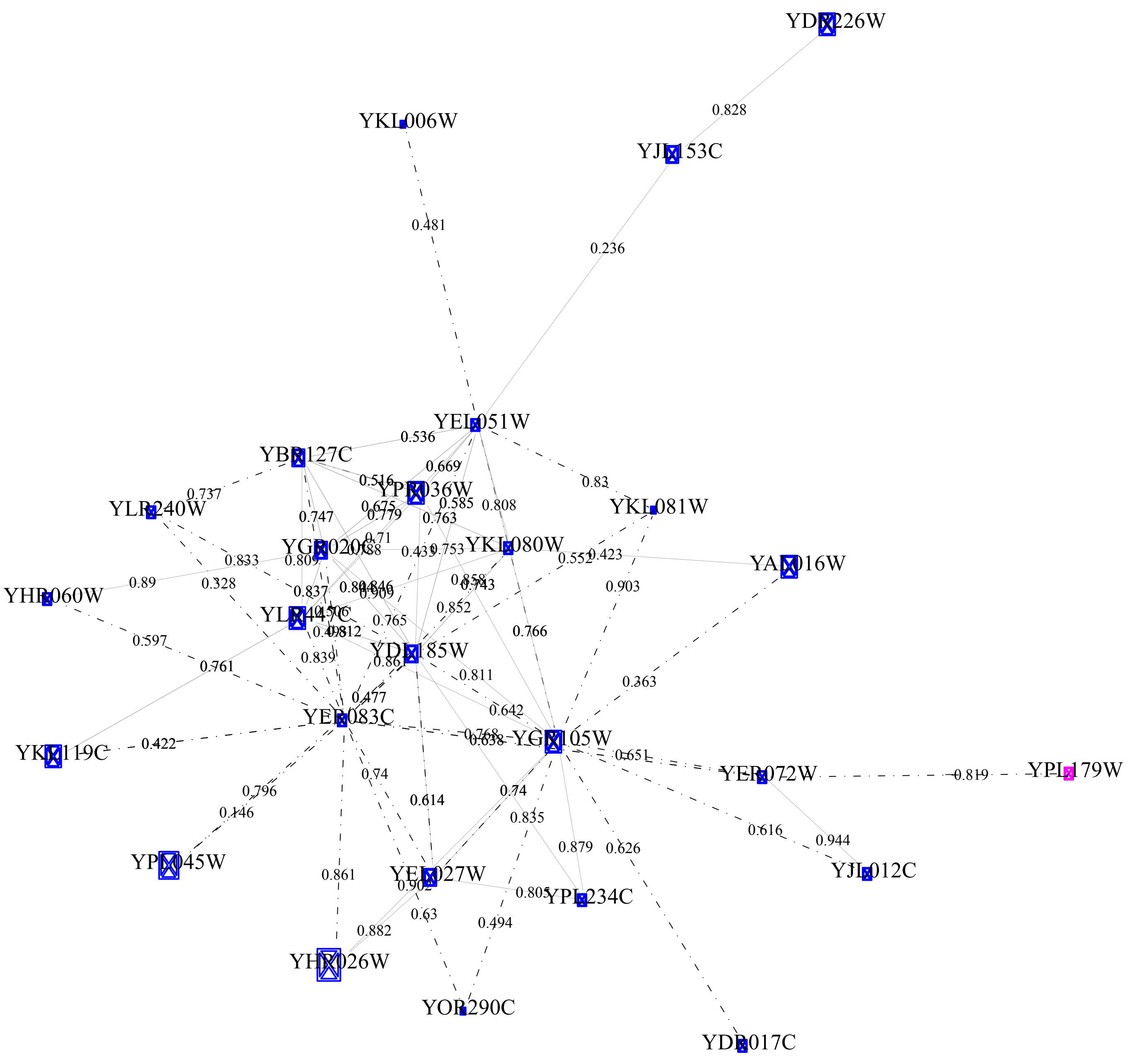

51 physical interactions

36 genetic interactions

Negative values

Positive values

KO S : 39 significant mutants, 4 nodes, 2 edges

Number of significant elements in a mutant is between 1 and 5 .
Edge value is the correlation between the all elements profile of two mutants.

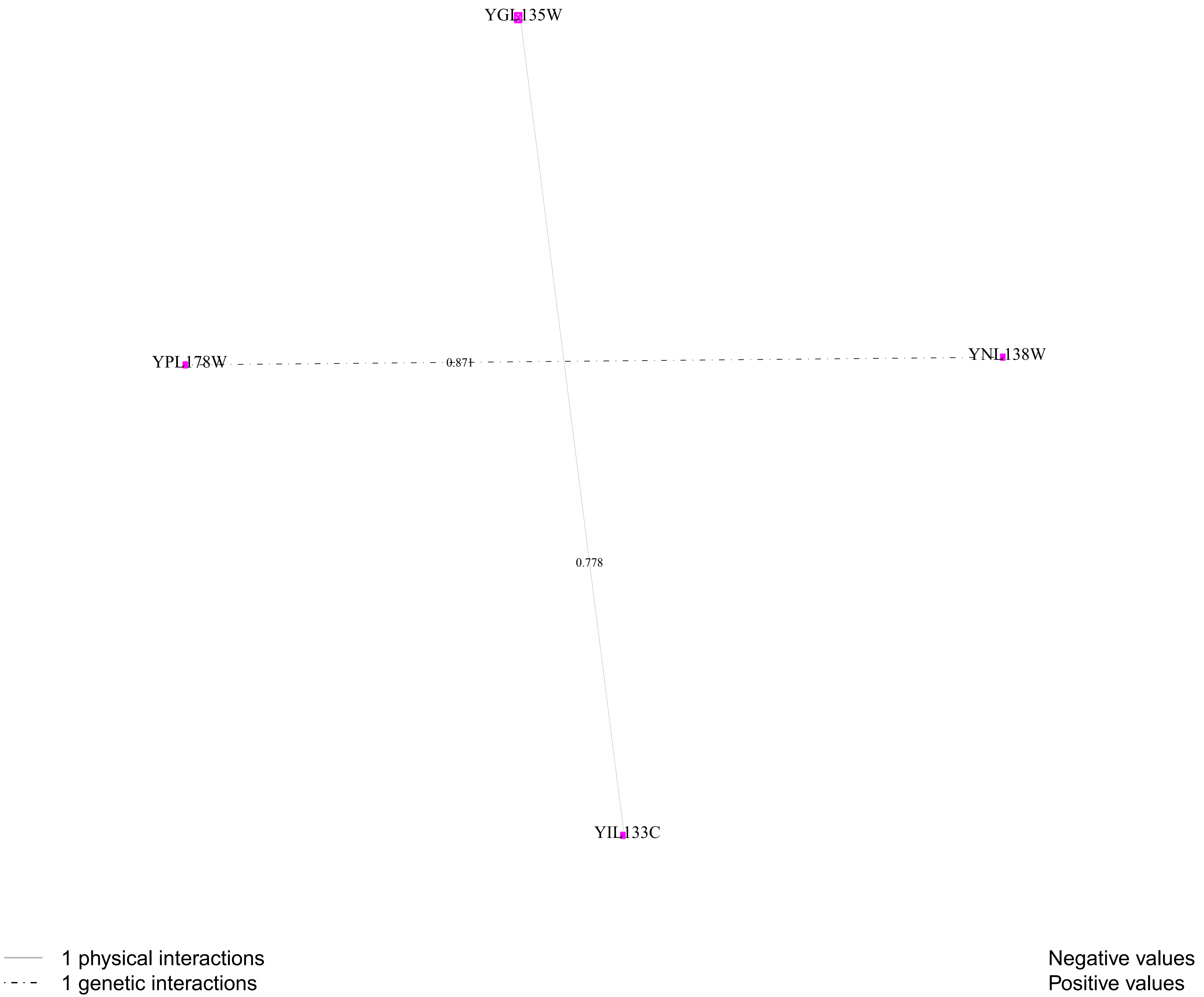

Supplement: Additional file 6: Figure S5 — Visualization of ionomic gene interaction networks for all elements quantified in the ionome of yeast in the knockout collection (KO). For each element quantified in the KO data genes that significantly affect the abundance of that element were selected and an interaction network built based on known protein protein and genetic interactions. Protein protein and genetic interaction information were obtained from BioGRID [48]. Nodes represent genes, node color represents the direction of changes in elemental abundance (magenta increase in abundance, blue decrease in abundance), and node size represents the magnitude of the change in the ionome based on moderated Z-score. Lines joining the nodes (edges) in the graph represent the interactions. The type of line used for the edge represents the type of known interaction between the pairs of genes, with a dotted line representing a genetic interaction, and solid line represents a physical interaction. Numbers on the edges represent the correlation between connected nodes (genes) based on the ionomic profiles of the loss of function mutants in genes represented by the nodes. Only networks are shown with at least 2 nodes and 1 edge. [file 1471-2164-13-623-S6.pdf]
